# Lead Optimization of the 5-Phenylpyrazolopyrimidinone NPD-2975 toward Compounds with Improved Antitrypanosomal Efficacy

**DOI:** 10.1021/acs.jmedchem.3c01976

**Published:** 2024-02-08

**Authors:** Yang Zheng, Magali van den Kerkhof, Mohamed Ibrahim, Iwan J. P. De Esch, Louis Maes, Geert Jan Sterk, Guy Caljon, Rob Leurs

**Affiliations:** † Amsterdam Institute of Molecular and Life Sciences, Division of Medicinal Chemistry, Faculty of Science, Vrije Universiteit Amsterdam, De Boelelaan 1108, Amsterdam 1081 HZ, The Netherlands; ‡ Laboratory of Microbiology, Parasitology and Hygiene (LMPH), University of Antwerp, Universiteitsplein 1, Wilrijk 2610, Belgium

## Abstract

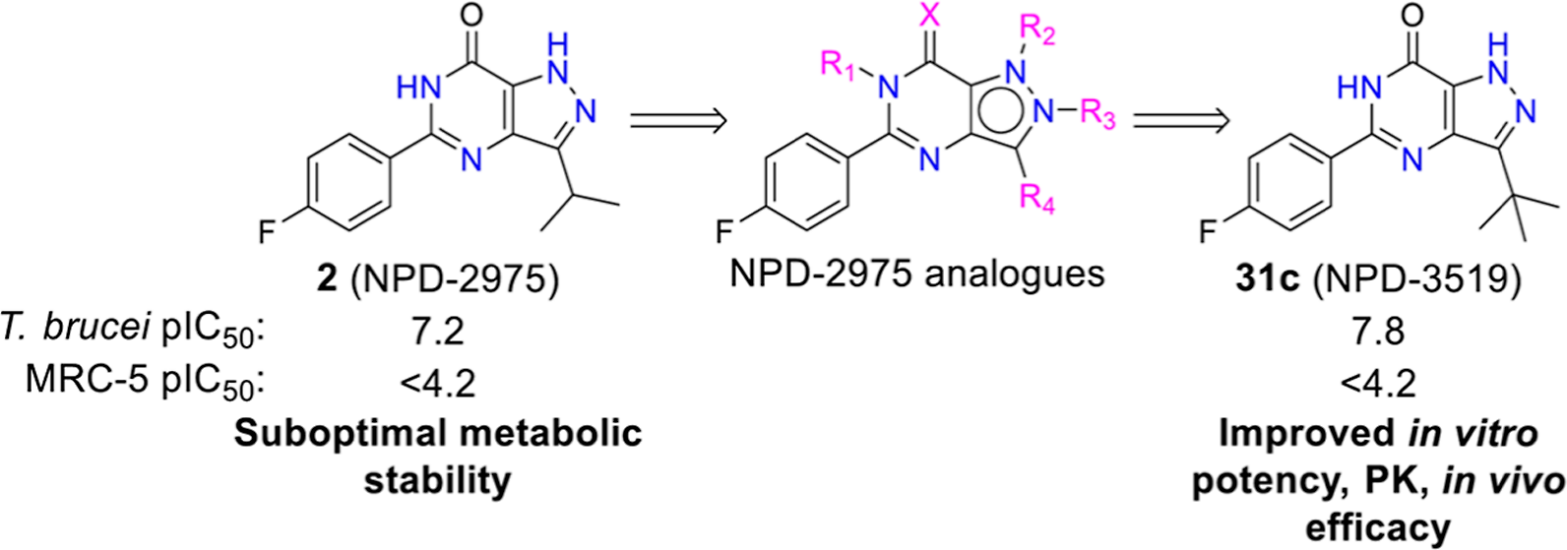

Human African trypanosomiasis (HAT) still faces few therapeutic options and emerging drug resistance, stressing an urgency for novel antitrypanosomal drug discovery. Here, we describe lead optimization efforts aiming at improving antitrypanosomal efficacy and better physicochemical properties based on our previously reported optimized hit NPD-2975 (pIC_50_ 7.2). Systematic modification of the 5-phenylpyrazolopyrimidinone NPD-2975 led to the discovery of a R_4_-substituted analogue **31c** (NPD-3519), showing higher *in vitro* potency (pIC_50_ 7.8) against *Trypanosoma brucei* and significantly better metabolic stability. Further, *in vivo* pharmacokinetic evaluation of **31c** and experiments in an acute *T. brucei* mouse model confirmed improved oral bioavailability and antitrypanosomal efficacy at 50 mg/kg with no apparent toxicity. With good physicochemical properties, low toxicity, improved pharmacokinetic features, and *in vivo* efficacy, **31c** may serve as a promising candidate for future drug development for HAT.

## Introduction

Human African trypanosomiasis (HAT), also known as sleeping sickness, is an infectious disease that mainly occurs in remote and rural areas of sub-Saharan Africa. In 2020, 663 new HAT cases were reported worldwide, most of which were reported in the Democratic Republic of the Congo.^1^ As a member of neglected parasitic diseases (NPDs), there are undoubtedly more patients without diagnosis and proper treatment. To date, people in more than 30 countries are still at risk of contracting HAT.^2^

Two *Trypanosoma brucei* subspecies cause HAT. *T. b. gambiense* causes most of infections (∼95%) in Central and West Africa, while *T. b. rhodesiense* is responsible for infections (5%) in East and Southern Africa.^1^ Infection with *T. b. gambiense* leads to a chronic form of HAT, which can progress for years, while the acute form is caused by *T. b. rhodesiense* with symptoms appearing within weeks of infection.^3−5^ During the first hemolymphatic stage, parasites proliferate in the lymphatic system and cause acute febrile illness.^6^ In the second meningo-encephalitic stage, parasites invade the central nervous system, causing neurological disorders, coma, and eventually death without proper treatment.^7^

Treatment of HAT depends on the parasite subspecies and stages of the disease.^8,9^ Current treatments include pentamidine, melarsoprol, a nifurtimox–eflornithine combination, and fexinidazole.^10,11^ However, several of them were developed more than 50 years ago, showing low efficacy, side effects, and inconvenient administration.^12−15^ Another concern is the emerging drug resistance.^16−18^ Taking the side effects, the population at risk, underreporting, inconvenient administration, and drug resistance into account, there clearly is an urgent need for innovative drug discovery efforts to achieve the goal of HAT eradiation by 2030, as set by the WHO.^19^

One of the goals of the PDE4NPD consortium^20^ aimed to explore novel treatments for four NPDs, *e.g*., HAT, Chagas disease, leishmaniasis, and schistosomiasis. A phenotypic screening strategy was employed for hit discovery next to a structure-based strategy focusing on parasite cyclic nucleotide phosphodiesterases (PDEs). A promising “hit” series has already been reported as a result of the phenotypic screening approach, where the most potent compound NPD-2975 (**2**) showed an IC_50_ of 70 nM against *T. b. brucei* with confirmed *in vivo* efficacy.^21^ Despite high potency both *in vitro* and *in vivo*, one of the drawbacks was its moderate metabolic stability. Taking NPD-2975 (**2**) as a starting point, the present study reports our lead optimization efforts to further improve the antitrypanosomal efficacy and pharmacological/pharmacokinetic profile in the series of 5-phenylpyrazolopyrimidinones.

## Results

### Design Strategy

**Figure 1:**
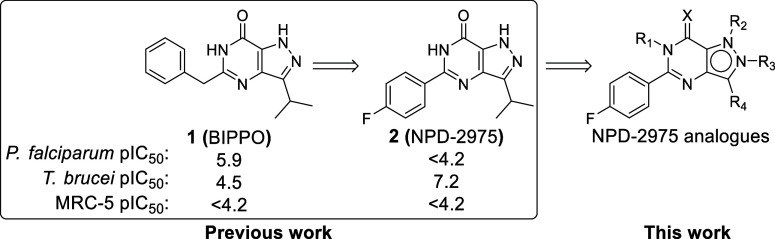
NPD-2975 modification aiming at improved antitrypanosomal activity.

As a modification of the phenyl ring of the 5-phenylpyrazolopyrimidinone scaffold was previously reported, optimization efforts here focused on the other positions of the NPD-2975 scaffold (Figure 1). As an initial step to further understand the structure–activity relationship (SAR) of this scaffold, we introduced methyl groups at R_1_–R_3_ to explore the chemical space around the three nitrogen atoms. Several substituents with various physiochemical properties were introduced to the position where methylation resulted in the highest antitrypanosomal activity (R_2_, Table 1). Meanwhile, to improve solubility and structural diversity, an analogue with an amino group instead of the carbonyl group was prepared. Finally, substituents with different sizes and physicochemical properties were introduced to explore the R_4_ position.

**Table 1:**
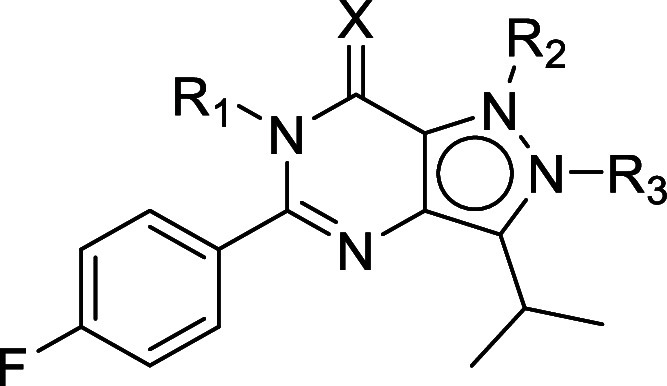
*In Vitro* Antitrypanosomal Potency and Cytotoxicity of Analogues **7**, **11**, **15**, and **17**

compound	R_1_	R_2_	R_3_	X	MW	cLogP^a^	tPSA^a^	pIC_50_^b^	
								*T. brucei*	MRC-5
**2**	H	H		O	272.3	3.1	70.1	7.2 ± 0.2	<4.2
**7**	Me	H		O	286.3	2.6	61.4	<5.0	<4.2
**11**	H	Me		O	286.3	3.0	59.3	7.1 ± 0.1	<4.2
**15**	H		Me	O	286.3	3.2	59.3	5.8 ± 0.1	<4.2
**17**	H	H		NH	271.3	3.5	80.5	<5.0	4.6 ± 0.0

a cLogP and tPSA are adopted from the Collaborative Drug Discovery (CDD) Vault.

b Mean values ± standard deviations, *n* ≥ 2.

### Chemistry

**Scheme 1:**
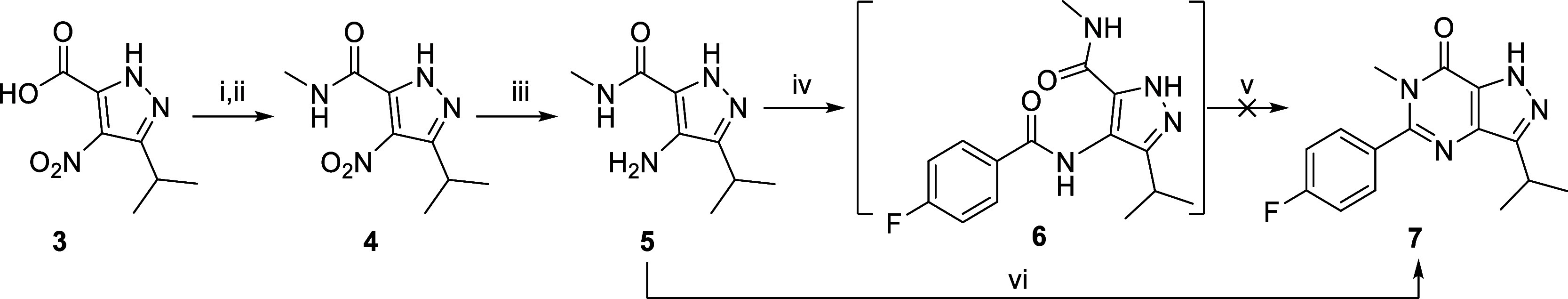
Synthetic Routes of R_1_ Analogue **7** Reagents and conditions: (i) *cat*. DMF, (COCl)_2_, DCM, 0 °C, 1 h, then RT, 2 h; (ii) CH_3_NH_2_, 0 °C to RT, 6 h, 88% over two steps; (iii) 10% Pd/C, H_2_ (g), EtOH, 75 °C, 18 h, 85%; (iv) 4-fluorobenzoic acid, TEA, PyBrop, DCE, MW 120 °C, 20 min; (v) KO^*t*^Bu, ^*i*^PrOH, MW 130 °C; (vi) 4-fluorobenzaldehyde, I_2_, DMF, 80 °C, 16 h, 59%.

**Scheme 2:**
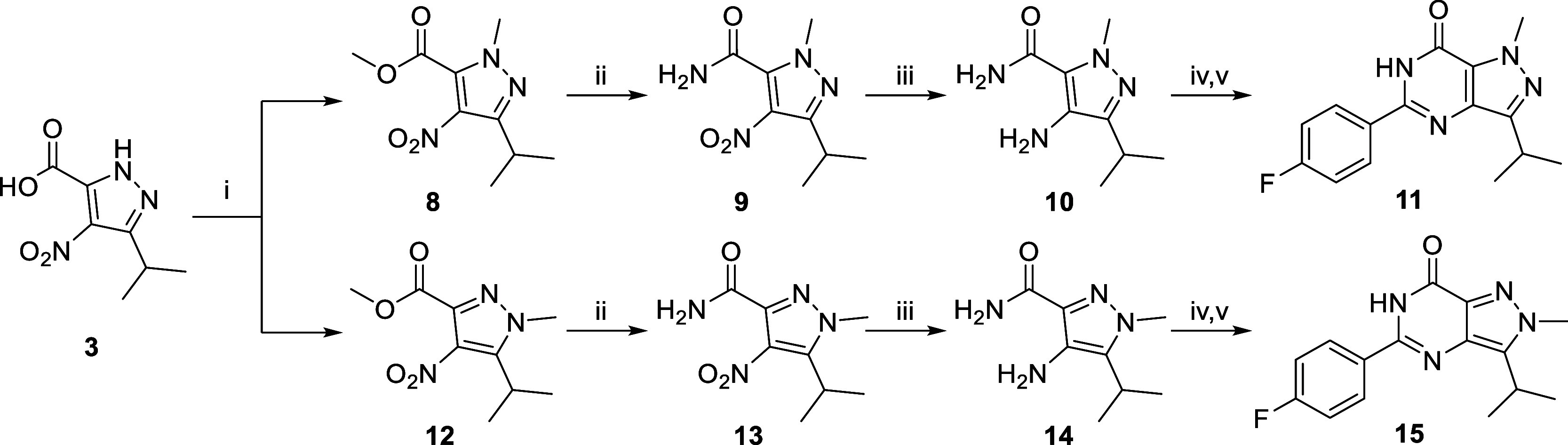
Synthetic Routes of R_2_ Analogue **11** and R_3_ Analogue **15** Reagents and conditions: (i) MeI, K_2_CO_3_, DMF, 60 °C, 1 h, 23% for **8** and 26% for **12**; (ii) 7 M NH_3_ in MeOH, RT, 16 h; (iii) 10% Pd/C, H_2_ (g), EtOH, 60 °C, 16 h, two-step yield 92% for **10** and 96% for **14**; (iv) 4-fluorobenzoic acid, TEA, PyBrop, DCE, MW 120 °C, 20 min; (v) KO^*t*^Bu, ^*i*^PrOH, MW 130 °C, 30 min, two-step yield 73% for **11** and 76% for **15**.

**Scheme 3:**
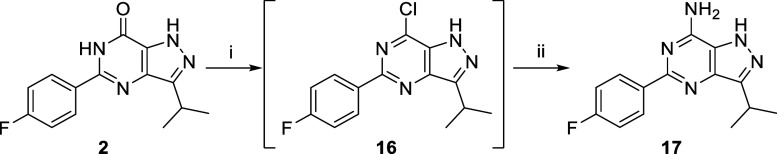
Synthetic Route of Analogue **17** Reagents and conditions: (i) POCl_3_, 120 °C, 1 h; (ii) 0.4 M NH_3_ in THF, MW 120 °C, 28 h, 33% over two steps.

**Scheme 4:**
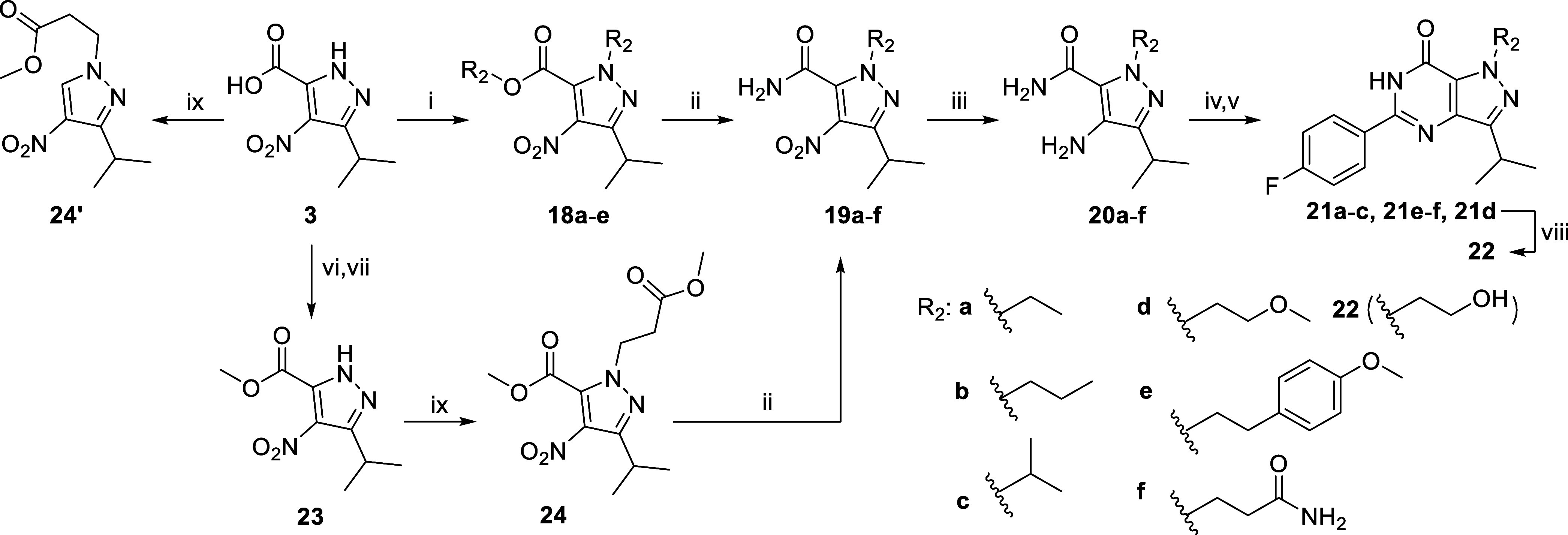
Synthetic Route of R_2_ Analogues **21a**–**f** and **22** Reagents and conditions: (i) R_2_Br, K_2_CO_3_, DMF, 60 °C, 4–16 h; (ii) 7 M NH_3_ in MeOH, RT to 90 °C, 16 h to 2 d; (iii) 10% Pd/C, H_2_ (g), EtOH, 60–75 °C, 16 h; (iv) 4-fluorobenzoic acid, TEA, PyBrop, DCE, MW 120 °C, 20 min; (v) KO^*t*^Bu, ^*i*^PrOH, MW 130 °C, 30 min; (vi) *cat*. DMF, (COCl)_2_, DCM, 0 °C, 1 h, then RT, 2 h; (vii) MeOH, 0 °C, 30 min; (viii) BBr_3_, DCM, −78 °C to RT, 16 h; (ix) CH_3_OCO(CH_2_)_2_Br, K_2_CO_3_, DMF, 60 °C, 16 h.

**Scheme 5:**
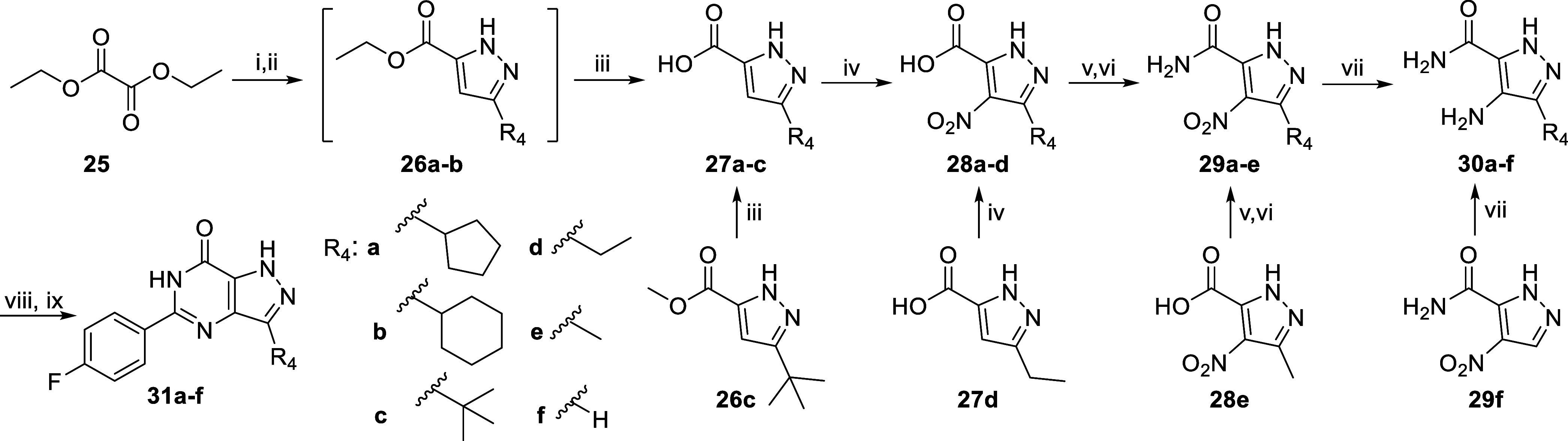
Synthetic Route of R_4_ Analogues **31a**–**f** Reagents and conditions: (i) R_4_COCH_3_, NaOEt, 60 °C, 2 h; (ii) N_2_H_4_·H_2_O, AcOH, EtOH, reflux, 2 h; (iii) NaOH, 1,4-dioxane/H_2_O, RT, 20–23 h; (iv) 65% HNO_3_, *conc*. H_2_SO_4_, 60 °C, 3–4 h; (v) *cat*. DMF, (COCl)_2_, DCM, 0 °C, 1 h, then RT, 2 h; (vi) 7 M NH_3_ in MeOH, 0 °C to RT, 6 h; (vii) 10% Pd/C, H_2_ (g), EtOH, 60 °C, 6–18 h; (viii) 4-fluorobenzoic acid, TEA, PyBrop, DCE, MW 120 °C, 20 min; (ix) KO^*t*^Bu, ^*i*^PrOH, MW 130 °C, 30 min.

**Scheme 6:**
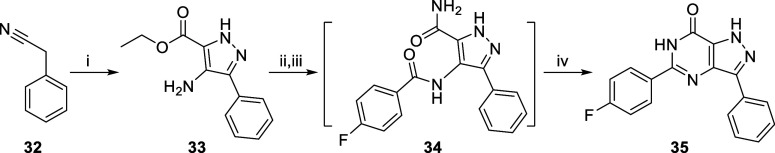
Synthetic Route of Analogue **35** Reagents and conditions: (i) ethyl 2-diazoacetate, NaOEt, toluene 0 °C to RT, 18 h, 20%; (ii) 4-fluorobenzoic acid, TEA, PyBrop, DCE, MW 120 °C, 30 min; (iii) 7 M NH_3_ in MeOH, MW 100 °C, 3 d; (iv) KO^*t*^Bu, ^*i*^PrOH, MW 130 °C, 85 min, 17% over four steps.

The synthesis of intermediates **3**, **14**, **20d**, **23**, **28a**, **28d**, **29d**, **30e**, and **33** has been reported previously.^21−28^ The synthesis of the R_1_-methyl analogue **7** originally started from a direct *N*-methylation of **2**. However, only mixtures of two *N*-methyl regioisomers (**11** and **15**) were obtained after several trials (synthetic conditions not shown). Ultimately, analogue **7** was prepared with the route depicted in Scheme 1. Starting from intermediate **3** (reported in the synthesis of **2**), a methyl amine group was introduced to form the amide **4**. A subsequent reduction with palladium on carbon and hydrogen gas yielded the amino intermediate **5**. The last ring-closure step was initially performed with 4-fluorobenzoic acid, as previously reported.^22,29^ However, reactions with **6** under basic conditions did not work out probably due to the steric hindrance of the extra methyl group. Finally, this ring-closure reaction was completed directly from **5** with 4-fluorobenzaldehyde in DMF with iodine at 80 °C.^30−32^ For the R_2_-methyl analogues, synthesis of an R_2_-*N*-methylated BIPPO (**1**) analogue directly with dimethyl sulfate (DMS) was reported previously, but the synthesis of the corresponding R_3_ analogue was not reported and the regiochemistry of the reaction was not investigated.^29^ Here, we report the synthesis of R_2_ and R_3_ analogues of **2** using a different synthetic route (Scheme 2) with confirmed regiochemistry using 1D-NOESY experiments (Figures S14 and S18). Starting from **3**, introduction of a methyl group with iodomethane yielded two separable regioisomers **8** and 1**2**. Next, the amidation and reduction reactions afforded the key intermediates **10** and **14** without purification of **9** and **13**. The last ring-closure steps were completed with 4-fluorobenzoic acid as reported for **2**.^21^ To improve solubility and structural diversity, an amino group was introduced with the route shown in Scheme 3. Starting with **2**, a chlorination reaction in POCl_3_ yielded **16**, which was further converted to **17** with an ammonia solution. After this initial modification, we focused on the R_2_ position with the highest activity (**7**, **11**, **15**, and **17**) whereby the synthetic route for **11** was applied for the synthesis of R_2_ analogues **21a**-**e** but slightly modified for **21f** (Scheme 4). In the first step of the synthesis of **21f**, introduction of the 3-methoxy-3-oxopropyl group at the R_2_ position with methyl 3-bromopropanoate yielded a decarboxylation side-product **24’**. Thus, an extra step to convert **3** to ester **23** was performed before the pyrazole alkylation reaction. Lastly, our synthetic efforts focused on the R_4_ position. Analogues (**31a**–**f**) with aliphatic substituents of various sizes were prepared (Scheme 5). Due to physiochemical properties, some intermediates in Schemes 4 and 5 were difficult to isolate and were used in the next step without further purification. Phenyl analogue **35** was prepared by a different route (Scheme 6). The first step formed a phenylpyrazole scaffold with benzyl cyanide and ethyl 2-diazoacetate under basic conditions.^33^ After the amide coupling and amidation reactions, **34** was obtained and used for the next step without further purification. The ring-closure reaction of **34** was completed under the same basic conditions as described for the synthesis of **2**.^21^

### *In Vitro* Evaluation against *T. brucei*

In our initial optimization of the 5-phenylpyrazolopyrimidinone **2** (NPD-2975) (Table 1), we investigated the effect of methylation on the three nitrogen atoms and replacement of the carbonyl group with an amino group. The synthesized analogues were tested against *T. b. brucei*, and inhibition of MRC-5 (human lung fibroblasts MRC-5_SV40_) cell proliferation was included as cytotoxicity control. Only the R_2_ methyl analogue **11** showed an equal potency (pIC_50_ 7.1) to **2**. The other two (R_1_ and R_3_) *N*-methyl analogues **7** and **15** exhibited significantly decreased activity (>100-fold and 20-fold, respectively). Analogue **17** with an amino group instead of the carbonyl group was prepared to improve solubility for further modification. However, a >100-fold activity decrease and slight cytotoxicity increase (pIC_50_ 4.6) were observed, discouraging the amino substituents at this position. The R_2_ analogue **11** which displayed the highest antitrypanosomal activity was selected as the basis for further SAR investigations.

The second round of modifications further focused on the R_2_ position, introducing several aliphatic substituents and one aromatic group on this position using the same synthetic route as used for **11** (Scheme 4). To increase diversity and solubility, analogues **21a**–**f** and **22** were synthesized and evaluated *in vitro* (Table 2). A clear trend of decreasing activity (from pIC_50_ 7.1 for **11** to pIC_50_ < 4.2 for **21d** and **21f**) can be correlated with the increasing size of the R_2_ substituent. Analogues **21a** and **21b** with an ethyl and *n*-propyl substituent showed a marginally lower activity (pIC_50_ 6.6 and 6.8) compared to **2**. However, introduction of an isopropyl group at the R_2_ position decreased the antitrypanosomal activity of **21c** (pIC_50_ 5.2) almost 100-fold compared with **2** (Table 2). Introducing an extra oxygen atom in the linker of **21b** to increase flexibility was also detrimental to the activity, and **21d** showed no antitrypanosomal activity at all. All these results indicate limited chemical space around this R_2_ position. Based on these SAR results, **21e** and **22** were prepared to increase the solubility. They exhibited micromolar IC_50_ values (3.2 and 0.8 μM, respectively) against *T. brucei* with lower cLogP values (1.9 and 2.3, respectively) compared with **21a** and **21b** (cLogP 3.4 and 3.9, respectively). Analogue **21f** with a bulky aromatic substituent showed no antitrypanosomal activity as can be expected from the above-described SAR.

**Table 2:**
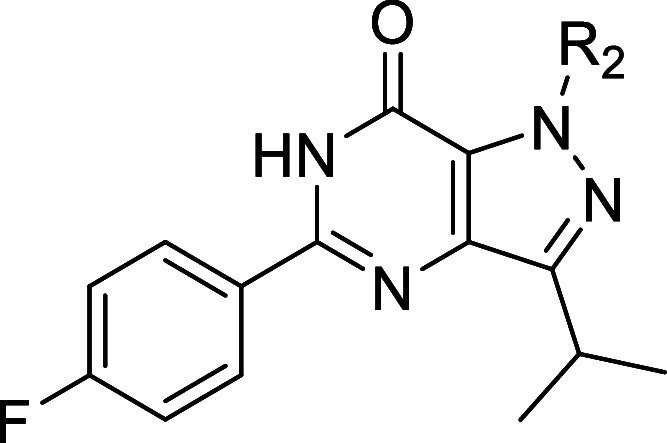
*In Vitro* Antitrypanosomal Activity and Cytotoxicity of R_2_ Analogues

compound	R_2_	MW	cLogP^a^	tPSA^a^	pIC_50_^b^	
					*T. brucei*	MRC-5
**2**	H	272.3	3.1	70.1	7.2 ± 0.2	<4.2
**11**	Me	286.3	3.0	59.3	7.1 ± 0.1	<4.2
**21a**	Et	300.3	3.4	59.3	6.6 ± 0.4	<4.2
**21b**	^*n*^Pr	314.4	3.9	59.3	6.8 ± 0.2	<4.2
**21c**	^*i*^Pr	314.4	3.8	59.3	5.2 ± 0.1	<4.2
**21d**	-(CH_2_)_2_OMe	330.4	3.0	68.5	<4.2	<4.2
**22**	-(CH_2_)_2_OH	316.3	2.3	79.5	6.2 ± 0.1	<4.2
**21e**	-(CH_2_)_2_CONH_2_	343.4	1.9	102.4	5.5 ± 0.3	<4.2
**21f**	-(CH_2_)_2_Ph-4-OMe	404.5	4.9	68.5	<4.2	<4.2

a cLogP and tPSA are adopted from CDD Vault.

b Mean values ± standard deviations, *n* ≥ 2.

Although a few analogues with lower cLogP and antitrypanosomal activity were identified in the R_2_ analogue series, none of them exhibited improved activity combined with better drug-like physiochemical properties. Thus, our last modification focused on the R_4_ position. Based on structure **2**, analogues with a phenyl group and different aliphatic substituents were synthesized and evaluated *in vitro* (Table 3). For analogues with aliphatic substituents at the R_4_ position, a difference in IC_50_ values against *T. brucei* of more than three log units was observed, with the most potent being **31a** (pIC_50_ 8.0) with a cyclopentyl substituent, and the least active being **31e** (pIC_50_ 4.6) with a methyl substitution at R_4_ (Table 3). An increase in antitrypanosomal activity is nicely correlated with the increasing size of R_4_ substituents with a maximum activity for the cyclopentyl group. Analogues (**31d**–**f**) with substituents smaller than an isopropyl group are less potent than **2**. Analogues (**31a** and **31c**) with larger R_4_ substituents exhibit improved potency (6 and 4-fold) compared to **2**. Introduction of a cyclohexyl (**31b**) or phenyl group (**35**) at R_4_ decreased the pIC_50_ values to 7.4 and 7.3, which is 4-fold lower compared to **31a** (Table 3). Due to the very low synthesis yield toward the phenyl analogue (Scheme 6), no more aryl analogues were prepared. It can be concluded that R_4_ is a key position in the 5-phenylpyrazolopyrimidinone structure, and proper substitution can significantly affect the antitrypanosomal activity potential. It also should be noted that none of the R_4_ substituted analogues showed noticeable cytotoxicity.

**Table 3:**
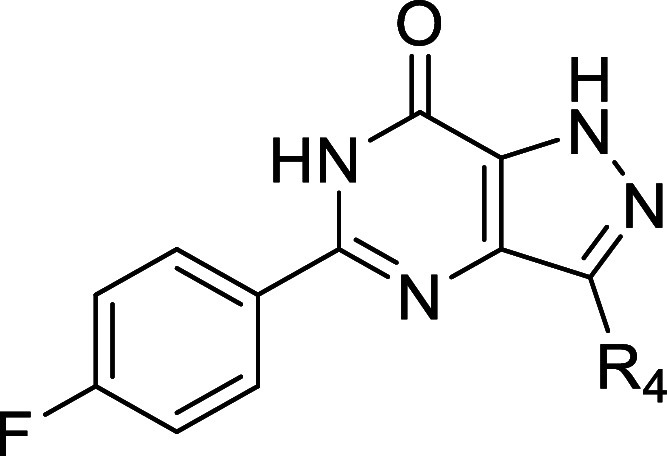
*In Vitro* Antitrypanosomal Activity and Cytotoxicity of R_4_ Analogues

compound	R_4_	MW	cLogP^a^	tPSA^a^	pIC_50_^b^	
					*T. brucei*	MRC-5
**31f**	H	230.2	2.0	70.1	<4.2	<4.2
**31e**	Me	244.2	2.2	70.1	4.6 ± 0.4	<4.2
**31d**	Et	258.3	2.7	70.1	6.4 ± 0.0	<4.2
**2**	^*i*^Pr	272.3	3.1	70.1	7.2 ± 0.2	<4.2
**31c**	^*t*^Bu	286.3	3.4	70.1	7.8 ± 0.3	<4.2
**31a**	cyclopentyl	298.3	3.5	70.1	8.0 ± 0.0	<4.2
**31b**	cyclohexyl	312.3	3.9	70.1	7.4 ± 0.0	<4.2
**35**	Ph	306.3	3.6	70.1	7.3 ± 0.1	<4.2

a cLogP and tPSA are adopted from the CDD Vault.

b Mean values ± standard deviations, *n* ≥ 2.

### Parasite Selectivity Panel and Metabolic Stability

Based on their promising antitrypanosomal activity, analogues **31a** and **31c** were selected for further antiparasitic profiling. First, they were tested *in vitro* against the protozoan species *Trypanosoma cruzi* and *Leishmania infantum* and the clinically relevant *T. b. rhodesiense*. Their potency against *T. b. rhodesiense* was similar to *T. b. brucei* while no activities were observed against *T*. *cruzi* and *L. infantum* as well as no cytotoxicity against MRC-5 cells and peritoneal mouse macrophages (PMM) (Table 4).

**Table 4:** Antiparasitic Profile and Toxicity of **2**, **31a**, and **31c**

compound		pIC_50_^a^				
	*T. b. brucei*	*T. b. rhodesiense*	*T. cruzi*	*L. infantum*	MRC-5	PMM
**2**	7.2 ± 0.2	N.D.	<4.2	<4.2	<4.2	<4.2
**31a**	8.0 ± 0.0	8.0 ± 0.0	<4.2	<4.2	<4.2	<4.2
**31c**	7.8 ± 0.0	8.0 ± 0.0	<4.2	<4.2	<4.2	<4.2

a Mean values ± standard deviations, *n* ≥ 2; PMM: peritoneal mouse macrophages.

**Figure 2:**
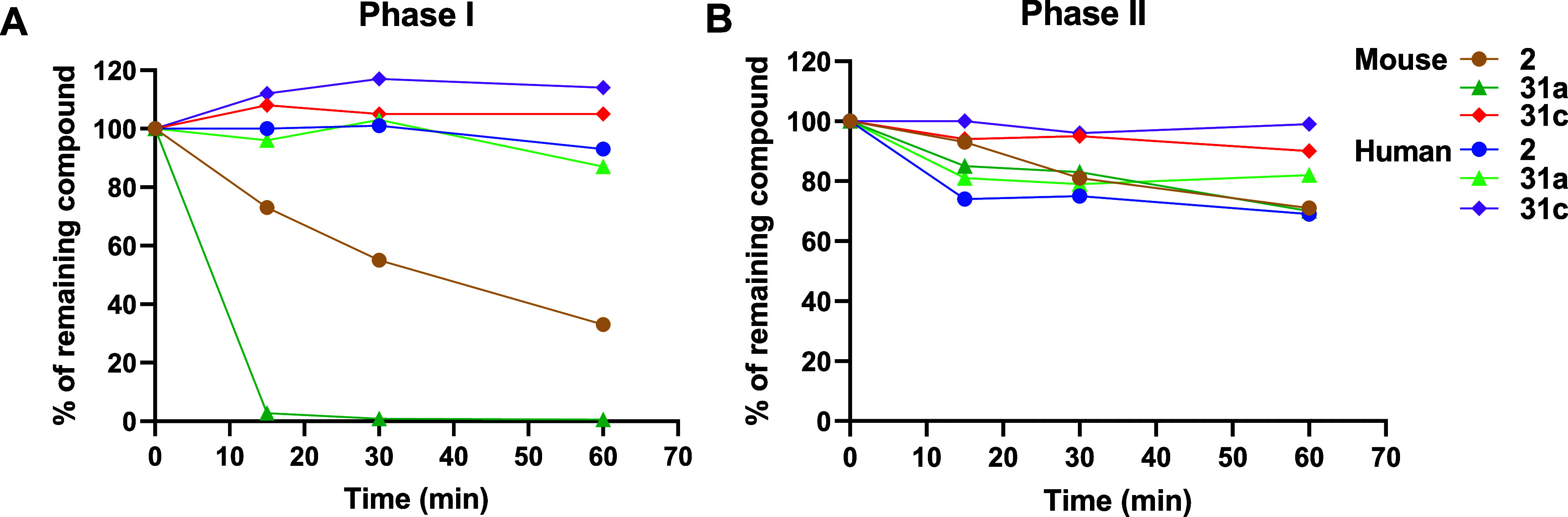
Metabolic stability of **31a** and **31c** in comparison with **2**. (A) Phase-I metabolic stability of **31a** and **31c** in the presence of mouse and human liver microsomes. (B) Phase-II metabolic stability of **31a** and **31c** against mouse and human liver microsomes. Source data are provided in Table S1.

Next, the metabolic stability of **31a** and **31c** was tested in human and mouse liver microsomes and compared with the previously published hit compound **2**. As shown in Figure 2, a significant difference in metabolic stability in the Phase-I metabolism was shown as a result of R_4_ substitution. Analogue **31a** with a cyclopentyl group at R_4_ was metabolized within 15 min by mouse liver microsomal Phase-I metabolism; **31c** with a *tert*-butyl group at R_4_ exhibited improved metabolic stability compared with **2**. No significant metabolism was observed for **31a** and **31c** by human liver microsomal Phase-I metabolism, as was also observed for **2**. For Phase-II metabolism, both R_4_-substituted compounds showed good stability with at least 69% of parent compound remaining after a 1 h incubation in both mouse and human liver microsomes, indicating that the Phase-I metabolism is indeed the main route of metabolism. For the other analogues (**11**, **31b**, and **35**) with pIC_50_ > 7.0, metabolic stability results are summarized in Table S1.

### *In Vivo* Pharmacokinetics

**Figure 3:**
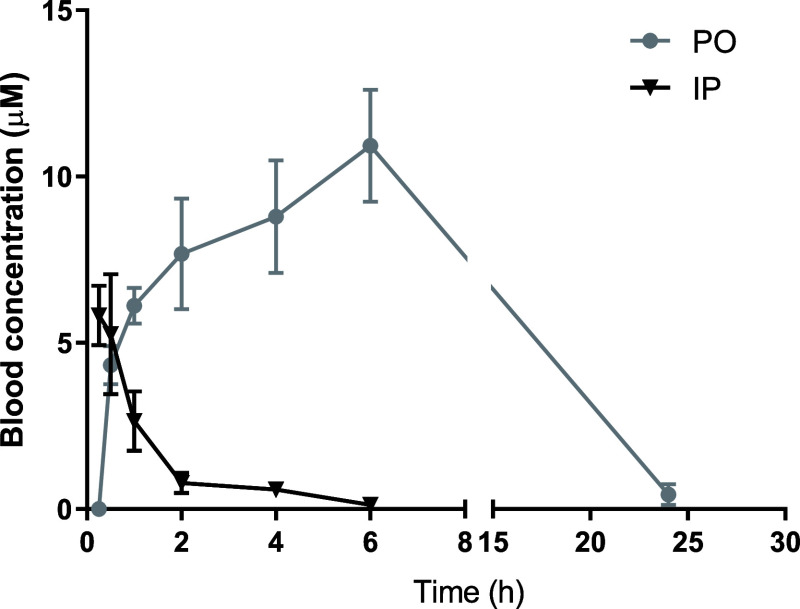
Blood levels (0–24 h) of **31c** in mice (*n* = 3/group) after a single i.p. dose (10 mg/kg) or p.o. dose (50 mg/kg). Results are expressed in mean blood concentration (μM) ± standard error of mean (SEM).

Due to its acceptable *in vitro* metabolic stability, the *in vivo* pharmacokinetic properties of **31c** were measured after either oral (PO) or intraperitoneal (IP) administration (Figure 3), and pharmacokinetic parameters were derived based on the measured blood concentrations (Table 5). Both routes of administration quickly led to micromolar blood concentrations that exceed the *in vitro* IC_50_ value against *T. brucei* by more than 300-fold (Table 3, Figure 3). With regard to metabolic stability, **31c** showed a slightly higher *T*_1/2_ than **2** after IP administration. Whereas the *T*_1/2_ of **31c** and **2** after PO administration were comparable, a more than 7-fold higher AUC_0–6 h_ was observed after PO administration of **31c** compared with **2** (Table 5). Since the oral bioavailability of **31c** was significantly higher, this route of administration was used for subsequent evaluation of antiparasitic efficacy in a mouse model of acute *T. b. brucei* infection. Remarkably, an average concentration of 296 nM **31**c was observed in the mice brain after a 24 h treatment (Table S2), which is more than 18 times of its IC_50_ value against *T. brucei*. This result shows its promising application for the treatment of second-stage HAT in the future.

**Table 5:** Pharmacokinetic Parameters of **31c** in Comparison with **2** in Non-Infected Mice

	compound	*T*_max_ (h)	*C*_max_ (μM)	*T*_1/2_ (h)	AUC_0__–__6 h_ (ng·h/mL)	Cl (mL/min)
**2**	50 mg/kg p.o.	3.0 ± 1.5	5.25 ± 1.99	3.46 ± 1.53	6064 ± 2773	58 ± 38
	10 mg/kg i.p.	0.25 ± 0.0	13.18 ± 0.47	1.06 ± 0.43	3928 ± 199	171 ± 10
**31c**	50 mg/kg p.o.	6.0 ± 0.0	10.93 ± 1.69	3.51 ± 1.34	43,351 ± 5946	11 ± 1
	10 mg/kg i.p.	0.5 ± 0.3	5.05 ± 1.51	1.56 ± 0.50	2472 ± 1561	326 ± 148

### *In Vivo* Evaluation of **31c**

**Figure 4:**
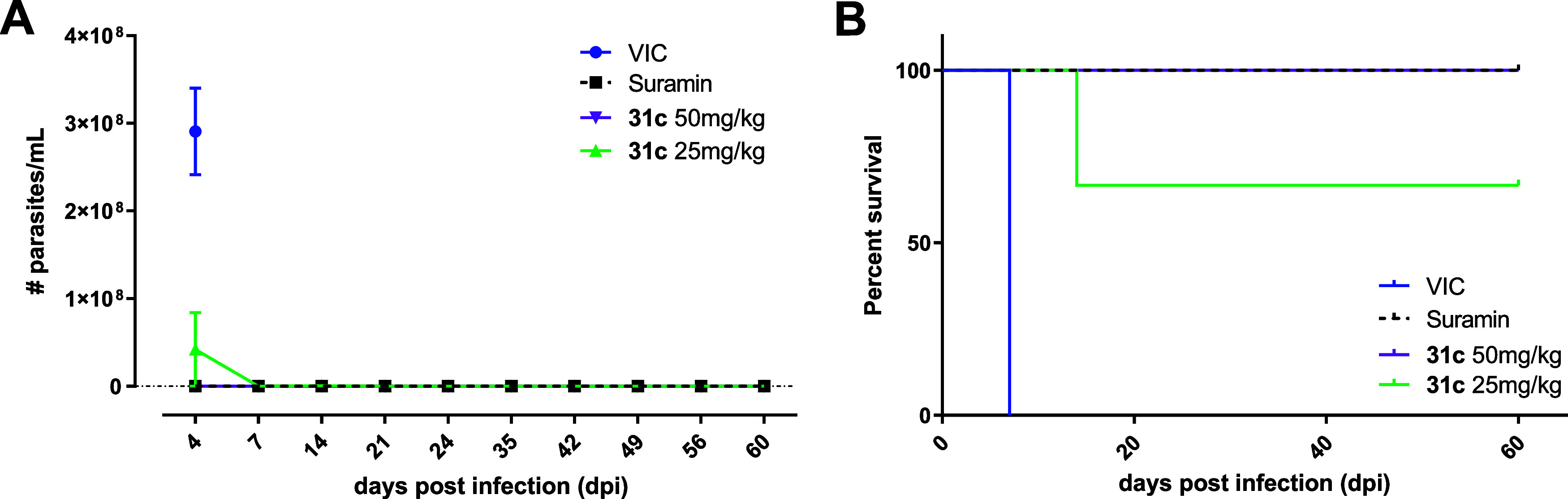
*In vivo* evaluation of **31c** in a stage-I mouse model of HAT. Parasitemia (A) and survival rate (B) of stage-I *T. b. brucei*-infected mice treated with vehicle (*n* = 3), suramin (*n* = 3) at 10 mg/kg or **31c** (*n* = 3) at 50 or 25 mg/kg. Results in figure A are expressed as mean number of bloodstream forms (BSF)/mL ± standard error of mean (SEM). VIC, vehicle.

In our previous *in vivo* results with **2**,^21^ all animals survived until the end of the experiment at 50 mg/kg dose. However, in the group of 25 mg/kg, all animals died at 11 days postinfection (dpi), which could be a result of its moderate metabolic stability. With promising pharmacokinetic parameters, **31c** was evaluated in a mouse model of acute *T. b. brucei* infection and compared to suramin at 10 mg/kg IP once a day (s.i.d.) for 5 days as positive control. Treatment with **31c** at 25 mg/kg and 50 mg/kg twice a day (b.i.d.) PO for 5 consecutive days led to apparent full clearance of parasitemia (Figure 4), with the exception of an accidental death in the 25 mg/kg group. All other animals in the experiment survived throughout the 60 days postinfection follow-up period without relapse, similar to the positive control suramin. The difficulty to detect trypanosome Spliced Leader (SL) RNA by qPCR in blood, spleen, fat, and brain tissue further corroborates the effective clearance of the acute *T. b. brucei* infection by exposure to **31c** (Figure S1). These data indicate a markedly improved *in vivo* potential compared with **2**.^21^

## Discussion

Starting from our previously reported antitrypanosomal hit compound **2** (NPD-2975), lead optimization efforts toward substituted 5-phenylpyrazolopyrimidinones with higher potency and improved physiochemical properties are presented. Systematic modification of the pyrazolopyrimidinone scaffold led to a library of 18 new compounds, with slightly higher molecular weight (average of 298 compared with 272 Da of **2**) and diverse physiochemical properties (cLogP and tPSA). These compounds were tested phenotypically against *T*. *b*. *brucei in vitro*. Our first modification focused on the three nitrogen atoms in the core scaffold of **2**. Substitution reactions on both nitrogen atoms of the imidazole ring yielded two regioisomers, which allowed us to study the influence on the antiparasitic potency of a methyl group at different positions of **2**. The developed synthetic routes can be utilized for lead optimization of this scaffold in the future. Drastic potency differences between analogues with a methyl group at R_1_, R_2_, and R_3_ positions (**7**, **11**, and **15**) guided us to focus on the R_2_ position, which maintained activity with small substituents. Although no potency improvement was observed for compounds with R_2_ substituents after further modification, its tolerance for polar groups can be explored to improve solubility in the future. Modification at the R_4_ position was not synthetically convenient based on the synthetic route for **2**, since for every analogue the R_4_ substituent had to be introduced at the beginning of the synthetic route. However, R_4_ turned out to be a key position for potency. A clear potency improvement is correlated with the increasing size of R_4_ groups up to a cyclopentyl group, with three analogues (**31a**-**c**) showing low nanomolar IC_50_ values (<100 nM) against *T. brucei*. Further replacement of the phenyl group in **35** exhibited comparable antitrypanosomal potency compared with **2**. As follow-up, analogues with pIC_50_ > 7 were tested against human and mouse liver microsomes for their metabolic stability, which was suboptimal for **2**. Remarkable metabolic stability was observed for **31c** (NPD-3519, pIC_50_ 7.8) next to its low toxicity for a number of other protozoan parasites and human cell lines.

As suggested by its metabolic stability, **31c** showed improved pharmacokinetic features, such as longer half-life (1.56 h after IP administration) and more than 7-fold increase in AUC_0–6 h_ after PO dosing compared with **2**. Also, **31c** was detected after 24 h in the brain tissues at significantly higher concentrations than its antitrypanosomal IC_50_. In an acute mouse model of HAT, **31c** yielded full clearance of parasitemia at 25 and 50 mg/kg b.i.d. for 5 days. These results warrant further exploration as drug candidate for HAT. The mode of action of **31c** is still unknown and is currently being investigated next to **2** with a metabolomics approach^34,35^ and an RNAi method, as previously reported.^36^

## Conclusions

To conclude, our lead optimization study starting from the previously reported **2** (NPD-2975) yielded a series of compounds with improved antitrypanosomal potency. Among them, **31c** with a *tert-butyl* group at R_4_ exhibits an IC_50_ of 17 nM against *T. b. brucei* and acceptable metabolic stability. Pharmacokinetic evaluation revealed improved drug-like properties (*T*_1/2_ and AUC). Most importantly, the absence of detectable parasitemia in peripheral blood following an oral dose of 50 mg/kg or 25 mg/kg b.i.d. for 5 days in mice unveiled its promising *in vivo* potential; hence, **31c** could serve as an antitrypanosomal candidate for future drug development against HAT.

## Experimental Section

### *In Vitro* and *In Vivo* Evaluation

All compounds tested are confirmed to pass a publicly available pan-assay interference compounds filter.^37,38^ The antiparasitic assays were carried out exactly as described in Blaazer *et al.*(39) Metabolic stability, pharmacokinetics, acute mouse infection model results were collected as described previously.^21^ All animal experiments were conducted in compliance with institutional guidelines and following approval by the Ethical Committee of the University of Antwerp, Belgium [UA-ECD 2014–96]. Female Swiss mice (15–20 g), were purchased from Janvier (Le Genest Saint Isle, France). The PK properties of two compounds (**2** and **31c**) were compared after a single 10 mg/kg intraperitoneal (IP) or 50 mg/kg oral (PO) dose in uninfected mice (*n* = 3/group, 12 mice in total). A total of 12 mice were used to evaluate the *in vivo* potency of **31c** at two doses, including a vehicle and reference (Suramin) control group. Animals were treated PO b.i.d. for 5 days at 25 and 50 mg/kg **31c**. For the SL-RNA qPCR experiments blood was collected sublingually and subjected to erythrocyte lysis before extracting RNA with the QIAamp RNA Blood Mini kit (Qiagen). The mice were sedated after blood sampling with a mixture of ketamine and xylazine, allowing extensive perfusion to eliminate blood contamination in other tissues. Small pieces of fat, brain, and spleen tissue were excised and immediately transferred to RNA later (Qiagen) and incubated overnight at 4 °C. RNA extraction was performed as instructed in the RNeasy Mini Plus Kit manual (Qiagen). The one-step SensiFAST SYBR Hi-Rox PCR kit (Bioline USA Inc., *via* Gentaur Belgium BVBA, Kampenhout, Belgium) was used for the PCR with the following forward and reverse primers, respectively 5′-AACTAACGCTATTATTAGAA-3′ and 5′-CAATATAGTACAGAAACTG-3′. An initial activation step of 10 min at 45 °C and 10 min at 95 °C was used, followed by the amplification step for 40 cycles (15 s at 95 °C, 15 s 50 °C and 15 s at 60 °C). At last, the melting curves were generated with an increment of 0.3 °C (15 s at 95 °C 1 min at 45 °C and 15 s at 95 °C). The PCR was run on the Step One Plus real-time PCR system (Applied Biosystems, California, USA). An additional qPCR was performed with the mouse housekeeping gene Eef2 to confirm successful RNA extraction.

### Parasite and Cell Cultures

*In vitro* experiments were carried out with the bloodstream form of the *T. b. brucei* Squib strain (suramin-sensitive). Parasites were routinely cultured in T25 culture flasks containing 10 mL of HMI-9 medium (Invitrogen) supplemented with 10% heat-inactivated fetal bovine serum (Life Technologies) and 2.5 μg/mL Geneticin (Life Technologies). MRC-5_SV2_ cells were cultured in MEM + Earl’s salts-medium, supplemented with 2 mM l-glutamine, 16.5 mM NaHCO_3_, and 5% inactivated fetal calf serum. All cultures and assays were conducted at 37 °C under an atmosphere of 5% CO_2_.

### *Trypanosoma* Susceptibility Assay

The compound stock solutions in 100% DMSO at 20 mM were first 4-fold serially diluted in DMSO and next in water to obtain a highest in-test compound concentration of 64 μM and of DMSO not exceeding 1%. Parasites were counted in a KOVA counting chamber and diluted to 1.5 × 10^4^ parasites/well of a 96-well plate (200 μL total volume), upon which the prediluted test compounds were added. Drug exposure covered a 72 h period without renewal of the culture medium. After 3 days of incubation, parasite growth was assessed fluorimetrically after addition of 50 μL of resazurin per well. After 6 h (*T. b. rhodesiense*) or 24 h (*T. b. brucei*) at 37 °C, fluorescence is measured (λ_ex_ 550 nm, λ_em_ 590 nm). Parasite viability was assessed using the resazurin viability assay, and drug activity was calculated as percentage viability reduction compared to nontreated controls. Results were used to determine the 50% inhibitory concentration (IC_50_).

### MRC-5_SV2_ Cytotoxicity Assays

Assays are performed in sterile 96-well microtiter plates, each well containing 10 μL of the watery compound dilutions together with 190 μL of MRC-5_SV2_ inoculum (1.5 × 10^5^ cells/mL). Cell growth is compared to untreated-control wells (100% cell growth) and medium-control wells (0% cell growth). After 3 days of incubation, cell viability is assessed fluorimetrically after addition of 50 μL of resazurin per well. After 4 h at 37 °C, fluorescence is measured (λ_ex_ 550 nm, λ_em_ 590 nm). The results are expressed as % reduction in cell growth/viability compared to control wells and an IC_50_ and an IC_90_ (50 and 90% inhibitory concentrations) are determined.

### Pharmacokinetics

Compounds **2** and **31c** were evaluated for their pharmacokinetic properties after a single 10 mg/kg intraperitoneal (IP) or 50 mg/kg oral (PO) dose in uninfected mice. Blood drops were sampled before treatment and at 0.5, 1, 2, 4, 6, and 24 h after PO dosage; samples after IP dosage were identical with an additional time point of 0.25 h. The blood drops were analyzed adopting the dry blood spot technique and analysis by LC-MS^2^. Briefly, blood was collected from the retro-orbital complex using capillary tubes and dropped (15 μL) on WhatmanFTADMPK cards (B). The spots were left to air-dry at room temperature for at least 2 h. For analysis, a 6 mm disk was punched out and extracted in 75:25 MeCN/water containing the internal standard tolbutamide. The amount of parent compound was determined using liquid chromatography (UPLC) (Waters Aquity) coupled with tandem quadrupole mass spectrometry (MS^2^) (Waters Xevo) equipped with an electrospray ionization (ESI) interface and operated in multiple reaction monitoring mode. Standard curves in whole blood were made for calibration and validation. Standard PK parameters were determined using Topfit software.

Brain tissue of the animals was collected on ice at autopsy 24 h post-treatment (50 mg/kg oral dose) after perfusion. For perfusion, mice were sedated with ketamine/xylazine allowing transcardial perfusion with 10 mL of KREBS Henseleit solution (Sigma-Aldrich) to eliminate blood contamination in the tissues. The tissues were immediately homogenized using a GentleMacs tissue homogenizer. The tissue samples were subjected to protein precipitation by adding MeCN, followed by a centrifugation step at 4 °C for 5 min at 21, 130 g. The supernatant was further diluted in 75:25 MeCN/water for LC-MS^2^ analysis as described above.

### Acute Mouse Model

Mice were allocated to groups of three and were infected by IP injection of 10^4^*T. brucei* Squib 427 trypomastigotes. Compounds **2** and **31c** were formulated in PEG_400_ at 12.5 and 6.25 mg/mL envisaging a maximal dosing volume of 100 μL/25 g live body weight. Suramin was included as a reference for *T. brucei* and injected IP s.i.d. for 5 days at 10 mg/kg. A PEG_400_ vehicle control group was also included. Compounds **2** and **31c** were administered PO b.i.d. for 5 days at 25 and 50 mg/kg. The first treatment was given 30 min prior to the artificial infection. Drug efficacy was evaluated by microscopic determination of the parasitemia in a blood drop collected from the tail vein at several time points until 63 days postinfection (dpi). Animals were observed for the occurrence/presence of clinical or adverse effects during the experiment. An SL-RNA qPCR assay was performed in all surviving animals to confirm parasitological cure. For the SL-RNA qPCR, peripheral blood was subjected to erythrocyte lysis before extracting RNA with the QIAamp RNA Blood Mini kit (Qiagen). The mice were sedated after blood sampling with a mixture of ketamine and xylazine, allowing extensive perfusion to eliminate blood contamination in other tissues. Small pieces of fat, brain, and spleen tissues were excised and immediately transferred to RNA later (Qiagen) and incubated overnight at 4 °C. RNA extraction was performed as instructed in the RNeasy Mini Plus Kit manual (Qiagen). The one-step SensiFAST SYBR Hi-Rox PCR kit (Bioline USA Inc., *via* Gentaur Belgium BVBA, Kampenhout, Belgium) was used for the PCR with the following forward and reverse primers, respectively, 5′-AACTAACGCTATTATTAGAA-3′ and 5′-CAATATAGTACAGAAACTG-3′. An initial activation step of 10 min at 45 °C and 10 min at 95 °C was used, followed by the amplification step for 40 cycles (15 s at 95 °C, 15 s 50 °C, and 15 s at 60 °C). At last, the melting curves were generated with an increment of 0.3 °C (15 s at 95 °C, 1 min at 45 °C, and 15 s at 95 °C). The PCR was run on the Step One Plus real-time PCR system (Applied Biosystems, California, USA). An additional qPCR was performed with the mouse housekeeping gene Eef2 to confirm successful RNA extraction.

### Chemistry

#### General Information

All starting materials were obtained from commercial suppliers and used without purification. Preparation of **2** and **3** has been reported previously.^21^ Anhydrous THF, DCM, and DMF were obtained by passing through an activated alumina column prior to use. All reactions were carried out under a nitrogen atmosphere unless mentioned otherwise. TLC analyses were performed using Merck F_254_ aluminum-backed silica plates and visualized with 254 nm UV light. Flash column chromatography was executed using Biotage Isolera equipment. All HRMS spectra were recorded on a Bruker microTOF mass spectrometer using ESI in positive-ion mode. Nuclear magnetic resonance (NMR) spectra were determined with a Bruker Avance II 300 MHz, a Bruker Avance II 500 MHz or a Bruker Avance III HD 600 MHz spectrometer. Chemical shifts are reported in parts per million (ppm) against the reference compound using the signal of the residual nondeuterated solvent (CDCl_3_ δ = 7.26 ppm (^1^H), δ = 77.16 ppm (^13^C); DMSO-*d*_6_ δ = 2.50 ppm (^1^H), δ = 39.52 ppm (^13^C)). NMR spectra were processed using MestReNova 14.0 software. The peak multiplicities are defined as follows: s, singlet; d, doublet; t, triplet; q, quartet; dd, doublet of doublets; ddd, doublet of doublets of doublets; dt, doublet of triplets; dq, doublet of quartets; td, triplet of doublets; tt, triplet of triplets; qd, quartet of doublets; p, pentet; dp, doublet of pentets; br, broad signal; m, multiplet. For NMR listings, in addition to specific instructions that are given by the journal in the guidelines for authors, the following additional procedures were used: (1) multiplicity is not solely reported based on peak shapes, but it also distinguishes the coupling to all nonequivalent protons that have similar *J* values; (2) if additional smaller couplings are observed but are too small for accurate quantitation because the precision is smaller than the digital resolution, a symbol ^Δ^ will be used; (3) the notation “m” is used in case of obscured accurate interpretation as a result of (i) overlapping signals for different protons or (ii) a result of overlapping signal lines within the same proton signal; (4) for any rotamers or diastereomers, signals will be listed separately; (5) NMR signals that could only be detected with HSQC analysis are denoted with a ^#^ symbol; (6) NMR signals that could only be detected with HMBC analysis are denoted with a * symbol; (7) signals for exchangeable proton atoms (such as NH and OH groups) are only listed if clearly visible (excluding *e.g*. the use of D_2_O or CD_3_OD) and if confirmed by a D_2_O shake and/or HSQC. Note: not all ^13^C signals are visible in the spectra due to the tautomerism of non-*N*-substituted pyrazoles. HSQC and HMBC were measured to assign ^13^C signals if applicable. IUPAC names were adapted from ChemBioDraw Ultra 19.0. Purities were measured using analytical LC-MS using a Shimadzu LC-20AD liquid chromatography pump system with a Shimadzu SPDM20A diode array detector with the MS detection performed with a Shimadzu LCMS-2010EV mass spectrometer operating in positive ionization mode. The column used was an Xbridge (C18) 5 μm column (100 mm × 4.6 mm). The following solutions are used for the eluents. Solvent A: H_2_O/HCOOH 999:1 and solvent B: MeCN/HCOOH 999:1. The eluent program used is as follows: flow rate: 1.0 mL/min, start with 95% A in a linear gradient to 10% A over 4.5 min, hold 1.5 min at 10% A, in 0.5 min in a linear gradient to 95% A, hold 1.5 min at 95% A, total run time: 8.0 min. Compound purities were calculated as the percentage peak area of the analyzed compound by UV detection at 254 nm. All final compounds are >95% pure by HPLC analysis.

### 3-Isopropyl-*N*-methyl-4-nitro-1*H*-pyrazole-5-carboxamide (**4**)

Oxalyl chloride (6.59 mL, 75.0 mmol) was added dropwise to a suspension of **3** (5.00 g, 25.1 mmol) in THF (100 mL) containing DMF (0.119 mL, 1.53 mmol) at 0 °C. The reaction was stirred at 0 °C for 1 h, allowed to warm to RT, and stirred for a further 2 h. The reaction mixture was added to 33% ethylamine in EtOH (9.38 mL, 75.3 mmol) dropwise at 0 °C and stirred at RT for 18 h. After evaporation, the reaction residue was purified by flash column chromatography on silica gel with a gradient elution of MeOH in DCM (0–10%) to get the title compound as a white solid (4.70 g, 88%). ^1^H NMR (300 MHz, DMSO-*d*_6_) δ 13.87 (br s, 1H), 8.52 (s, 1H), 3.54 (hept, *J* = 7.0 Hz, 1H), 2.75 (d, *J* = 4.7 Hz, 3H), 1.29 (d, *J* = 7.0 Hz, 6H). ^13^C NMR (151 MHz, DMSO-*d*_6_) δ 160.9, 150.7*, 128.7, 26.3, 25.6, 21.3. LC-MS: *t*_R_ = 2.77 min, purity: >99%, *m*/*z* [M + H]^+^: 213.

### 4-Amino-3-isopropyl-*N*-methyl-1*H*-pyrazole-5-carboxamide (**5**)

The suspension of 10% palladium on carbon (1.00 g) and **4** (4.70 g, 22.2 mmol) in EtOH (50 mL) was heated at 75 °C with a H_2_ gas insert for 18 h. The reaction mixture was filtered through Celite, concentrated *in vacuo*, and purified by flash column chromatography on silica gel with a gradient elution of EtOAc in cyclohexane (50–100%) to get the title compound as a white solid (3.45 g, 85%). ^1^H NMR (300 MHz, DMSO-*d*_6_) δ 12.31 (s, 1H), 7.80–7.63 (m, 1H), 4.52–4.24 (m, 2H), 3.04–2.88 (m, 1H), 2.70 (d, *J* = 4.6 Hz, 3H), 1.18 (d, *J* = 7.0 Hz, 6H). ^13^C NMR (151 MHz, DMSO-*d*_6_) δ 164.8, 133.4, 132.6, 127.6, 25.1, 23.4, 21.3. LC-MS: *t*_R_ = 2.29 min, purity: 97%, *m*/*z* [M + H]^+^: 183.

### 5-(4-Fluorophenyl)-3-isopropyl-6-methyl-2,6-dihydro-7*H*-pyrazolo[4,3-*d*]pyrimidin-7-one (**7**, NPD-3639)

A mixture of **5** (150 mg, 0.823 mmol), 4-fluorobenzaldehyde (102 mg, 0.823 mmol), and I_2_ (418 mg, 1.65 mmol) in DMF (5 mL) was heated at 80 °C for 16 h. The reaction mixture was dissolved in a 10% Na_2_S_2_O_3_ aqueous solution (100 mL), extracted with EtOAc (3 × 100 mL), washed with brine, concentrated *in vacuo*, and purified by flash column chromatography on silica gel with a gradient elution of EtOAc in cyclohexane (10–60%) to get the title compound as a white solid (140 mg, 59%). ^1^H NMR (500 MHz, CDCl_3_) δ 7.57–7.53 (m, 2H), 7.24–7.19 (m, 2H), 3.53 (s, 3H), 3.45 (hept, *J* = 7.0 Hz, 1H), 1.44 (d, *J* = 7.0 Hz, 6H). ^13^C NMR (126 MHz, CDCl_3_) δ 163.6 (d, *J* = 251.3 Hz), 155.1, 153.0*, 130.7 (d, *J* = 8.6 Hz), 116.1 (d, *J* = 21.9 Hz), 34.4, 26.3, 21.9. LC-MS: *t*_R_ = 3.96 min, purity: >99%, *m*/*z* [M + H]^+^: 287; HR-MS: calcd for C_15_H_15_FN_4_O [M + H]^+^. 287.1303; found, 287.1303.

### Methyl 3-Isopropyl-1-methyl-4-nitro-1*H*-pyrazole-5-carboxylate (**8**) and Methyl 5-Isopropyl-1-methyl-4-nitro-1*H*-pyrazole-3-carboxylate (**12**)

To a mixture of K_2_CO_3_ (13.9 g, 100 mmol) and **3** (5.00 g, 25.1 mmol) in DMF (50 mL) was added MeI (3.45 mL, 55.2 mmol), and the reaction mixture was heated at 60 °C for 1 h. After that, this mixture was concentrated *in vacuo*, dissolved in water (50 mL), extracted with EtOAc (3 × 50 mL), and washed with brine. The combined organic layers were concentrated *in vacuo* and purified by flash column chromatography on silica gel eluting with EtOAc in cyclohexane (10–50%) to give the title compounds **8** (1.33 g, 23%) and **12** (1.46 g, 26%) as off-white solids. Compound **8**: ^1^H NMR (600 MHz, CDCl_3_) δ 3.98 (s, 3H), 3.96 (s, 3H), 3.43 (hept, *J* = 7.1 Hz, 1H), 1.30 (d, *J* = 6.9 Hz, 6H). ^13^C NMR (151 MHz, CDCl_3_) δ 159.3, 153.3, 132.1, 131.9*, 53.7, 39.2, 26.5, 21.5. LC-MS: *t*_R_ = 4.46 min, purity: >99%, *m*/*z* [M + H]^+^: 228. Compound **12**: ^1^H NMR (600 MHz, CDCl_3_) δ 3.94 (s, 3H), 3.94 (s, 3H), 3.48 (hept, *J* = 7.2 Hz, 1H), 1.40 (d, *J* = 7.2 Hz, 6H). ^13^C NMR (151 MHz, CDCl_3_) δ 160.8, 146.3, 137.4, 132.1*, 53.1, 39.1, 25.8, 19.4. LC-MS: *t*_R_ = 3.96 min, purity: >99%, *m*/*z* [M + H]^+^: 228. Regiochemistry was confirmed by 1D NOESY spectrum (Figures S14 and S18).

### 3-Isopropyl-1-methyl-4-nitro-1*H*-pyrazole-5-carboxamide (**10**)

Ester **8** (1.33 g, 5.84 mmol) was dissolved in 7 M NH_3_ in MeOH (4.17 mL, 29.2 mmol) and stirred at RT for 16 h. The reaction mixture was then concentrated *in vacuo* and used in the next step without further purification. The crude intermediate **9** (1.40 g) was added to a suspension of 10% palladium on carbon (0.200 g, 1.88 mmol) in EtOH (50 mL) and heated at 60 °C with a H_2_ gas insert for 16 h. After that, the reaction mixture was filtered through Celite, concentrated *in vacuo*, and purified by flash column chromatography on silica gel with a gradient elution of MeOH in DCM (0–10%) to give the title compound **10** as a pink solid (0.98 g, 92% over two steps). ^1^H NMR (300 MHz, DMSO-*d*_6_) δ 7.51 (br s, 2H), 4.09 (s, 2H), 3.86 (s, 3H), 2.97 (hept, *J* = 7.0 Hz, 1H), 1.16 (d, *J* = 6.9 Hz, 6H). ^13^C NMR (151 MHz, DMSO-*d*_6_) δ 162.0, 146.1, 128.0, 124.3, 39.0, 24.3, 21.8. LC-MS: *t*_R_ = 2.14 min, purity: 97%, *m*/*z* [M + H]^+^: 183.

### 5-(4-Fluorophenyl)-3-isopropyl-1-methyl-1,6-dihydro-7*H*-pyrazolo[4,3-*d*]pyrimidin-7-one (**11**, NPD-3205)

Amine **10** (0.15 g, 0.82 mmol) and 4-fluorobenzoic acid (0.12 g, 0.82 mmol), PyBrop (0.42 g, 0.91 mmol), and TEA (0.23 mL, 1.7 mmol) were combined in DCE (5.0 mL) and heated using microwave irradiation at 120 °C for 20 min. The solvent was evaporated, and the reaction mixture was purified by column chromatography with an eluent of DCM in MeOH (0–10%) to get the amide intermediate. The amide intermediate was combined with KO^*t*^Bu (185 mg, 1.65 mmol) in ^*i*^PrOH (10.0 mL) and heated using microwave irradiation at 130 °C for 30 min. The reaction mixture was concentrated *in vacuo* and purified by column chromatography with an eluent of cyclohexane in EtOAc (10–50%) with 2% AcOH to get the title compound as a white solid (0.17 g, 73% over two steps). ^1^H NMR (600 MHz, CDCl_3_) δ 10.65 (s, 1H), 8.13–8.08 (m, 2H), 7.24–7.18 (m, 2H), 4.28 (s, 3H), 3.43 (hept, *J* = 7.1 Hz, 1H), 1.47 (d, *J* = 7.1 Hz, 6H). ^13^C NMR (151 MHz, DMSO-*d*_6_) δ 163.6 (d, *J* = 248.6 Hz), 154.7, 149.8, 149.0, 137.0, 130.0 (d, *J* = 8.8 Hz), 129.5 (d, *J* = 2.8 Hz), 124.5, 115.5 (d, *J* = 21.8 Hz), 37.8, 26.2, 21.9. LC-MS: *t*_R_ = 4.49 min, purity: >99%, *m*/*z* [M + H]^+^: 287; HR-MS: calcd for C_15_H_15_FN_4_O [M + H]^+^. 287.1303; found, 287.1312.

### 4-Amino-5-isopropyl-1-methyl-1*H*-pyrazole-3-carboxamide (**14**)

Ester **12** (1.46 g, 6.88 mmol) was dissolved in 7 M NH_3_ in MeOH (4.58 mL, 32.1 mmol) and stirred at RT for 16 h. The reaction mixture was then concentrated *in vacuo* and used in the next step without further purification. The crude intermediate **13** (1.40 g) was added to the suspension of 10% palladium on carbon (0.250 g, 2.35 mmol) in EtOH (50 mL) and heated at 60 °C with a H_2_ gas insert for 16 h. After that, the reaction mixture was filtered through Celite, concentrated *in vacuo*, and purified by flash column chromatography on silica gel with a gradient elution of MeOH in DCM (0–10%) to give the title compound as a pink solid (1.20 g, 96% over two steps). ^1^H NMR (300 MHz, DMSO-*d*_6_) δ 7.07 (s, 1H), 6.94 (s, 1H), 4.42 (s, 2H), 3.71 (s, 3H), 3.06 (hept, *J* = 6.9 Hz, 1H), 1.24 (d, *J* = 7.1 Hz, 6H). ^13^C NMR (151 MHz, DMSO-*d*_6_) δ 166.0, 132.5, 130.4, 129.8, 37.6, 24.3, 20.0. LC-MS: *t*_R_ = 1.78 min, purity: >99%, *m*/*z* [M + H]^+^: 183. Spectral data agree with a previous report.^22^

### 5-(4-Fluorophenyl)-3-isopropyl-2-methyl-2,6-dihydro-7*H*-pyrazolo[4,3-*d*]pyrimidin-7-one (**15**, NPD-3541)

The product was prepared from **14** as described for **11** to get the title compound as a white solid (183 mg, 78% over two steps). ^1^H NMR (300 MHz, DMSO-*d*_6_) δ 12.01 (s, 1H), 8.20–8.08 (m, 2H), 7.41–7.29 (m, 2H), 4.04 (s, 3H), 3.41 (hept, *J* = 7.1 Hz, 1H), 1.48 (d, *J* = 7.0 Hz, 6H). ^13^C NMR (151 MHz, DMSO-*d*_6_) δ 164.0 (d, *J* = 248.1 Hz), 157.9, 148.4, 142.0, 135.4, 134.0, 130.24 (d, *J* = 8.8 Hz), 130.21 (d, *J* = 1.9 Hz), 116.0 (d, *J* = 21.7 Hz), 38.9, 26.1, 21.7. LC-MS: *t*_R_ = 4.12 min, purity: >99%, *m*/*z* [M + H]^+^: 287; HR-MS: calcd for C_15_H_15_FN_4_O [M + H]^+^. 287.1303; found, 287.1314.

### 5-(4-Fluorophenyl)-3-isopropyl-1,6-dihydro-7*H*-pyrazolo[4,3-*d*]pyrimidin-7-imine (**17**, NPD-3651)

A mixture of **2** (0.20 g, 0.74 mmol) and POCl_3_ (10.0 mL, 0.110 mmol) was stirred at 120 °C for 1 h. The reaction mixture was cooled to RT and concentrated *in vacuo*. The residue was coevaporated with toluene three times to yield a yellow oil. A solution of NH_3_ in THF (0.4 M, 16 mL) was added to the crude intermediate **16** (0.22 g), after which the reaction mixture was heated using microwave irradiation at 120 °C for 28 h. The reaction mixture was concentrated *in vacuo* and purified by flash column chromatography on silica gel with a gradient elution of EtOAc in cyclohexane (0–90%) to get the title compound as a white solid (61 mg, 33% over two steps). ^1^H NMR (600 MHz, DMSO-*d*_6_) δ 12.52 (s, 1H), 8.43–8.36 (m, 2H), 7.34 (br s, 2H), 7.30–7.24 (m, 2H), 3.33 (1H, confirmed by HSQC), 1.44 (d, *J* = 6.9 Hz, 6H). ^13^C NMR (151 MHz, DMSO-*d*_6_) δ 163.0 (d, *J* = 245.5 Hz), 155.3, 150.5*, 135.5 ^Δ^, 129.5 (d, *J* = 8.8 Hz), 114.9 (d, *J* = 21.6 Hz), 26.6^#^, 21.8. LC-MS: *t*_R_ = 3.40 min, purity: >99%, *m*/*z* [M + H]^+^: 272; HR-MS: calcd for C_14_H_14_FN_5_ [M + H]^+^. 272.1306; found, 272.1313.

### Ethyl 1-Ethyl-3-isopropyl-4-nitro-1*H*-pyrazole-5-carboxylate (**18a**)

To a mixture of **3** (5.00 g, 25.1 mmol) and K_2_CO_3_ (10.4 g, 75.3 mmol) in DMF (50.0 mL) was added ethyl bromide (3.90 mL, 52.7 mmol), stirred at 60 °C for 4 h. The reaction mixture was concentrated *in vacuo*, dissolved in water (100 mL), and extracted with EtOAc (3 × 100 mL); the combined organic layers were washed with brine, dried over MgSO_4_, concentrated *in vacuo*, and purified with flash column chromatography on silica gel with a gradient elution of EtOAc in cyclohexane (0–30%) to get the title compound as a yellow oil (4.70 g, 73%). ^1^H NMR (500 MHz, CDCl_3_) δ 4.46 (q, *J* = 7.1 Hz, 2H), 4.24 (q, *J* = 7.3 Hz, 2H), 3.46 (hept, *J* = 6.9 Hz, 1H), 1.46 (t, *J* = 7.3 Hz, 3H), 1.39 (t, *J* = 7.1 Hz, 3H), 1.30 (d, *J* = 6.9 Hz, 6H). ^13^C NMR (126 MHz, CDCl_3_) δ 159.1, 153.4, 132.1, 131.5, 63.3, 47.4, 26.7, 21.4, 15.6, 13.9. LC-MS: *t*_R_ = 5.08 min, purity: >99%, *m*/*z* [M + H]^+^: 256. Regiochemistry was confirmed by 1D NOESY spectrum (Figure S37).

### Propyl 3-Isopropyl-4-nitro-1-propyl-1*H*-pyrazole-5-carboxylate (**18b**)

The compound was prepared from **3** and 1-bromopropane as described for **18a** to get the title compound as a yellow oil (4.82 g, 69%). ^1^H NMR (500 MHz, CDCl_3_) δ 4.34 (t, *J* = 6.7 Hz, 2H), 4.17–4.12 (m, 2H), 3.46 (hept, *J* = 6.9 Hz, 1H), 1.91–1.83 (m, 2H), 1.81–1.74 (m, 2H), 1.31 (s, 3H), 1.29 (s, 3H), 1.00 (t, *J* = 7.5 Hz, 3H), 0.92 (t, *J* = 7.4 Hz, 3H). ^13^C NMR (126 MHz, CDCl_3_) δ 159.2, 153.4, 132.5, 131.4, 68.8, 53.6, 26.6, 23.6, 21.8, 21.4, 11.0, 10.5. LC-MS: *t*_R_ = 5.62 min, purity: >99%, *m*/*z* [M + H]^+^: 284. Regiochemistry was confirmed by 1D NOESY spectrum (Figure S41).

### Isopropyl 1,3-Diisopropyl-4-nitro-1*H*-pyrazole-5-carboxylate (**18c**)

The compound was prepared from **3** and 2-bromopropane as described for **18a** to get the title compound as a white solid (2.75 g, 39%). ^1^H NMR (300 MHz, CDCl_3_) δ 5.33 (hept, *J* = 6.0 Hz, 1H), 4.58 (hept, *J* = 6.5 Hz, 1H), 3.49 (hept, *J* = 7.1 Hz, 1H), 1.50 (d, *J* = 6.6 Hz, 6H), 1.39 (d, *J* = 6.3 Hz, 6H), 1.30 (d, *J* = 6.9 Hz, 6H). ^13^C NMR (126 MHz, CDCl_3_) δ 159.1, 153.2, 132.9, 130.5*, 71.6, 54.0, 26.9, 22.3, 21.5, 21.4. LC-MS: *t*_R_ = 5.70 min, purity: >99%, *m*/*z* [M + H]^+^: 284. Regiochemistry was confirmed by 1D NOESY spectrum (Figure S45).

### 2-Methoxyethyl 3-Isopropyl-1-(2-methoxyethyl)-4-nitro-1*H*-pyrazole-5-carboxylate (**18d**)

The compound was prepared from **3** with 1-bromo-2-methoxyethane as described for **18a** but reacted for 16 h to get the title compound as a yellow oil (4.50 g, 56%). ^1^H NMR (500 MHz, CDCl_3_) δ 4.54–4.50 (m, 2H), 4.42 (t, *J* = 5.3 Hz, 2H), 3.71–3.65 (m, 4H), 3.49 (hept, *J* = 7.0 Hz, 1H), 3.39 (s, 3H), 3.29 (s, 3H), 1.30 (d, *J* = 6.9 Hz, 6H). ^13^C NMR (126 MHz, CDCl_3_) δ 159.1, 153.8, 133.5, 131.8, 70.5, 69.8, 65.7, 59.12, 59.07, 51.6, 26.7, 21.4. LC-MS: *t*_R_ = 4.61 min, purity: 94%, *m*/*z* [M + H]^+^: 316. Regiochemistry was confirmed by 1D NOESY spectrum (Figure S49).

### 4-Methoxyphenethyl 3-Isopropyl-1-(4-methoxyphenethyl)-4-nitro-1*H*-pyrazole-5-carboxylate (**18e**)

The compound was prepared from **3** with 4-methoxyphenethyl bromide as described for **18a** but reacted for 16 h to get the title compound as a white solid (8.02 g, 68%). ^1^H NMR (500 MHz, CDCl_3_) δ 7.16–7.11 (m, 2H), 6.95–6.90 (m, 2H), 6.86–6.82 (m, 2H), 6.81–6.78 (m, 2H), 4.39 (t, *J* = 7.4 Hz, 2H), 4.33 (t, *J* = 7.1 Hz, 2H), 3.77 (s, 3H), 3.76 (s, 3H), 3.45 (hept, *J* = 6.9 Hz, 1H), 3.00 (t, *J* = 7.3 Hz, 2H), 2.91 (t, *J* = 7.4 Hz, 2H), 1.30 (d, *J* = 6.9 Hz, 6H). ^13^C NMR (126 MHz, CDCl_3_) δ 158.7, 158.7, 158.6, 153.5, 132.2, 130.0, 129.9, 128.9, 128.9, 114.2, 114.2, 67.5, 55.4, 55.4, 53.4, 35.7, 33.7, 26.6, 21.5. The ^13^C signal of the carbonyl substituted pyrazole carbon is missing. LC-MS: *t*_R_ = 5.75 min, purity: >99%, *m*/*z* [M + H]^+^: 468. Regiochemistry was confirmed by the 1D NOESY spectrum (Figure S53).

### 1,3-Diisopropyl-4-nitro-1*H*-pyrazole-5-carboxamide (**19c**)

Ester **18c** (2.75 g, 9.71 mmol) was dissolved in 7 M NH_3_ in MeOH (6.9 mL, 48.6 mmol) and stirred at RT for 16 h. The reaction mixture was then concentrated *in vacuo* and purified with flash column chromatography on silica gel with a gradient elution of EtOAc in cyclohexane (30–70%) to get the title compound as a white solid (1.72 g, 74%). ^1^H NMR (500 MHz, CDCl_3_) δ 6.67 (s, 1H), 6.06 (s, 1H), 4.85 (hept, *J* = 6.6 Hz, 1H), 3.52 (hept, *J* = 6.9 Hz, 1H), 1.50 (d, *J* = 6.6 Hz, 6H), 1.30 (d, *J* = 6.9 Hz, 6H). ^13^C NMR (126 MHz, CDCl_3_) δ 160.4, 154.0, 134.2, 129.5*, 53.9, 27.2, 22.5, 21.3. LC-MS: *t*_R_ = 4.05 min, purity: 96%, *m*/*z* [M + H]^+^: 241.

### 3-Isopropyl-1-(4-methoxyphenethyl)-4-nitro-1*H*-pyrazole-5-carboxamide (**19e**)

The compound was prepared from **18e** as described for **19c** but heated in a microwave vial at 90 °C for 2 days to get the title compound as a white solid (3.05 g, 53%). ^1^H NMR (500 MHz, DMSO-*d*_6_) δ 8.43 (s, 1H), 8.25 (s, 1H), 7.10–7.06 (m, 2H), 6.86–6.81 (m, 2H), 4.27–4.21 (m, 2H), 3.71 (s, 3H), 3.44 (hept, *J* = 6.9 Hz, 1H), 3.01 (d, *J* = 8.3 Hz, 2H), 1.21 (d, *J* = 6.9 Hz, 6H). ^13^C NMR (126 MHz, DMSO-*d*_6_) δ 159.7, 158.1, 152.3, 138.2, 129.7, 129.2, 128.4, 113.9, 55.0, 52.3, 34.6, 26.3, 21.2. LC-MS: *t*_R_ = 4.37 min, purity: >99%, *m*/*z* [M + H]^+^: 333.

### 1-(3-Amino-3-oxopropyl)-3-isopropyl-4-nitro-1*H*-pyrazole-5-carboxamide (**19f**)

The compound was prepared from crude **24** as described for **19c** but heated in a microwave vial at 60 °C for 2 days to get the title compound as a white solid (423 mg, 7% over two steps). ^1^H NMR (500 MHz, DMSO-*d*_6_) δ 7.89 (s, 1H), 7.66 (s, 1H), 7.47 (s, 1H), 6.99 (s, 1H), 4.34 (t, *J* = 6.9 Hz, 2H), 3.54 (hept, *J* = 6.9 Hz, 1H), 2.68 (t, *J* = 6.8 Hz, 2H), 1.30 (d, *J* = 7.2 Hz, 6H). ^13^C NMR (126 MHz, DMSO-*d*_6_) δ 171.1, 162.0, 146.2, 142.3, 129.9, 46.3, 34.7, 25.0, 19.1. LC-MS: *t*_R_ = 2.84 min, purity: >99%, *m*/*z* [M + H]^+^: 270.

### 4-Amino-1-ethyl-3-isopropyl-1*H*-pyrazole-5-carboxamide (**20a**)

Ester **18a** (4.70 g, 18.4 mmol) was dissolved in 7 M NH_3_ in MeOH (7.90 mL, 55.2 mmol) and stirred at RT for 16 h. The reaction mixture was then concentrated *in vacuo* and used in the next step without further purification. The crude intermediate **19a** (4.90 g) was added to the suspension of 10% palladium on carbon (1.00 g) in EtOH (50 mL) and heated at 75 °C with a H_2_ gas insert for 16 h. Then, the reaction mixture was filtered through Celite and concentrated *in vacuo* to get the title compound as a pink solid (1.90 g), which was used in the next step without purification.

### 4-Amino-3-isopropyl-1-propyl-1*H*-pyrazole-5-carboxamide (**20b**)

The compound was prepared from **18b** as described for **20a** to get the title compound as a pink solid (2.85 g, 68% over two steps). ^1^H NMR (500 MHz, CDCl_3_) δ 4.48–4.43 (m, 2H), 2.97 (hept, *J* = 6.9 Hz, 1H), 2.83 (s, 2H), 1.84–1.74 (m, 2H), 1.29 (d, *J* = 7.0 Hz, 6H), 0.88 (t, *J* = 7.5 Hz, 3H). ^13^C NMR (126 MHz, CDCl_3_) δ 162.0, 149.7, 126.7, 124.4, 53.6, 25.7, 24.3, 22.0, 11.1. LC-MS: *t*_R_ = 2.90 min, purity: >99%, *m*/*z* [M + H]^+^: 211.

### 4-Amino-1,3-diisopropyl-1*H*-pyrazole-5-carboxamide (**20c**)

Amide **19c** (1.72 g, 7.16 mmol) was added to the suspension of 10% palladium on carbon (300 mg) in EtOH (20 mL) and heated at 60 °C with a H_2_ gas insert for 16 h. Then, the reaction mixture was filtered through Celite, concentrated *in vacuo*, and used in the next step without further purification.

### 4-Amino-3-isopropyl-1-(2-methoxyethyl)-1*H*-pyrazole-5-carboxamide (**20d**)

The compound was prepared from **18d** as described for **20a** to get the title compound as a yellow oil (2.80 g, 71% over two steps). ^1^H NMR (500 MHz, CDCl_3_) δ 4.47 (t, *J* = 4.7 Hz, 2H), 3.81 (t, *J* = 4.8 Hz, 2H), 3.36 (s, 3H), 2.92 (hept, *J* = 6.9 Hz, 1H), 1.29 (d, *J* = 6.9 Hz, 6H). ^13^C NMR (126 MHz, CDCl_3_) δ 163.2, 147.3, 130.1, 124.1, 72.5, 59.2, 51.2, 26.0, 21.5. LC-MS: *t*_R_ = 2.43 min, purity: 98%, *m*/*z* [M + H]^+^: 227. Spectral data agree with a previous report.^23^

### 4-Amino-3-isopropyl-1-(4-methoxyphenethyl)-1*H*-pyrazole-5-carboxamide (**20e**)

The compound was prepared from **19e** as described for **20c** to get the title compound as a white solid (1.85 g, 67%). ^1^H NMR (500 MHz, DMSO-*d*_6_) δ 7.58 (br s, 2H), 7.10–7.04 (m, 2H), 6.85–6.78 (m, 2H), 4.48–4.39 (m, 2H), 4.05 (s, 2H), 3.70 (s, 3H), 2.97 (hept, *J* = 6.9 Hz, 1H), 2.86–2.79 (m, 2H), 1.14 (d, *J* = 6.9 Hz, 6H). ^13^C NMR (126 MHz, DMSO-*d*_6_) δ 161.9, 157.8, 146.9, 130.5, 129.7, 127.6, 124.2, 113.7, 55.0, 52.4, 35.7, 24.4, 21.9. LC-MS: *t*_R_ = 3.59 min, purity: >99%, *m*/*z* [M + H]^+^: 303.

### 4-Amino-1-(3-amino-3-oxopropyl)-3-isopropyl-1*H*-pyrazole-5-carboxamide (**20f**)

Amide **19f** (0.25 g, 0.94 mmol) was added to the suspension of 10% palladium on carbon (300 mg) in EtOH (20 mL) and heated at 60 °C with a H_2_ gas insert for 16 h. Then, the reaction mixture was filtered through Celite, concentrated *in vacuo*, and used in the next step without further purification.

### 1-Ethyl-5-(4-fluorophenyl)-3-isopropyl-1,6-dihydro-7*H*-pyrazolo[4,3-*d*]pyrimidin-7-one (**21a**, NPD-3649)

The compound was prepared from crude **20a** as described for **11** to get the title compound as a white solid (170 mg, 35% over four steps). ^1^H NMR (500 MHz, CDCl_3_) δ 11.64 (s, 1H), 8.27–8.22 (m, 2H), 7.26–7.20 (m, 2H), 4.69 (q, *J* = 7.2 Hz, 2H), 3.47 (hept, *J* = 7.0 Hz, 1H), 1.57 (t, *J* = 7.2 Hz, 3H), 1.51 (d, *J* = 7.0 Hz, 6H). ^13^C NMR (151 MHz, CDCl_3_) δ 164.7 (d, *J* = 252.1 Hz), 155.8, 151.6, 148.3, 138.7, 129.6 (d, *J* = 8.7 Hz), 129.2 (d, *J* = 3.0 Hz), 123.7, 116.1 (d, *J* = 22.0 Hz), 46.8, 27.0, 22.2, 16.4. LC-MS: *t*_R_ = 4.81 min, purity: >99%, *m*/*z* [M + H]^+^: 301; HR-MS: calcd for C_16_H_17_FN_4_O [M + H]^+^. 301.1459; found, 301.1466.

### 5-(4-Fluorophenyl)-3-isopropyl-1-propyl-1,6-dihydro-7*H*-pyrazolo[4,3-*d*]pyrimidin-7-one (**21b**, NPD-3733)

The compound was prepared from **20b** as described for **11** to get the title compound as a white solid (178 mg, 79% over two steps). ^1^H NMR (500 MHz, CDCl_3_) δ 11.52 (s, 1H), 8.23–8.15 (m, 2H), 7.24–7.16 (m, 2H), 4.60–4.54 (m, 2H), 3.43 (hept, *J* = 7.0 Hz, 1H), 2.01–1.90 (m, 2H), 1.47 (d, *J* = 6.9 Hz, 6H), 0.93 (t, *J* = 7.5 Hz, 3H). ^13^C NMR (126 MHz, CDCl_3_) δ 164.6 (d, *J* = 252.0 Hz), 155.8, 151.7, 148.2, 138.6, 129.5 (d, *J* = 8.7 Hz), 129.4 (d, *J* = 3.2 Hz), 124.1, 116.1 (d, *J* = 21.9 Hz), 53.0, 27.0, 24.5, 22.2, 11.1. LC-MS: *t*_R_ = 5.17 min, purity: >99%, *m*/*z* [M + H]^+^: 315; HR-MS: calcd for C_17_H_19_FN_4_O [M + H]^+^. 315.1616; found, 315.1631.

### 5-(4-Fluorophenyl)-1,3-diisopropyl-1,6-dihydro-7*H*-pyrazolo[4,3-*d*]pyrimidin-7-one (**21c**, NPD-3735)

The compound was prepared from crude **20c** as described for **11** to get the title compound as a white solid (174 mg, 55% over three steps). ^**1**^H NMR (500 MHz, CDCl_3_) δ 11.52 (s, 1H), 8.20 (ddd, *J* = 10.1, 5.1, 2.5 Hz, 2H), 7.23–7.15 (m, 2H), 5.38 (hept, *J* = 6.7 Hz, 1H), 3.42 (hept, *J* = 7.0 Hz, 1H), 1.60 (d, *J* = 6.7 Hz, 6H), 1.48 (d, *J* = 7.0 Hz, 6H). ^13^C NMR (151 MHz, CDCl_3_) δ 164.6 (d, *J* = 252.1 Hz), 155.7, 151.2, 147.9, 138.7, 129.5 (d, *J* = 8.7 Hz), 129.4^Δ^, 123.3, 116.1 (d, *J* = 21.6 Hz), 53.4, 27.3, 22.6, 22.2. LC-MS: *t*_R_ = 5.34 min, purity: >99%, *m*/*z* [M + H]^+^: 315.

### 5-(4-Fluorophenyl)-3-isopropyl-1-(2-methoxyethyl)-1,6-dihydro-7*H*-pyrazolo[4,3-*d*]pyrimidin-7-one (**21d**, NPD-3652)

The compound was prepared from **20d** as described for **11** to get the title compound as a white solid (55 mg, 25% over two steps). ^1^H NMR (500 MHz, CDCl_3_) δ 11.56 (s, 1H), 8.22–8.17 (m, 2H), 7.24–7.19 (m, 2H), 4.80 (t, *J* = 5.9 Hz, 2H), 3.87 (t, *J* = 5.9 Hz, 2H), 3.44 (hept, *J* = 7.0 Hz, 1H), 3.33 (s, 3H), 1.48 (d, *J* = 7.0 Hz, 6H). ^13^C NMR (126 MHz, CDCl_3_) δ 164.6 (d, *J* = 251.8 Hz), 155.8, 152.2, 148.3, 138.9, 129.5 (d, *J* = 8.7 Hz), 129.3 (d, *J* = 3.1 Hz), 124.6, 116.1 (d, *J* = 22.0 Hz), 71.6, 59.0, 50.7, 27.0, 22.1. LC-MS: *t*_R_ = 4.58 min, purity: 99%, *m*/*z* [M + H]^+^: 331; HR-MS: calcd for C_17_H_19_FN_4_O_2_ [M + H]^+^. 331.1565; found, 331.1572.

### 5-(4-Fluorophenyl)-3-isopropyl-1-(4-methoxyphenethyl)-1,6-dihydro-7*H*-pyrazolo[4,3-*d*]pyrimidin-7-one (**21e**, NPD-3653)

The compound was prepared from **20e** as described for **11** to get the title compound as a white solid (90 mg, 45% over two steps). ^1^H NMR (500 MHz, DMSO-*d*_6_) δ 11.44 (s, 1H), 8.20–8.14 (m, 2H), 7.13–7.07 (m, 2H), 7.06–7.02 (m, 2H), 6.78–6.74 (m, 2H), 4.83–4.77 (m, 2H), 3.75 (s, 3H), 3.44 (hept, *J* = 6.9 Hz, 1H), 3.19–3.13 (m, 2H), 1.47 (d, *J* = 7.0 Hz, 6H). ^13^C NMR (126 MHz, DMSO-*d*_6_) δ 163.6 (d, *J* = 248.4 Hz), 157.8, 154.5, 149.9, 149.1, 137.1, 130.0 (d, *J* = 8.9 Hz), 129.9, 129.7, 129.6 (d, *J* = 2.6 Hz), 124.2, 115.6 (d, *J* = 22.0 Hz), 113.7, 55.0, 51.8, 35.6, 26.2, 22.0. LC-MS: *t*_R_ = 5.35 min, purity: 99%, *m*/*z* [M + H]^+^: 407; HR-MS: calcd for C_23_H_23_FN_4_O_2_ [M + H]^+^. 407.1878; found, 407.1882.

### 3-(5-(4-Fluorophenyl)-3-isopropyl-7-oxo-6,7-dihydro-1*H*-pyrazolo[4,3-*d*]pyrimidin-1-yl)propanamide (**21f**, NPD-3732)

The compound was prepared from crude **20f** as described for **11** to get the title compound as a white solid (37 mg, 17% over three steps). ^1^H NMR (600 MHz, DMSO-*d*_6_) δ 12.46 (s, 1H), 8.16–8.10 (m, 2H), 7.39 (s, 1H), 7.38–7.33 (m, 2H), 6.88 (s, 1H), 4.72–4.65 (m, 2H), 3.28 (hept, *J* = 7.0 Hz, 1H), 2.70–2.65 (m, 2H), 1.38 (d, *J* = 7.0 Hz, 6H). ^13^C NMR (151 MHz, DMSO-*d*_6_) δ 171.3, 163.6 (d, *J* = 248.2 Hz), 154.5, 150.0, 149.0, 137.2, 130.0 (d, *J* = 8.8 Hz), 129.5 (d, *J* = 2.7 Hz), 124.2, 115.5 (d, *J* = 22.1 Hz), 46.8, 35.7, 26.3, 21.8. LC-MS: *t*_R_ = 3.84 min, purity: 96%, *m*/*z* [M + H]^+^: 344; HR-MS: calcd for C_17_H_18_FN_5_O_2_ [M + H]^+^. 344.1517; found, 344.1500.

### 5-(4-Fluorophenyl)-1-(2-hydroxyethyl)-3-isopropyl-1,6-dihydro-7*H*-pyrazolo[4,3-*d*]pyrimidin-7-one (**22**, NPD-3731)

To a solution of **21d** (0.19 g, 0.58 mmol) in DCM (60 mL) was added 1 M BBr_3_ in DCM (2.4 mL, 2.4 mmol) dropwise at −78 °C and slowly increased to RT for 16 h. Saturated aq. NaHCO_3_ solution was added, and the reaction mixture was extracted with EtOAc (3 × 100 mL). The combined organic layers were washed with brine, dried over MgSO_4_, and concentrated *in vacuo*. The crude product was purified with flash column chromatography on silica gel with a gradient elution of MeOH in DCM (0–26%) to get the title compound as a white solid (89 mg, 48%). ^1^H NMR (600 MHz, DMSO-*d*_6_) δ 12.44 (s, 1H), 8.16–8.10 (m, 2H), 7.39–7.33 (m, 2H), 4.86 (t, *J* = 5.6 Hz, 1H), 4.55 (t, *J* = 6.0 Hz, 2H), 3.82–3.76 (m, 2H), 3.28 (hept, *J* = 7.1 Hz, 1H), 1.39 (d, *J* = 7.0 Hz, 6H). ^13^C NMR (151 MHz, DMSO-*d*_6_) δ 163.6 (d, *J* = 248.2 Hz), 154.5, 150.0, 148.9, 137.2, 130.0 (d, *J* = 8.8 Hz), 129.6 (d, *J* = 2.8 Hz), 124.7, 115.6 (d, *J* = 21.6 Hz), 60.3, 52.9, 26.3, 21.8. LC-MS: *t*_R_ = 4.05 min, purity: 98%, *m*/*z* [M + H]^+^: 317; HR-MS: calcd for C_16_H_17_FN_4_O_2_ [M + H]^+^. 317.1408; found, 317.1396.

### Methyl 3-Isopropyl-4-nitro-1*H*-pyrazole-5-carboxylate (**23**)

To a mixture of **3** (5.00 g, 25.1 mmol) in DCM (70 mL) containing DMF (0.10 mL, 1.3 mmol) was added oxalyl chloride (6.50 mL, 75.3 mmol) dropwise at 0 °C. The reaction mixture was stirred for 1 h, allowed to warm to RT, and stirred for another 2 h. Subsequently, the reaction mixture was added to a flask containing MeOH dropwise at 0 °C and stirred for 30 min. The reaction mixture was concentrated *in vacuo* and purified with flash column chromatography on silica gel with a gradient elution of EtOAc in cyclohexane (18–58%) to get the title compound as a white solid (4.73 g, 88% over two steps). ^1^H NMR (500 MHz, CDCl_3_) δ 3.98 (s, 3H), 3.63 (hept, *J* = 7.0 Hz, 1H), 1.38 (d, *J* = 7.0 Hz, 6H). ^13^C NMR (126 MHz, CDCl_3_) δ 161.0, 53.4, 25.8, 20.9. LC-MS: *t*_R_ = 3.58 min, purity: >99%, *m*/*z* [M + H]^+^: 214. Spectral data agree with a previous report.^24,40^

### Methyl 3-Isopropyl-1-(3-methoxy-3-oxopropyl)-4-nitro-1*H*-pyrazole-5-carboxylate (**24**)

To a mixture of **23** (4.73 g, 22.2 mmol) and K_2_CO_3_ (9.20 g, 66.5 mmol) in DMF (70 mL) was added methyl 3-bromopropionate (8.47 g, 46.8 mmol) and stirred at 60 °C for 16 h. Water (200 mL) was added, and the reaction mixture was extracted with EtOAc (3 × 200 mL). The combined organic layers were washed with brine, dried over Na_2_SO_4_, and concentrated *in vacuo*. This intermediate was used as a crude product (3.17 g) in the next step without further purification.

### 3-Cyclopentyl-1*H*-pyrazole-5-carboxylic acid (**27a**)

NaOEt (3.89 g, 54.9 mmol) was dissolved in EtOH (50 mL) at RT, and a solution of diethyl oxalate (7.56 mL, 55.4 mmol) in 1-cyclopentylethanone (5.67 mL, 46.1 mmol) was added dropwise at RT for 30 min. The reaction mixture was diluted with EtOH (50 mL) and heated to 60 °C for 2 h, after which AcOH (8.9 mL, 55 mmol) and 64–65% N_2_H_4_ monohydrate (2.20 mL, 46.1 mmol) were added, and the mixture was stirred under reflux for 2 h. The reaction mixture was concentrated under reduced pressure and used in the next step without further purification. The crude intermediate **26a** (6.1 g) was added to an aqueous 1 M NaOH solution (97 mL, 97 mmol) in 1,4-dioxane (112 mL); the reaction mixture was heated to 50 °C and stirred for 20 h. Then, the reaction was cooled to RT, and 1,4-dioxane was removed under reduced pressure. The residue was washed with diethyl ether (100 mL). The water layer was acidified to pH 1 with concentrated HCl (37% w/w). The white solid was filtered and dried *in vacuo* to yield the title product as a white solid (5.21 g, 63% for three steps). ^1^H NMR (600 MHz, DMSO-*d*_6_) δ 12.90 (br s, 1H), 6.46 (s, 1H), 3.04 (p, *J* = 8.1 Hz, 1H), 2.02–1.94 (m, 2H), 1.73–1.66 (m, 2H), 1.64–1.53 (m, 4H). ^13^C NMR (151 MHz, DMSO-*d*_6_) δ 104.6, 36.6^#^, 32.7, 24.6. LC-MS: *t*_R_ = 3.19 min, purity: >99%, *m*/*z* [M-H]^−^: 179.

### 3-Cyclohexyl-1*H*-pyrazole-5-carboxylic acid (**27b**)

A solution of diethyl oxalate (7.57 mL, 55.4 mmol) in 1-cyclohexylethanone (6.37 mL, 46.1 mmol) was added dropwise to NaOEt (3.89 g, 54.9 mmol) in EtOH (50 mL) at RT for 30 min. The reaction mixture was heated at 60 °C for 2 h, after which AcOH (8.9 mL, 55 mmol) and 64–65% N_2_H_4_ (2.20 mL, 46.1 mmol) were added. The reaction mixture was stirred under reflux for 2 h, concentrated under reduced pressure, and used in the next step without further purification. NaOH (6.45 g, 161 mmol) was added to a suspension of the crude intermediate **26b** (8.31 g) in a mixture of 1,4-dioxane (150 mL) and H_2_O (150 mL), and the reaction mixture was stirred at RT for 23 h. Upon completion, the reaction mixture was concentrated under reduced pressure, diluted with H_2_O (50 mL), and extracted with EtOAc (3 × 50 mL). The aqueous layer was adjusted to pH 1 concentrated aq. HCl. The precipitated off-white solid was filtered as the title compound (7.72 g, 37% over three steps). ^1^H NMR (300 MHz, DMSO-*d*_6_) δ 12.83 (br s, 1H), 6.45 (s, 1H), 2.69–2.55 (m, 1H), 1.90 (m, 2H), 1.79–1.60 (m, 3H), 1.45–1.11 (m, 5H). LC-MS: *t*_R_ = 3.47 min, purity: 98%, *m*/*z* [M + H]^+^: 195.

### 3-Cyclopentyl-4-nitro-1*H*-pyrazole-5-carboxylic acid (**28a**)

Acid **27a** (5.21 g, 28.9 mmol) was added portion-wise to concentrated H_2_SO_4_ (8.91 mL, 159 mmol) at RT with stirring. The reaction mixture was then heated to 60 °C and 65% HNO_3_ (6.95 mL, 101 mmol) was added dropwise, keeping the temperature at 60 °C. The reaction was stirred at 60 °C for 3 h, cooled to RT, and poured onto 200 g of ice. After 15 min, the white precipitate was isolated by filtration, washed with water, and dried under reduced pressure to give the title product as a white solid (4.01 g, 61%). ^1^H NMR (600 MHz, DMSO-*d*_6_ + 1 drop of D_2_O) δ 3.47 (p, *J* = 8.6 Hz, 1H), 2.08–1.99 (m, 2H), 1.79–1.70 (m, 2H), 1.69–1.57 (m, 4H). ^13^C NMR (151 MHz, DMSO-*d*_6_) δ 36.0, 32.0, 25.5. LC-MS: *t*_R_ = 3.26 min, purity: >99%, *m*/*z* [M-H]^−^: 224. Spectral data agree with a previous report.^41^

### 3-Cyclohexyl-4-nitro-1*H*-pyrazole-5-carboxylic acid (**28b**)

Acid **27b** (5.83 g, 30.0 mmol) was added in portions to 98% H_2_SO_4_ (30 mL, 0.54 mol) at RT. The suspension was heated to 60 °C, and 65% HNO_3_ (7.7 mL, 0.12 mol) was added dropwise. The reaction mixture was stirred at 60 °C for 3 h, then cooled to RT, and poured onto 230 g of ice. The white precipitate was filtered, washed with water, and dried under reduced pressure to get the title compound as a white solid (1.98 g), which was then used in the next step without further purification.

### 3-(*tert*-Butyl)-4-nitro-1*H*-pyrazole-5-carboxylic acid (**28c**)

Ester **26c** (25.0 g, 127 mmol) was dissolved in a mixture of 1,4-dioxane (100 mL) and water (100 mL), after which NaOH (15.3 g, 382 mmol) was added. The reaction mixture was concentrated under reduced pressure after heating at 60 °C for 4 h, washed with EtOAc (3 × 100 mL), the pH adjusted to 1 with concentrated HCl solution, and the off-white solid was filtered as intermediate **27c** (16.5 g, 77%), which was used in the next step without further purification. Acid **27c** (3.95 g, 23.5 mmol) was added portion-wise to concentrated H_2_SO_4_ (19.1 mL, 352 mmol) at RT with stirring. The reaction mixture was then heated to 60 °C and 65% HNO_3_ (4.50 mL, 70.4 mmol) was added dropwise, keeping the temperature at 60 °C. The reaction was stirred at 60 °C for 3 h, cooled to RT, and poured onto 200 g of ice. After 15 min, the white precipitate was isolated by filtration, washed with water, and dried under reduced pressure to give the title product as a white solid (4.50 g, 90%). ^1^H NMR (300 MHz, DMSO-*d*_6_) δ 13.82 (s, 1H), 1.34 (s, 9H). ^13^C NMR (151 MHz, DMSO-*d*_6_) δ 147.3*, 32.4*, 28.2. LC-MS: *t*_R_ = 3.26 min, purity: 96%, *m*/*z* [M + H]^+^: 214.

### 3-Ethyl-4-nitro-1*H*-pyrazole-5-carboxylic acid (**28d**)

The compound was prepared from **27d** as described for **28a** to get the title product as a white solid (3.23 g, 60%). ^1^H NMR (300 MHz, DMSO-*d*_6_) δ 13.98 (br s, 1H), 2.91 (q, *J* = 7.5 Hz, 2H), 1.23 (t, *J* = 7.5 Hz, 3H). ^13^C NMR (151 MHz, DMSO-*d*_6_) δ 145.7*, 129.2*, 18.3, 12.1. LC-MS: *t*_R_ = 2.23 min, purity: >99%, *m*/*z* [M-H]^−^: 184. Spectral data agree with a previous report.^27^

### 3-Ethyl-4-nitro-1*H*-pyrazole-5-carboxamide (**29d**)

Oxalyl chloride (4.58 mL, 52.3 mmol) was added dropwise to a suspension of **28d** (3.23 g, 17.5 mmol) in DCM (240 mL) containing DMF (0.082 mL, 1.1 mmol) at 0 °C. The reaction was stirred at 0 °C for 1 h, allowed to warm to RT, and stirred for a further 2 h. The reaction mixture was concentrated *in vacuo* and coevaporated with toluene three times. The residue was dissolved in DCM (100 mL) and added dropwise to 7 M NH_3_ in MeOH (7.48 mL, 52.3 mmol) at 0 °C. After stirring for 3 h, the reaction mixture was concentrated *in vacuo* and purified by flash column chromatography on silica gel with a gradient elution of EtOAc in cyclohexane (50–90%) to get the title product (3.00 g, 93%) as an off-white solid. ^1^H NMR (300 MHz, DMSO-*d*_6_) δ 13.84 (br s, 1H), 7.98 (s, 1H), 7.72 (s, 1H), 2.92 (q, *J* = 7.5 Hz, 2H), 1.23 (t, *J* = 7.5 Hz, 3H). ^13^C NMR (151 MHz, DMSO-*d*_6_) δ 146.0*, 128.7, 18.7, 12.2. LC-MS: *t*_R_ = 2.24 min, purity: >99%, *m*/*z* [M-H]^−^: 185. Spectral data agree with a previous report.^27^

### 3-Methyl-4-nitro-1*H*-pyrazole-5-carboxamide (**29e**)

The compound was prepared from **28e** (3.00 g, 23.8 mmol) as described for **29d** to get the title compound as a white solid (1.5 g, 54%). ^1^H NMR (500 MHz, DMSO-*d*_6_) δ 13.78 (s, 1H), 8.00 (s, 1H), 7.71 (s, 1H), 2.50 (s, 3H, confirmed by HSQC). ^13^C NMR (126 MHz, DMSO-*d*_6_) δ 162.5, 141.1*, 129.4, 11.2^#^. LC-MS: *t*_R_ = 1.69 min, purity: 96%, *m*/*z* [M + H]^+^: 171.

### 4-Amino-3-cyclopentyl-1*H*-pyrazole-5-carboxamide (**30a**)

Oxalyl chloride (1.09 mL, 12.5 mmol) was added dropwise to a suspension of **28a** (0.94 g, 4.2 mmol) in DCM (20 mL) containing DMF (0.014 mL, 0.18 mmol) under nitrogen at 0 °C. The reaction was stirred at 0 °C for 1 h, allowed to warm to RT, and stirred for a further 2 h. The reaction mixture was concentrated *in vacuo*, coevaporated with toluene three times, and used in the next step without further purification. The crude intermediate **29a** (1.5 g) was combined with 10% palladium on carbon (0.85 g, 8.0 mmol) in EtOH (90 mL) and stirred under a H_2_ gas insert at 60 °C for 6 h. The reaction mixture was filtered through Celite and the solid was washed with MeOH (50 mL). The filtrate was concentrated under reduced pressure, and the residue was used in the next step without further purification.

### 4-Amino-3-cyclohexyl-1*H*-pyrazole-5-carboxamide (**30b**)

Oxalyl chloride (15.0 mL, 11.1 mmol) was added dropwise to a solution of **28b** (2.42 g) in DCM (80 mL) containing two drops of DMF at 0 °C, which was then warmed to RT and stirred for 2 h. The reaction mixture was evaporated under reduced pressure, and coevaporated three times with toluene. The residue was then dissolved in toluene, added dropwise to a solution of NH_3_ in MeOH (7 M, 7.2 mL, 50 mmol) at 0 °C, and stirred at RT for 18 h. The resulting suspension was concentrated under reduced pressure and used for the next step without further purification. The crude intermediate **29b** (2.5 g) was combined with 10% palladium on carbon (0.24 g) in EtOH (50 mL) and stirred under H_2_ gas insert at 60 °C for 16 h. The reaction mixture was filtered through Celite and the solid was washed with MeOH (50 mL). The filtrate was concentrated under reduced pressure and used in the next step without further purification.

### 4-Amino-3-(*tert*-butyl)-1*H*-pyrazole-5-carboxamide (**30c**)

Oxalyl chloride (6.16 mL, 70.4 mmol) was added dropwise to a suspension of **28c** (5.00 g, 23.5 mmol) in DCM (240 mL) containing DMF (0.082 mL, 1.1 mmol) under nitrogen at 0 °C. The reaction mixture was stirred at 0 °C for 1 h, allowed to warm to RT, and stirred for a further 2 h. The reaction mixture was concentrated *in vacuo* and coevaporated with toluene three times. The residue was dissolved in DCM (100 mL) and added dropwise to 7 M NH_3_ in MeOH (10.1 mL, 70.4 mmol) at 0 °C. After stirring for 3 h, the reaction mixture was concentrated *in vacuo* and used in the next step without further purification. The crude intermediate **29c** was combined with 10% palladium on carbon (0.85 g, 0.80 mmol) in EtOH (90 mL) and stirred under a H_2_ gas insert at 60 °C for 6 h. The reaction mixture was filtered through Celite and the solid was washed with MeOH (50 mL). The filtrate was concentrated under reduced pressure and the residue was used in the next step without further purification.

### 4-Amino-3-ethyl-1*H*-pyrazole-5-carboxamide (**30d**)

Amide **29d** (830 mg, 4.51 mmol) and 10% palladium on carbon (200 mg) in EtOH (90 mL) were stirred under a H_2_ insert at 60 °C for 6 h. The reaction mixture was filtered and the residue was washed with MeOH (50 mL). The filtrate was concentrated *in vacuo* under reduced pressure and the residue was used for the next step without further purification.

### 4-Amino-3-methyl-1*H*-pyrazole-5-carboxamide (**30e**)

The compound was prepared from **29e** (0.10 g, 0.59 mmol) as described for **30d** to get the title compound as a white solid (57 mg, 69%). ^1^H NMR (500 MHz, DMSO-*d*_6_) δ 12.35 (s, 1H), 7.16 (s, 1H), 6.97 (s, 1H), 4.41 (s, 2H), 2.05 (s, 3H). LC-MS: *t*_R_ = 0.71 min, purity: 97%, *m*/*z* [M + H]^+^: 141. Spectral data agree with a previous report.^23^

### 4-Amino-1*H*-pyrazole-5-carboxamide (**30f**)

Amide **29f** (1.00 g, 6.41 mmol) and 10% palladium on carbon (0.20 g) in MeOH (50 mL) were stirred with a H_2_ (g) insert at 60 °C for 18 h. The reaction mixture was filtered and the residue was washed with MeOH (50 mL). After evaporation, the off-white solid was used in the next step without further purification.

### 3-Cyclopentyl-5-(4-fluorophenyl)-1,6-dihydro-7*H*-pyrazolo[4,3-*d*]pyrimidin-7-one (**31a**, NPD-3504)

The compound was prepared from **30a** as described for **11** to get the title compound as a white solid (105 mg, 22% over three steps). ^1^H NMR (600 MHz, DMSO-*d*_6_) δ 13.74 (br s, 1H), 12.32 (br s, 1H), 8.16–8.11 (m, 2H), 7.38–7.33 (m, 2H), 3.40 (p, *J* = 8.3 Hz, 1H), 2.11–2.04 (m, 2H), 1.97–1.91 (m, 2H), 1.85–1.77 (m, *J* = 4.6 Hz, 2H), 1.72–1.64 (m, 2H). ^13^C NMR (151 MHz, DMSO-*d*_6_) δ 163.5 (d, *J* = 248.2 Hz), 150.0*, 148.7*, 137.0*, 129.9 (d, *J* = 8.8 Hz), 129.8^Δ^, 115.5 (d, *J* = 21.8 Hz), 36.7^#^, 32.1, 25.1. LC-MS: *t*_R_ = 4.30 min, purity: >99%, *m*/*z* [M + H]^+^: 299; HR-MS: calcd for C_16_H_15_FN_4_O [M + H]^+^. 299.1303; found, 299.1301.

### 3-Cyclohexyl-5-(4-fluorophenyl)-1,6-dihydro-7*H*-pyrazolo[4,3-*d*]pyrimidin-7-one (**31b**, NPD-3540)

The compound was prepared from **30b** as described for **11** to get the title compound as a white solid (64 mg, 6% over three steps). ^1^H NMR (500 MHz, DMSO-*d*_6_) δ 13.76 (br s, 1H), 12.37 (br s, 1H), 8.13 (ddd, *J* = 8.7, 5.5, 2.6 Hz, 2H), 7.40–7.33 (m, 2H), 3.07–2.96 (m, 1H), 2.05–1.94 (m, 2H), 1.86–1.65 (m, 5H), 1.47–1.34 (m, 2H), 1.33–1.20 (m, 1H). ^13^C NMR (126 MHz, DMSO-*d*_6_) δ 163.6 (d, *J* = 248.4 Hz), 148.8*, 130.0 (d, *J* = 8.6 Hz), 129.8 (d, *J* = 3.2 Hz), 115.6 (d, *J* = 22.3 Hz), 35.6^#^, 31.8, 25.9, 25.7. LC-MS: *t*_R_ = 4.53 min, purity: >99%, *m*/*z* [M + H]^+^: 313; HR-MS: calcd for C_17_H_17_FN_4_O [M + H]^+^. 313.1459; found, 313.1453.

### 3-(*tert*-Butyl)-5-(4-fluorophenyl)-1,6-dihydro-7*H*-pyrazolo[4,3-*d*]pyrimidin-7-one (**31c**, NPD-3519)

The compound was prepared from **30c** as described for **11** to get the title compound as a white solid (0.71 g, 43% over three steps). ^1^H NMR (600 MHz, DMSO-*d*_6_) δ 13.67 (br s, 1H), 12.43 (br s, 1H), 8.18–8.12 (m, 2H), 7.40–7.33 (m, 2H), 1.49 (s, 9H). ^13^C NMR (151 MHz, DMSO-*d*_6_) δ 163.6 (d, *J* = 248.2 Hz), 154.2*, 153.3*, 148.1*, 136.7, 129.9 (d, *J* = 8.3 Hz), 129.7^Δ^, 126.3, 115.6 (d, *J* = 21.7 Hz), 32.8*, 29.5. LC-MS: *t*_R_ = 4.34 min, purity: >99%, *m*/*z* [M + H]^+^: 287; HR-MS: calcd for C_15_H_15_FN_4_O [M + H]^+^. 287.1303; found, 287.1293.

### 3-Ethyl-5-(4-fluorophenyl)-1,6-dihydro-7*H*-pyrazolo[4,3-*d*]pyrimidin-7-one (**31d**, NPD-3500)

The compound was prepared from **30d** as described for **11** to get the title compound as a white solid (0.14 g, 33% over three steps). ^1^H NMR (600 MHz, DMSO-*d*_6_) δ 13.78 (br s, 1H), 12.30 (br s, 1H), 8.17–8.11 (m, 2H), 7.38–7.31 (m, 2H), 2.88 (q, *J* = 7.6 Hz, 2H), 1.33 (t, *J* = 7.6 Hz, 3H). ^13^C NMR (151 MHz, DMSO-*d*_6_) δ 163.5 (d, *J* = 248.2 Hz), 149.1*, 147.2*, 137.1*, 130.0 (d, *J* = 8.8 Hz), 129.9^Δ^, 115.5 (d, *J* = 21.8 Hz), 19.2^#^, 13.3. LC-MS: *t*_R_ = 3.52 min, purity: >99%, *m*/*z* [M + H]^+^: 259; HR-MS: calcd for C_13_H_11_FN_4_O [M + H]^+^. 259.0990; found, 259.0982.

### 5-(4-Fluorophenyl)-3-methyl-1,6-dihydro-7*H*-pyrazolo[4,3-*d*]pyrimidin-7-one (**31e**, NPD-3224)

The compound was prepared from **30e** as described for **11** to get the title compound as a white solid (0.85 g, 23% over three steps). ^1^H NMR (500 MHz, DMSO-*d*_6_) δ 13.83 (br s, 1H), 12.35 (br s, 1H), 8.18–8.11 (m, 2H), 7.38–7.31 (m, 2H), 2.44 (s, 3H). ^13^C NMR (126 MHz, DMSO-*d*_6_) δ 164.0 (d, *J* = 248.2 Hz), 149.7, 130.4 (d, *J* = 8.7 Hz), 130.3 (d, *J* = 1.6 Hz), 115.9 (d, *J* = 21.9 Hz), 22.2. LC-MS: *t*_R_ = 3.14 min, purity: 96%, *m*/*z* [M + H]^+^: 245; HR-MS: calcd for C_12_H_9_FN_4_O [M + H]^+^. 245.0833; found, 245.0827.

### 5-(4-Fluorophenyl)-1,6-dihydro-7*H*-pyrazolo[4,3-*d*]pyrimidin-7-one Formate (**31f**, NPD-3223)

The compound was prepared from **30f** as described for **11** to get the title compound as a white solid (43 mg, 16% over three steps). ^1^H NMR (500 MHz, DMSO-*d*_6_) δ 13.13 (s, 1H), 8.54 (s, 1H), 8.32–8.25 (m, 2H), 7.80 (s, 1H), 7.22–7.15 (m, 2H). ^13^C NMR (126 MHz, DMSO-*d*_6_) δ 165.4, 163.6*, 162.6 (d, *J* = 244.5 Hz), 156.4*, 141.8*, 131.7^#^, 129.5 (d, *J* = 8.2 Hz), 114.4 (d, *J* = 21.1 Hz). LC-MS: *t*_R_ = 2.91 min, purity: 99%, *m*/*z* [M + H]^+^: 231; HR-MS: calcd for C_11_H_7_FN_4_O [M + Na]^+^. 253.0496; found, 253.0493.

### Ethyl 4-Amino-3-phenyl-1*H*-pyrazole-5-carboxylate (**33**)

To an ice-cooled solution of NaOEt (0.61 g, 9.0 mmol) in toluene (15.0 mL) was added benzyl cyanide (**32**, 1.0 mL, 9.0 mmol) dropwise over 30 min. The reaction mixture was stirred at 0 °C for 30 min and then, 15% ethyl 2-diazoacetate (4.4 mL, 35 mmol) in toluene was added dropwise over 15 min. The reaction mixture was allowed to warm to RT and stirred for 16 h, after which the reaction mixture was neutralized with CO_2_, and extracted with EtOAc (3 × 50 mL). The combined organic layers were concentrated *in vacuo* and purified by flash column chromatography on silica gel with a gradient elution of EtOAc in cyclohexane (20–60%) to get the title compound as a brown oil (0.40 g, 20%). ^1^H NMR (600 MHz, CDCl_3_) δ 10.26 (br s, 1H), 7.69 (d, *J* = 7.3 Hz, 2H), 7.49–7.44 (m, 2H), 7.36 (tt, *J* = 7.1, 1.2 Hz, 1H), 4.42 (q, *J* = 7.1 Hz, 2H), 4.30 (s, 2H), 1.42 (t, *J* = 7.1 Hz, 3H). ^13^C NMR (151 MHz, CDCl_3_) δ 160.9*, 132.7, 132.0, 129.4, 129.2, 128.0, 126.6, 60.9, 14.6. LC-MS: *t*_R_ = 3.51 min, purity: 99%, *m*/*z* [M + H]^+^: 232. Spectral data agree with a previous report.^28^

### 5-(4-Fluorophenyl)-3-phenyl-1,6-dihydro-7*H*-pyrazolo[4,3-*d*]pyrimidin-7-one (**35**, NPD-3640)

To a solution of **33** (0.40 g, 1.7 mmol) and PyBroP (0.89 g, 1.9 mmol) in DCE (5.0 mL) was added 4-fluorobenzoic acid (0.24 g, 1.7 mmol) and TEA (0.48 mL, 3.5 mmol). The reaction was heated under microwave irradiation at 120 °C for 30 min, concentrated *in vacuo*, and purified by flash column chromatography on silica gel with a gradient elution of EtOAc in cyclohexane (10–30%) to get the amide intermediate as a white solid (0.54 g), which was used in the next step without further purification. A mixture of the amide intermediate (0.54 g) and 7 M NH_3_ in MeOH (6.6 mL, 46 mmol) was heated under microwave irradiation at 100 °C for 3 d, concentrated *in vacuo*, and used in the next step with no further purification and analysis. To a solution of **34** (0.30 g) in ^*i*^PrOH (10 mL) was added KO^*t*^Bu (0.21 g, 1.9 mmol) and heated using microwave irradiation at 120 °C for 85 min. The reaction mixture was concentrated *in vacuo*, after which it was purified with reverse phase column chromatography (MeCN/H_2_O from 1:9 to 9:1 with 0.1% HCOOH) to get the title compound as a white solid (85 mg, 17% over three steps). ^1^H NMR (500 MHz, DMSO-*d*_6_) δ 14.36 (s, 1H), 12.59 (s, 1H), 8.40–8.34 (m, 2H), 8.27–8.19 (m, 2H), 7.56–7.49 (m, 2H), 7.45–7.36 (m, 3H). ^13^C NMR (126 MHz, DMSO-*d*_6_) δ 163.7 (d, *J* = 248.6 Hz), 150.1, 132.4, 130.3 (d, *J* = 8.9 Hz), 129.6 (d, *J* = 3.0 Hz), 128.8, 128.1, 126.0, 115.7 (d, *J* = 21.9 Hz). LC-MS: *t*_R_ = 4.27 min, purity: >99%, *m*/*z* [M + H]^+^: 307; HR-MS: calcd for C_17_H_11_FN_4_O [M + Na]^+^. 329.0809; found, 329.0815.

## Supporting Information Available

*In vitro* metabolism: intrinsic clearance of **11**, **31a**–**c** and **35** in comparison with **2**; measured brain concentrations of **31c** in the pharmacokinetic experiments at 24 h after treatment; detection of parasite infection by SL-RNA detection by qPCR; LCMS, and ^1^H NMR, ^13^C NMR, and 1D NOESY spectroscopy data (PDF)
Molecular formula strings and associated biochemical data of **2**, **7**, **11**, **15**, **17**, **21a**–**f**, **22**, **31a**–**f**, and **35** (CSV)

## Abbreviations

b.i.d.: twice a day
CDD: Collaborative Drug Discovery
dpi: days post infection
HAT: human African Trypanosomiasis
MRC-5: medical research council cell strain 5
NPD: neglected parasitic disease
PDE: phosphodiesterase
PMM: peritoneal macrophage
RT: room temperature
s.i.d.: once a day
WHO: World Health Organization
