# Supplementary material for: Lead Optimization of the 5-Phenylpyrazolopyrimidinone NPD-2975 toward Compounds with Improved Antitrypanosomal Efficacy
Source: J Med Chem. 2024 Feb 8;67(4):2849–63. doi: 10.1021/acs.jmedchem.3c01976 (PMC10895668; doi:10.1021/acs.jmedchem.3c01976)
Supplement: Supplementary file 1 — jm3c01976_si_001.pdf [file jm3c01976_si_001.pdf]

Supporting information

Lead optimization of the 5-phenylpyrazolopyrimidinone NPD-2975 towards compounds with improved antitrypanosomal efficacy

Yang Zheng,[a,1] Magali van den Kerkhof,[b,1] Mohamed Ibrahim,[a] Iwan J.P. de Esch,[a] Louis Maes,[b] Geert Jan Sterk,[a] Guy Caljon[b,*] and Rob Leurs[a,*]

[a] Amsterdam Institute of Molecular and Life Sciences, Division of Medicinal Chemistry, Faculty of Science, Vrije Universiteit Amsterdam, De Boelelaan 1108, 1081 HZ Amsterdam, the Netherlands.

[b] Laboratory of Microbiology, Parasitology and Hygiene (LMPH), University of Antwerp, Universiteitsplein 1, 2610 Wilrijk, Belgium.

[1] Authors contributed equally to this manuscript.

* Corresponding author: guy.caljon@uantwerpen.be (Guy Caljon); r.leurs@vu.nl (Rob Leurs).

Table of contents

Table S1. *In vitro* metabolism: intrinsic clearance of **11**, **31a-c** and **35** in comparison with **2**. Page S2

Table S2. Measured brain concentrations of **31c** in the pharmacokinetic experiments at 24 h after treatment. Page S3

Figure S1. Detection of parasite infection by SL-RNA detection by qPCR Page S4

Figure S2-S141. LCMS, ^{1}H NMR, ^{13}C NMR and 1D NOESY spectroscopy data. Page S5-101

Table S1. *In vitro* metabolism: intrinsic clearance of **11**, **31a-c** and **35** in comparison with **2**.

Species	Phase I/II	Time	**2 (%)**		**11 (%)**		**31a (%)**		**31b** (%)		**31c (%)**		**35 (%)**	
			Mean	STEDV	Mean	STEDV	Mean	STEDV	Mean	STEDV	Mean	STEDV	Mean	STEDV
Mouse	I	0	100	-	100	-	100	-	100	-	100	-	100	-
		15	73	3.9	69	3	2.7	1	43	11	108	11.8	113	7.5
		30	55	2.6	54	1.5	0.8	0.02	22	6.4	105	3	110	5.9
		60	33	4.8	31	0.4	0.6	0.2	7	1.8	105	8.8	110	3.6
	II	0	100	-	100	-	100	-	100	-	100	-	100	-
		15	93	6	102	2.4	85	13.4	107	0.7	94	1.2	56	2
		30	81	3.9	108	15	83	15.2	99	11.3	95	8.7	25	3.4
		60	71	7.6	97	8.9	70	4.2	83	0.1	90	11	10	1.5
Human	I	0	100	-	100	-	100	-	100	-	100	-	100	-
		15	100	12	97	2.8	96	4.5	89	4.4	112	10.1	113	2.5
		30	101	5.6	93	2.8	103	13.6	90	3.9	117	13.7	107	4
		60	93	8.5	91	5.5	87	3.5	86	5.8	114	7	109	7
	II	0	100	-	100	-	100	-	100	-	100	-	100	-
		15	74	0.8	100	5.1	81	0.7	99	12.9	100	3.5	82	8.4
		30	75	6.2	98	1.7	79	3.3	118	3	96	8.9	67	8
		60	69	2	99	3.8	82	10.8	95	0.6	99	7.7	46	0.1

Table S2. Measured brain concentrations of **31c** in the pharmacokinetic experiments at 24 h after treatment.

Compound	Administration route	Mouse	Brain levels (ng/mL)	Stdev	Brain levels (µM)	Stdev
**31c**	PO	m1	24	23	0.088	0.083
		m2	188	80	0.690	0.294
		m3	30	28	0.111	0.103
	IP	m1	11	4	0.040	0.016
		m2	8	-	0.029	-
		m3	91	101	0.335	0.370

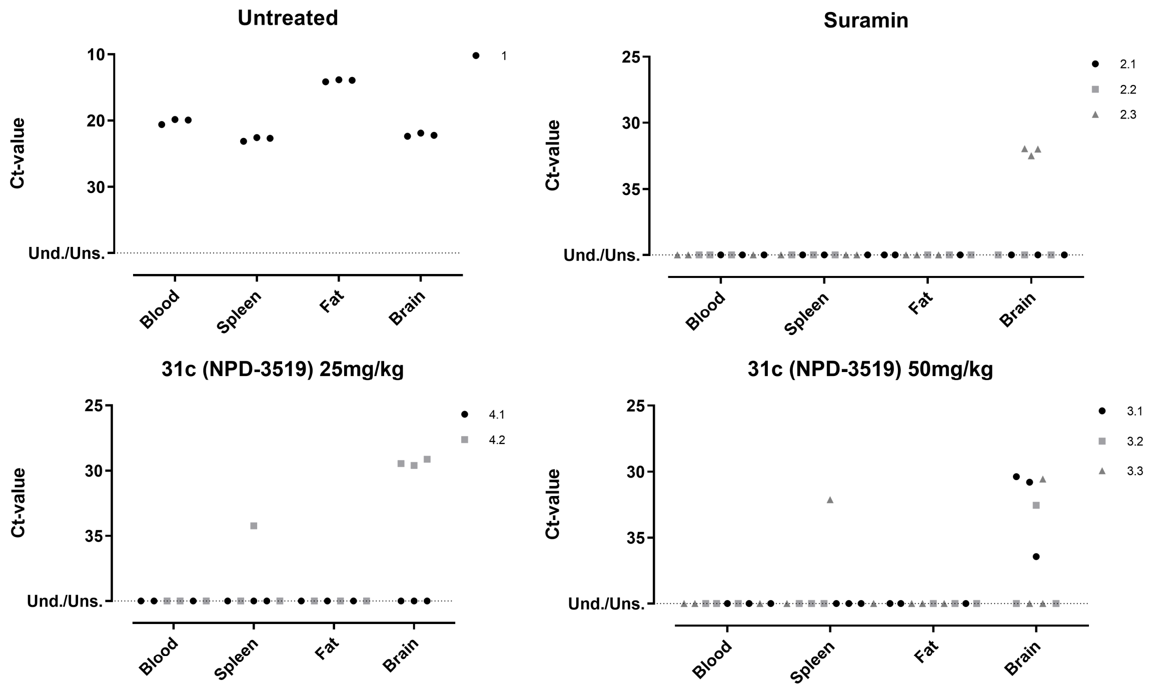

Figure S1. Ct-values of the SL-RNA qPCR of various organs collected from treated animals after infection with *T. brucei*. Results are expressed as the Ct-value for each individual measurement. Results were obtained from three independent repeats. Und./Uns.: Undetected or unspecific.

Acquired by	: Admin
Date Acquired	: 20/8/2018 4:45:22 PM
Sample Name	: YAZH01-240-1
Sample ID	:
Tray#	: 1
Vial#	: 17
Injection Volume	: 2
Data File	: C:\LabSolutions\Data\2018\2018-wk34\YAZH01-240-1.lcd
Background File	: blanco 200818.lcd
Method File	: Method SCAN ACID standard.lcm
Report Format	: DefaultLCMS.lcr
Tuning File	: C:\LabSolutions\Tuning\Tuning-ESI-pos-neg01072015a.lct
Processed by	: Admin
Modified Date	: 21/8/2018 9:01:55 AM

Chromatogram
YAZH01-240-1 C:\LabSolutions\Data\2018\2018-wk34\YAZH01-240-1.lcd

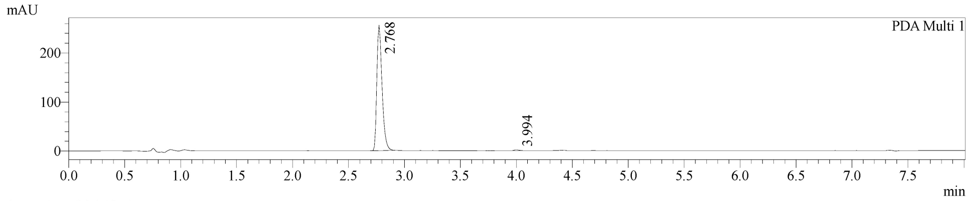

1 PDA Multi 1 / 254nm 4nm

Peak#	Name	Ret. Time	Area	Area %
1		2.768	833110	99.270
2		3.994	6128	0.730

MS Chromatogram
YAZH01-240-1 C:\LabSolutions\Data\2018\2018-wk34\YAZH01-240-1.lcd

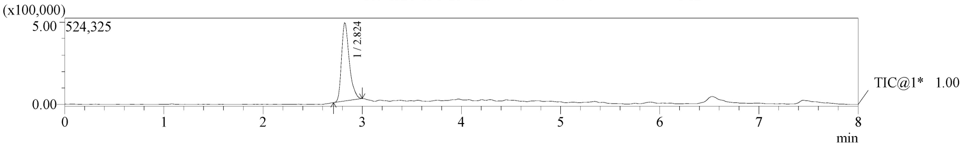

MS Spectrum Graph

#:1 Ret.Time:Averaged 2.810-2.830(Scan#:282-284)
Mass Peaks:10 Base Peak:213.00(333159) Polarity:Pos Segment1 - Event1

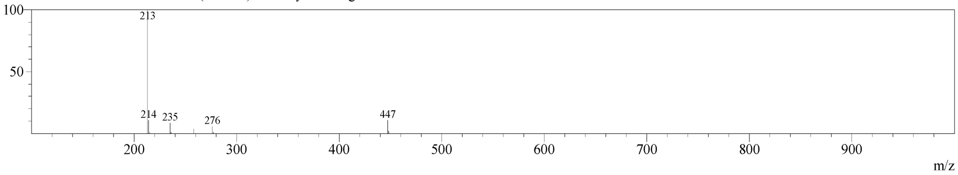

MS Spectrum Table

#:1 Ret.Time:
BG Mode:Calc 2.710<->3.000(272<->301)
Mass Peaks:10 Base Peak:213.00(333159) Polarity:Pos Segment1 - Event1

#	m/z	Abs.Inten.	Rel.Inten.	Charge	Polarity	Monoisotopic
1	213.00	333159	100.00			
2	214.05	35617	10.69			
3	215.10	3891	1.17			
4	235.10	28364	8.51			
5	236.00	3950	1.19			
6	258.15	12526	3.76			
7	276.10	19140	5.75			
8	277.15	3742	1.12			
9	447.25	35732	10.73			
10	448.15	6737	2.02			

Figure S2. LCMS spectrum of compound **4**.

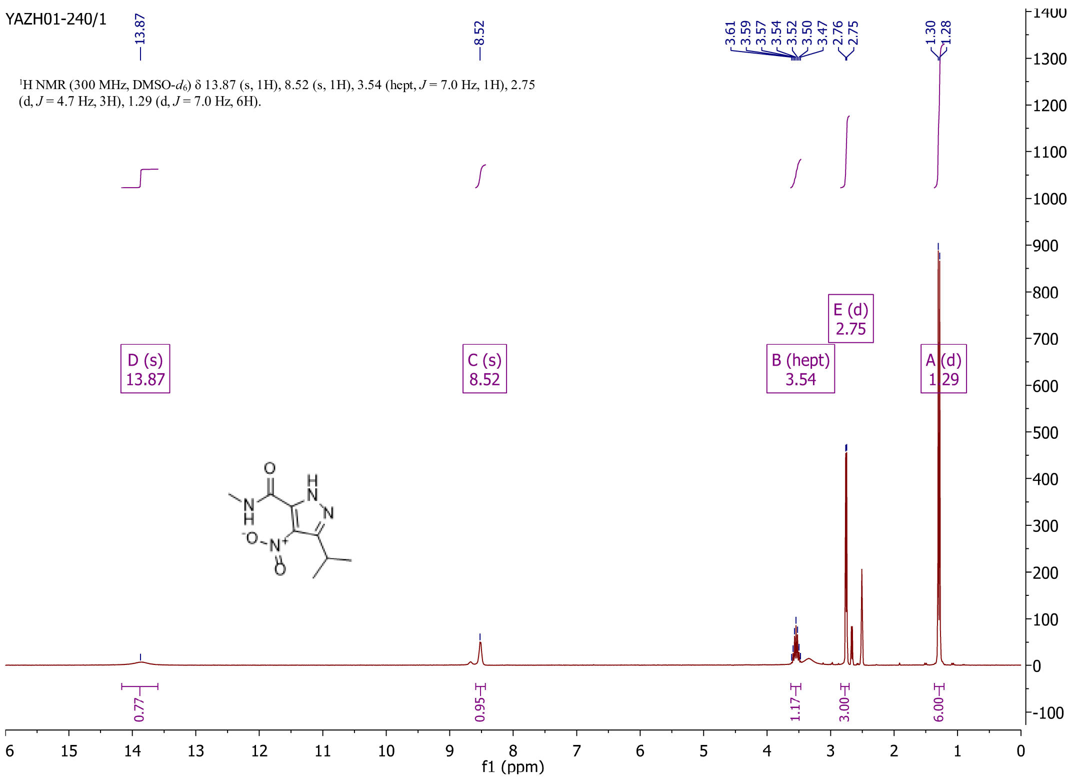

Figure S3. ^{1}H NMR spectrum of compound **4**.

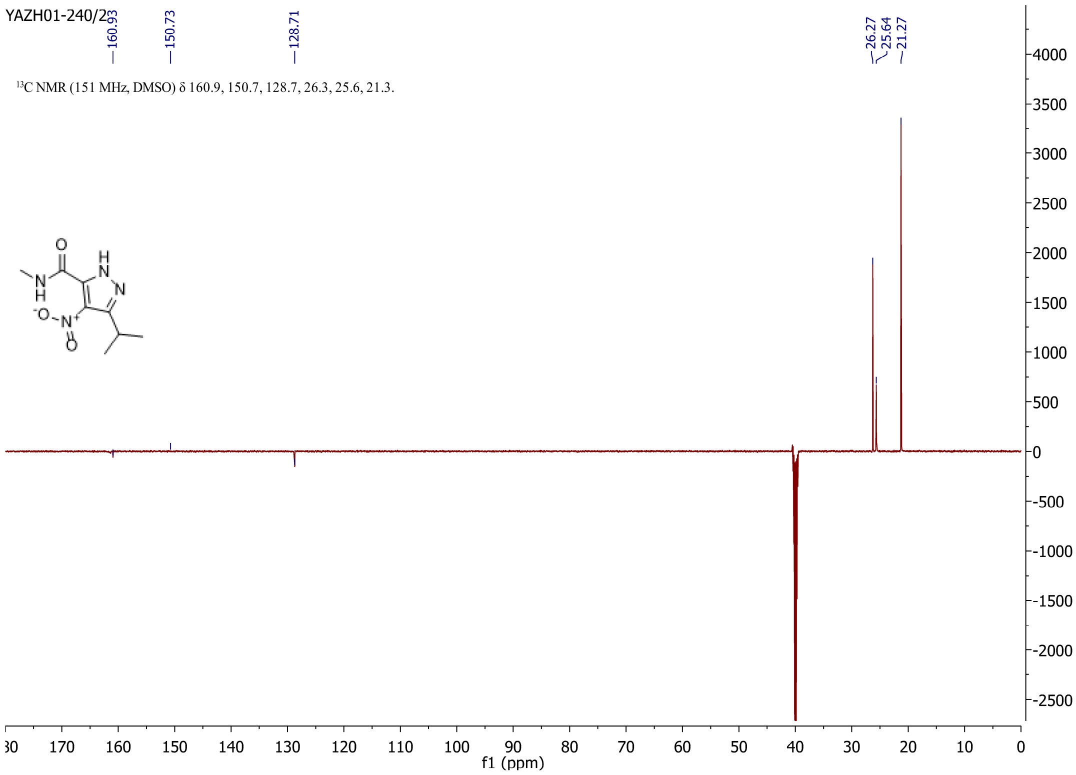

Figure S4. ^{13}C NMR spectrum of compound **4**.

Acquired by	: Admin
Date Acquired	: 4/6/2019 12:23:28 PM
Sample Name	: YAZH01-246
Sample ID	:
Tray#	: 1
Vial#	: 14
Injection Volume	: 2
Data File	: C:\LabSolutions\Data\2019\2019-wk23\YAZH01-246.lcd
Background File	:
Method File	: Method SCAN BASE standard.lcm
Report Format	: DefaultLCMS.lcr
Tuning File	: C:\LabSolutions\Tuning\Tuning-ESI-pos-neg01072015a.lct
Processed by	: Admin
Modified Date	: 4/6/2019 1:37:50 PM

PDA Graph

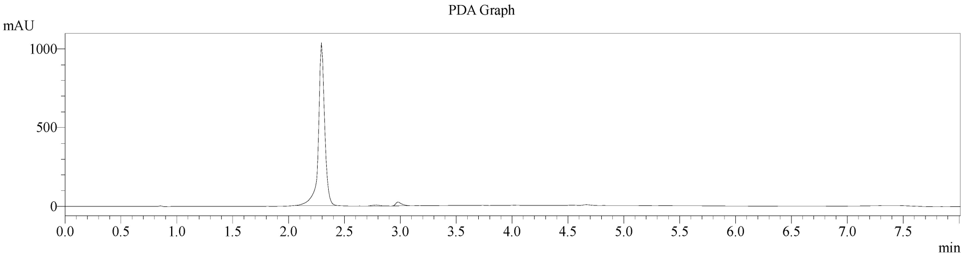

PeakTable

PDA Ch1 254nm 4nm

Peak#	Name	Ret. Time	Area	Area %
1		2.289	4043163	97.221
2		2.779	28032	0.674
3		2.974	87555	2.105

MS Chromatogram

YAZH01-246 C:\LabSolutions\Data\2019\2019-wk23\YAZH01-246.lcd

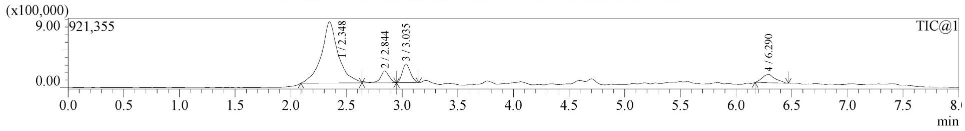

MS Spectrum Graph

#:1 Ret.Time:Averaged 2.340-2.360(Scan#:235-237)
BG Mode:Calc 2.090<->2.640(210<->265)
Mass Peaks:6 Base Peak:183.00(630062) Polarity:Pos Segment1 - Event1

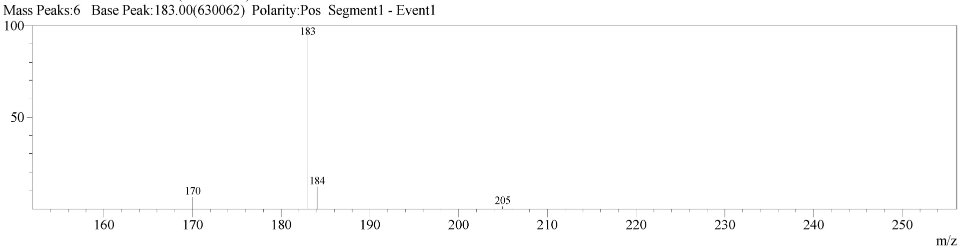

MS Spectrum Table

#:1 Ret.Time:
BG Mode:Calc 2.090<->2.640(210<->265)
Mass Peaks:6 Base Peak:183.00(630062) Polarity:Pos Segment1 - Event1

#	m/z	Abs.Inten.	Rel.Inten.	Charge	Polarity	Monoisotopic
1	151.95	17274	2.74			
2	170.00	40497	6.43			
3	183.00	630062	100.00			
4	184.05	75227	11.94			
5	204.95	7757	1.23			
6	256.15	16405	2.60			

Figure S5. LCMS spectrum of compound **5**.

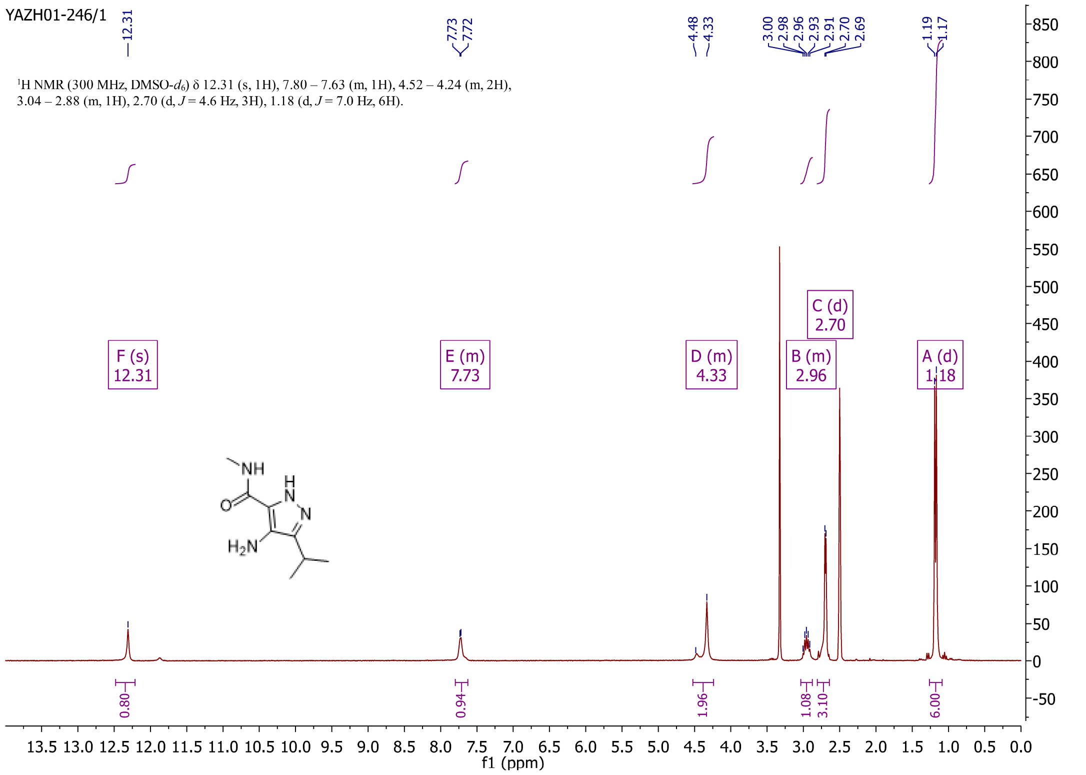

Figure S6. ^{1}H NMR spectrum of compound **5**.

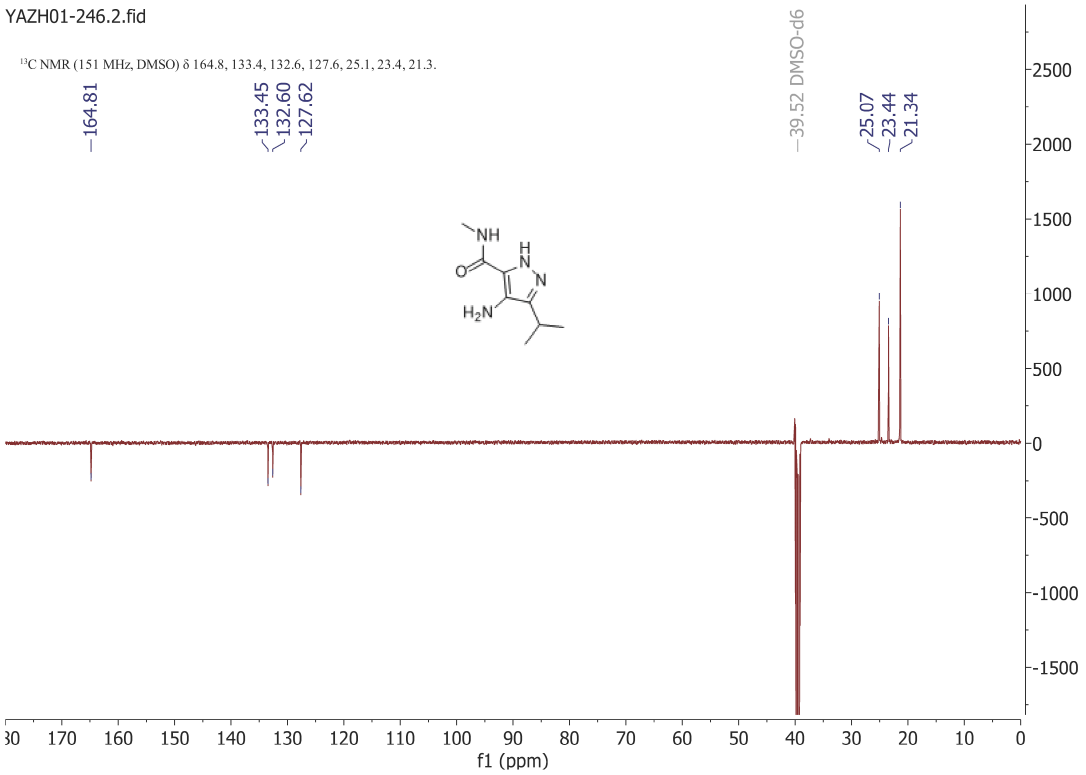

Figure S7. ^{13}C NMR spectrum of compound **5**.

Acquired by	: Admin
Date Acquired	: 13/3/2019 4:10:07 PM
Sample Name	: YAZH_28_20190313
Sample ID	:
Tray#	: 1
Vial#	: 18
Injection Volume	: 2
Data File	: C:\LabSolutions\Data\2019\2019-wk10\YAZH_28_20190313.lcd
Background File	: blanco 13032019.lcd
Method File	: Method SCAN ACID standard.lcm
Report Format	: DefaultLCMS.lcr
Tuning File	: C:\LabSolutions\Tuning\Tuning-ESI-pos-neg01072015a.lct
Processed by	: Admin
Modified Date	: 13/3/2019 4:23:24 PM

Chromatogram
YAZH_28_20190313 C:\LabSolutions\Data\2019\2019-wk10\YAZH_28_20190313.lcd

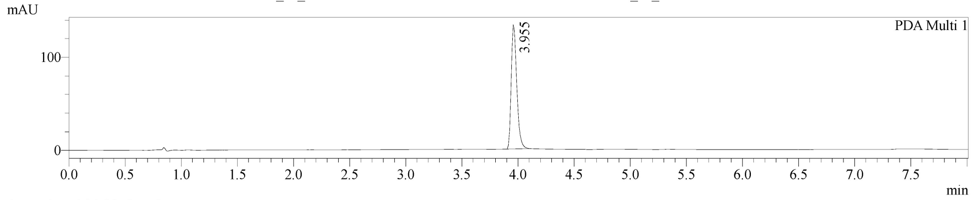

1 PDA Multi 1 / 254nm 4nm

Peak#	Name	Ret. Time	Area	Area %
1		3.955	460523	100.000

MS Chromatogram
YAZH_28_20190313 C:\LabSolutions\Data\2019\2019-wk10\YAZH_28_20190313.lcd

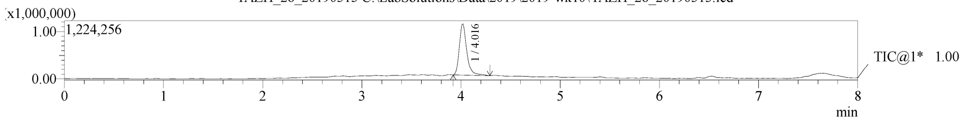

MS Spectrum Graph
#:1 Ret.Time:Averaged 4.010-4.030(Scan#:402-404)
Mass Peaks:8 Base Peak:287.10(825619) Polarity:Pos Segment1 - Event1

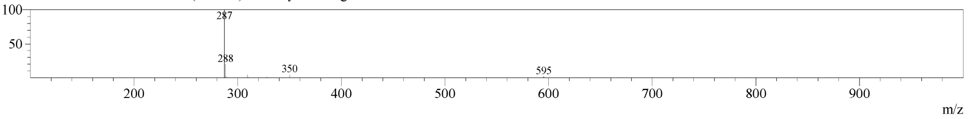

MS Spectrum Table
#:1 Ret.Time:
BG Mode:Calc 3.920<->4.290(393<->430)
Mass Peaks:8 Base Peak:287.10(825619) Polarity:Pos Segment1 - Event1

#	m/z	Abs.Inten.	Rel.Inten.	Charge	Polarity	Monoisotopic	#	m/z	Abs.Inten.	Rel.Inten.	Charge	Polarity	Monoisotopic
1	287.10	825619	100.00				5	310.00	9548	1.16			
2	288.10	162597	19.69				6	328.10	9457	1.15			
3	289.10	14314	1.73				7	350.10	35884	4.35			
4	309.05	36108	4.37				8	595.30	14294	1.73			

Figure S8. LCMS spectrum of compound **7**.

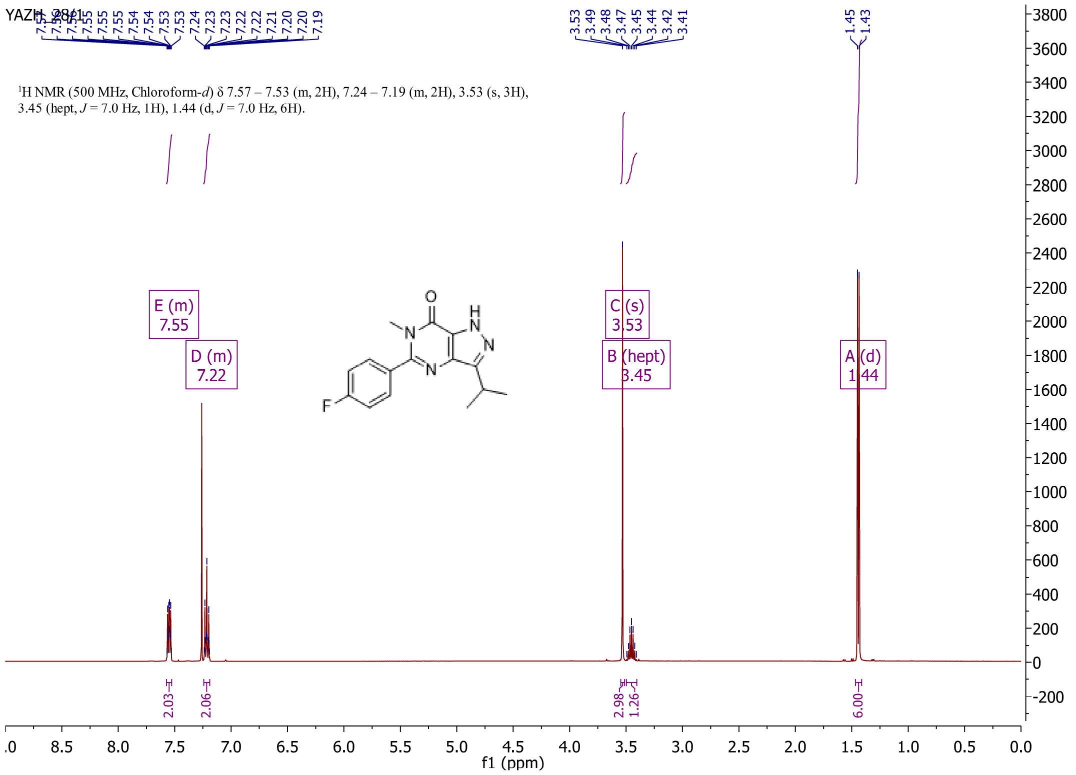

Figure S9. ^{1}H NMR spectrum of compound **7**.

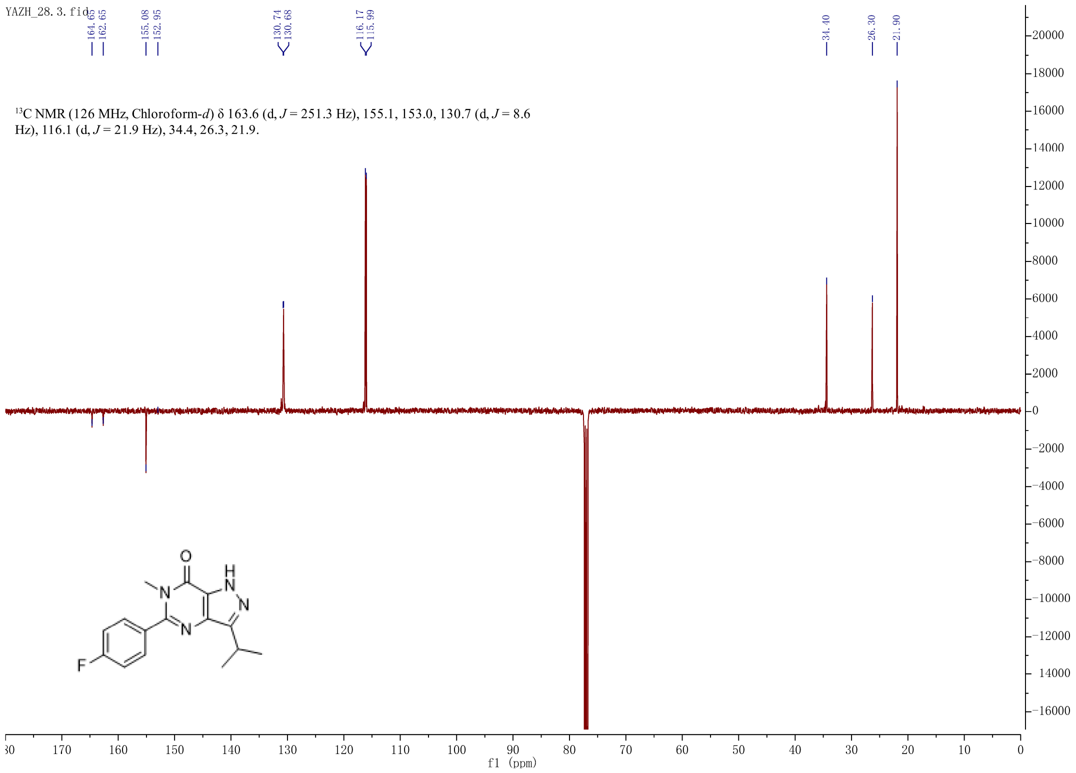

Figure S10. ^{13}C NMR spectrum of compound **7**.

Acquired by	: Admin
Date Acquired	: 3/9/2018 2:19:49 PM
Sample Name	: YAZH01-248-1
Sample ID	:
Tray#	: 1
Vial#	: 13
Injection Volume	: 1
Data File	: C:\LabSolutions\Data\2018\2018-wk36\YAZH01-248-1.lcd
Background File	: blanco 03092018.lcd
Method File	: Method SCAN ACID standard MW50.lcm
Report Format	: DefaultLCMS.lcr
Tuning File	: C:\LabSolutions\Tuning\Tuning-ESI-pos-neg01072015a.lct
Processed by	: Admin
Modified Date	: 3/9/2018 2:52:36 PM

Chromatogram
YAZH01-248-1 C:\LabSolutions\Data\2018\2018-wk36\YAZH01-248-1.lcd

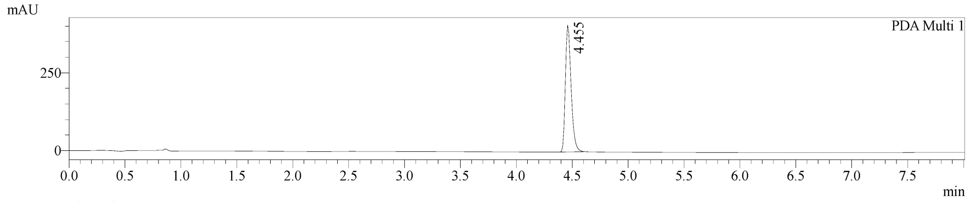

1 PDA Multi 1 / 254nm 4nm

Peak#	Name	Ret. Time	Area	Area %
1		4.455	1443459	100.000

MS Chromatogram
YAZH01-248-1 C:\LabSolutions\Data\2018\2018-wk36\YAZH01-248-1.lcd

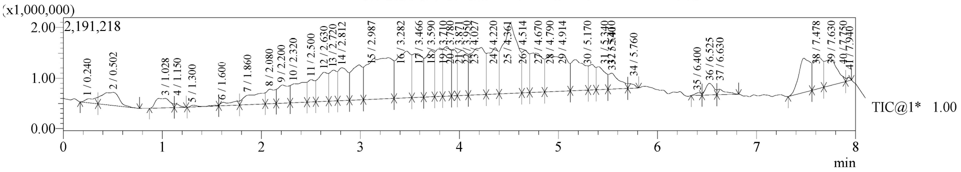

MS Spectrum Graph
#:1 Ret.Time:Averaged 4.500-4.520(Scan#:451-453)
Mass Peaks:11 Base Peak:228.05(343753) Polarity:Pos Segment1 - Event1

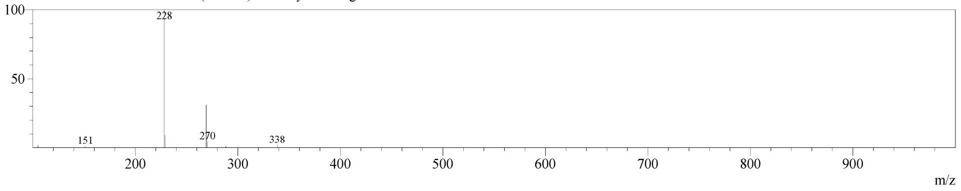

MS Spectrum Table
#:1 Ret.Time:
BG Mode:Calc 4.400<->4.630(441<->464)
Mass Peaks:11 Base Peak:228.05(343753) Polarity:Pos Segment1 - Event1

#	m/z	Abs.Inten.	Rel.Inten.	Charge	Polarity	Monoisotopic
1	59.05	9624	2.80			
2	83.05	95735	27.85			
3	84.05	3885	1.13			
4	105.05	5189	1.51			
5	151.00	3535	1.03			
6	228.05	343753	100.00			
7	229.05	31162	9.07			
8	269.15	106700	31.04			
9	270.15	15272	4.44			
10	288.30	3630	1.06			
11	338.30	5872	1.71			

Figure S11. LCMS spectrum of compound **8**.

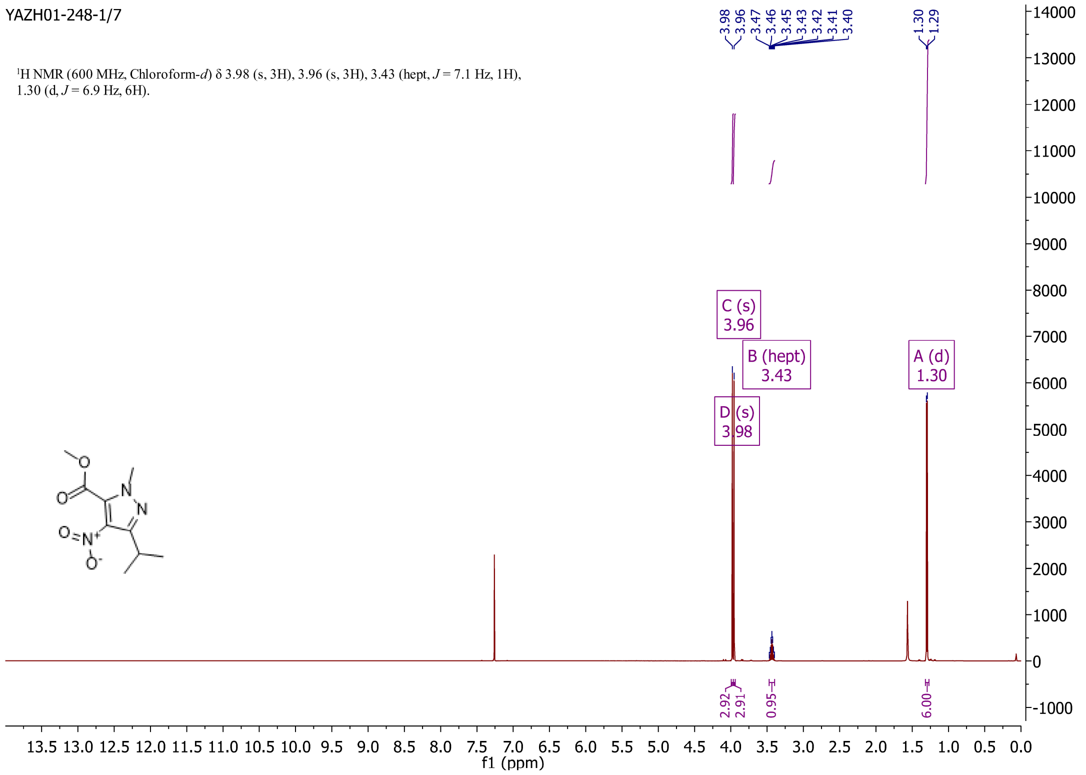

Figure S12. ^{1}H NMR spectrum of compound **8**.

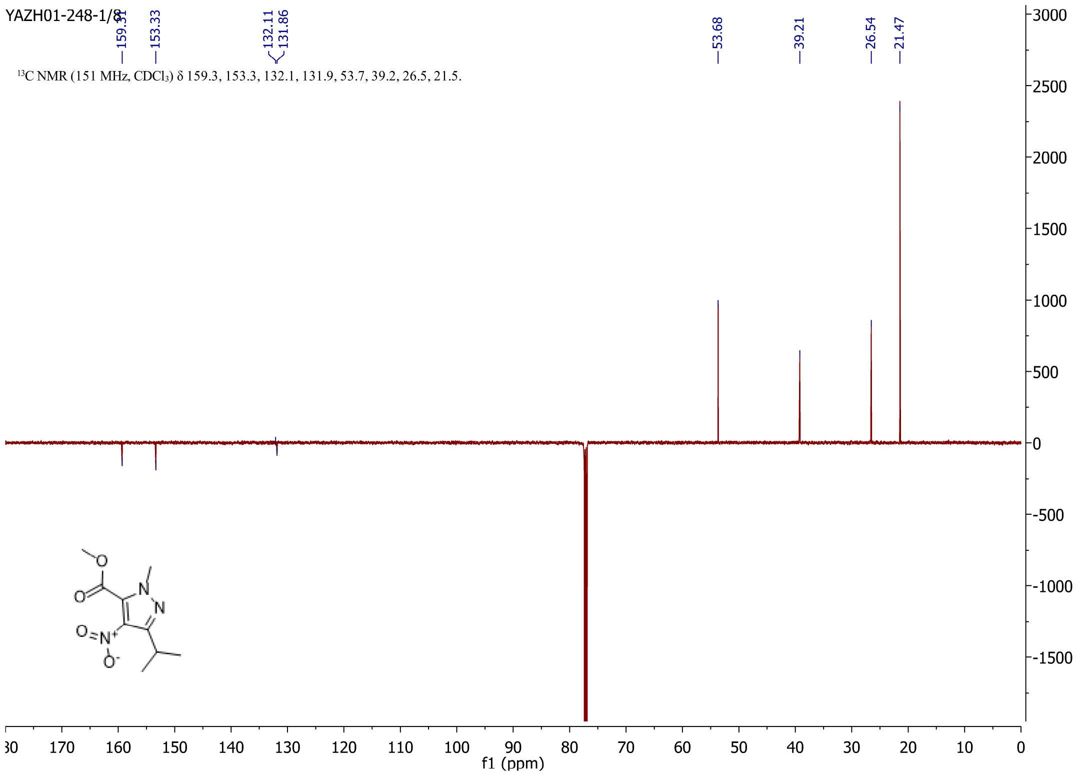

Figure S13. ^{13}C NMR spectrum of compound **8**.

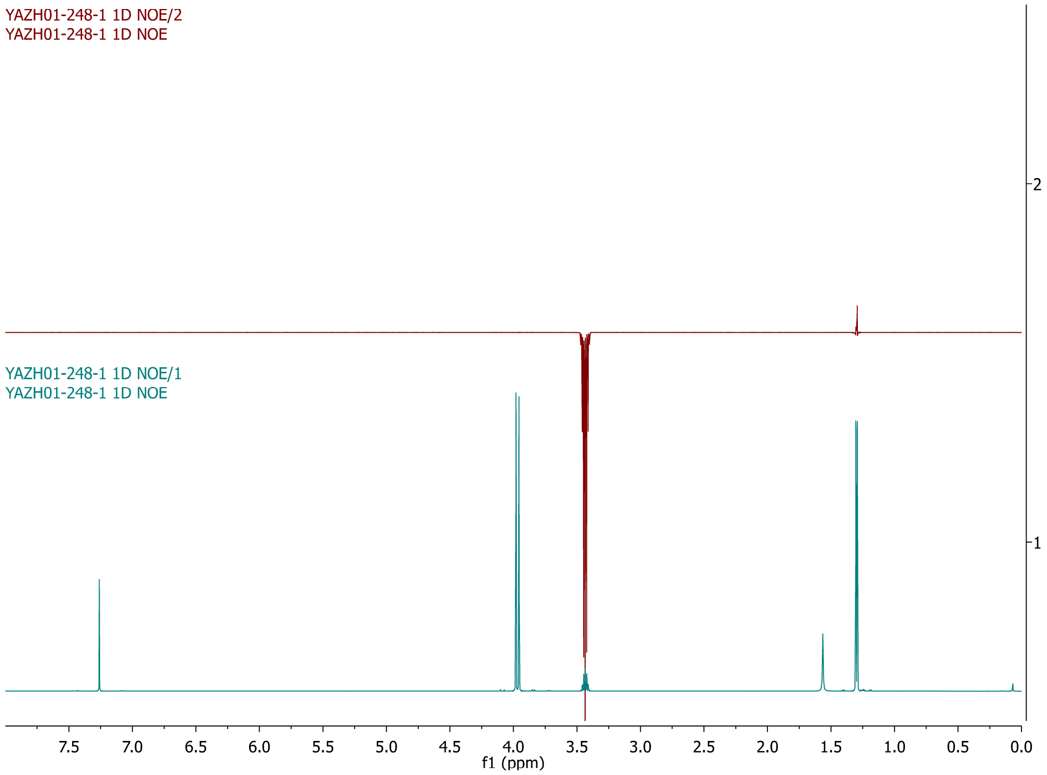

Figure S14. 1D NOESY spectrum of compound **8**.

Acquired by	: Admin
Date Acquired	: 3/9/2018 2:38:23 PM
Sample Name	: YAZH01-248-2
Sample ID	:
Tray#	: 1
Vial#	: 14
Injection Volume	: 1
Data File	: C:\LabSolutions\Data\2018\2018-wk36\YAZH01-248-2.lcd
Background File	: blanco 03092018.lcd
Method File	: Method SCAN ACID standard MW50.lcm
Report Format	: DefaultLCMS.lcr
Tuning File	: C:\LabSolutions\Tuning\Tuning-ESI-pos-neg01072015a.lct
Processed by	: Admin
Modified Date	: 3/9/2018 2:53:35 PM

Chromatogram
YAZH01-248-2 C:\LabSolutions\Data\2018\2018-wk36\YAZH01-248-2.lcd

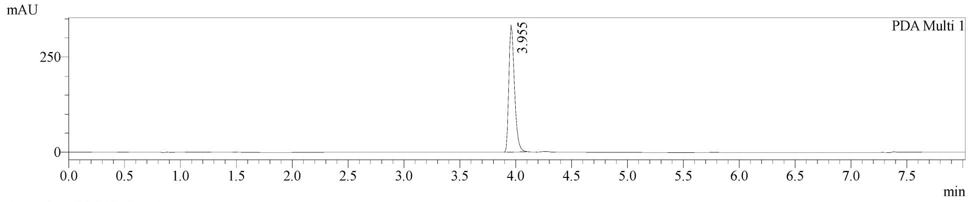

1 PDA Multi 1 / 254nm 4nm

Peak#	Name	Ret. Time	Area	Area %
1		3.955	1168339	100.000

MS Chromatogram
YAZH01-248-2 C:\LabSolutions\Data\2018\2018-wk36\YAZH01-248-2.lcd

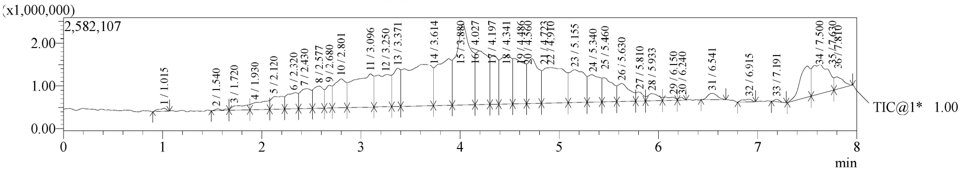

MS Spectrum Graph

#:1 Ret.Time:Averaged 4.020-4.040(Scan#:403-405)
Mass Peaks:17 Base Peak:228.05(435416) Polarity:Pos Segment1 - Event1

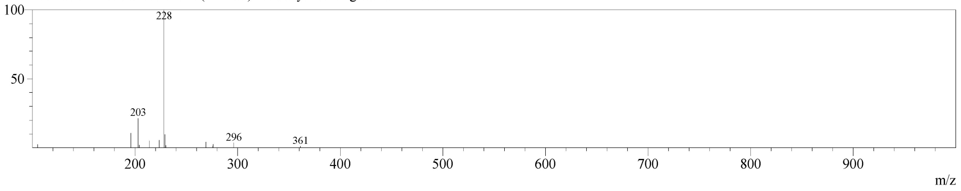

MS Spectrum Table

#:1 Ret.Time:
BG Mode:Calc 3.920<->4.150(393<->416)
Mass Peaks:17 Base Peak:228.05(435416) Polarity:Pos Segment1 - Event1

#	m/z	Abs.Inten.	Rel.Inten.	Charge	Polarity	Monoisotopic
1	59.05	11139	2.56			
2	83.05	30697	7.05			
3	105.05	10233	2.35			
4	196.00	47038	10.80			
5	203.00	93195	21.40			
6	204.20	8468	1.94			
7	214.10	23006	5.28			
8	223.50	18131	4.16			
9	223.60	24965	5.73			
10	228.05	435416	100.00			
11	229.10	43077	9.89			
12	230.05	7305	1.68			
13	269.15	17962	4.13			
14	275.50	4931	1.13			
15	276.10	10672	2.45			
16	296.10	15696	3.60			
17	361.10	4417	1.01			

Figure S15. LCMS spectrum of compound **12**.

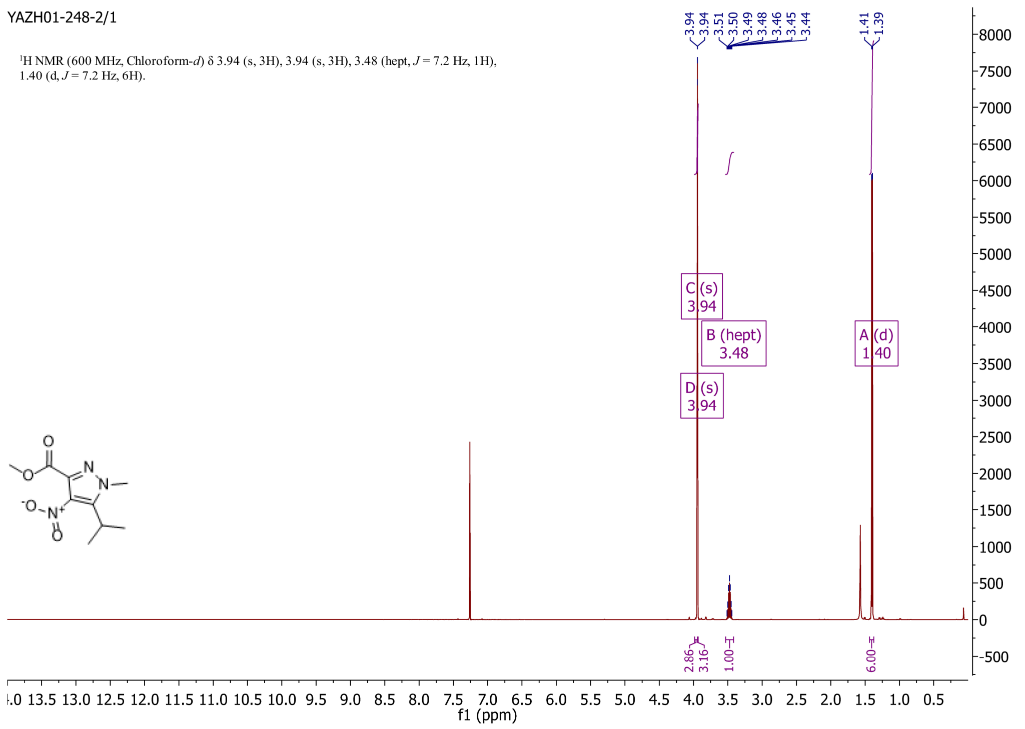

Figure S16. ^{1}H NMR spectrum of compound **12**.

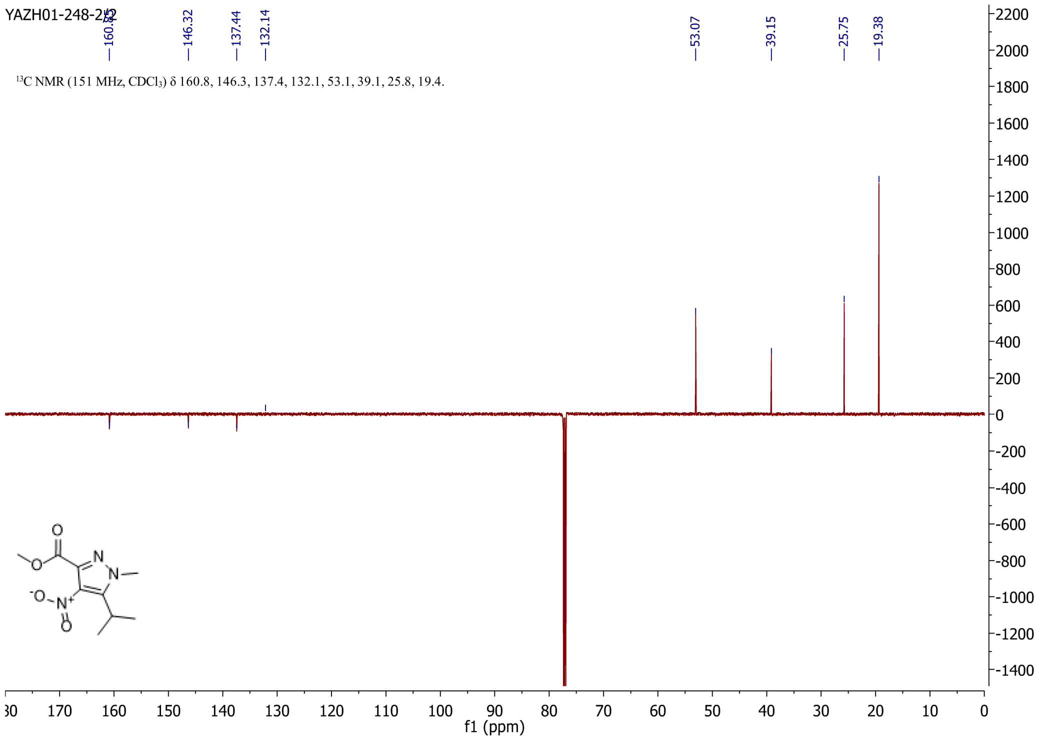

Figure S17. ^{13}C NMR spectrum of compound **12.**

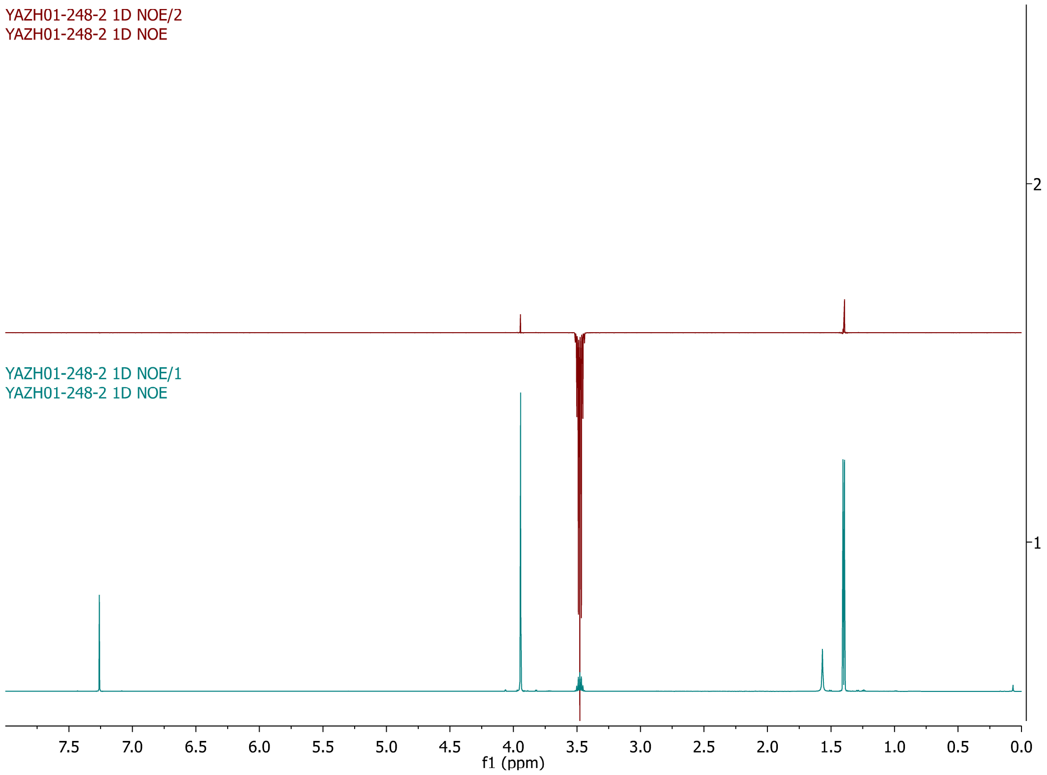

Figure S18. 1D NOESY spectrum of compound **12.**

Acquired by : Admin
Date Acquired : 13/9/2018 4:39:27 PM
Sample Name : YAZH01-257
Sample ID :
Tray# : 1
Vial# : 27
Injection Volume : 1
Data File : C:\LabSolutions\Data\2018\2018-wk37\YAZH01-257.lcd
Background File : blanco 13092018.lcd
Method File : Method SCAN ACID standard MW100.lcm
Report Format : DefaultLCMS.lcr
Tuning File : C:\LabSolutions\Tuning\Tuning-ESI-pos-neg01072015a.lct
Processed by : Admin
Modified Date : 13/9/2018 5:21:56 PM

Chromatogram
YAZH01-257 C:\LabSolutions\Data\2018\2018-wk37\YAZH01-257.lcd

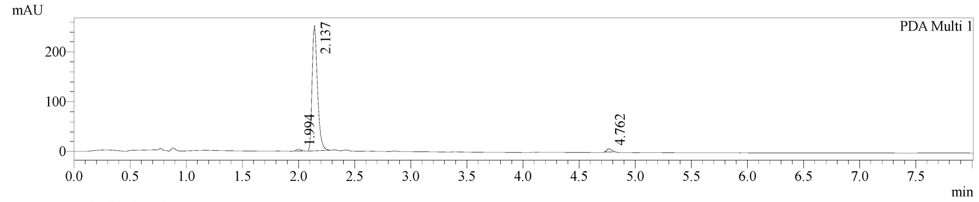

1 PDA Multi 1 / 254nm 4nm

Peak#	Name	Ret. Time	Area	Area %
1		1.994	10665	1.228
2		2.137	838126	96.509
3		4.762	19653	2.263

MS Chromatogram
YAZH01-257 C:\LabSolutions\Data\2018\2018-wk37\YAZH01-257.lcd

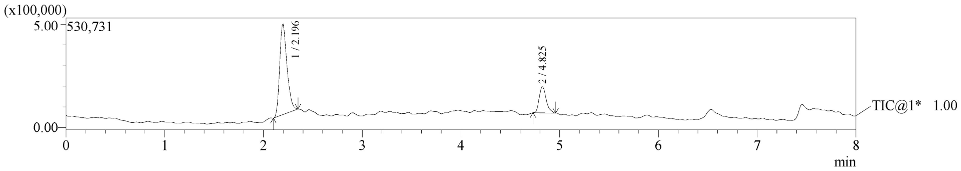

MS Spectrum Graph
#:1 Ret.Time:Averaged 2.190-2.210(Scan#:220-222)
Mass Peaks:7 Base Peak:183.05(315118) Polarity:Pos Segment1 - Event1

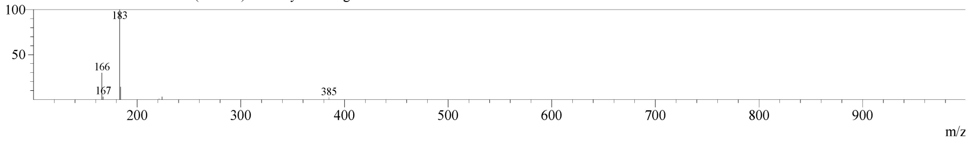

MS Spectrum Table
#:1 Ret.Time:
BG Mode:Calc 2.100<->2.350(211<->236)
Mass Peaks:7 Base Peak:183.05(315118) Polarity:Pos Segment1 - Event1

#	m/z	Abs.Inten.	Rel.Inten.	Charge	Polarity	Monoisotopic
1	166.00	92920	29.49			
2	167.10	9564	3.04			
3	183.05	315118	100.00			
4	184.05	44037	13.97			
5	221.10	3883	1.23			
6	224.05	9755	3.10			
7	385.15	6722	2.13			

Figure S19. LCMS spectrum of compound **10.**

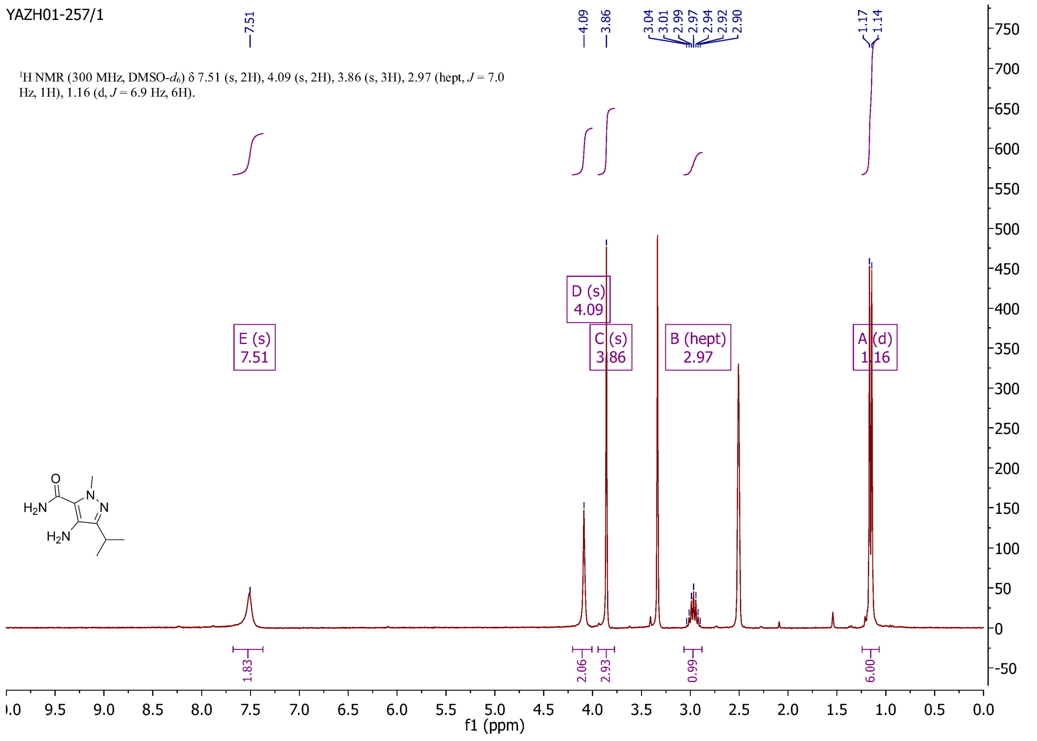

Figure S20. ^{1}H NMR spectrum of compound **10**.

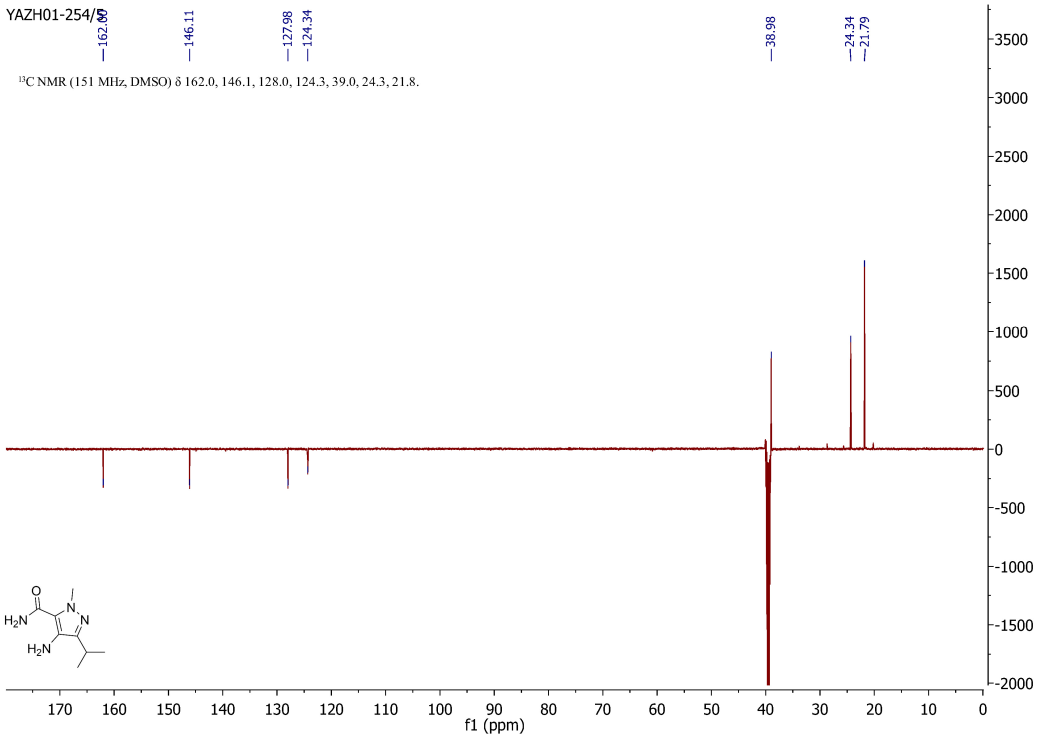

Figure S21. ^{13}C NMR spectrum of compound **10**.

Acquired by : Admin
Date Acquired : 9/10/2018 10:39:03 AM
Sample Name : YAZH01-262
Sample ID :
Tray# : 1
Vial# : 6
Injection Volume : 3
Data File : C:\LabSolutions\Data\2018\2018-wk41\YAZH01-262.lcd
Background File : blanco 09102018.lcd
Method File : Method SCAN ACID standard.lcm
Report Format : DefaultLCMS.lcr
Tuning File : C:\LabSolutions\Tuning\Tuning-ESI-pos-neg01072015a.lct
Processed by : Admin
Modified Date : 9/10/2018 11:37:02 AM

Chromatogram
YAZH01-262 C:\LabSolutions\Data\2018\2018-wk41\YAZH01-262.lcd

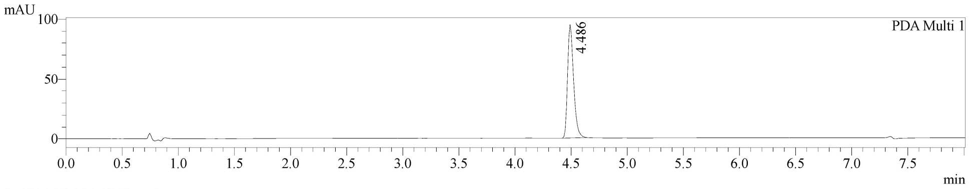

1 PDA Multi 1 / 254nm 4nm
PDA Ch1 254nm 4nm

Peak#	Name	Ret. Time	Area	Area %
1		4.486	351625	100.000

MS Chromatogram
YAZH01-262 C:\LabSolutions\Data\2018\2018-wk41\YAZH01-262.lcd

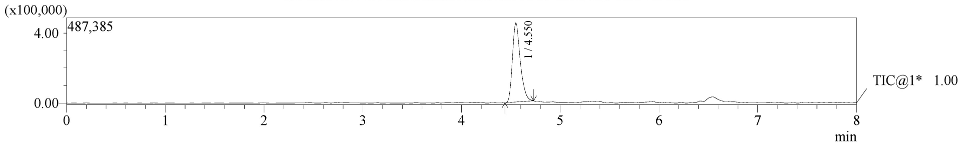

MS Spectrum Graph

#:1 Ret.Time:Averaged 4.540-4.560(Scan#:455-457)
Mass Peaks:5 Base Peak:287.05(398536) Polarity:Pos Segment1 - Event1

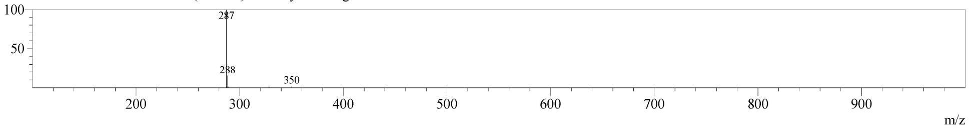

MS Spectrum Table

#:1 Ret.Time:
BG Mode:Calc 4.440<->4.730(445<->474)
Mass Peaks:5 Base Peak:287.05(398536) Polarity:Pos Segment1 - Event1

#	m/z	Abs.Inten.	Rel.Inten.	Charge	Polarity	Monoisotopic
1	287.05	398536	100.00			
2	288.15	60500	15.18			
3	289.30	5307	1.33			
4	328.15	5201	1.31			
5	350.10	7664	1.92			

Figure S22. LCMS spectrum of compound **11**.

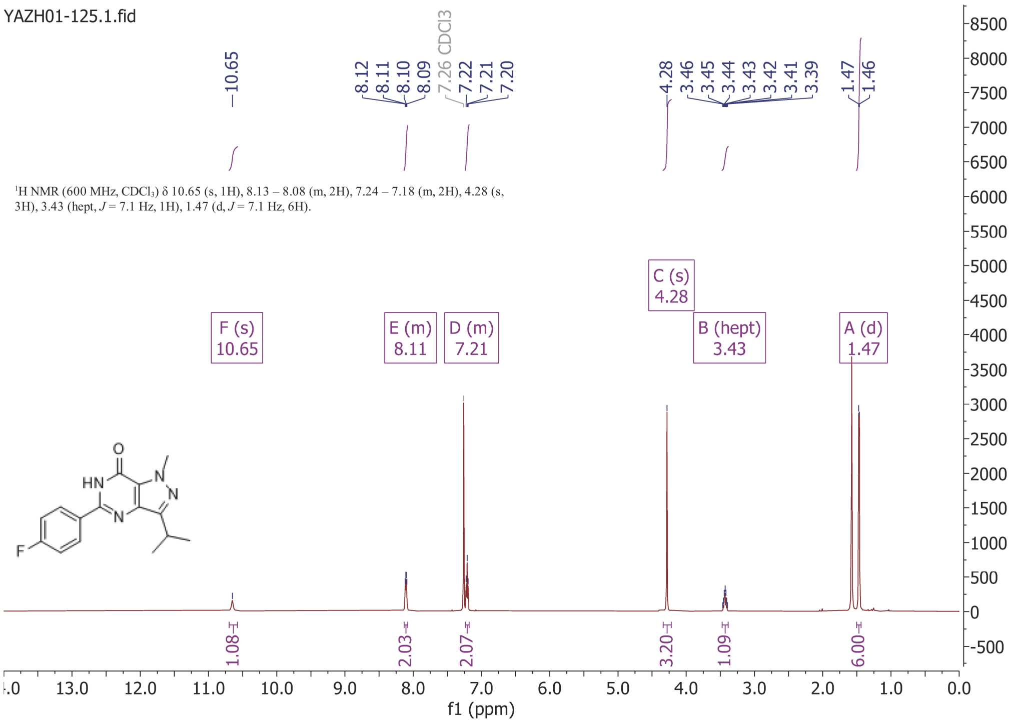

Figure S23. ^{1}H NMR spectrum of compound **11**.

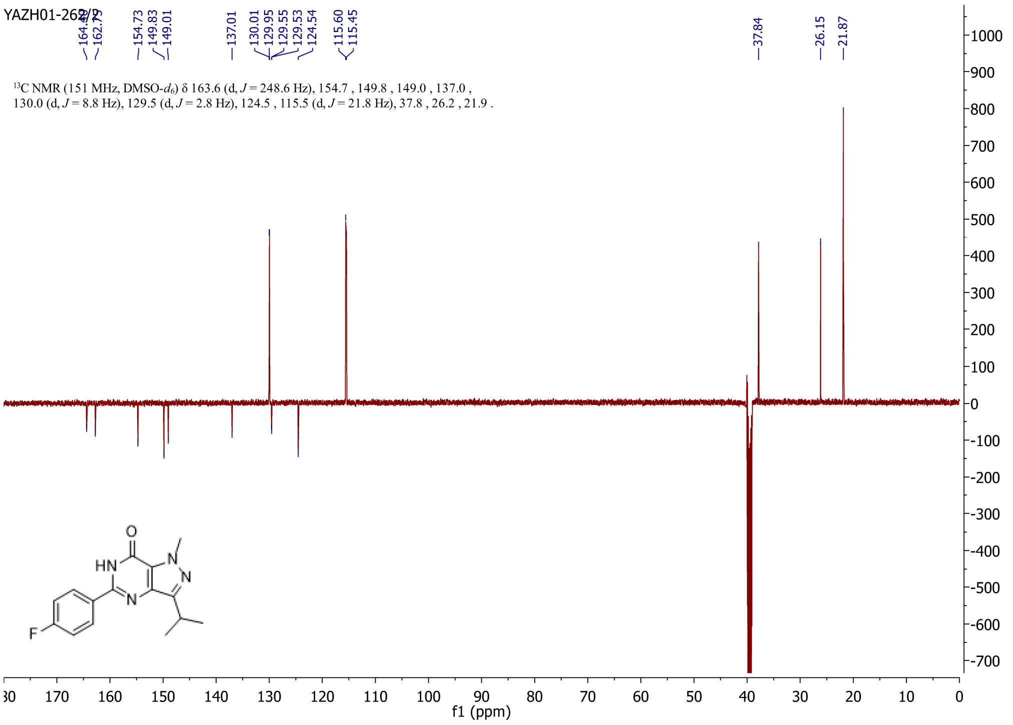

Figure S24. ^{13}C NMR spectrum of compound **11**.

Acquired by	: Admin
Date Acquired	: 13/9/2018 4:48:05 PM
Sample Name	: YAZH01-258
Sample ID	:
Tray#	: 1
Vial#	: 28
Injection Volume	: 1
Data File	: C:\LabSolutions\Data\2018\2018-wk37\YAZH01-258.lcd
Background File	: blanco 13092018.lcd
Method File	: Method SCAN ACID standard MW100.lcm
Report Format	: DefaultLCMS.lcr
Tuning File	: C:\LabSolutions\Tuning\Tuning-ESI-pos-neg01072015a.lct
Processed by	: Admin
Modified Date	: 13/9/2018 5:22:57 PM

Chromatogram
YAZH01-258 C:\LabSolutions\Data\2018\2018-wk37\YAZH01-258.lcd

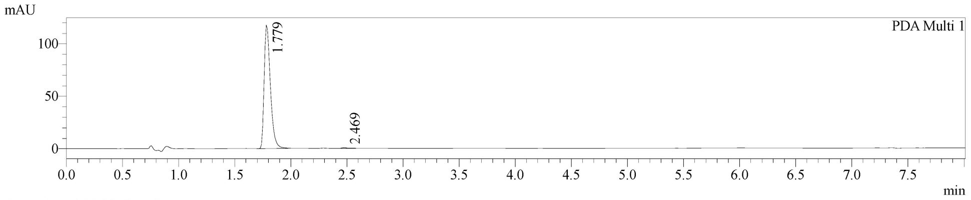

1 PDA Multi 1 / 254nm 4nm

Peak#	Name	Ret. Time	Area	Area %
1		1.779	453050	99.404
2		2.469	2715	0.596

MS Chromatogram
YAZH01-258 C:\LabSolutions\Data\2018\2018-wk37\YAZH01-258.lcd

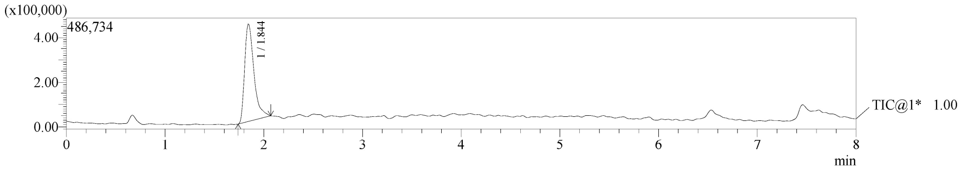

MS Spectrum Graph
#:1 Ret.Time:Averaged 1.830-1.850(Scan#:184-186)
Mass Peaks:10 Base Peak:183.05(279952) Polarity:Pos Segment1 - Event1

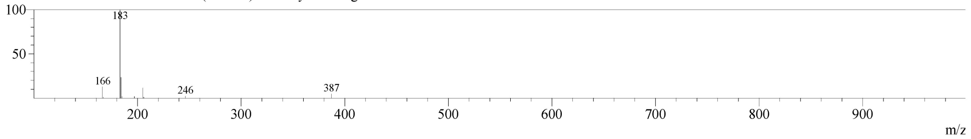

MS Spectrum Table
#:1 Ret.Time:
BG Mode:Calc 1.740<->2.070(175<->208)
Mass Peaks:10 Base Peak:183.05(279952) Polarity:Pos Segment1 - Event1

#	m/z	Abs.Inten.	Rel.Inten.	Charge	Polarity	Monoisotopic	#	m/z	Abs.Inten.	Rel.Inten.	Charge	Polarity	Monoisotopic
1	166.10	35487	12.68				6	197.00	5190	1.85			
2	167.00	3201	1.14				7	205.00	31743	11.34			
3	183.05	279952	100.00				8	206.00	3173	1.13			
4	184.05	65044	23.23				9	246.10	6568	2.35			
5	185.05	5591	2.00				10	387.15	13120	4.69			

Figure S25. LCMS spectrum of compound **14**.

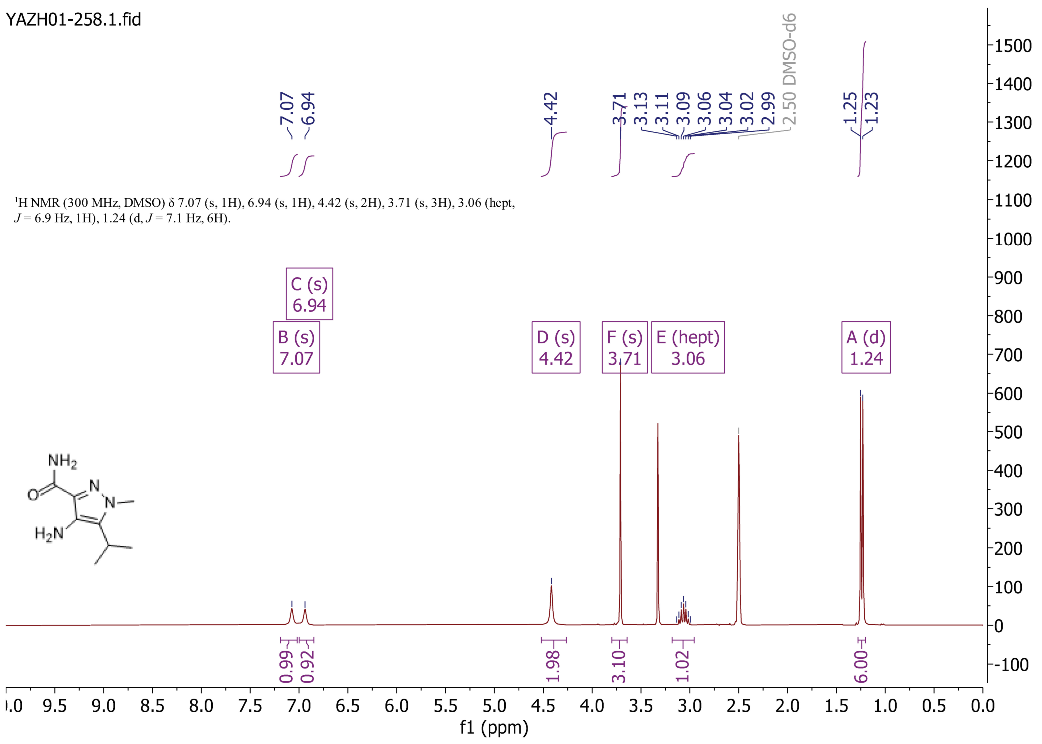

Figure S26. ^{1}H NMR spectrum of compound **14**.

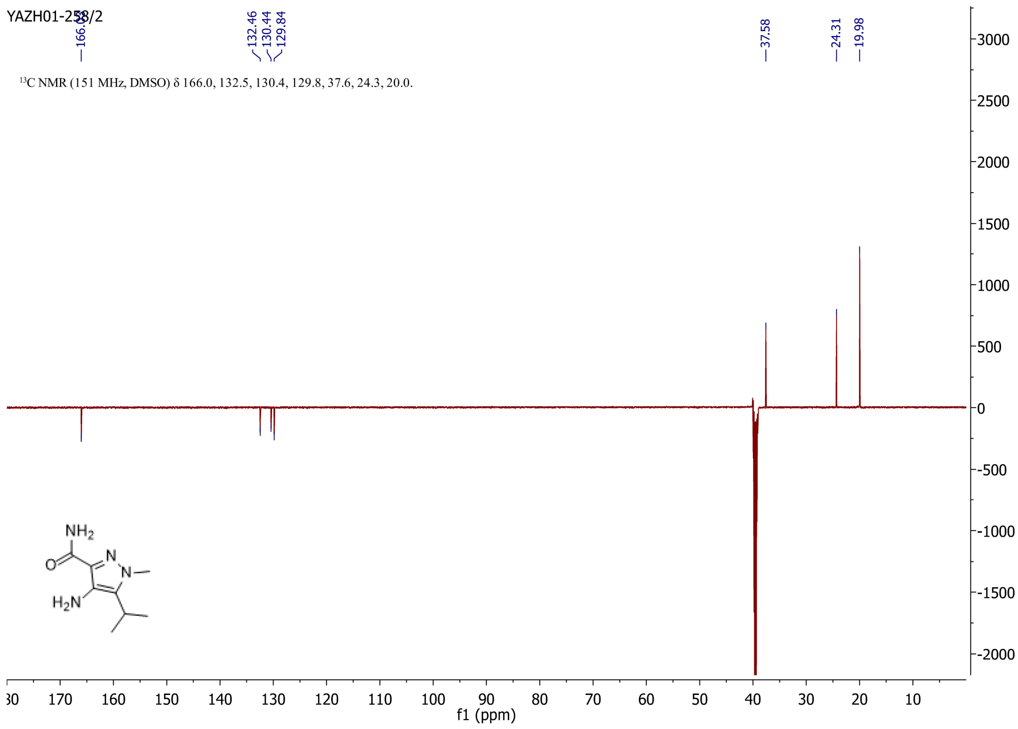

Figure S27. ^{13}C NMR spectrum of compound **14**.

Acquired by	: Admin
Date Acquired	: 15/10/2018 10:05:15 AM
Sample Name	: YAZH01-263
Sample ID	:
Tray#	: 1
Vial#	: 7
Injection Volume	: 3
Data File	: C:\LabSolutions\Data\2018\2018-wk42\YAZH01-263.lcd
Background File	: blanco 15102018.lcd
Method File	: Method SCAN ACID standard.lcm
Report Format	: DefaultLCMS.lcr
Tuning File	: C:\LabSolutions\Tuning\Tuning-ESI-pos-neg01072015a.lct
Processed by	: Admin
Modified Date	: 15/10/2018 10:36:12 AM

Chromatogram
YAZH01-263 C:\LabSolutions\Data\2018\2018-wk42\YAZH01-263.lcd

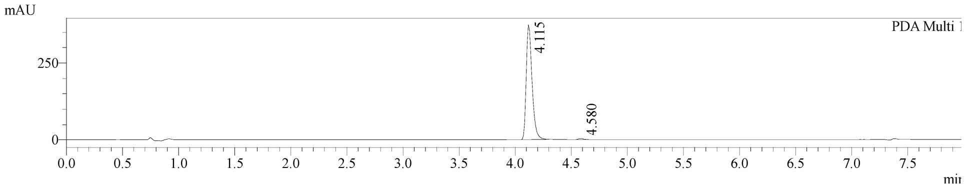

1 PDA Multi 1 / 254nm 4nm

Peak#	Name	Ret. Time	Area	Area %
1		4.115	1342813	99.184
2		4.580	11050	0.816

MS Chromatogram
YAZH01-263 C:\LabSolutions\Data\2018\2018-wk42\YAZH01-263.lcd

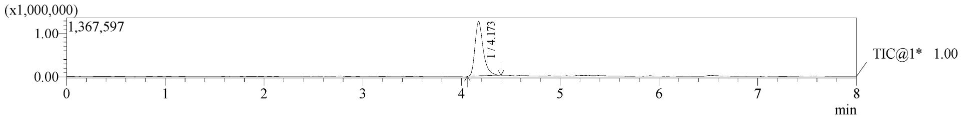

MS Spectrum Graph

#:1 Ret.Time:Averaged 4.160-4.180(Scan#:417-419)
Mass Peaks:11 Base Peak:287.10(814033) Polarity:Pos Segment1 - Event1

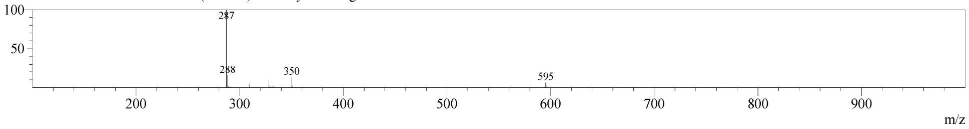

MS Spectrum Table

#:1 Ret.Time:
BG Mode:Calc 4.060<->4.400(407<->441)
Mass Peaks:11 Base Peak:287.10(814033) Polarity:Pos Segment1 - Event1

#	m/z	Abs.Inten.	Rel.Inten.	Charge	Polarity	Monoisotopic
1	287.10	814033	100.00			
2	288.15	127144	15.62			
3	289.10	14865	1.83			
4	309.10	35012	4.30			
5	328.10	73982	9.09			
6	329.15	14796	1.82			
7	332.15	13825	1.70			
8	350.10	106657	13.10			
9	351.10	17400	2.14			
10	595.25	50713	6.23			
11	596.25	23883	2.93			

Figure S28. LCMS spectrum of compound **15**.

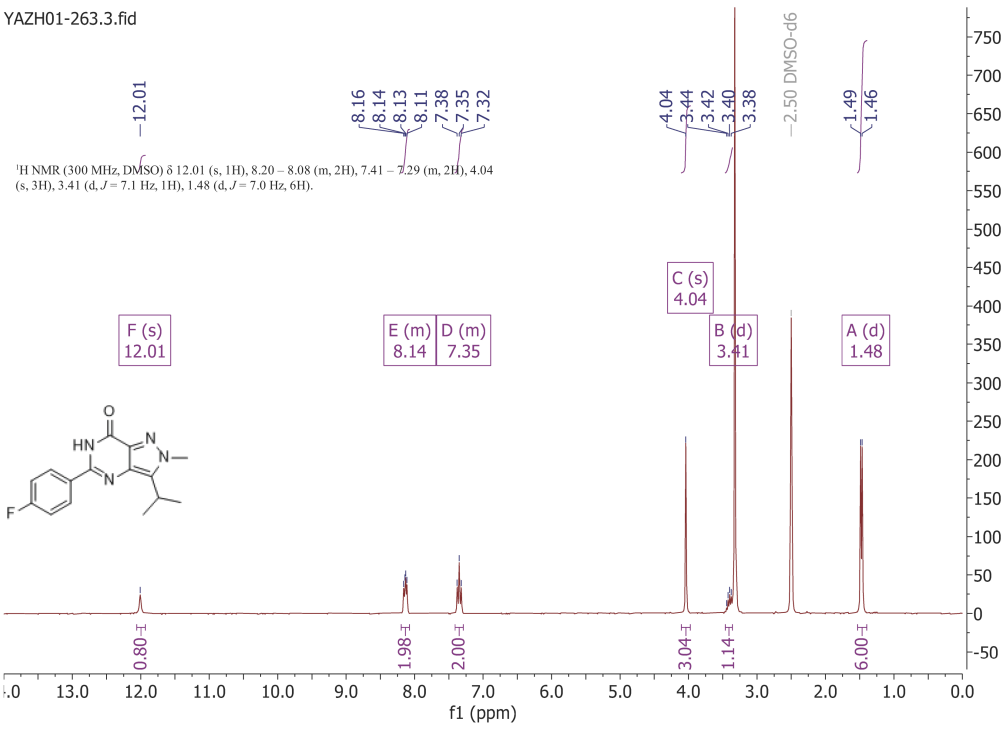

Figure S29. ^{1}H NMR spectrum of compound **15**.

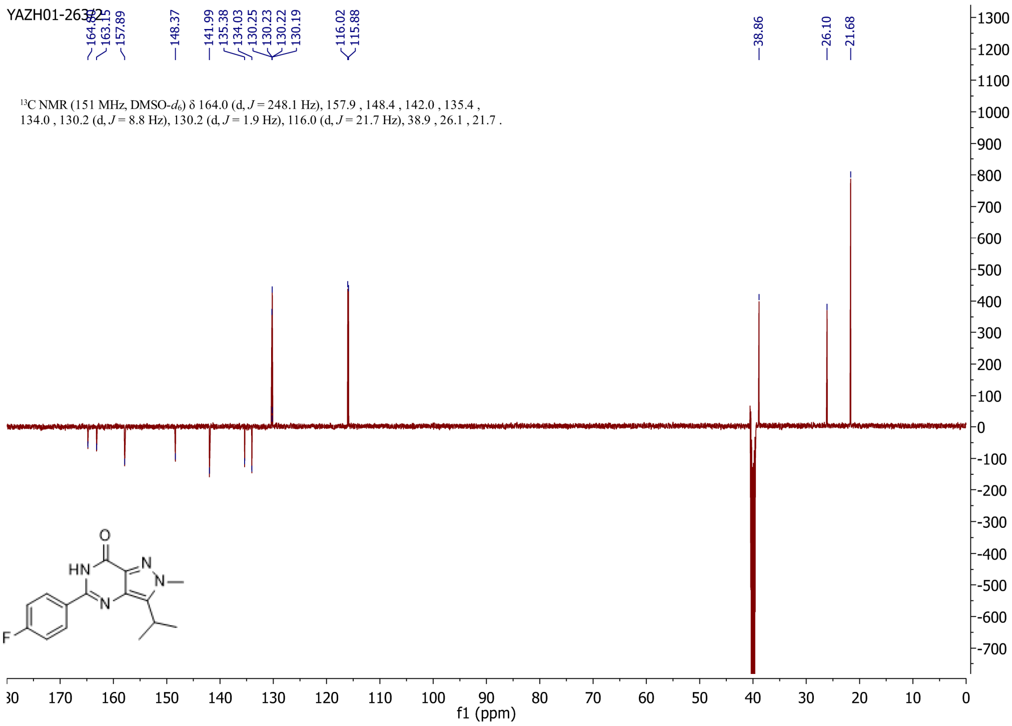

Figure S30. ^{13}C NMR spectrum of compound **15**.

Acquired by	: Admin
Date Acquired	: 17/4/2019 8:59:50 AM
Sample Name	: LABI-044
Sample ID	:
Tray#	: 1
Vial#	: 1
Injection Volume	: 5
Data File	: C:\LabSolutions\Data\2019\2019-wk16\LABI-044.lcd
Background File	: blanco 17042019.lcd
Method File	: Method SCAN ACID standard.lcm
Report Format	: DefaultLCMS.lcr
Tuning File	: C:\LabSolutions\Tuning\Tuning-ESI-pos-neg01072015a.lct
Processed by	: Admin
Modified Date	: 19/6/2019 3:30:24 PM

PDA Graph

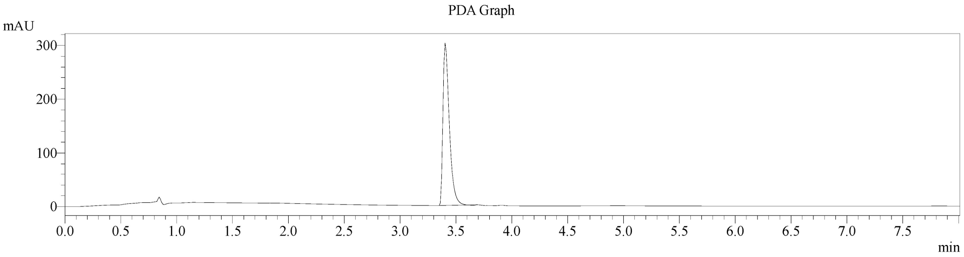

PeakTable

PDA Ch1 254nm 4nm

Peak#	Name	Ret. Time	Area	Area %
1		3.400	1205913	100.000

MS Chromatogram
LABI-044 C:\LabSolutions\Data\2019\2019-wk11-20\2019-wk16\LABI-044.lcd

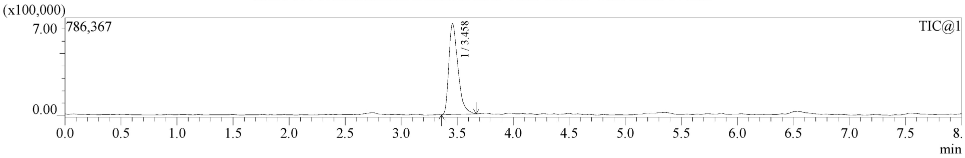

MS Spectrum Graph

#:1 Ret.Time:Averaged 3.450-3.470(Scan#:346-348)
BG Mode:Calc 3.360<->3.670(337<->368)
Mass Peaks:5 Base Peak:272.10(595466) Polarity:Pos Segment1 - Event1

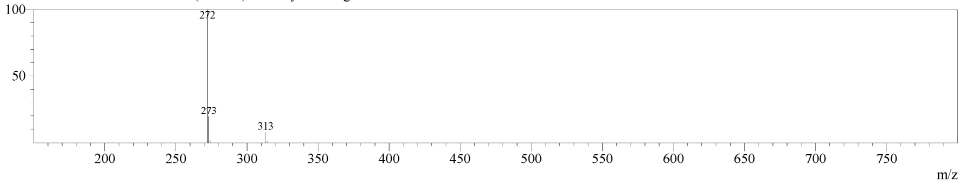

MS Spectrum Table

#:1 Ret.Time:
BG Mode:Calc 3.360<->3.670(337<->368)
Mass Peaks:5 Base Peak:272.10(595466) Polarity:Pos Segment1 - Event1

#	m/z	Abs.Inten.	Rel.Inten.	Charge	Polarity	Monoisotopic
1	272.10	595466	100.00			
2	273.10	116312	19.53			
3	274.10	10013	1.68			
4	313.10	47323	7.95			
5	314.10	8585	1.44			

Figure S31. LCMS spectrum of compound **17**.

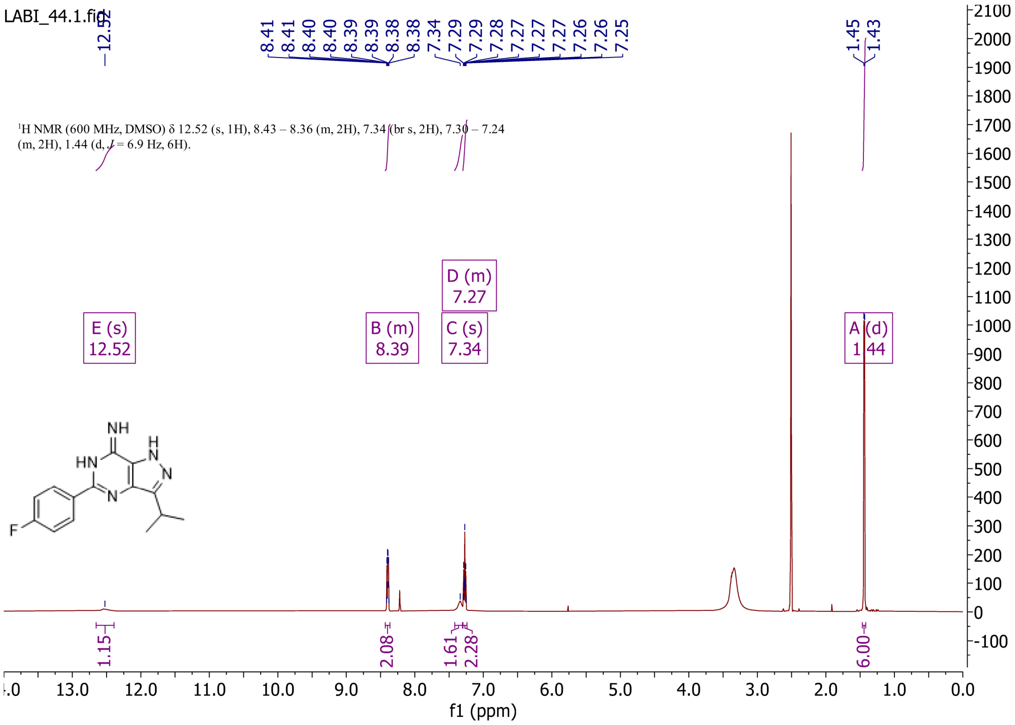

Figure S32. ^{1}H NMR spectrum of compound **17**.

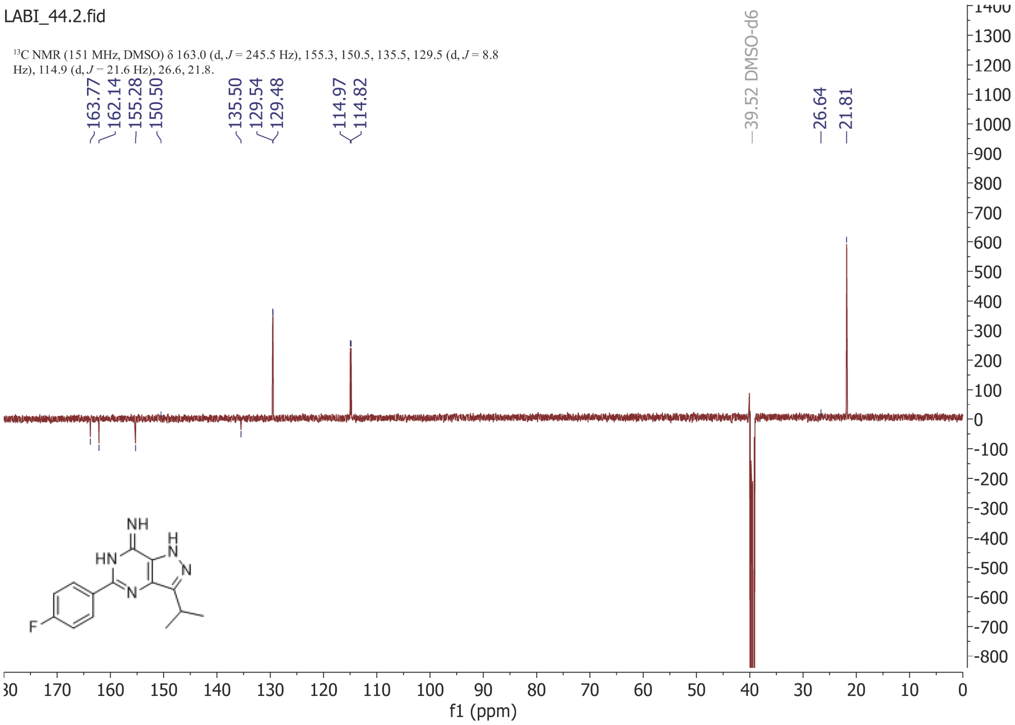

Figure S33. ^{13}C NMR spectrum of compound **17**.

Acquired by : Admin
Date Acquired : 7/3/2019 4:10:39 PM
Sample Name : YAZH_23-1
Sample ID :
Tray# : 1
Vial# : 17
Injection Volume : 4
Data File : C:\LabSolutions\Data\2019\2019-wk09\YAZH_23-1.lcd
Background File : blanco 07032019.lcd
Method File : Method SCAN ACID standard.lcm
Report Format : DefaultLCMS.lcr
Tuning File : C:\LabSolutions\Tuning\Tuning-ESI-pos-neg01072015a.lct
Processed by : Admin
Modified Date : 7/3/2019 4:29:18 PM

PDA Graph

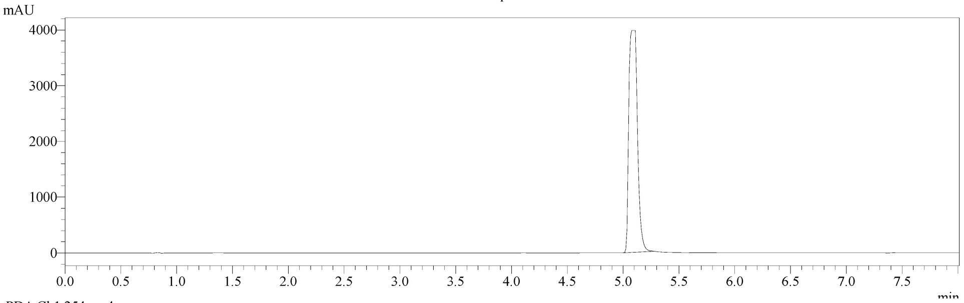

PDA Ch1 254nm 4nm

Peak#	Name	Ret. Time	Area	Area %
1		5.077	21334096	100.000

MS Spectrum Graph

#:1 Ret.Time:Averaged 5.130-5.150(Scan#:514-516)
Mass Peaks:5 Base Peak:256.05(1386430) Polarity:Pos Segment1 - Event1

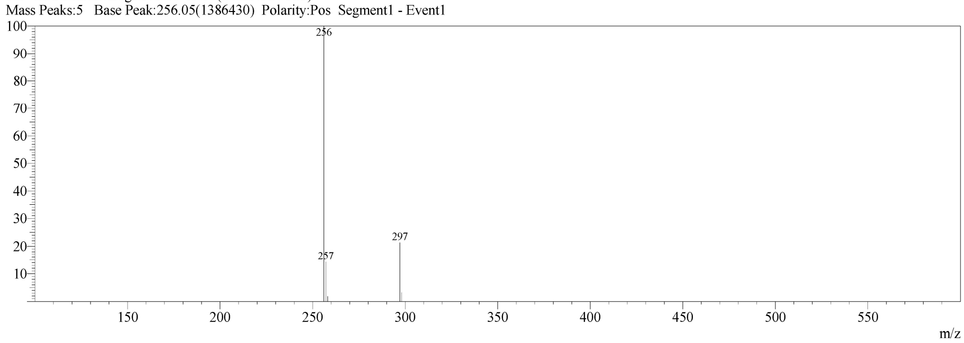

MS Spectrum Table

#:1 Ret.Time:
BG Mode:Calc 5.020<->5.570(503<->558)
Mass Peaks:5 Base Peak:256.05(1386430) Polarity:Pos Segment1 - Event1

#	m/z	Abs.Inten.	Rel.Inten.	Charge	Polarity	Monoisotopic
1	256.05	1386430	100.00			
2	257.10	198226	14.30			
3	258.10	25936	1.87			
4	297.10	296569	21.39			
5	298.10	45252	3.26			

Figure S34. LCMS spectrum of compound **18a**.

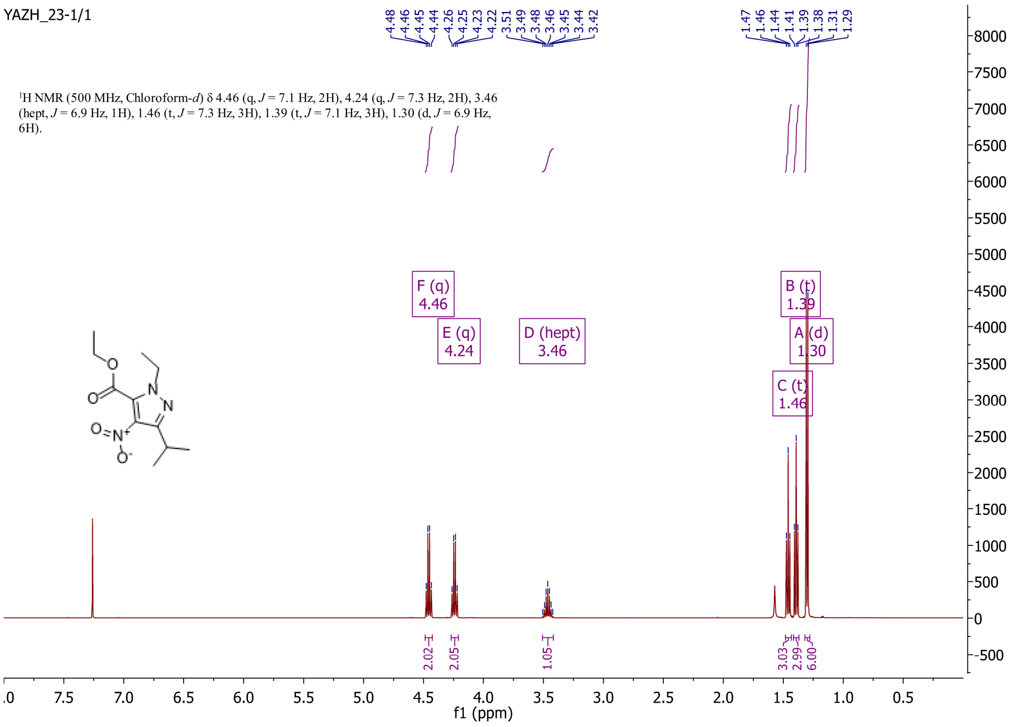

Figure S35. ¹H NMR spectrum of compound **18a**.

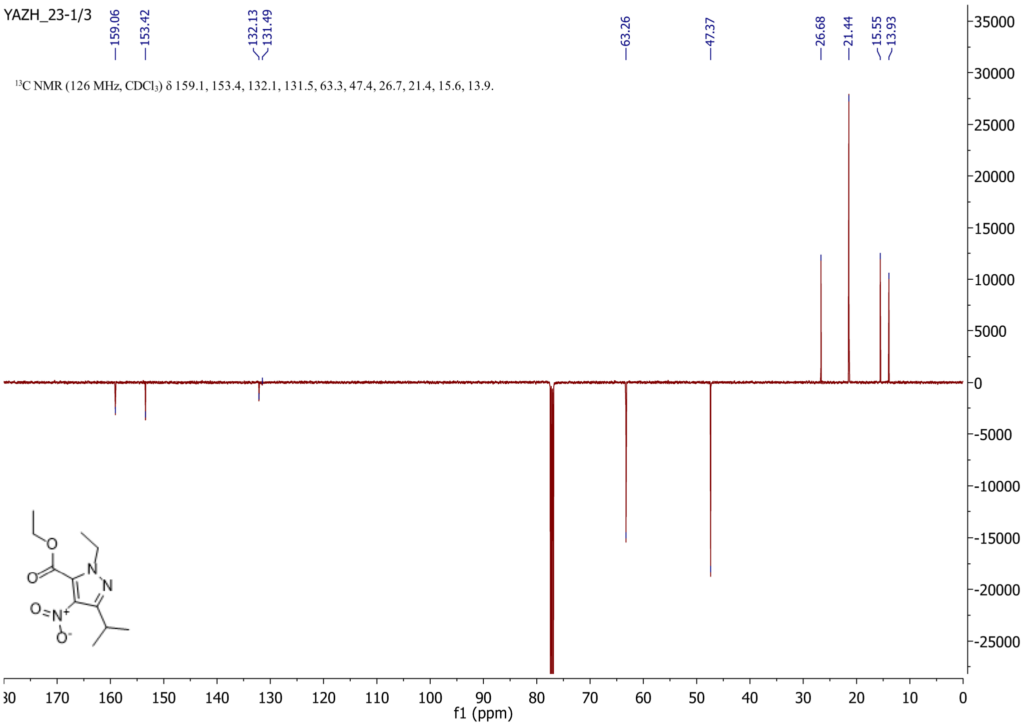

Figure S36. ¹³C NMR spectrum of compound **18a**.

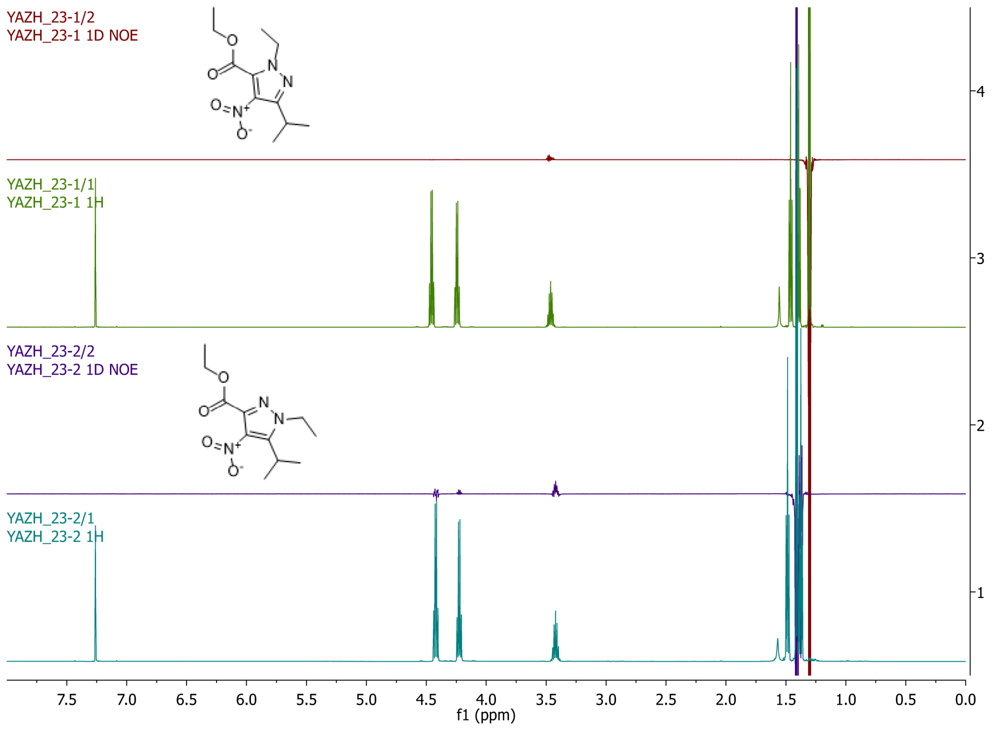

Figure S37. 1D NOESY spectrum of compound **18a** and its regioisomer.

Acquired by : Admin
Date Acquired : 18/6/2019 9:45:35 AM
Sample Name : MOIB_23_1
Sample ID :
Tray# : 1
Vial# : 3
Injection Volume : 1
Data File : C:\LabSolutions\Data\2019\2019-wk25\MOIB_23_1.lcd
Background File : blanco 18062019.lcd
Method File : Method SCAN ACID standard.lcm
Report Format : DefaultLCMS.lcr
Tuning File : C:\LabSolutions\Tuning\Tuning-ESI-pos-neg01072015a.lct
Processed by : Admin
Modified Date : 18/6/2019 10:59:57 AM

PDA Graph

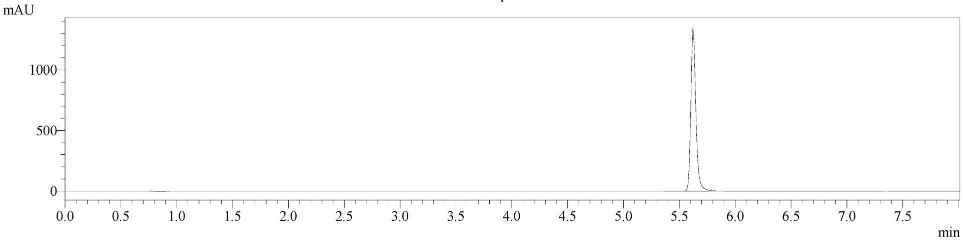

PeakTable

PDA Ch1 254nm 4nm

Peak#	Name	Ret. Time	Area	Area %
1		5.618	4142058	100.000

MS Chromatogram
MOIB_23_1 C:\LabSolutions\Data\2019\2019-wk25\MOIB_23_1.lcd

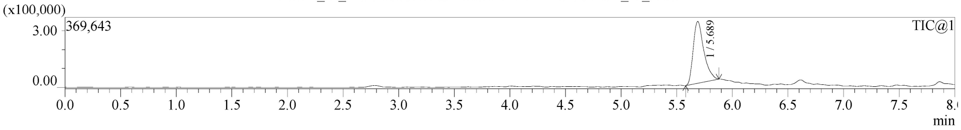

MS Spectrum Graph

#:1 Ret.Time:Averaged 5.680-5.700(Scan#:569-571)
BG Mode:Calc 5.580<->5.880(559<->589)
Mass Peaks:10 Base Peak:284.10(196283) Polarity:Pos Segment1 - Event1

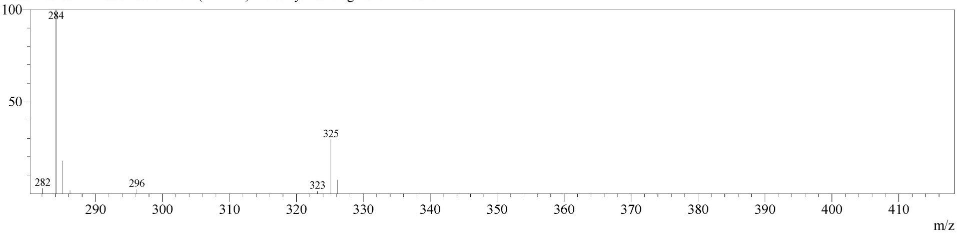

MS Spectrum Table

#:1 Ret.Time:
BG Mode:Calc 5.580<->5.880(559<->589)
Mass Peaks:10 Base Peak:284.10(196283) Polarity:Pos Segment1 - Event1

#	m/z	Abs.Inten.	Rel.Inten.	Charge	Polarity	Monoisotopic
1	280.25	3989	2.03			
2	282.10	5645	2.88			
3	284.10	196283	100.00			
4	285.05	35064	17.86			
5	286.20	3600	1.83			
6	296.15	4447	2.27			
7	323.15	2573	1.31			
8	325.15	57615	29.35			
9	326.10	14474	7.37			
10	418.30	2919	1.49			

Figure S38. LCMS spectrum of compound **18b**.

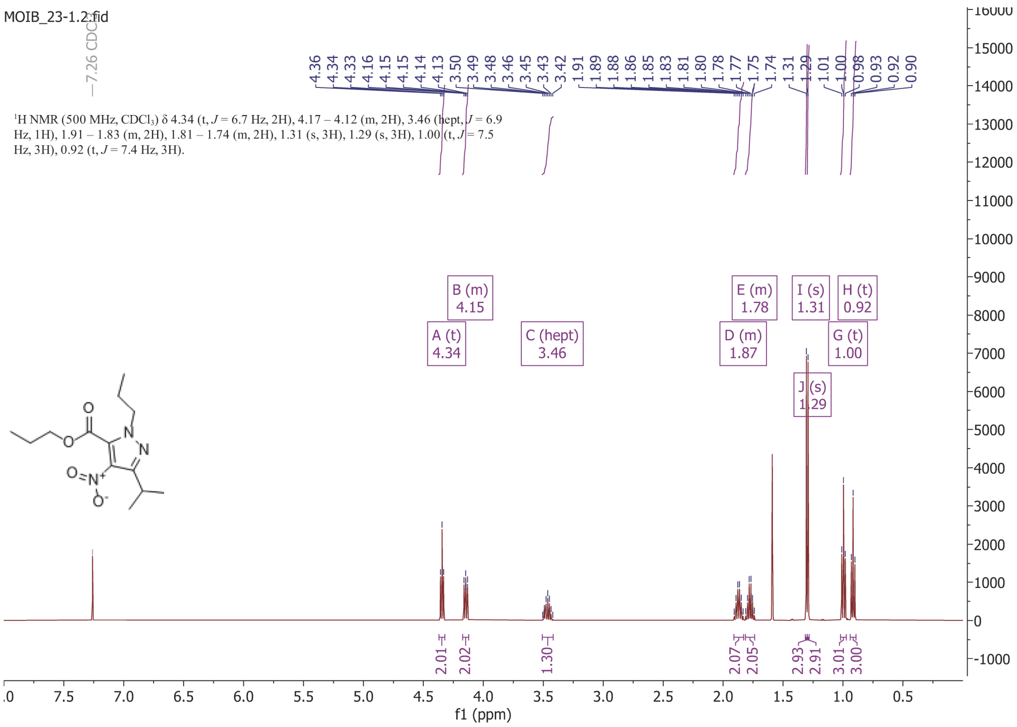

Figure S39. ^{1}H NMR spectrum of compound **18b**.

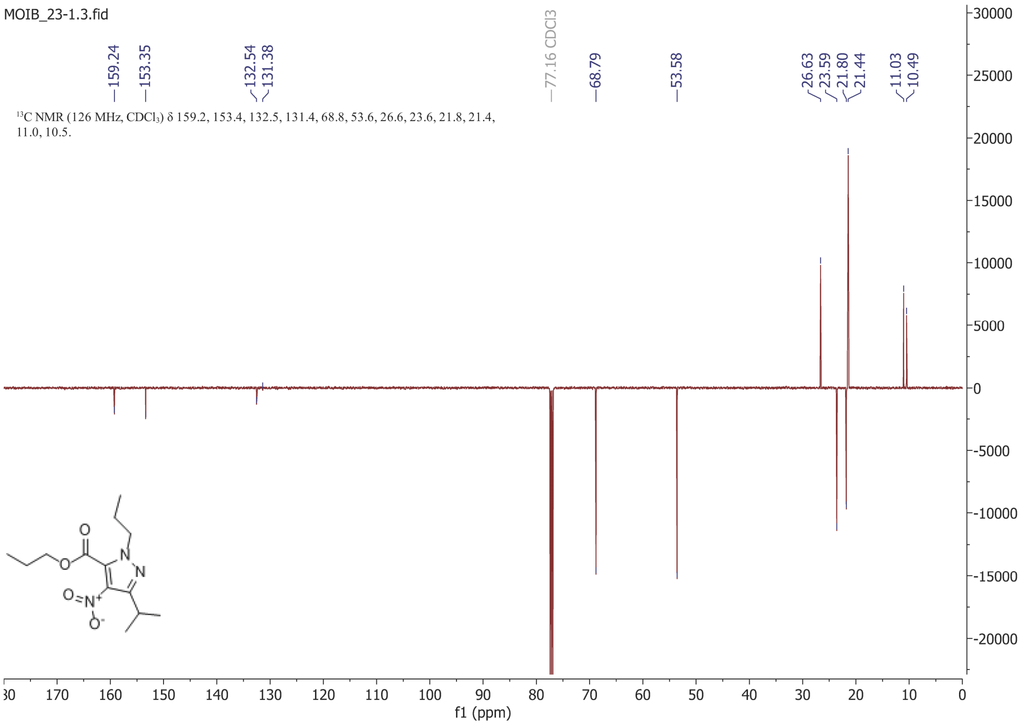

Figure S40. ^{13}C NMR spectrum of compound **18b**.

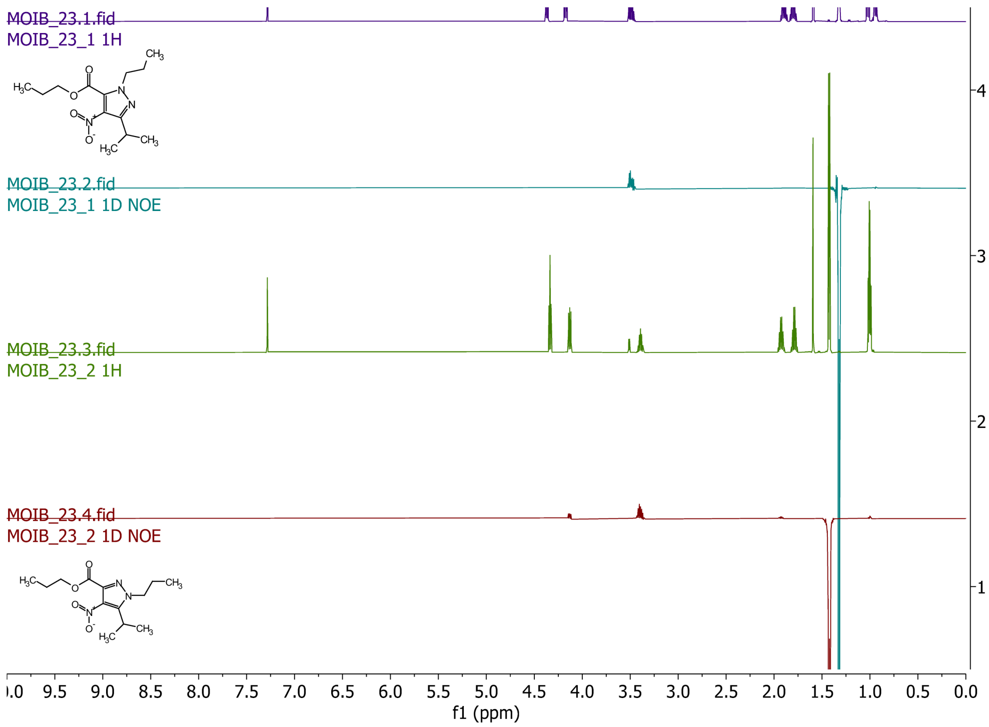

Figure S41. 1D NOESY spectrum of compound **18b** and its regioisomer.

Acquired by	: Admin
Date Acquired	: 19/6/2019 10:34:53 AM
Sample Name	: YAZH_43-1
Sample ID	:
Tray#	: 1
Vial#	: 8
Injection Volume	: 1
Data File	: C:\LabSolutions\Data\2019\2019-wk25\YAZH_43-1.lcd
Background File	: blanco 19062019.lcd
Method File	: Method SCAN ACID standard.lcm
Report Format	: DefaultLCMS.lcr
Tuning File	: C:\LabSolutions\Tuning\Tuning-ESI-pos-neg01072015a.lct
Processed by	: Admin
Modified Date	: 19/6/2019 4:22:46 PM

PDA Graph

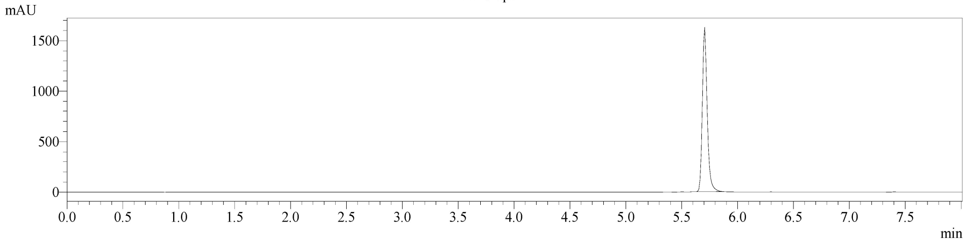

PeakTable

PDA Ch1 254nm 4nm

Peak#	Name	Ret. Time	Area	Area %
1		5.699	4966643	100.000

MS Chromatogram
YAZH_43-1 C:\LabSolutions\Data\2019\2019-wk25\YAZH_43-1.lcd

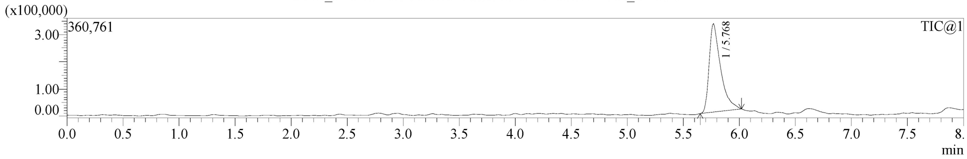

MS Spectrum Graph

#:1 Ret.Time:Averaged 5.760-5.780(Scan#:577-579)
BG Mode:Calc 5.650<->6.020(566<->603)
Mass Peaks:15 Base Peak:284.10(224105) Polarity:Pos Segment1 - Event1

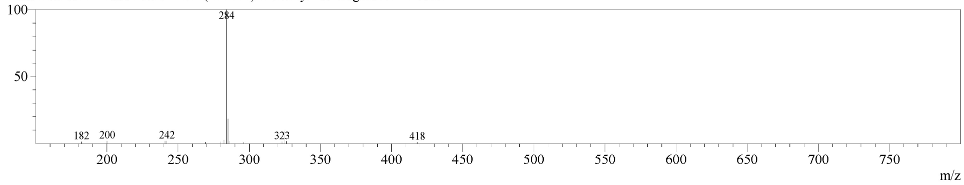

MS Spectrum Table

#:1 Ret.Time:
BG Mode:Calc 5.650<->6.020(566<->603)
Mass Peaks:15 Base Peak:284.10(224105) Polarity:Pos Segment1 - Event1

#	m/z	Abs.Inten.	Rel.Inten.	Charge	Polarity	Monoisotopic
1	181.95	3035	1.35			
2	200.05	4316	1.93			
3	240.90	4325	1.93			
4	242.05	4400	1.96			
5	269.25	2361	1.05			
6	280.15	3036	1.35			
7	282.30	5961	2.66			
8	284.10	224105	100.00			

#	m/z	Abs.Inten.	Rel.Inten.	Charge	Polarity	Monoisotopic
9	285.10	41155	18.36			
10	286.35	3826	1.71			
11	296.20	2329	1.04			
12	323.05	3143	1.40			
13	325.10	21110	9.42			
14	326.15	3795	1.69			
15	418.00	2245	1.00			

Figure S42. LCMS spectrum of compound **18c**.

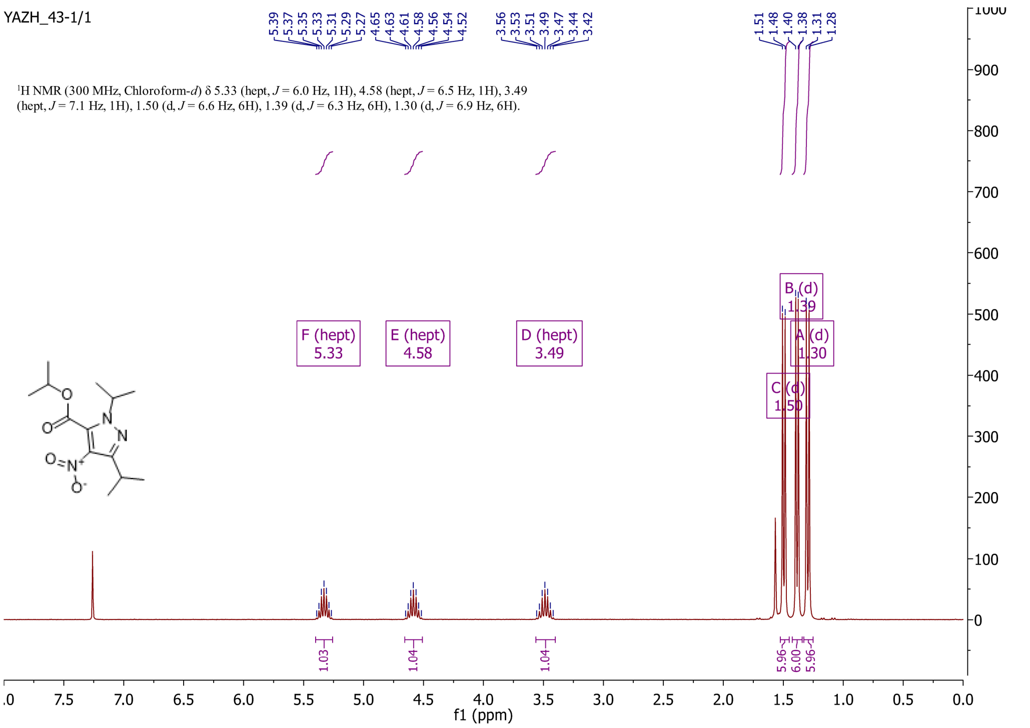

Figure S43. ^{1}H NMR spectrum of compound **18c**.

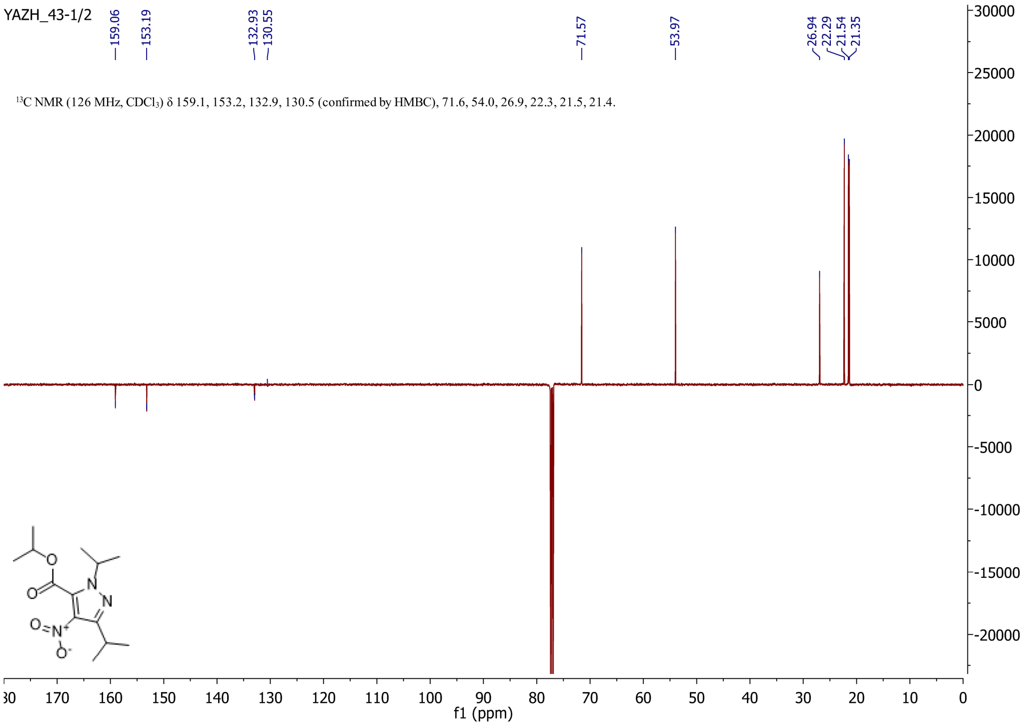

Figure S44. ^{13}C NMR spectrum of compound **18c**.

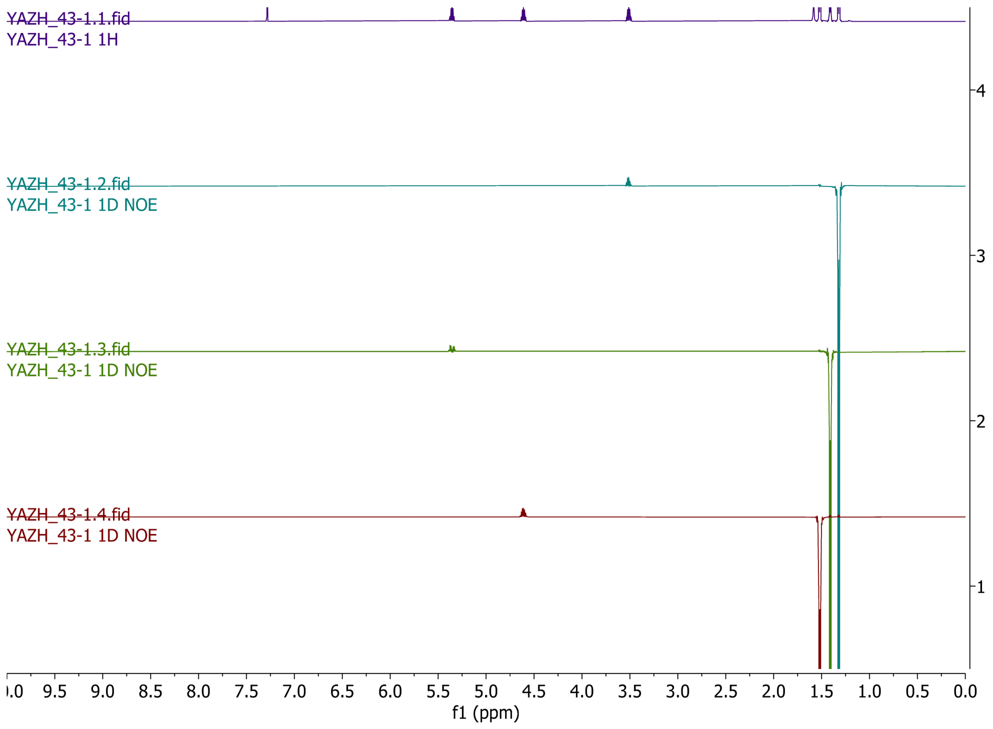

Figure S45. 1D NOESY spectrum of compound **18c**.

Acquired by	: Admin
Date Acquired	: 21/3/2019 12:05:23 PM
Sample Name	: MOIB_1_pure_biggest_peak
Sample ID	:
Tray#	: 1
Vial#	: 9
Injection Volume	: 1
Data File	: C:\LabSolutions\Data\2019\2019-wk11\MOIB_1_pure_biggest_peak.lcd
Background File	: blanco 21032019.lcd
Method File	: Method SCAN ACID standard.lcm
Report Format	: DefaultLCMS.lcr
Tuning File	: C:\LabSolutions\Tuning\Tuning-ESI-pos-neg01072015a.lct
Processed by	: Admin
Modified Date	: 30/3/2021 4:44:59 PM

PDA Graph

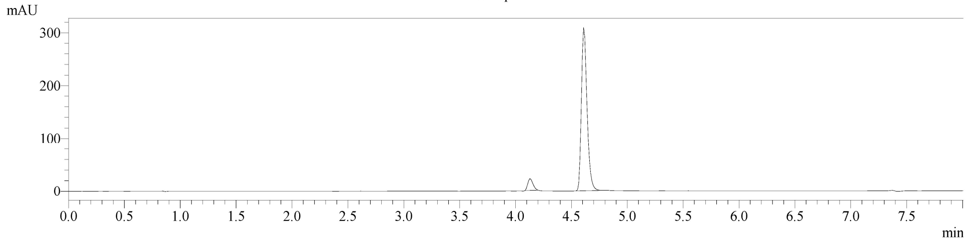

PeakTable

PDA Ch1 254nm 4nm

Peak#	Name	Ret. Time	Area	Area %
1		4.125	71087	6.087
2		4.606	1096754	93.913

MS Chromatogram
MOIB_1_pure_biggest_peak C:\LabSolutions\Data\2019\2019-wk11-20\2019-wk12\MOIB_1_pure_biggest_peak.lcd

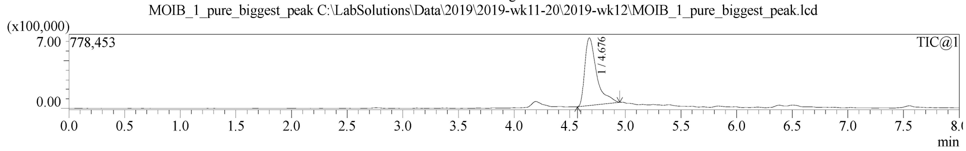

MS Spectrum Graph

#:1 Ret.Time:Averaged 4.670-4.690(Scan#:468-470)
BG Mode:Calc 4.570<->4.950(458<->496)
Mass Peaks:5 Base Peak:316.10(579289) Polarity:Pos Segment1 - Event1

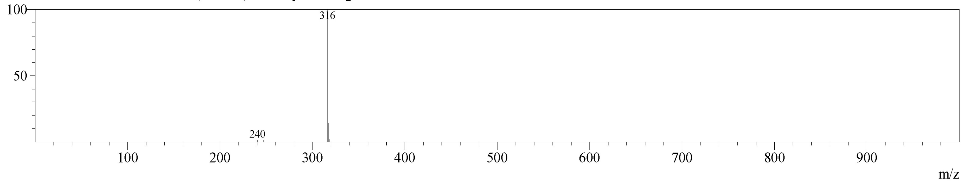

MS Spectrum Table

#:1 Ret.Time:
BG Mode:Calc 4.570<->4.950(458<->496)
Mass Peaks:5 Base Peak:316.10(579289) Polarity:Pos Segment1 - Event1

#	m/z	Abs.Inten.	Rel.Inten.	Charge	Polarity	Monoisotopic
1	240.15	7836	1.35			
2	247.05	8419	1.45			
3	316.10	579289	100.00			
4	317.10	82577	14.25			
5	318.15	11594	2.00			

Figure S46. LCMS spectrum of compound **18d**.

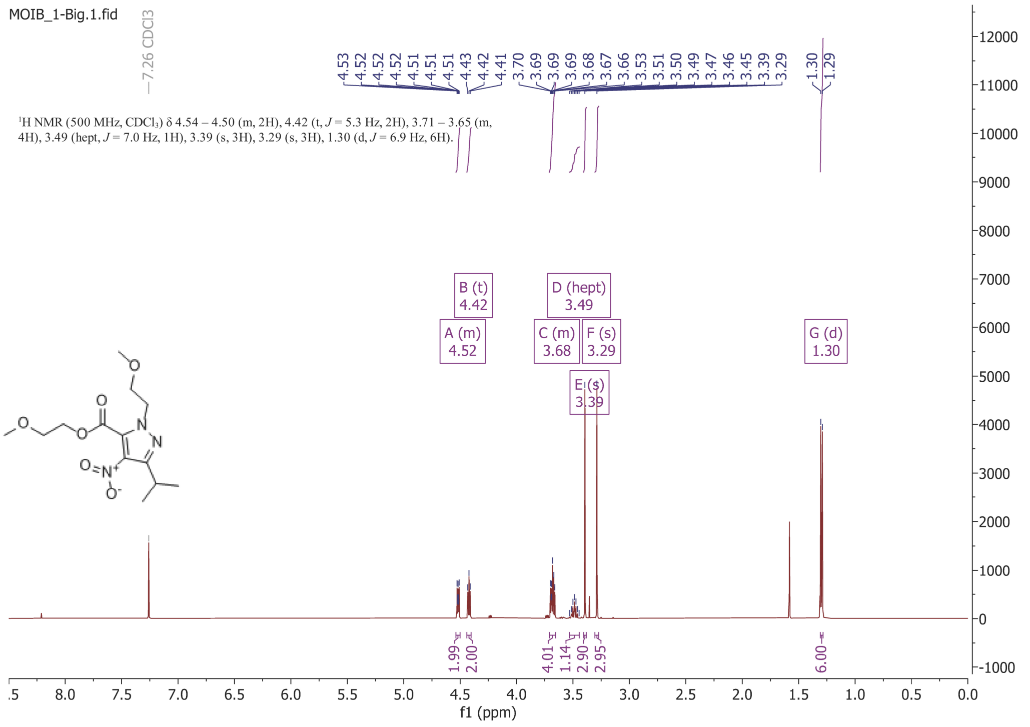

Figure S47. ^{1}H NMR spectrum of compound **18d**.

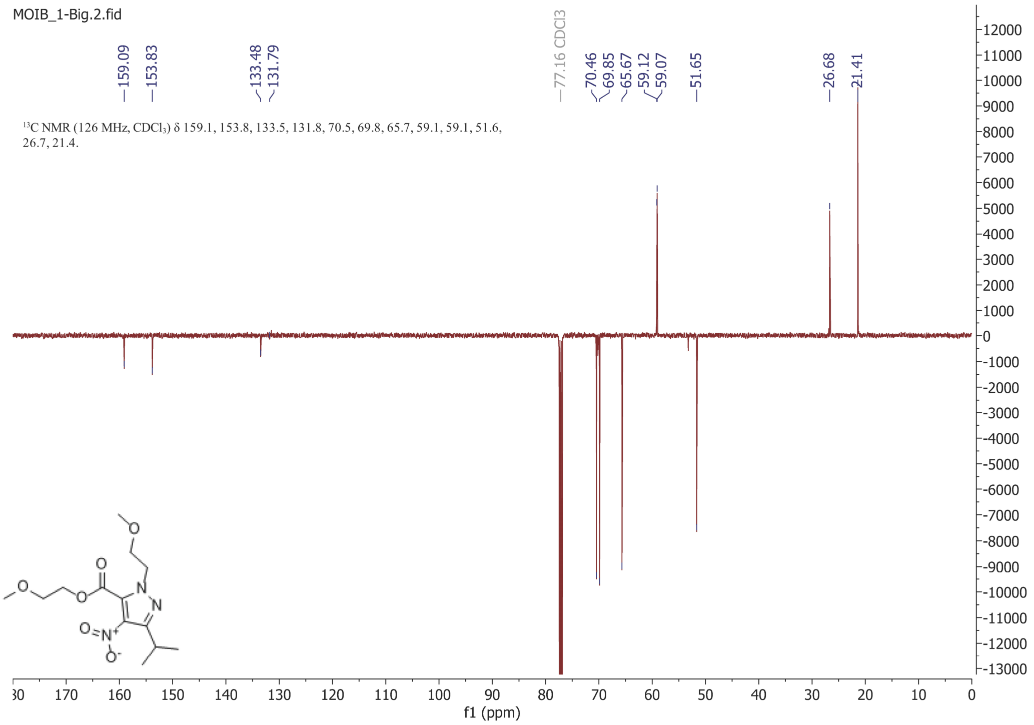

Figure S48. ^{13}C NMR spectrum of compound **18d**.

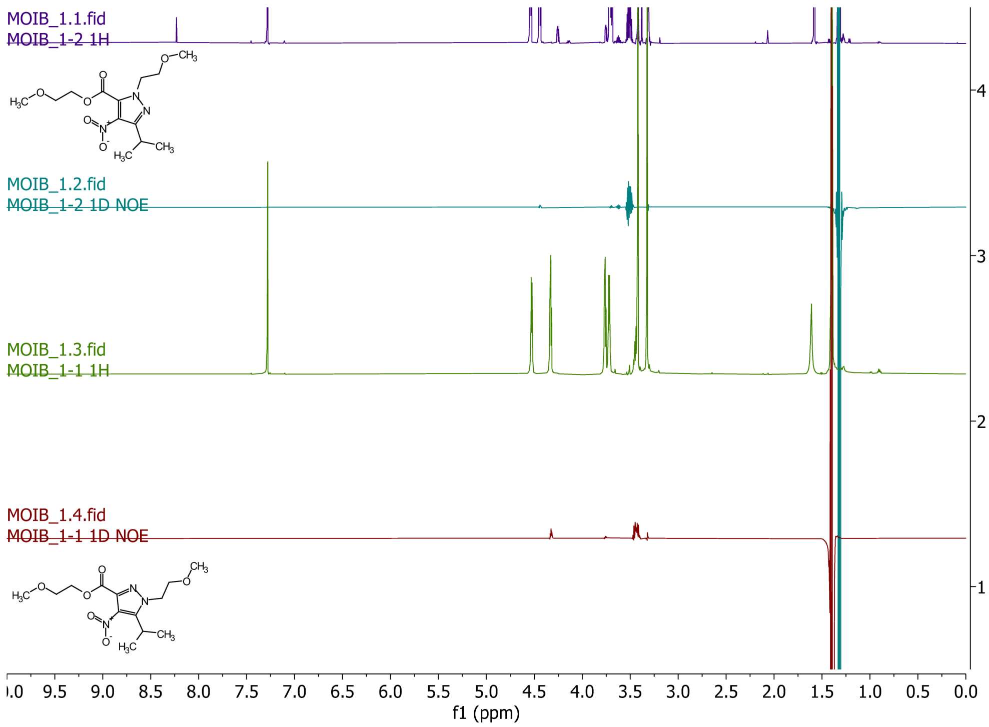

Figure S49. 1D NOESY spectrum of compound **18d** and its regioisomer.

Acquired by	: Admin
Date Acquired	: 26/3/2019 4:14:25 PM
Sample Name	: MOIB_2_pure_big
Sample ID	:
Tray#	: 1
Vial#	: 32
Injection Volume	: 1
Data File	: C:\LabSolutions\Data\2019\2019-wk13\MOIB_2_pure_big.lcd
Background File	: blanco 26032019.lcd
Method File	: Method SCAN ACID standard.lcm
Report Format	: DefaultLCMS.lcr
Tuning File	: C:\LabSolutions\Tuning\Tuning-ESI-pos-neg01072015a.lct
Processed by	: Admin
Modified Date	: 6/4/2021 10:55:03 AM

PDA Graph

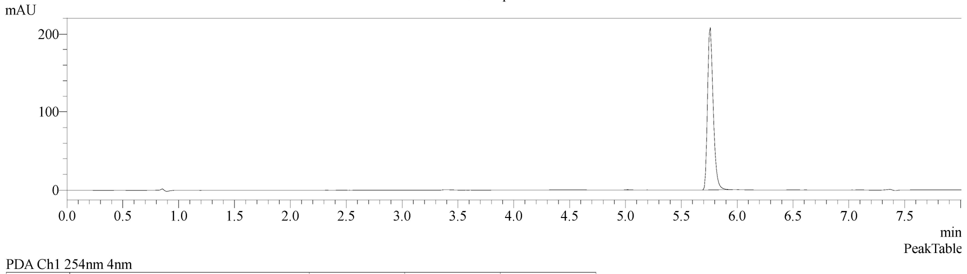

PeakTable

PDA Ch1 254nm 4nm

Peak#	Name	Ret. Time	Area	Area %
1		4.022	714	0.098
2		5.014	1235	0.169
3		5.752	728241	99.733

MS Chromatogram

MOIB_2_pure_big C:\LabSolutions\Data\2019\2019-wk11-20\2019-wk13\MOIB_2_pure_big.lcd

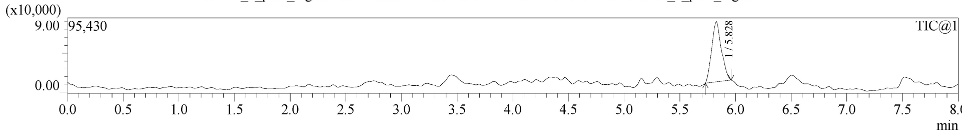

MS Spectrum Graph

#:2 Ret.Time:Averaged 5.820-5.840(Scan#:583-585)

BG Mode:Calc 5.730<->5.960(574<->597)

Mass Peaks:13 Base Peak:468.20(50927) Polarity:Pos Segment1 - Event1

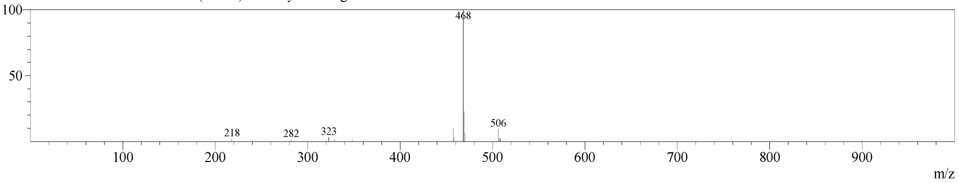

MS Spectrum Table

#:2 Ret.Time:

BG Mode:Calc 5.730<->5.960(574<->597)

Mass Peaks:13 Base Peak:468.20(50927) Polarity:Pos Segment1 - Event1

#	m/z	Abs.Inten.	Rel.Inten.	Charge	Polarity	Monoisotopic
1	217.90	1105	2.17			
2	282.10	845	1.66			
3	322.75	1627	3.19			
4	329.10	873	1.71			
5	348.05	675	1.33			
6	457.40	5176	10.16			
7	458.25	1603	3.15			

#	m/z	Abs.Inten.	Rel.Inten.	Charge	Polarity	Monoisotopic
8	468.20	50927	100.00			
9	469.15	11685	22.94			
10	470.20	3443	6.76			
11	506.25	4792	9.41			
12	507.25	1404	2.76			
13	508.45	1305	2.56			

Figure S50. LCMS spectrum of compound **18e**.

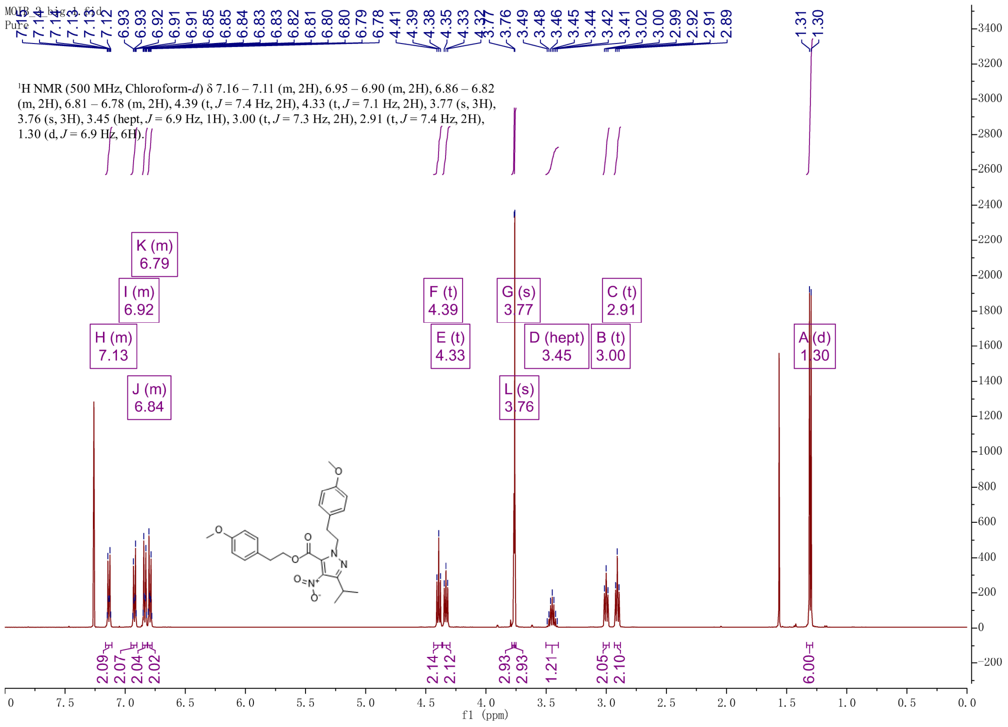

Figure S51. ^{1}H NMR spectrum of compound **18e**.

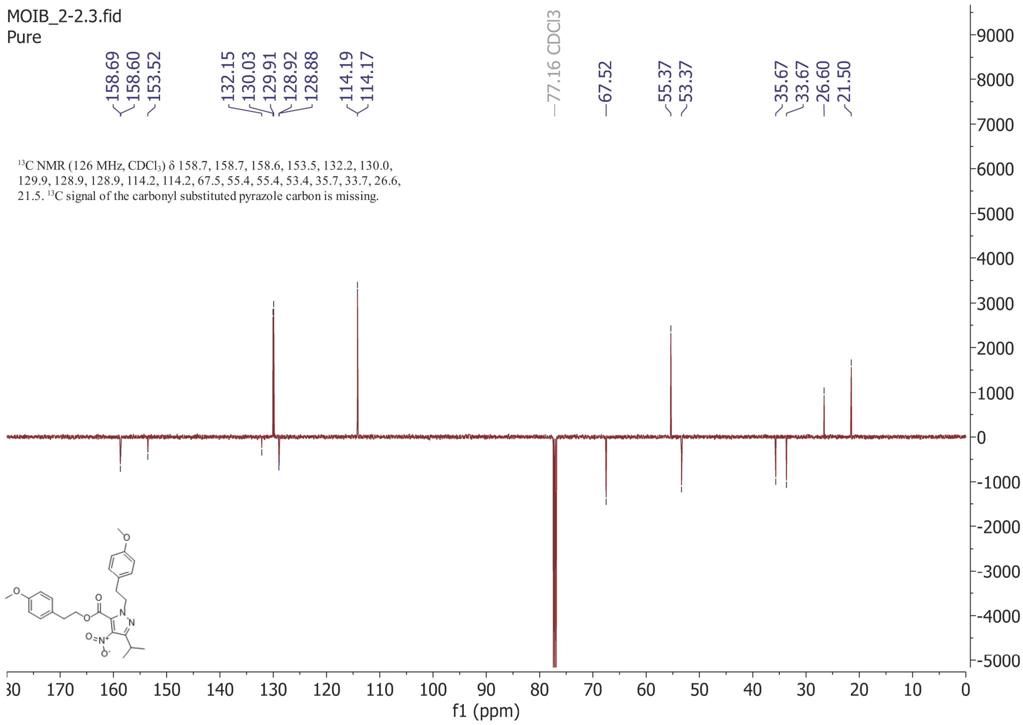

Figure S52. ^{13}C NMR spectrum of compound **18e**.

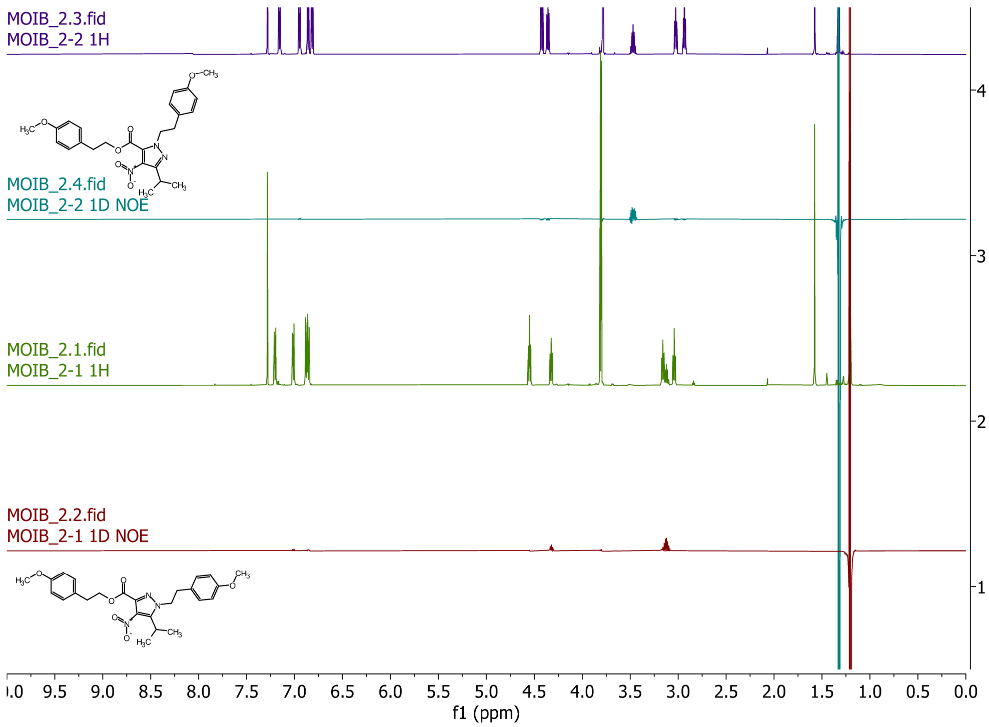

Figure S53. 1D NOESY spectrum of compound **18e** and its regioisomer.

Acquired by	: Admin
Date Acquired	: 2/7/2019 11:38:22 AM
Sample Name	: YAZH_44
Sample ID	:
Tray#	: 1
Vial#	: 3
Injection Volume	: 3
Data File	: C:\LabSolutions\Data\2019\2019-wk27\YAZH_44-20190702.lcd
Background File	: 19.lcd
Method File	: Method SCAN ACID standard.lcm
Report Format	: DefaultLCMS.lcr
Tuning File	: C:\LabSolutions\Tuning\Tuning-ESI-pos-neg01072015a.lct
Processed by	: Admin
Modified Date	: 2/7/2019 12:25:47 PM

PDA Graph

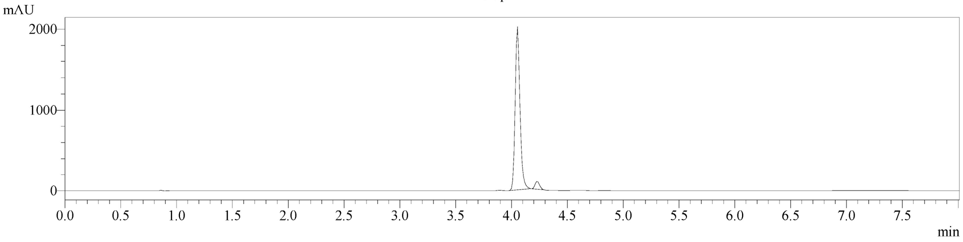

PeakTable

PDA Ch1 254nm 4nm

Peak#	Name	Ret. Time	Area	Area %
1		4.047	6035513	95.733
2		4.223	269043	4.267

MS Chromatogram
YAZH_44 C:\LabSolutions\Data\2019\2019-wk27\YAZH_44-20190702.lcd

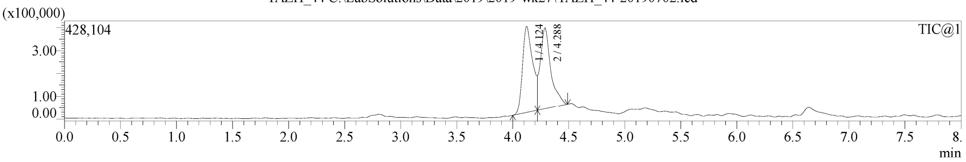

MS Spectrum Graph

#:1 Ret.Time:Averaged 4.110-4.130(Scan#:412-414)
BG Mode:Calc 4.000<->4.220(401<->423)
Mass Peaks:10 Base Peak:241.05(208535) Polarity:Pos Segment1 - Event1

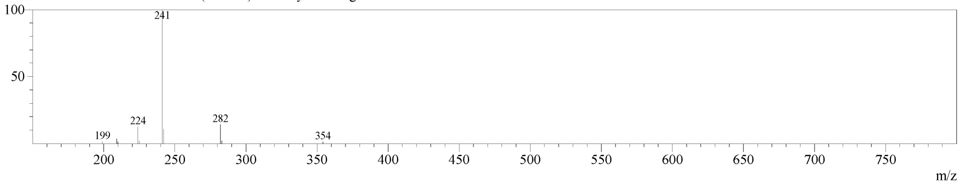

MS Spectrum Table

#:1 Ret.Time:
BG Mode:Calc 4.000<->4.220(401<->423)
Mass Peaks:10 Base Peak:241.05(208535) Polarity:Pos Segment1 - Event1

#	m/z	Abs.Inten.	Rel.Inten.	Charge	Polarity	Monoisotopic	#	m/z	Abs.Inten.	Rel.Inten.	Charge	Polarity	Monoisotopic
1	198.90	3110	1.49				6	241.05	208535	100.00			
2	209.05	7479	3.59				7	242.00	22572	10.82			
3	209.95	2828	1.36				8	282.05	29765	14.27			
4	224.00	26262	12.59				9	283.10	4444	2.13			
5	225.05	4312	2.07				10	354.10	2765	1.33			

Figure S54. LCMS spectrum of compound **19c**.

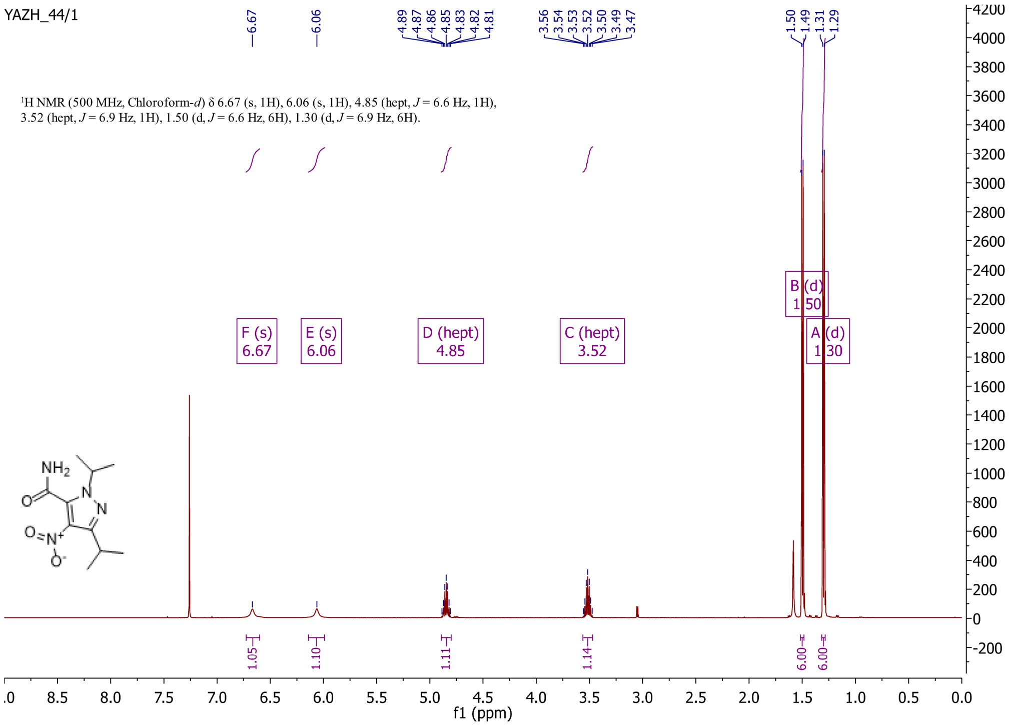

Figure S55. ^{1}H NMR spectrum of compound **19c**.

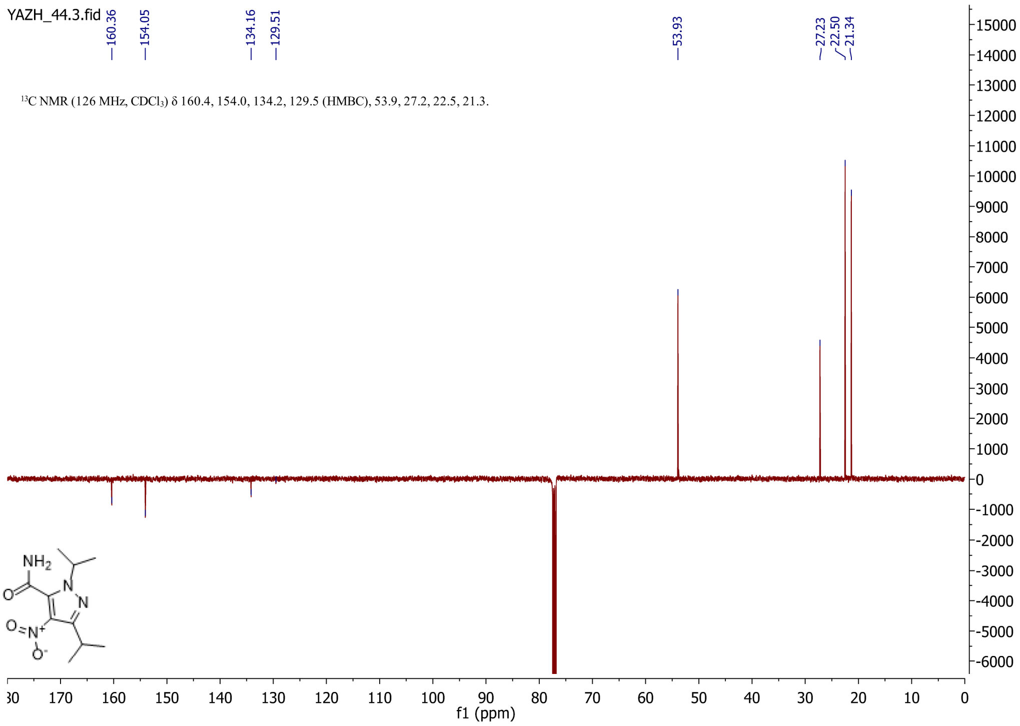

Figure S56. ^{13}C NMR spectrum of compound **19c**.

Acquired by	: Admin
Date Acquired	: 1/5/2019 4:50:28 PM
Sample Name	: MOIB_9-6
Sample ID	:
Tray#	: 1
Vial#	: 54
Injection Volume	: 1
Data File	: C:\LabSolutions\Data\2019\2019-wk18\MOIB_9-6.lcd
Background File	: blanco 01052019.lcd
Method File	: Method SCAN ACID standard.lcm
Report Format	: DefaultLCMS.lcr
Tuning File	: C:\LabSolutions\Tuning\Tuning-ESI-pos-neg01072015a.lct
Processed by	: Admin
Modified Date	: 6/4/2021 10:57:17 AM

PDA Graph

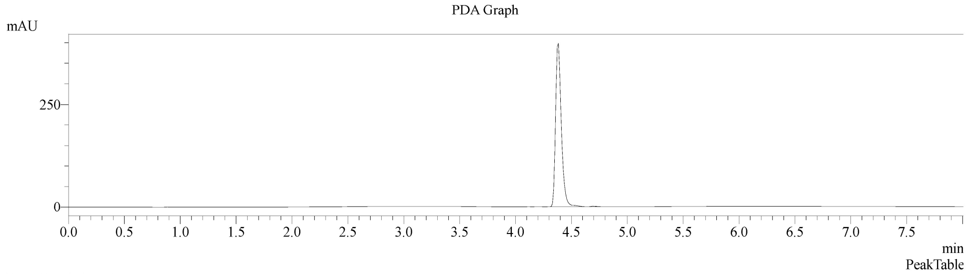

PeakTable

PDA Ch1 254nm 4nm

Peak#	Name	Ret. Time	Area	Area %
1		4.374	1419030	99.676
2		4.691	4610	0.324

MS Chromatogram
MOIB_9-6 C:\LabSolutions\Data\2019\2019-wk11-20\2019-wk18\MOIB_9-6.lcd

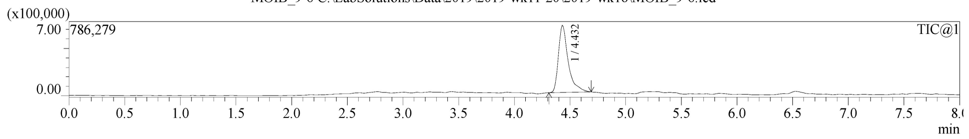

MS Spectrum Graph

#:1 Ret.Time:Averaged 4.420-4.440(Scan#:443-445)
BG Mode:Calc 4.310<->4.690(432<->470)
Mass Peaks:8 Base Peak:333.10(505858) Polarity:Pos Segment1 - Event1

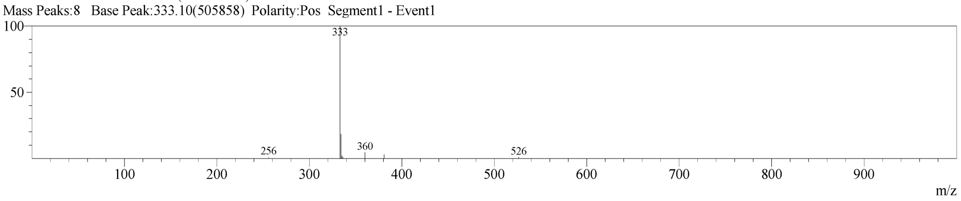

MS Spectrum Table

#:1 Ret.Time:
BG Mode:Calc 4.310<->4.690(432<->470)
Mass Peaks:8 Base Peak:333.10(505858) Polarity:Pos Segment1 - Event1

#	m/z	Abs.Inten.	Rel.Inten.	Charge	Polarity	Monoisotopic	#	m/z	Abs.Inten.	Rel.Inten.	Charge	Polarity	Monoisotopic
1	255.90	6300	1.25				5	336.20	5295	1.05			
2	333.10	505858	100.00				6	360.10	23540	4.65			
3	334.15	92961	18.38				7	380.85	15211	3.01			
4	335.15	11248	2.22				8	526.30	5702	1.13			

Figure S57. LCMS spectrum of compound **19e**.

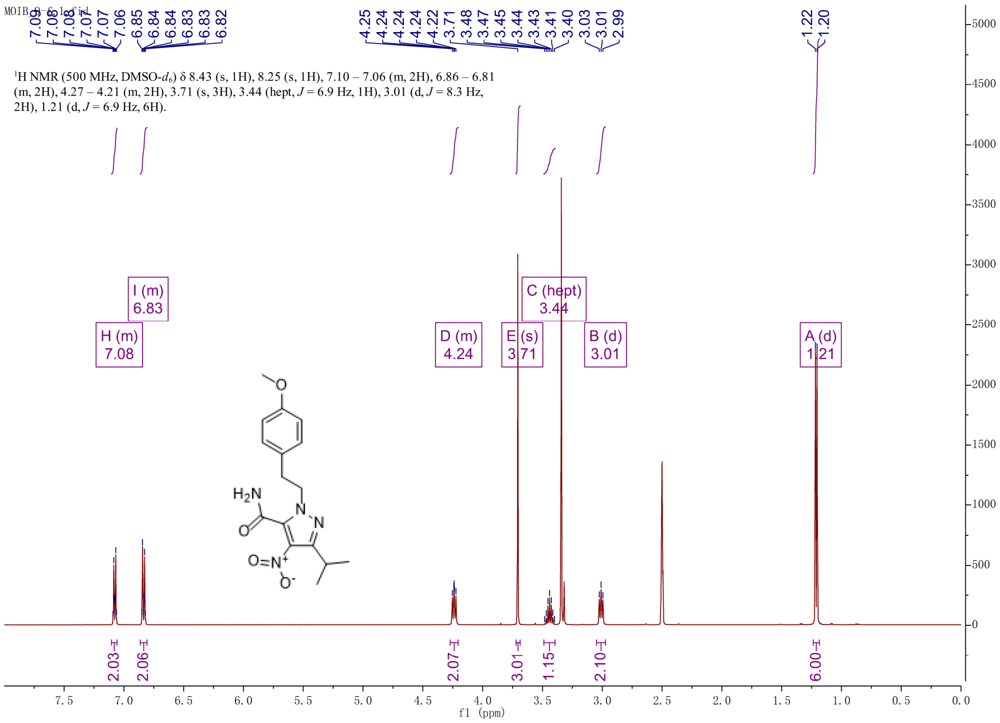

Figure S58. ^{1}H NMR spectrum of compound **19e**.

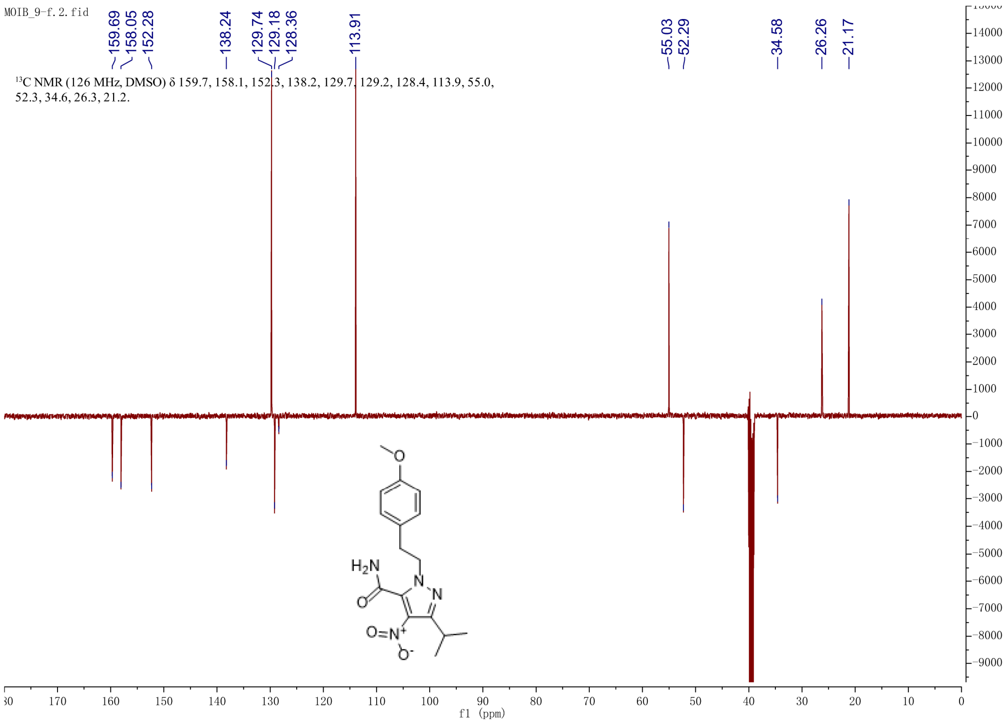

Figure S59. ^{13}C NMR spectrum of compound **19e**.

Acquired by : Admin
Date Acquired : 26/6/2019 10:12:06 AM
Sample Name : MOIB_20_pure
Sample ID :
Tray# : 1
Vial# : 3
Injection Volume : 1
Data File : C:\LabSolutions\Data\2019\2019-wk26\MOIB_20_pure.lcd
Background File : Blanco 26062019.lcd
Method File : Method SCAN ACID standard.lcm
Report Format : DefaultLCMS.lcr
Tuning File : C:\LabSolutions\Tuning\Tuning-ESI-pos-neg01072015a.lct
Processed by : Admin
Modified Date : 26/6/2019 10:41:04 AM

PDA Graph

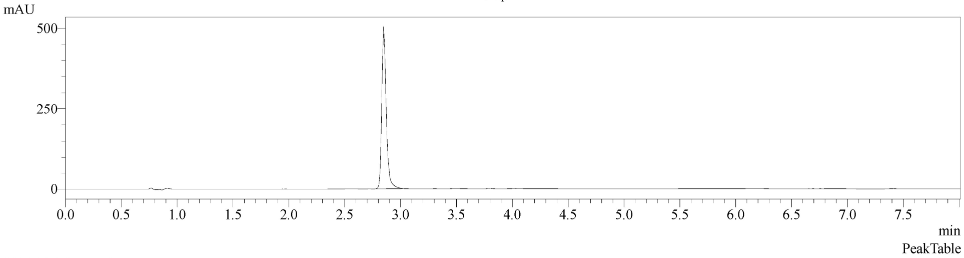

PeakTable

PDA Ch1 254nm 4nm

Peak#	Name	Ret. Time	Area	Area %
1		2.844	1429628	100.000

MS Chromatogram
MOIB_20_pure C:\LabSolutions\Data\2019\2019-wk26\MOIB_20_pure.lcd

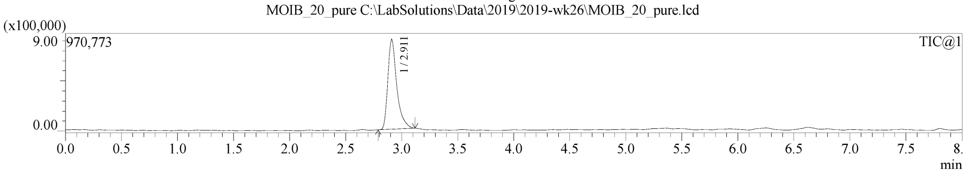

MS Spectrum Graph

#:1 Ret.Time:Averaged 2.900-2.920(Scan#:291-293)
BG Mode:Calc 2.790<->3.120(280<->313)
Mass Peaks:14 Base Peak:270.05(544210) Polarity:Pos Segment1 - Event1

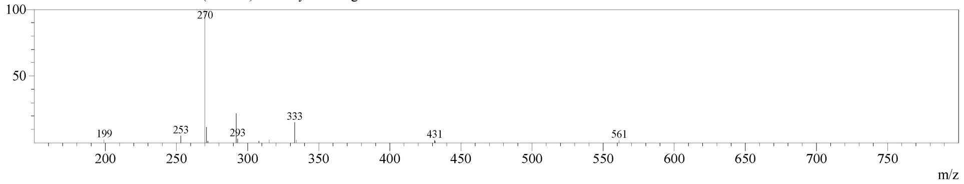

MS Spectrum Table

#:1 Ret.Time:
BG Mode:Calc 2.790<->3.120(280<->313)
Mass Peaks:14 Base Peak:270.05(544210) Polarity:Pos Segment1 - Event1

#	m/z	Abs.Inten.	Rel.Inten.	Charge	Polarity	Monoisotopic	#	m/z	Abs.Inten.	Rel.Inten.	Charge	Polarity	Monoisotopic
1	199.05	12940	2.38				8	307.95	8087	1.49			
2	253.05	28192	5.18				9	315.10	12831	2.36			
3	270.05	544210	100.00				10	333.10	83124	15.27			
4	271.10	63109	11.60				11	334.15	11363	2.09			
5	272.10	8015	1.47				12	431.50	10826	1.99			
6	292.05	119659	21.99				13	431.85	7007	1.29			
7	293.05	16508	3.03				14	561.25	10716	1.97			

Figure S60. LCMS spectrum of compound **19f**.

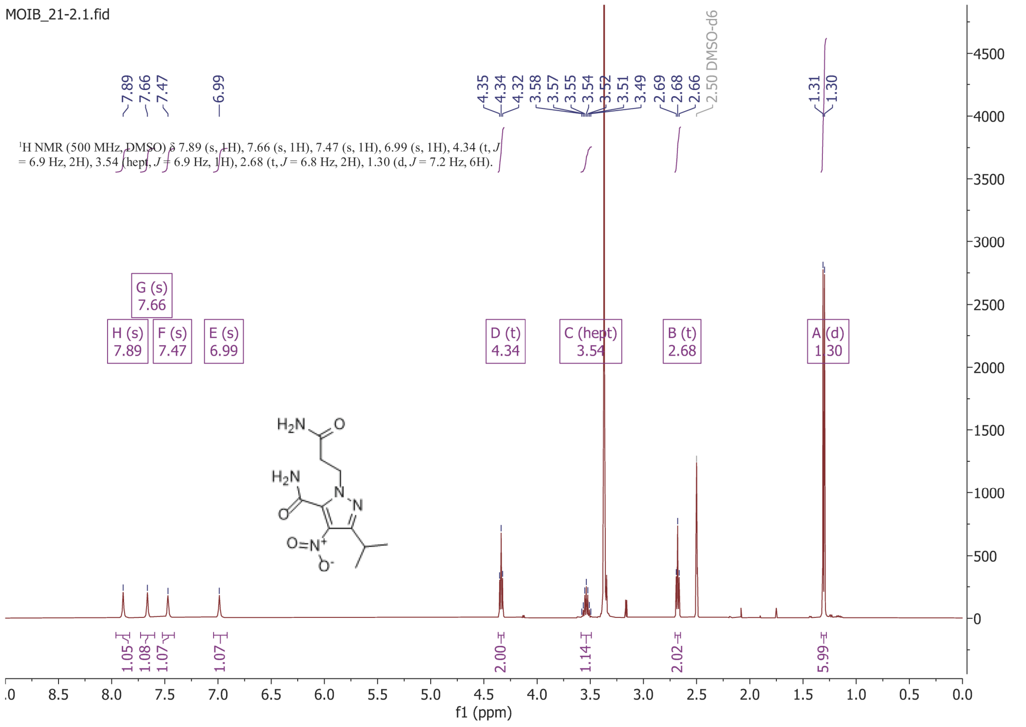

Figure S61. ^{1}H NMR spectrum of compound **19f**.

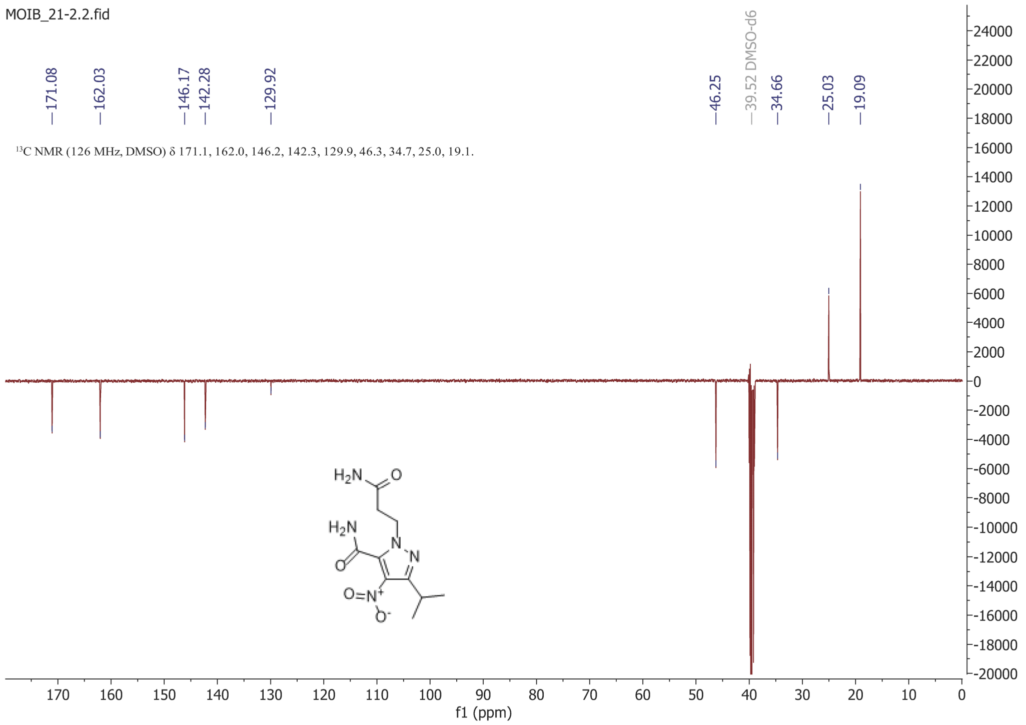

Figure S62. ^{13}C NMR spectrum of compound **19f**.

Acquired by	: Admin
Date Acquired	: 21/6/2019 1:13:59 PM
Sample Name	: MOIB_27-af
Sample ID	:
Tray#	: 1
Vial#	: 23
Injection Volume	: 1
Data File	: C:\LabSolutions\Data\2019\2019-wk25\MOIB_27-af.lcd
Background File	: blanco_an 21062019.lcd
Method File	: Method SCAN ACID standard.lcm
Report Format	: DefaultLCMS.lcr
Tuning File	: C:\LabSolutions\Tuning\Tuning-ESI-pos-neg01072015a.lct
Processed by	: Admin
Modified Date	: 21/6/2019 1:27:50 PM

PDA Graph

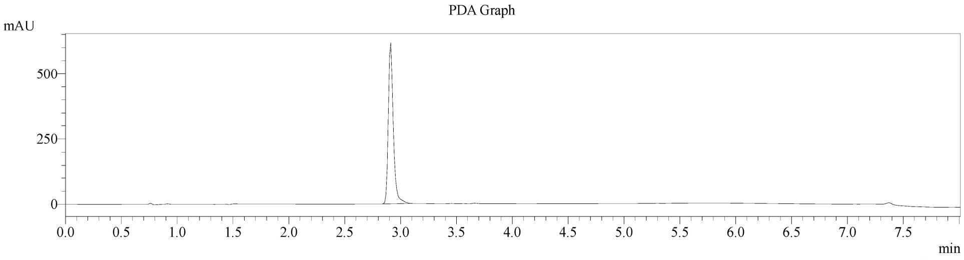

PeakTable

PDA Ch1 254nm 4nm

Peak#	Name	Ret. Time	Area	Area %
1		2.904	1902079	100.000

MS Chromatogram
MOIB_27-af C:\LabSolutions\Data\2019\2019-wk25\MOIB_27-af.lcd

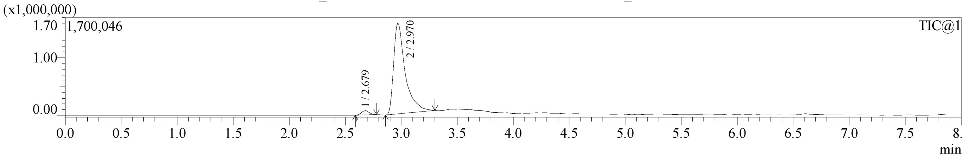

MS Spectrum Graph

#:1 Ret.Time:Averaged 2.960-2.980(Scan#:297-299)
BG Mode:Calc 2.860<->3.300(287<->331)
Mass Peaks:7 Base Peak:211.05(1030108) Polarity:Pos Segment1 - Event1

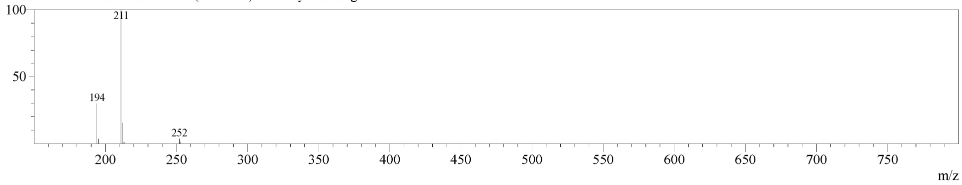

MS Spectrum Table

#:1 Ret.Time:
BG Mode:Calc 2.860<->3.300(287<->331)
Mass Peaks:7 Base Peak:211.05(1030108) Polarity:Pos Segment1 - Event1

#	m/z	Abs.Inten.	Rel.Inten.	Charge	Polarity	Monoisotopic
1	194.00	309501	30.05			
2	195.05	36760	3.57			
3	211.05	1030108	100.00			
4	212.05	159664	15.50			
5	213.10	13033	1.27			
6	252.10	37148	3.61			
7	253.05	14027	1.36			

Figure S63. LCMS spectrum of compound **20b**.

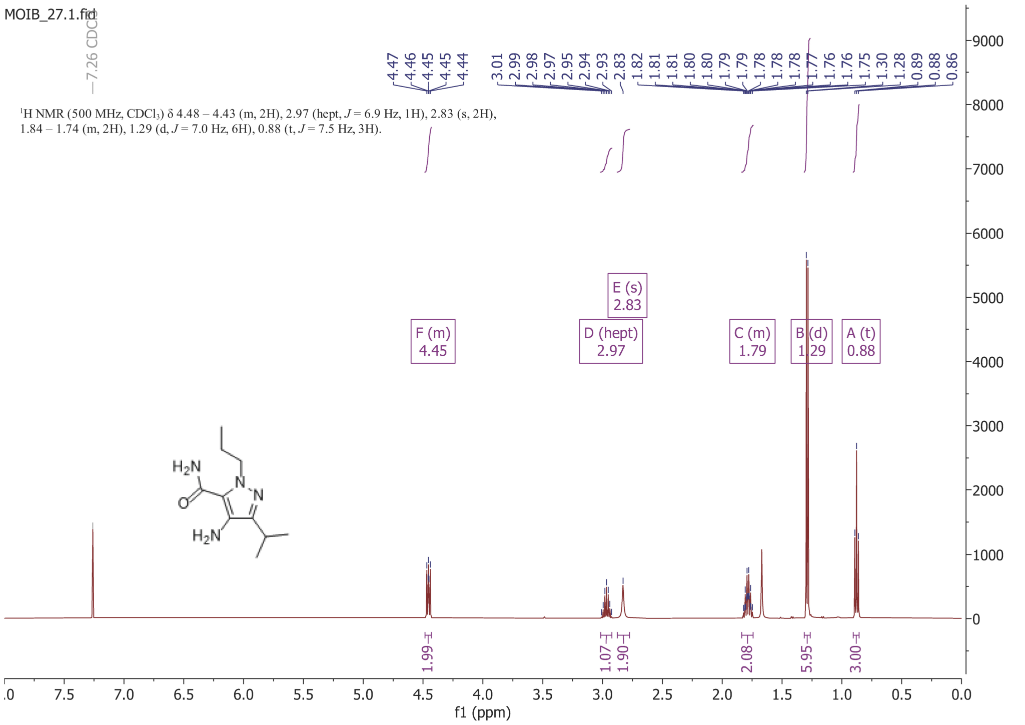

Figure S64. ^{1}H NMR spectrum of compound **20b**.

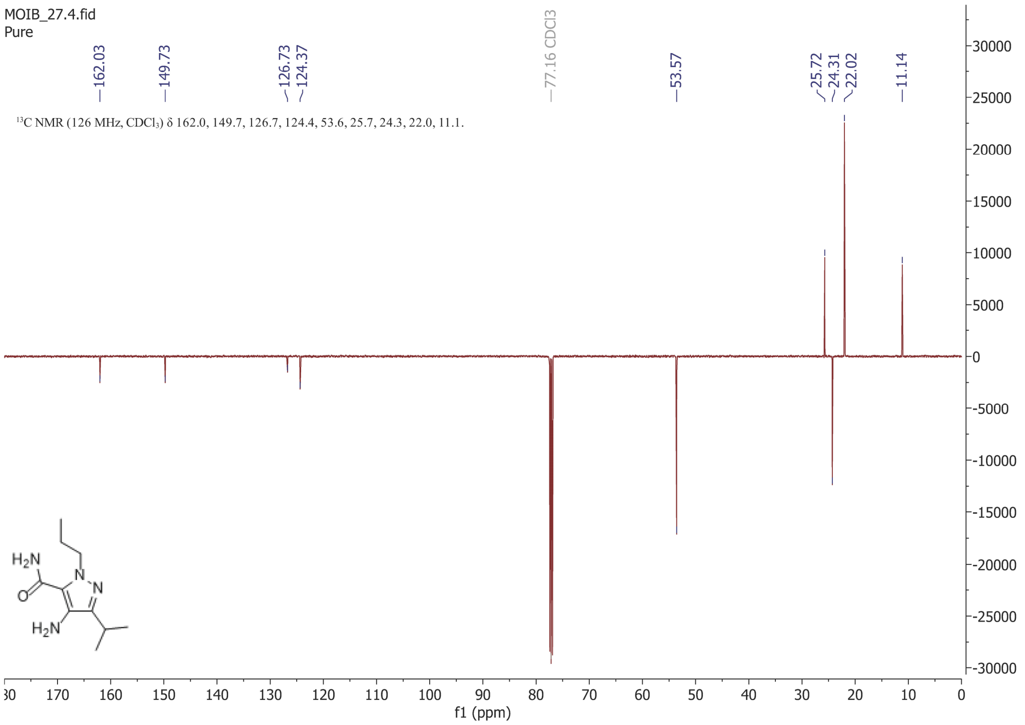

Figure S65. ^{13}C NMR spectrum of compound **20b**.

Acquired by : Admin
Date Acquired : 10/4/2019 9:48:23 AM
Sample Name : MOIB_6_pure
Sample ID :
Tray# : 1
Vial# : 44
Injection Volume : 1
Data File : C:\LabSolutions\Data\2019\2019-wk15\MOIB_6_pure.lcd
Background File : blanco 10042019.lcd
Method File : Method SCAN ACID standard.lcm
Report Format : DefaultLCMS.lcr
Tuning File : C:\LabSolutions\Tuning\Tuning-ESI-pos-neg01072015a.lct
Processed by : Admin
Modified Date : 30/3/2021 5:22:22 PM

PDA Graph

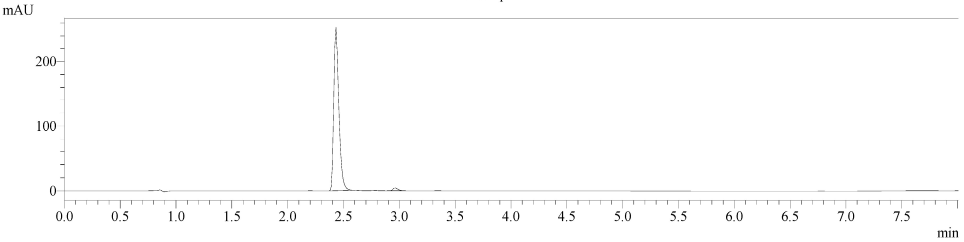

PeakTable

PDA Ch1 254nm 4nm

Peak#	Name	Ret. Time	Area	Area %
1		2.426	794189	98.234
2		2.956	14276	1.766

MS Chromatogram
MOIB_6_pure C:\LabSolutions\Data\2019\2019-wk11-20\2019-wk15\MOIB_6_pure.lcd

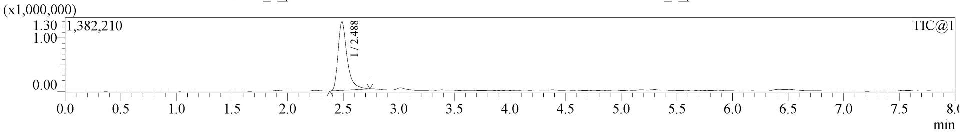

MS Spectrum Graph

#:1 Ret.Time:Averaged 2.480-2.500(Scan#:249-251)
BG Mode:Calc 2.380<->2.740(239<->275)
Mass Peaks:8 Base Peak:227.10(974979) Polarity:Pos Segment1 - Event1

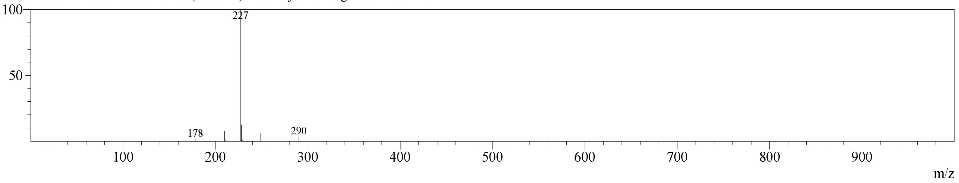

MS Spectrum Table

#:1 Ret.Time:
BG Mode:Calc 2.380<->2.740(239<->275)
Mass Peaks:8 Base Peak:227.10(974979) Polarity:Pos Segment1 - Event1

#	m/z	Abs.Inten.	Rel.Inten.	Charge	Polarity	Monoisotopic	#	m/z	Abs.Inten.	Rel.Inten.	Charge	Polarity	Monoisotopic
1	178.05	16005	1.64				5	228.05	121523	12.46			
2	210.05	73336	7.52				6	229.10	11674	1.20			
3	211.10	12632	1.30				7	249.05	60850	6.24			
4	227.10	974979	100.00				8	290.10	32523	3.34			

Figure S66. LCMS spectrum of compound **20d**.

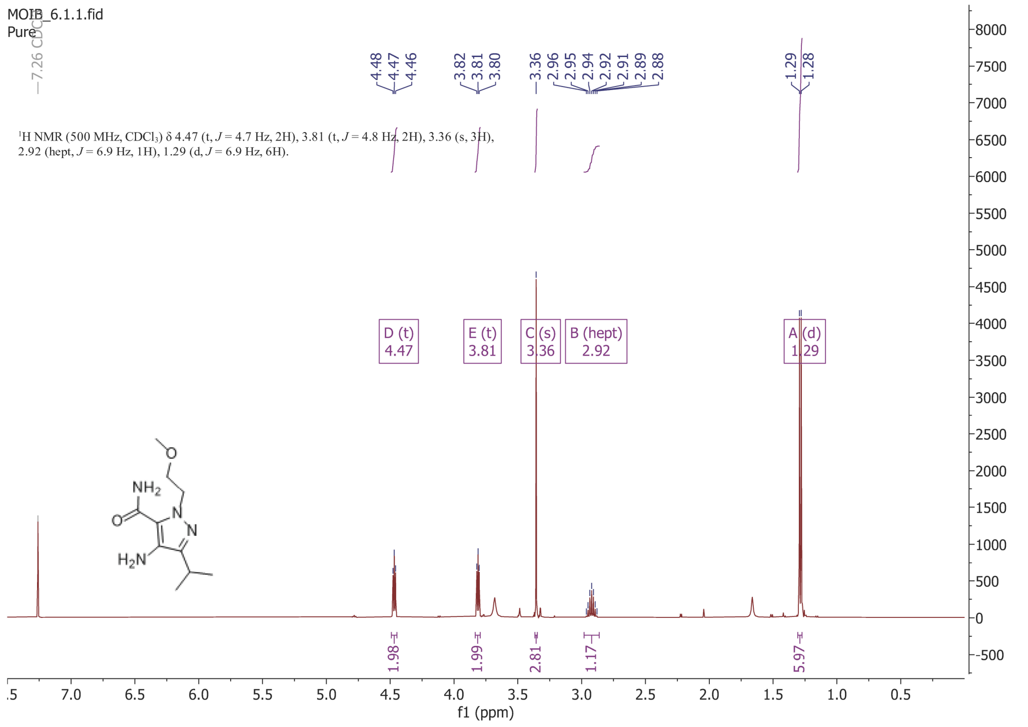

Figure S67. ^{1}H NMR spectrum of compound **20d**.

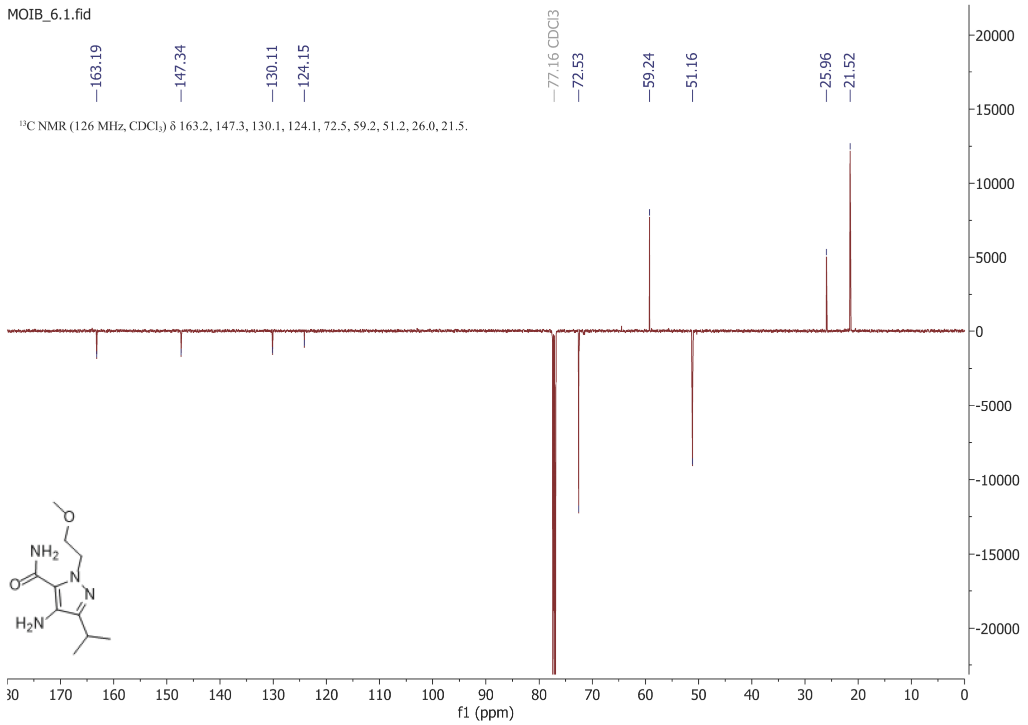

Figure S68. ^{13}C NMR spectrum of compound **20d**.

Acquired by	: Admin
Date Acquired	: 3/5/2019 4:04:24 PM
Sample Name	: MOIB_13-5
Sample ID	:
Tray#	: 1
Vial#	: 42
Injection Volume	: 1
Data File	: C:\LabSolutions\Data\2019\2019-wk18\MOIB_13-5.lcd
Background File	: blanco 03052019.lcd
Method File	: Method SCAN ACID standard.lcm
Report Format	: DefaultLCMS.lcr
Tuning File	: C:\LabSolutions\Tuning\Tuning-ESI-pos-neg01072015a.lct
Processed by	: Admin
Modified Date	: 3/5/2019 4:13:44 PM

Chromatogram
MOIB_13-5 C:\LabSolutions\Data\2019\2019-wk18\MOIB_13-5.lcd

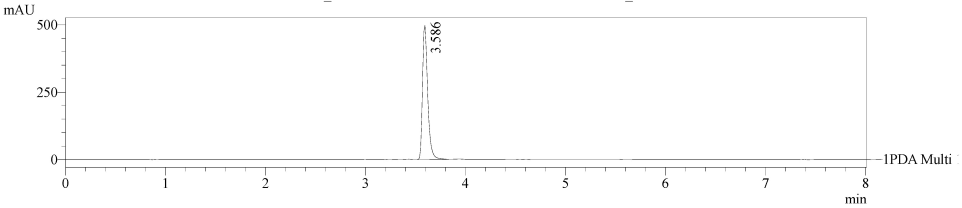

1 PDA Multi 1 / 254nm 4nm
PDA Ch1 254nm 4nm

Peak#	Name	Ret. Time	Area	Area %
1		3.586	1786961	100.000

MS Chromatogram
MOIB_13-5 C:\LabSolutions\Data\2019\2019-wk18\MOIB_13-5.lcd

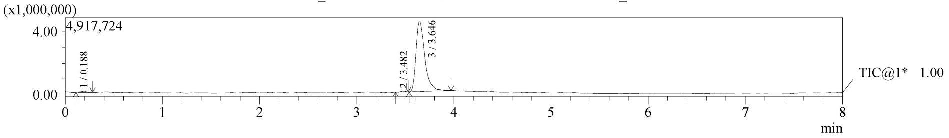

MS Spectrum Graph

#:1 Ret.Time:Averaged 3.640-3.660(Scan#:365-367)
Mass Peaks:5 Base Peak:303.15(3195339) Polarity:Pos Segment1 - Event1

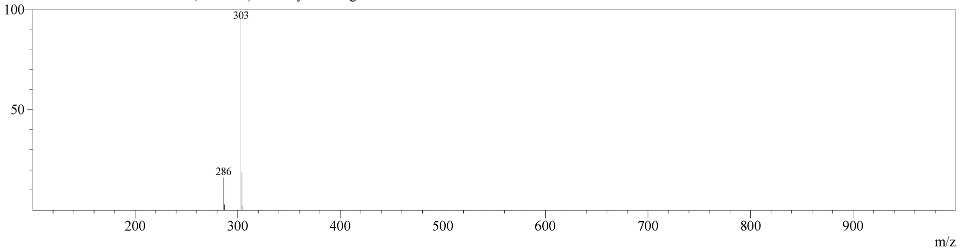

MS Spectrum Table

#:1 Ret.Time:
BG Mode:Calc 3.540<->3.970(355<->398)
Mass Peaks:5 Base Peak:303.15(3195339) Polarity:Pos Segment1 - Event1

#	m/z	Abs.Inten.	Rel.Inten.	Charge	Polarity	Monoisotopic
1	286.05	521756	16.33			
2	287.10	88123	2.76			
3	303.15	3195339	100.00			
4	304.15	606797	18.99			
5	305.15	63853	2.00			

Figure S69. LCMS spectrum of compound **20e**.

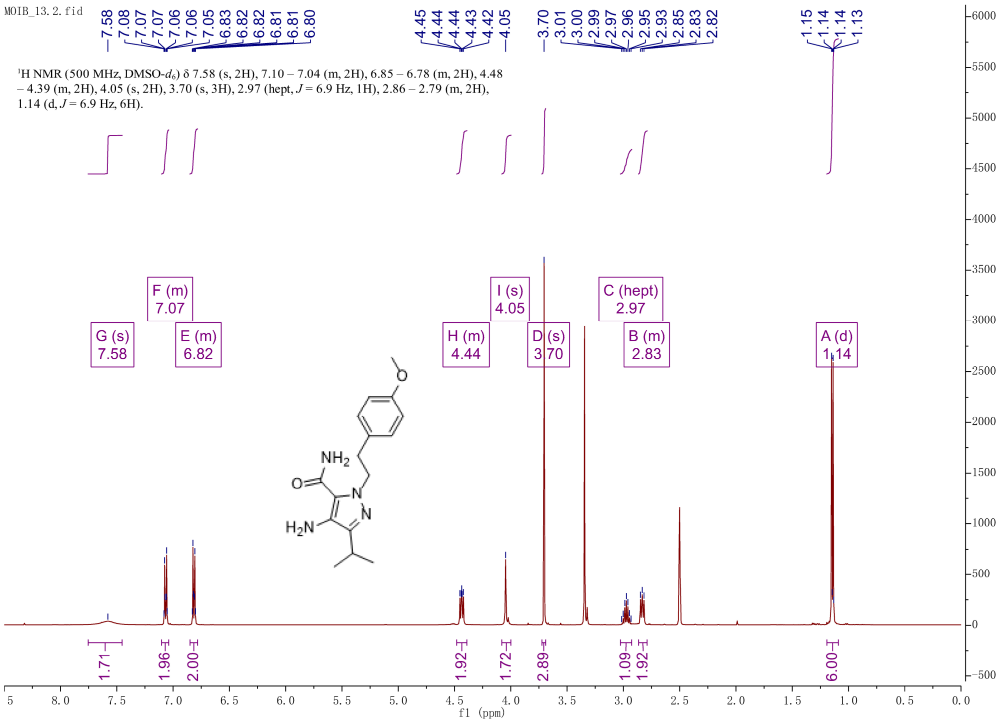

Figure S70. ^{1}H NMR spectrum of compound **20e**.

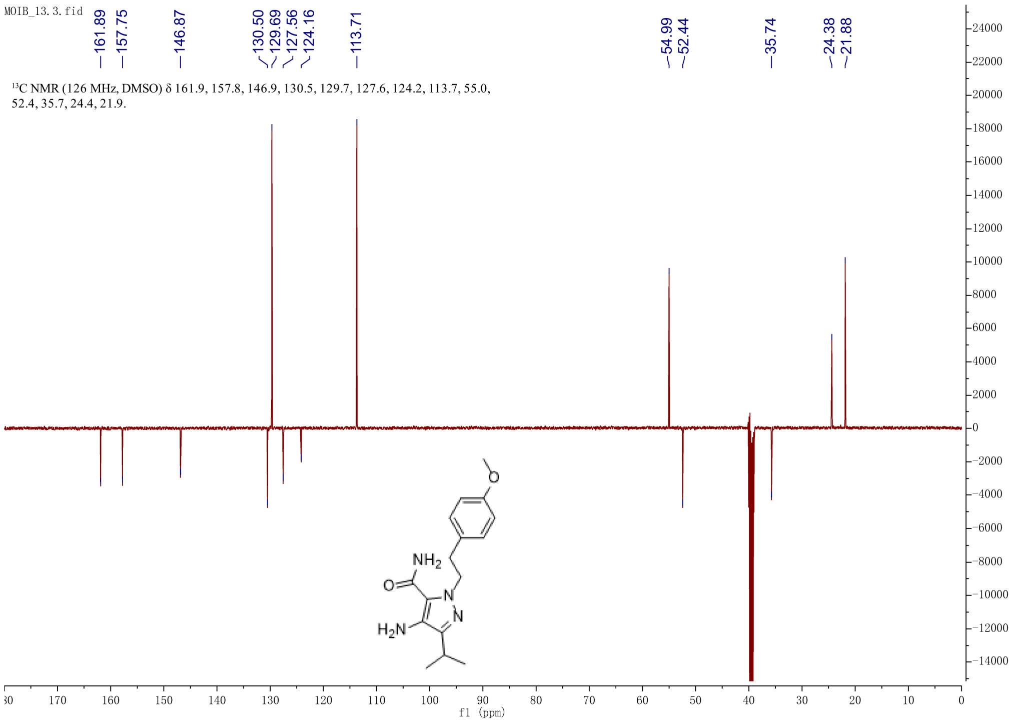

Figure S71. ^{13}C NMR spectrum of compound **20e**.

Acquired by	: Admin
Date Acquired	: 17/4/2019 1:15:29 PM
Sample Name	: YAZH_40_20190417
Sample ID	:
Tray#	: 1
Vial#	: 7
Injection Volume	: 1
Data File	: C:\LabSolutions\Data\2019\2019-wk16\YAZH_40_20190417.lcd
Background File	: blanco 17042019.lcd
Method File	: Method SCAN ACID standard.lcm
Report Format	: DefaultLCMS.lcr
Tuning File	: C:\LabSolutions\Tuning\Tuning-ESI-pos-neg01072015a.lct
Processed by	: Admin
Modified Date	: 17/4/2019 2:32:24 PM

PDA Graph

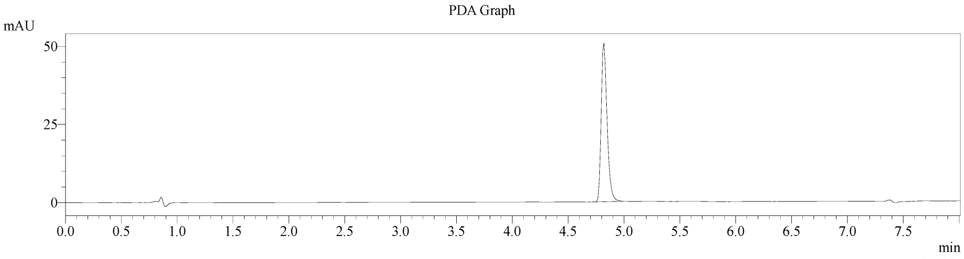

PeakTable

PDA Ch1 254nm 4nm

Peak#	Name	Ret. Time	Area	Area %
1		4.813	187701	100.000

MS Chromatogram
YAZH_40_20190417 C:\LabSolutions\Data\2019\2019-wk16\YAZH_40_20190417.lcd

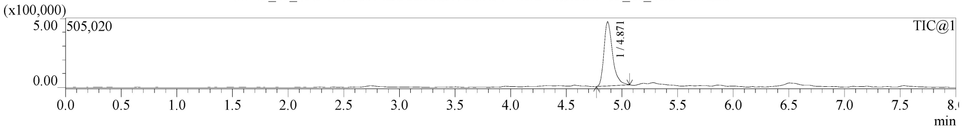

MS Spectrum Graph

#:1 Ret.Time:Averaged 4.860-4.880(Scan#:487-489)
BG Mode:Calc 4.770<->5.070(478<->508)
Mass Peaks:5 Base Peak:301.10(387117) Polarity:Pos Segment1 - Event1

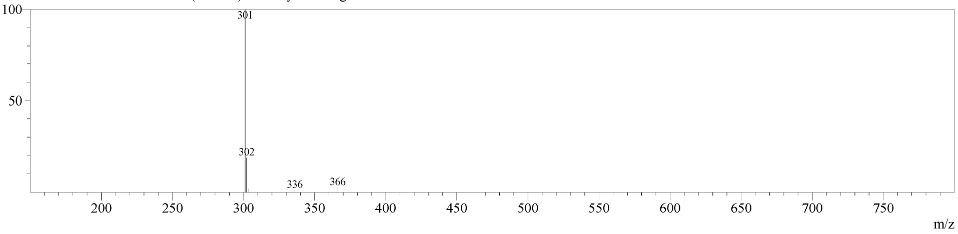

MS Spectrum Table

#:1 Ret.Time:
BG Mode:Calc 4.770<->5.070(478<->508)
Mass Peaks:5 Base Peak:301.10(387117) Polarity:Pos Segment1 - Event1

#	m/z	Abs.Inten.	Rel.Inten.	Charge	Polarity	Monoisotopic
1	301.10	387117	100.00			
2	302.10	72822	18.81			
3	303.15	7328	1.89			
4	336.10	3948	1.02			
5	366.25	9420	2.43			

Figure S72. LCMS spectrum of compound **21a**.

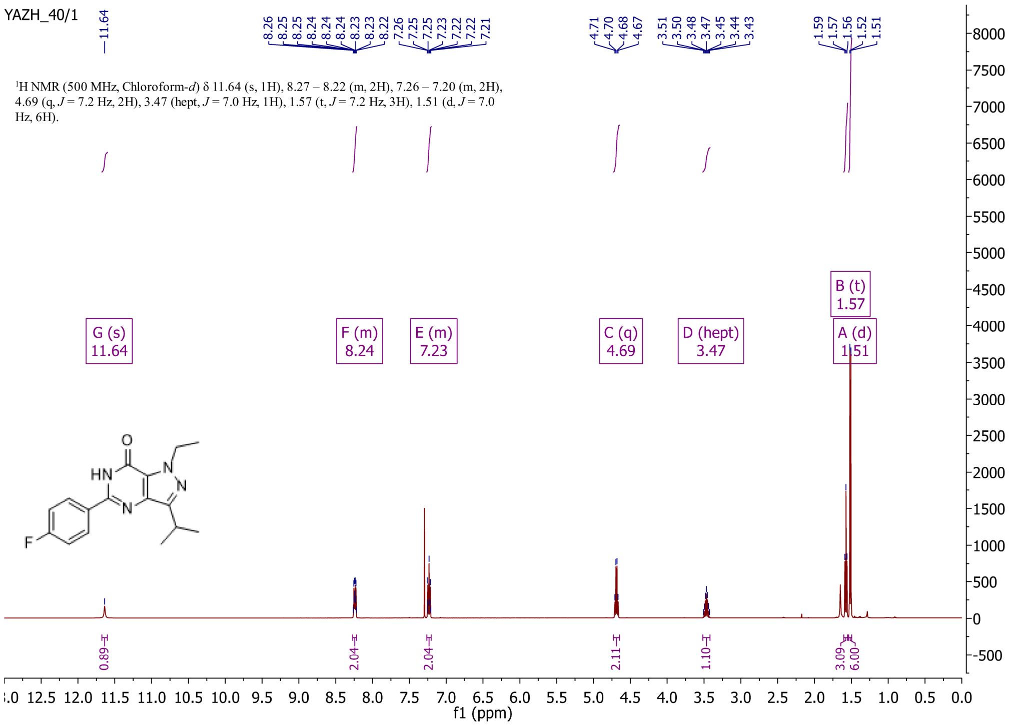

Figure S73. ^{1}H NMR spectrum of compound **21a**.

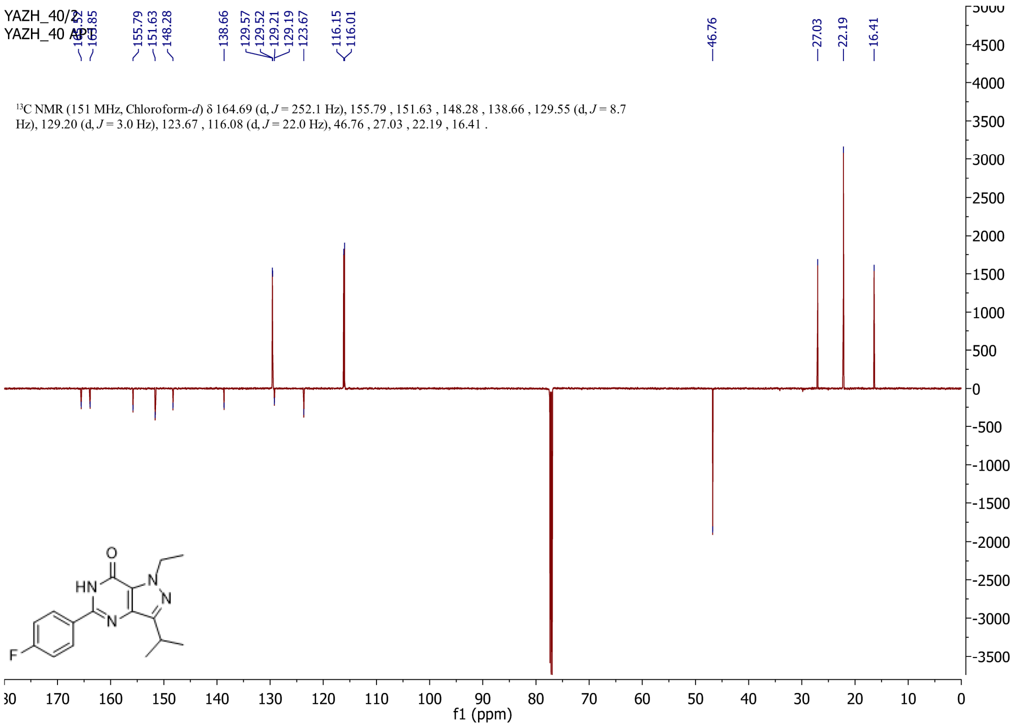

Figure S74. ^{13}C NMR spectrum of compound **21a**.

Acquired by	: Admin
Date Acquired	: 27/6/2019 2:18:26 PM
Sample Name	: MOIB_28_rc_pure
Sample ID	:
Tray#	: 1
Vial#	: 43
Injection Volume	: 1
Data File	: C:\LabSolutions\Data\2019\2019-wk26\MOIB_28_rc_pure.lcd
Background File	: blanco 27062019.lcd
Method File	: Method SCAN ACID standard.lcm
Report Format	: DefaultLCMS.lcr
Tuning File	: C:\LabSolutions\Tuning\Tuning-ESI-pos-neg01072015a.lct
Processed by	: Admin
Modified Date	: 27/6/2019 2:31:01 PM

PDA Graph

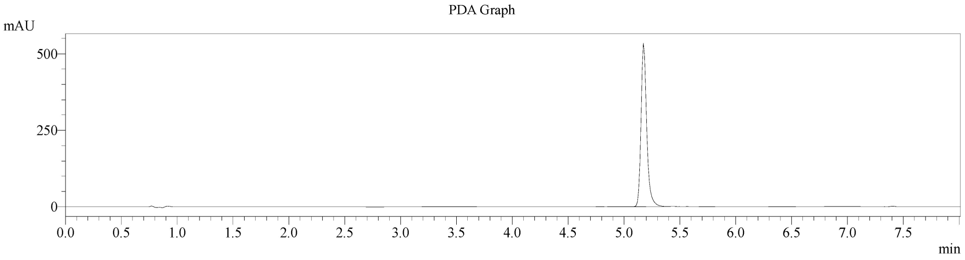

PeakTable

PDA Ch1 254nm 4nm

Peak#	Name	Ret. Time	Area	Area %
1		5.170	1889998	100.000

MS Chromatogram
MOIB_28_rc_pure C:\LabSolutions\Data\2019\2019-wk26\MOIB_28_rc_pure.lcd

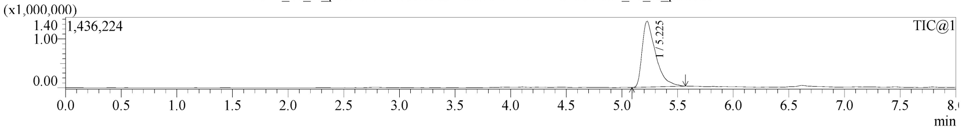

MS Spectrum Graph

#:1 Ret.Time:Single 5.240(Scan#:525)
BG Mode:None
Mass Peaks:8 Base Peak:315.10(917553) Polarity:Pos Segment1 - Event1

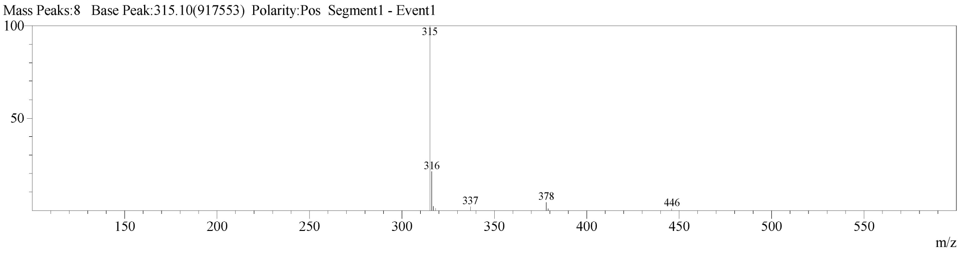

MS Spectrum Table

#:1 Ret.Time:
BG Mode:None
Mass Peaks:8 Base Peak:315.10(917553) Polarity:Pos Segment1 - Event1

#	m/z	Abs.Inten.	Rel.Inten.	Charge	Polarity	Monoisotopic	#	m/z	Abs.Inten.	Rel.Inten.	Charge	Polarity	Monoisotopic
1	315.10	917553	100.00				5	337.05	18540	2.02			
2	316.10	194142	21.16				6	378.05	41095	4.48			
3	317.00	20939	2.28				7	379.10	9874	1.08			
4	318.20	11618	1.27				8	446.00	9436	1.03			

Figure S75. LCMS spectrum of compound **21b**.

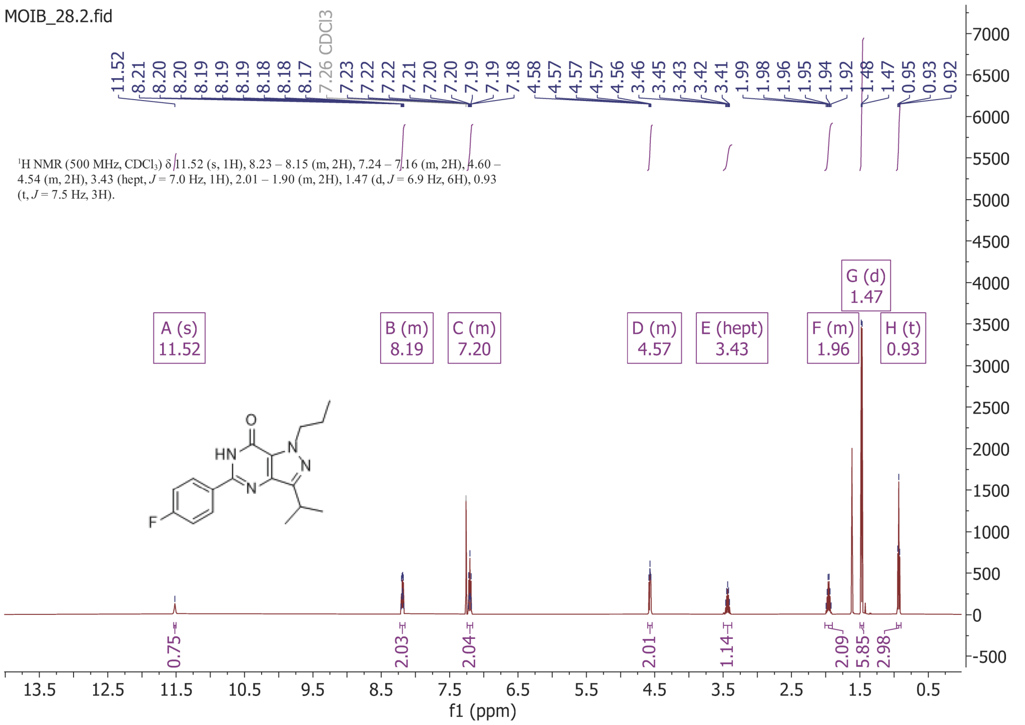

Figure S76. ^{1}H NMR spectrum of compound **21b**.

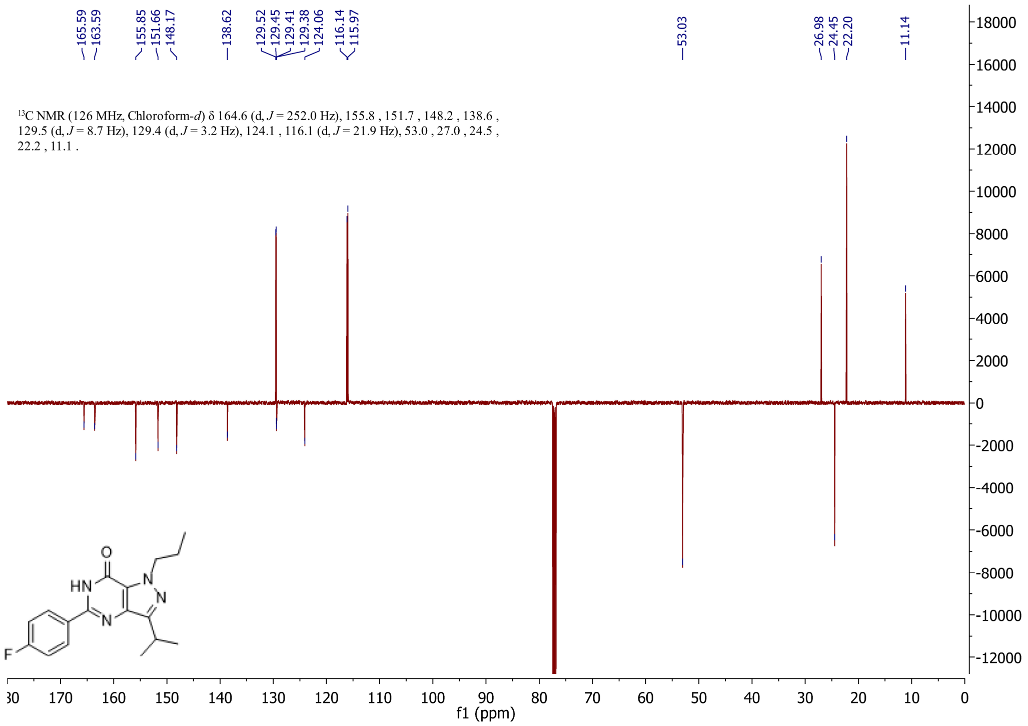

Figure S77. ^{13}C NMR spectrum of compound **21b**.

Acquired by	: Admin
Date Acquired	: 10/7/2019 10:42:25 AM
Sample Name	: YAZH_46
Sample ID	:
Tray#	: 1
Vial#	: 8
Injection Volume	: 2
Data File	: C:\LabSolutions\Data\2019\2019-wk28\YAZH_46.lcd
Background File	: blanco10072019.lcd
Method File	: Method SCAN ACID standard.lcm
Report Format	: DefaultLCMS.lcr
Tuning File	: C:\LabSolutions\Tuning\Tuning-ESI-pos-neg01072015a.lct
Processed by	: Admin
Modified Date	: 10/7/2019 11:01:10 AM

Chromatogram
YAZH_46 C:\LabSolutions\Data\2019\2019-wk28\YAZH_46.lcd

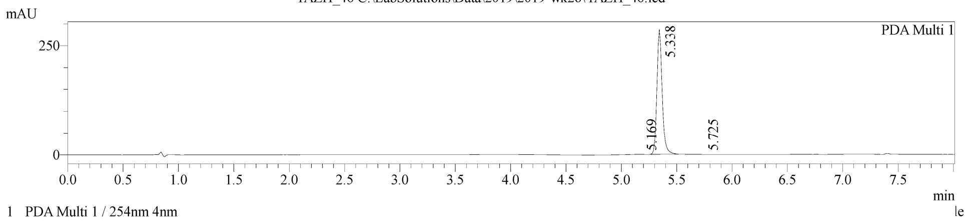

1 PDA Multi 1 / 254nm 4nm
PDA Ch1 254nm 4nm

Peak#	Name	Ret. Time	Area	Area %
1		5.169	1664	0.175
2		5.338	945899	99.746
3		5.725	748	0.079

MS Chromatogram
YAZH_46 C:\LabSolutions\Data\2019\2019-wk28\YAZH_46.lcd

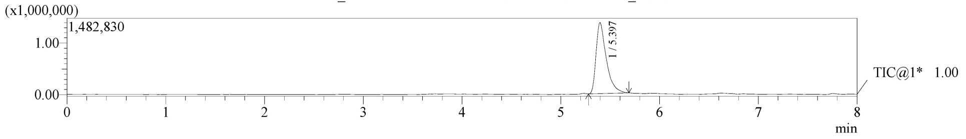

MS Spectrum Graph
#:1 Ret.Time:Averaged 5.390-5.410(Scan#:540-542)
Mass Peaks:5 Base Peak:315.10(1043830) Polarity:Pos Segment1 - Event1

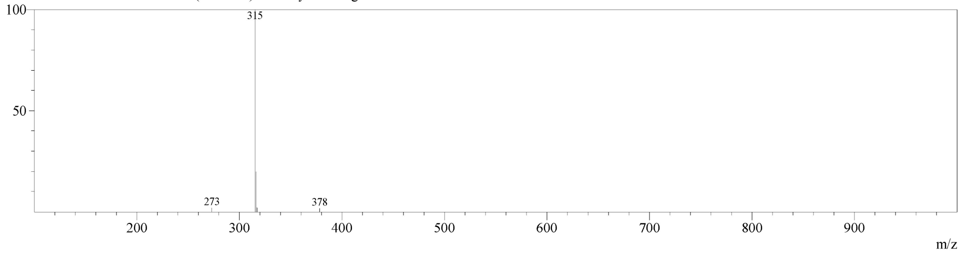

MS Spectrum Table
#:1 Ret.Time:
BG Mode:Calc 5.280<->5.690(529<->570)
Mass Peaks:5 Base Peak:315.10(1043830) Polarity:Pos Segment1 - Event1

#	m/z	Abs.Inten.	Rel.Inten.	Charge	Polarity	Monoisotopic
1	273.00	21147	2.03			
2	315.10	1043830	100.00			
3	316.10	207896	19.92			
4	317.15	21843	2.09			
5	378.15	18923	1.81			

Figure S78. LCMS spectrum of compound **21c**.

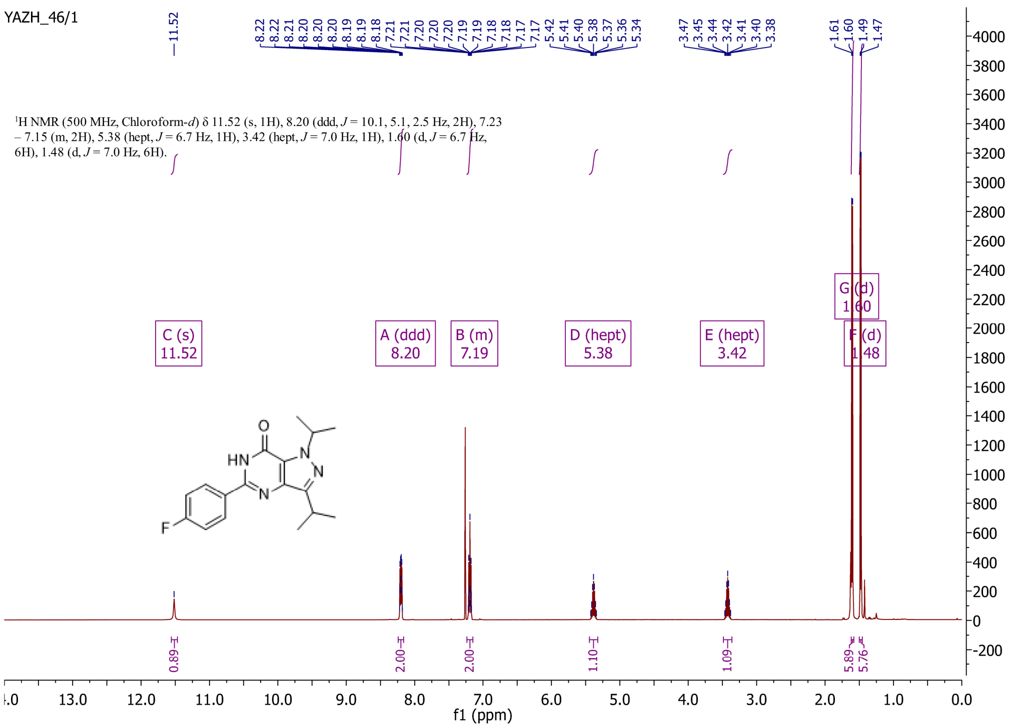

Figure S79. ^{1}H NMR spectrum of compound **21c**.

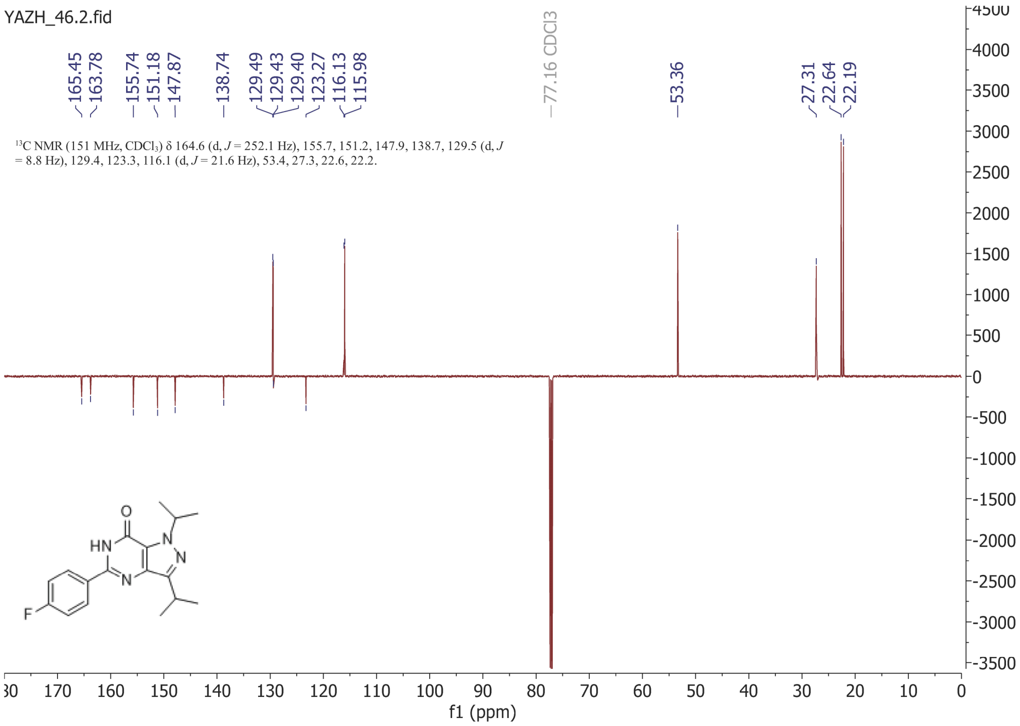

Figure S80. ^{13}C NMR spectrum of compound **21c**.

Acquired by	: Admin
Date Acquired	: 26/4/2019 12:01:34 PM
Sample Name	: MOIB_10_final_pure
Sample ID	:
Tray#	: 1
Vial#	: 16
Injection Volume	: 1
Data File	: C:\LabSolutions\Data\2019\2019-wk17\MOIB_10_final_pure.lcd
Background File	: blanco 26042019.lcd
Method File	: Method SCAN ACID standard.lcm
Report Format	: DefaultLCMS.lcr
Tuning File	: C:\LabSolutions\Tuning\Tuning-ESI-pos-neg01072015a.lct
Processed by	: Admin
Modified Date	: 17/6/2019 4:58:25 PM

PDA Graph

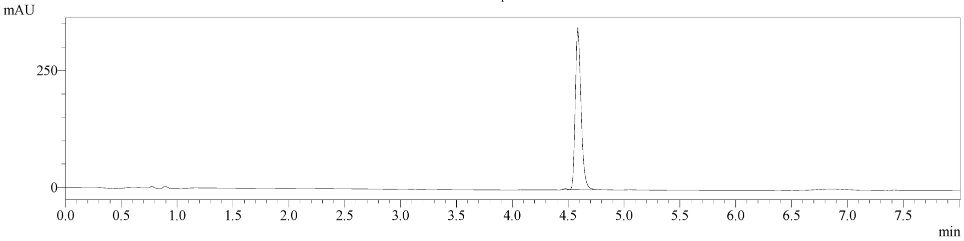

PeakTable

PDA Ch1 254nm 4nm

Peak#	Name	Ret. Time	Area	Area %
1		4.469	5959	0.482
2		4.581	1231102	99.518

MS Chromatogram

MOIB_10_final_pure C:\LabSolutions\Data\2019\2019-wk11-20\2019-wk17\MOIB_10_final_pure.lcd

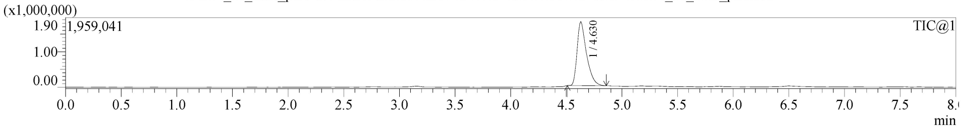

MS Spectrum Graph

#:5 Ret.Time:Averaged 4.620-4.640(Scan#:463-465)
BG Mode:Calc 4.510<->4.860(452<->487)
Mass Peaks:5 Base Peak:331.10(1435605) Polarity:Pos Segment1 - Event1

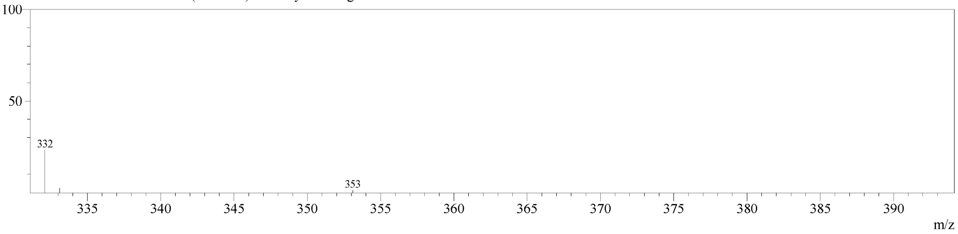

MS Spectrum Table

#:5 Ret.Time:
BG Mode:Calc 4.510<->4.860(452<->487)
Mass Peaks:5 Base Peak:331.10(1435605) Polarity:Pos Segment1 - Event1

#	m/z	Abs.Inten.	Rel.Inten.	Charge	Polarity	Monoisotopic
1	331.10	1435605	100.00			
2	332.10	336947	23.47			
3	333.10	36941	2.57			
4	353.10	20172	1.41			
5	394.15	17736	1.24			

Figure S81. LCMS spectrum of compound **21d**.

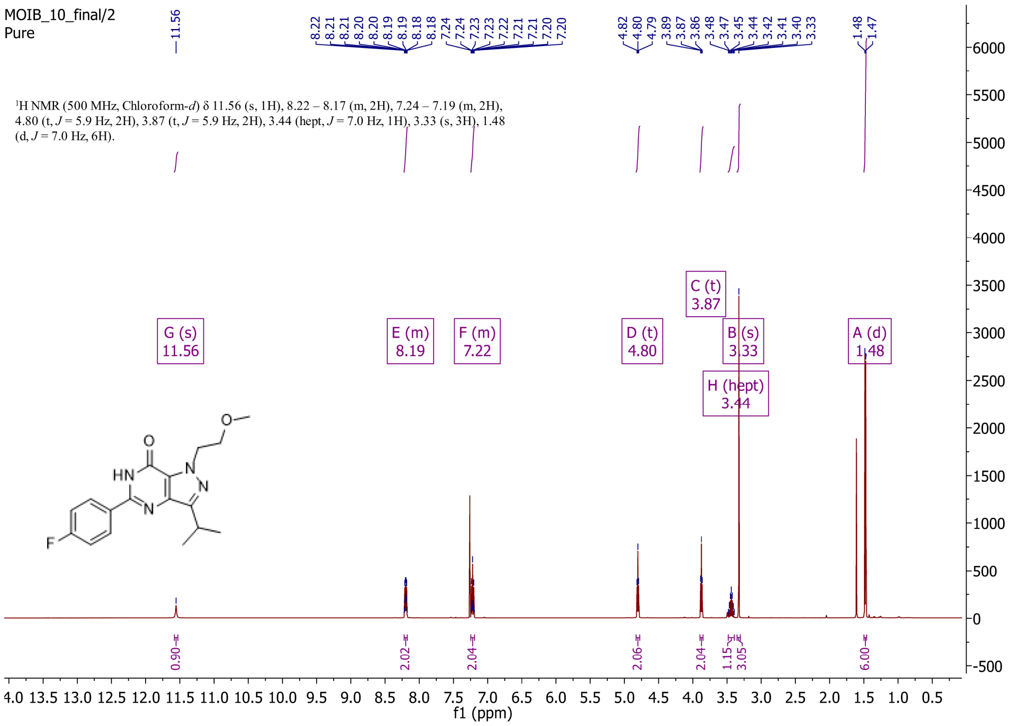

Figure S82. ^{1}H NMR spectrum of compound **21d**.

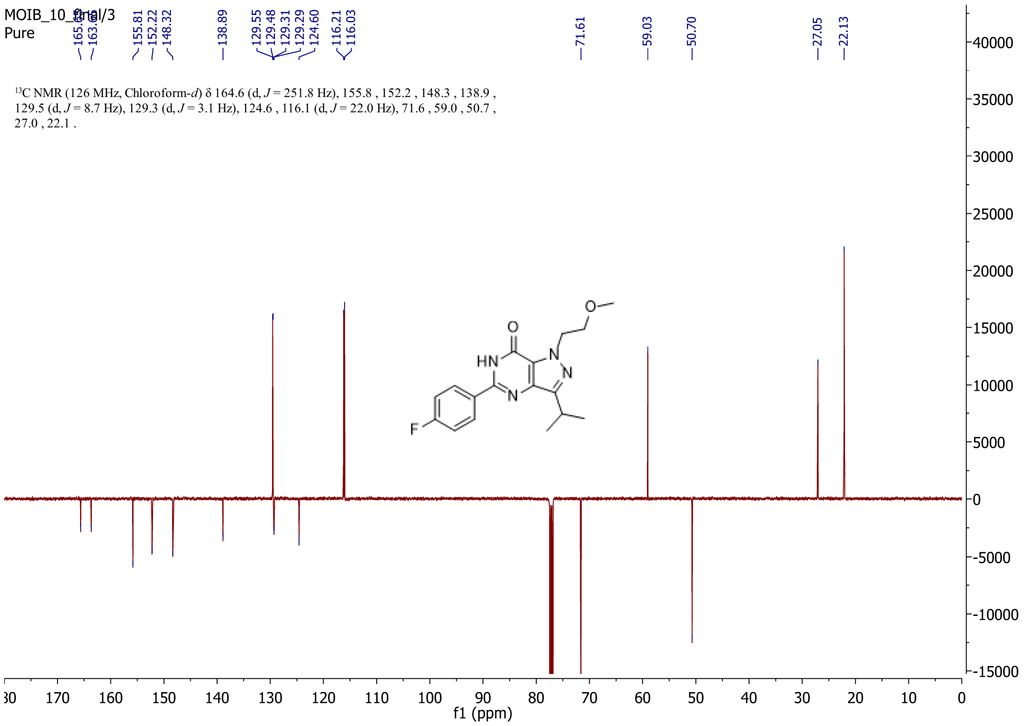

Figure S83. ^{13}C NMR spectrum of compound **21d**.

Acquired by	: Admin
Date Acquired	: 7/5/2019 5:45:32 PM
Sample Name	: MOIB_14_RC_pure
Sample ID	:
Tray#	: 1
Vial#	: 64
Injection Volume	: 1
Data File	: C:\LabSolutions\Data\2019\2019-wk19\MOIB_14_RC_pure.lcd
Background File	: blanco 07052019.lcd
Method File	: Method SCAN ACID standard.lcm
Report Format	: DefaultLCMS.lcr
Tuning File	: C:\LabSolutions\Tuning\Tuning-ESI-pos-neg01072015a.lct
Processed by	: Admin
Modified Date	: 17/6/2019 5:01:55 PM

PDA Graph

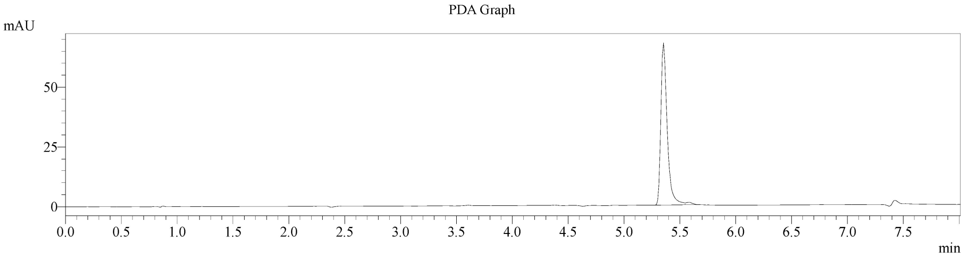

PeakTable

PDA Ch1 254nm 4nm

Peak#	Name	Ret. Time	Area	Area %
1		5.348	268843	98.593
2		5.574	3836	1.407

MS Chromatogram

MOIB_14_RC_pure C:\LabSolutions\Data\2019\2019-wk11-20\2019-wk19\MOIB_14_RC_pure.lcd

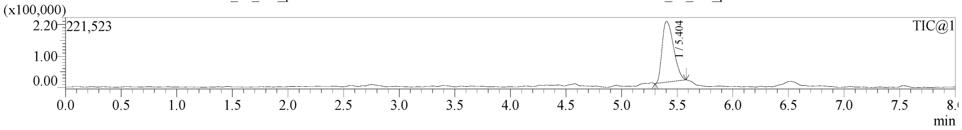

MS Spectrum Graph

#:6 Ret.Time:Averaged 5.390-5.410(Scan#:540-542)

BG Mode:Calc 5.300<->5.580(531<->559)

Mass Peaks:7 Base Peak:407.15(132273) Polarity:Pos Segment1 - Event1

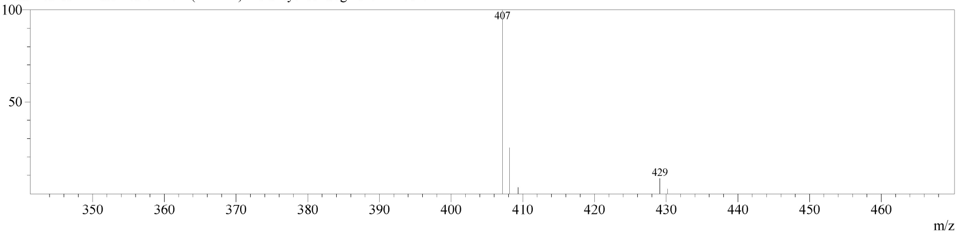

MS Spectrum Table

#:6 Ret.Time:

BG Mode:Calc 5.300<->5.580(531<->559)

Mass Peaks:7 Base Peak:407.15(132273) Polarity:Pos Segment1 - Event1

#	m/z	Abs.Inten.	Rel.Inten.	Charge	Polarity	Monoisotopic
1	341.30	1343	1.02			
2	407.15	132273	100.00			
3	408.15	33503	25.33			
4	409.35	4694	3.55			

#	m/z	Abs.Inten.	Rel.Inten.	Charge	Polarity	Monoisotopic
5	429.10	11031	8.34			
6	430.20	3538	2.67			
7	470.20	3787	2.86			

Figure S84. LCMS spectrum of compound **21e**.

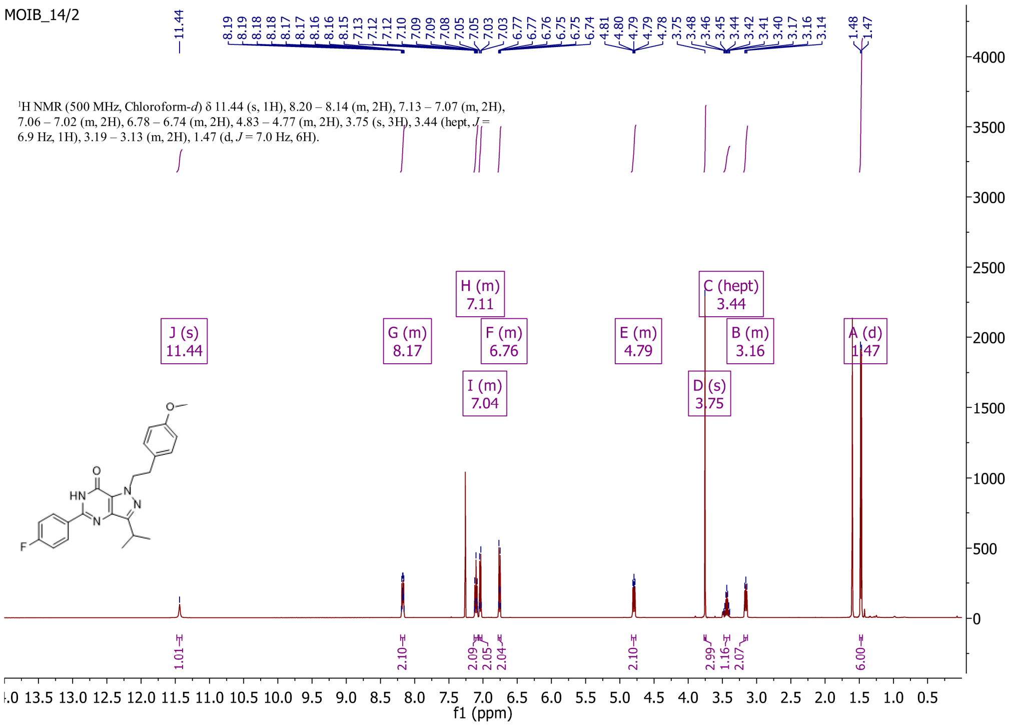

Figure S85. ^{1}H NMR spectrum of compound **21e**.

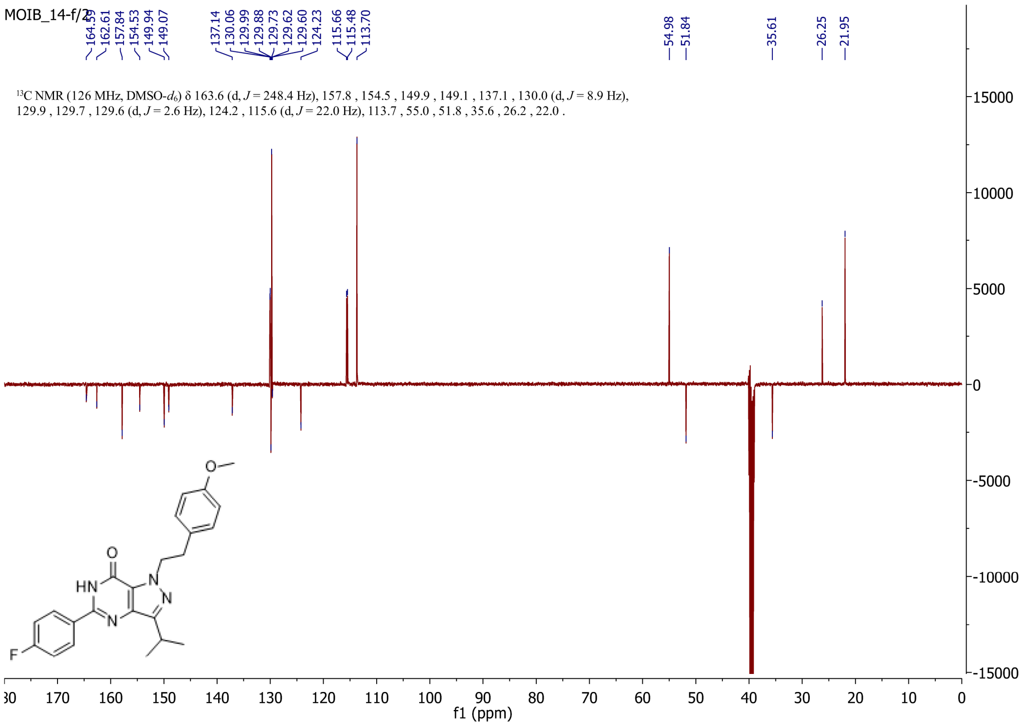

Figure S86. ^{13}C NMR spectrum of compound **21e**.

Acquired by : Admin
Date Acquired : 10/7/2019 5:27:37 PM
Sample Name : MOIB_33_rc
Sample ID :
Tray# : 1
Vial# : 51
Injection Volume : 1
Data File : C:\LabSolutions\Data\2019\2019-wk28\MOIB_33_rc.lcd
Background File : blanco10072019.lcd
Method File : Method SCAN ACID standard.lcm
Report Format : DefaultLCMS.lcr
Tuning File : C:\LabSolutions\Tuning\Tuning-ESI-pos-neg01072015a.lct
Processed by : Admin
Modified Date : 11/7/2019 2:39:12 PM

Chromatogram
MOIB_33_rc C:\LabSolutions\Data\2019\2019-wk28\MOIB_33_rc.lcd

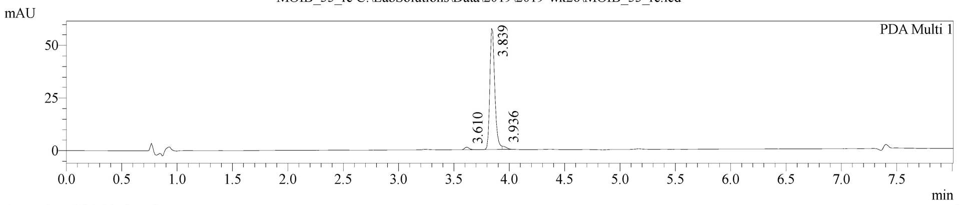

1 PDA Multi 1 / 254nm 4nm
PDA Ch1 254nm 4nm

Peak#	Name	Ret. Time	Area	Area %
1		3.610	3354	1.806
2		3.839	178768	96.262
3		3.936	3589	1.932

MS Chromatogram
MOIB_33_rc C:\LabSolutions\Data\2019\2019-wk28\MOIB_33_rc.lcd

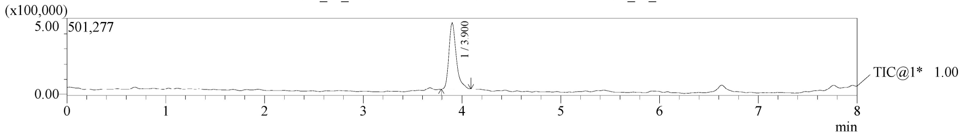

#:2 Ret.Time:Averaged 3.890-3.910(Scan#:390-392)
Mass Peaks:22 Base Peak:344.05(236385) Polarity:Pos Segment1 - Event1

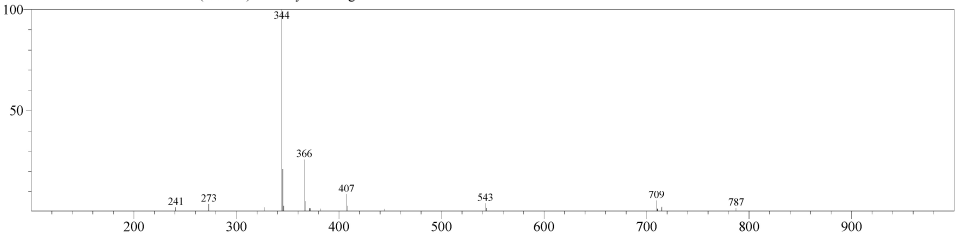

#:2 Ret.Time:
BG Mode:Calc 3.790<->4.090(380<->410)
Mass Peaks:22 Base Peak:344.05(236385) Polarity:Pos Segment1 - Event1

#	m/z	Abs.Inten.	Rel.Inten.	Charge	Polarity	Monoisotopic
1	240.80	4303	1.82			
2	273.05	8266	3.50			
3	327.05	4572	1.93			
4	344.05	236385	100.00			
5	345.15	50190	21.23			
6	346.15	6162	2.61			
7	366.10	61037	25.82			
8	367.05	11640	4.92			
9	371.30	3347	1.42			
10	371.80	3246	1.37			
11	382.15	2841	1.20			

#	m/z	Abs.Inten.	Rel.Inten.	Charge	Polarity	Monoisotopic
12	407.15	20315	8.59			
13	408.15	6037	2.55			
14	444.10	2386	1.01			
15	542.75	9356	3.96			
16	543.75	3479	1.47			
17	709.35	12682	5.36			
18	710.35	2952	1.25			
19	710.65	2410	1.02			
20	714.30	4332	1.83			
21	714.95	5069	2.14			
22	787.25	3681	1.56			

Figure S87. LCMS spectrum of compound **21f**.

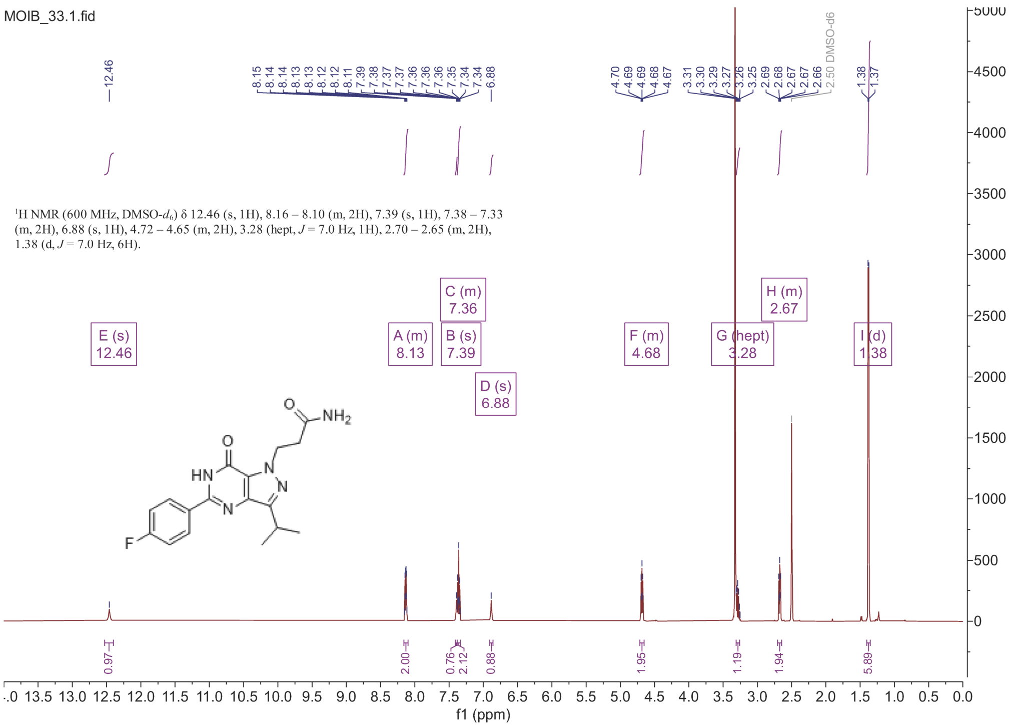

Figure S88. ^{1}H NMR spectrum of compound **21f**.

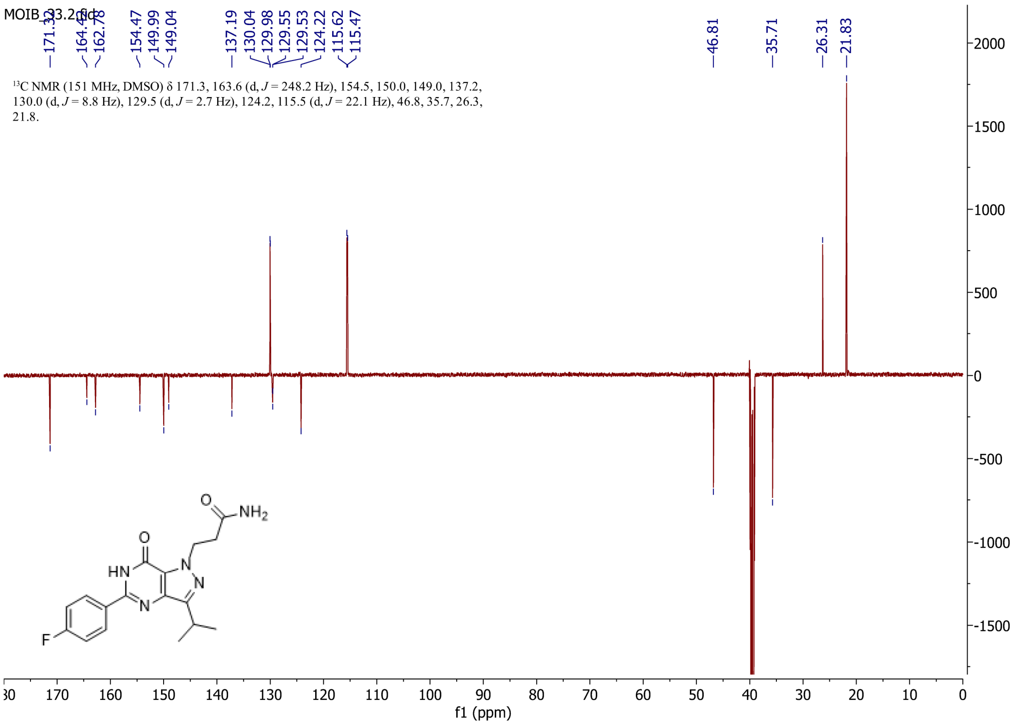

Figure S89. ^{13}C NMR spectrum of compound **21f**.

Acquired by	: Admin
Date Acquired	: 11/7/2019 11:19:41 AM
Sample Name	: MOIB_36_final
Sample ID	:
Tray#	: 1
Vial#	: 12
Injection Volume	: 3
Data File	: C:\LabSolutions\Data\2019\2019-wk28\MOIB_36_final.lcd
Background File	: blanco 11072019.lcd
Method File	: Method SCAN ACID standard.lcm
Report Format	: DefaultLCMS.lcr
Tuning File	: C:\LabSolutions\Tuning\Tuning-ESI-pos-neg01072015a.lct
Processed by	: Admin
Modified Date	: 31/3/2021 10:51:15 AM

PDA Graph

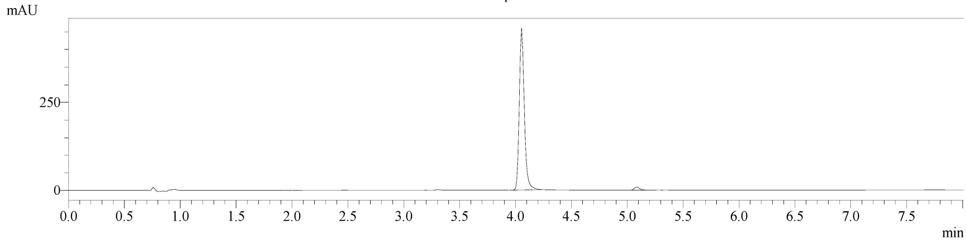

PeakTable

PDA Ch1 254nm 4nm

Peak#	Name	Ret. Time	Area	Area %
1		4.048	1436731	97.987
2		5.080	29518	2.013

MS Chromatogram

MOIB_36_final C:\LabSolutions\Data\2019\2019-wk21-30\2019-wk28\MOIB_36_final.lcd

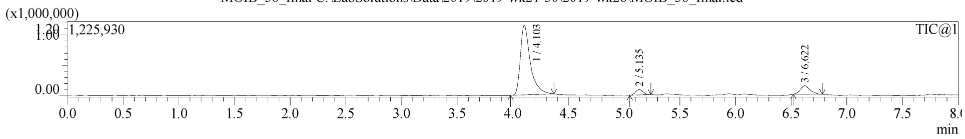

MS Spectrum Graph

#:1 Ret.Time:Averaged 4.090-4.110(Scan#:410-412)
BG Mode:Calc 3.980<->4.370(399<->438)
Mass Peaks:7 Base Peak:317.10(916094) Polarity:Pos Segment1 - Event1

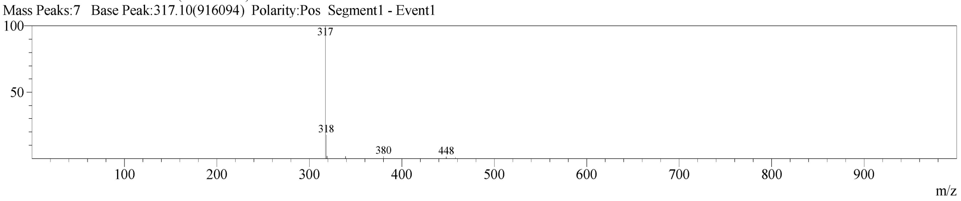

MS Spectrum Table

#:1 Ret.Time:
BG Mode:Calc 3.980<->4.370(399<->438)
Mass Peaks:7 Base Peak:317.10(916094) Polarity:Pos Segment1 - Event1

#	m/z	Abs.Inten.	Rel.Inten.	Charge	Polarity	Monoisotopic
1	317.10	916094	100.00			
2	318.10	162880	17.78			
3	319.05	17836	1.95			
4	339.10	16003	1.75			
5	380.20	17058	1.86			
6	448.00	13229	1.44			
7	458.00	9755	1.06			

Figure S90. LCMS spectrum of compound **22**.

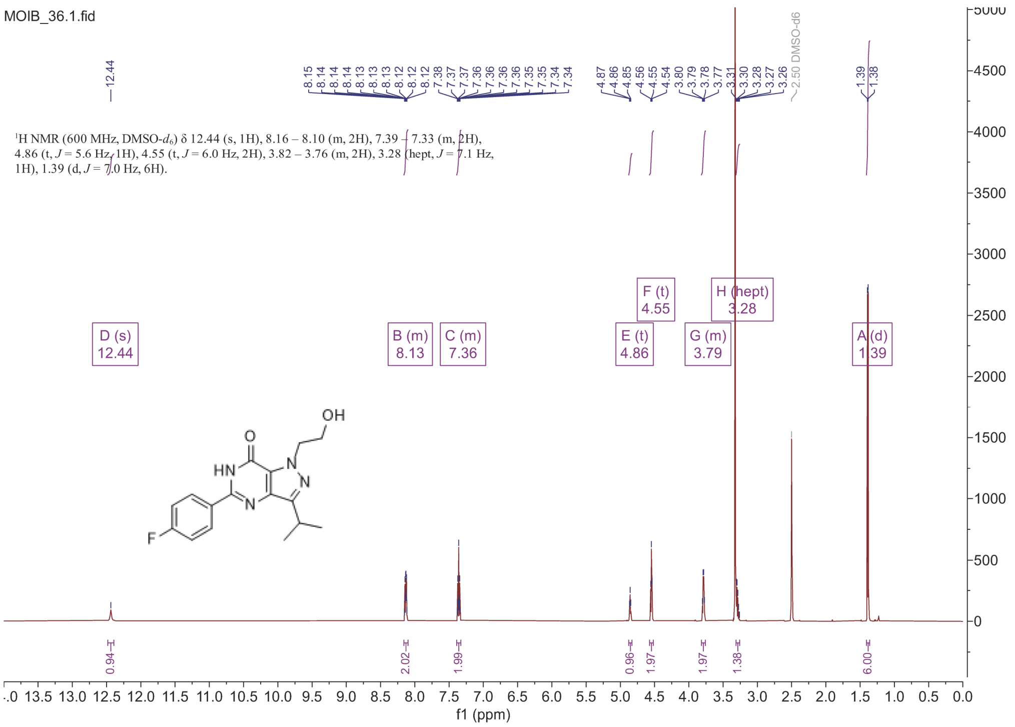

Figure S91. ¹H NMR spectrum of compound **22**.

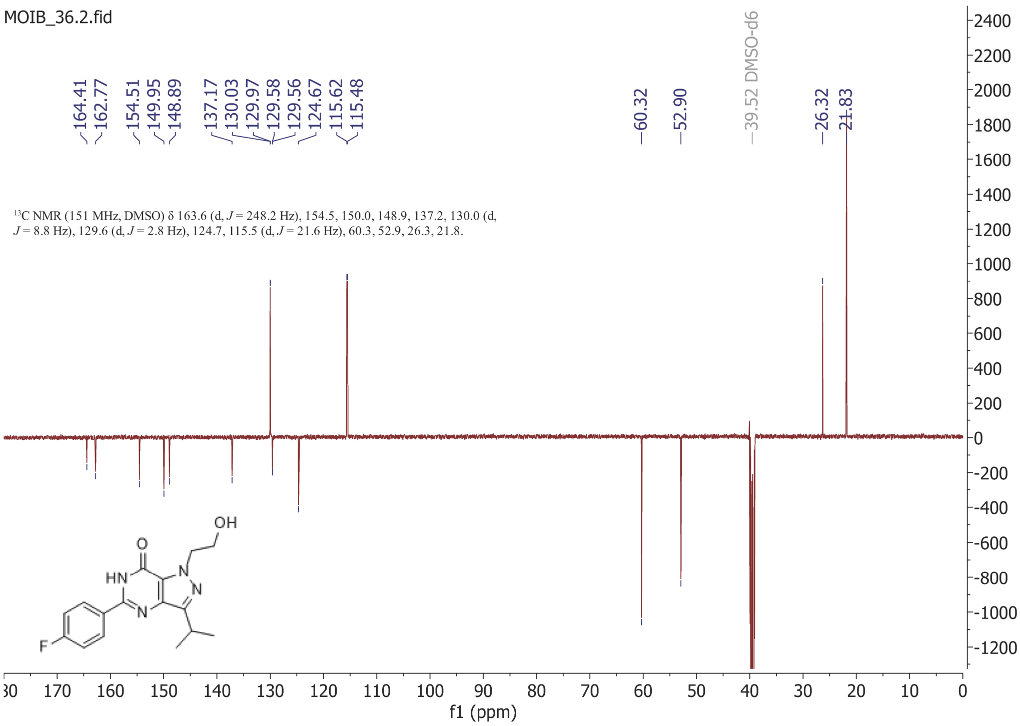

Figure S92. ¹³C NMR spectrum of compound **22**.

Acquired by	: Admin
Date Acquired	: 29/5/2019 12:37:27 PM
Sample Name	: MOIB_18_pure
Sample ID	:
Tray#	: 1
Vial#	: 21
Injection Volume	: 1
Data File	: C:\LabSolutions\Data\2019\2019-wk22\MOIB_18_pure.lcd
Background File	: blanco 29052019.lcd
Method File	: Method SCAN ACID standard.lcm
Report Format	: DefaultLCMS.lcr
Tuning File	: C:\LabSolutions\Tuning\Tuning-ESI-pos-neg01072015a.lct
Processed by	: Admin
Modified Date	: 29/5/2019 1:03:53 PM

Chromatogram
MOIB_18_pure C:\LabSolutions\Data\2019\2019-wk22\MOIB_18_pure.lcd

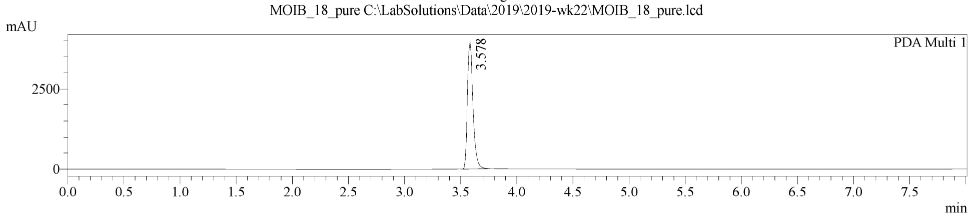

1 PDA Multi 1 / 254nm 4nm

PeakTabl

PDA Ch1 254nm 4nm

Peak#	Name	Ret. Time	Area	Area %
1		3.578	13616793	100.000

MS Chromatogram
MOIB_18_pure C:\LabSolutions\Data\2019\2019-wk22\MOIB_18_pure.lcd

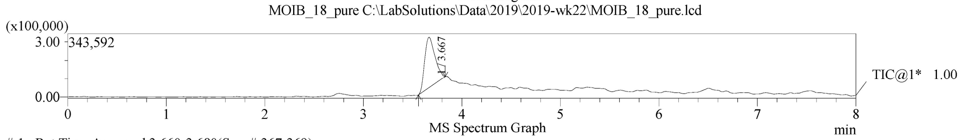

MS Spectrum Graph

#:1 Ret.Time:Averaged 3.660-3.680(Scan#:367-369)
Mass Peaks:9 Base Peak:214.00(190889) Polarity:Pos Segment1 - Event1

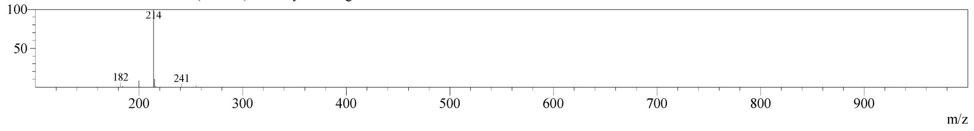

MS Spectrum Table

#:1 Ret.Time:
BG Mode:Calc 3.560<->3.830(357<->384)
Mass Peaks:9 Base Peak:214.00(190889) Polarity:Pos Segment1 - Event1

#	m/z	Abs.Inten.	Rel.Inten.	Charge	Polarity	Monoisotopic
1	181.95	10119	5.30			
2	184.10	2766	1.45			
3	199.95	16007	8.39			
4	201.15	2044	1.07			
5	214.00	190889	100.00			
6	215.00	19639	10.29			
7	215.90	2171	1.14			
8	241.00	6945	3.64			
9	255.15	4946	2.59			

Figure S93. LCMS spectrum of compound **23**.

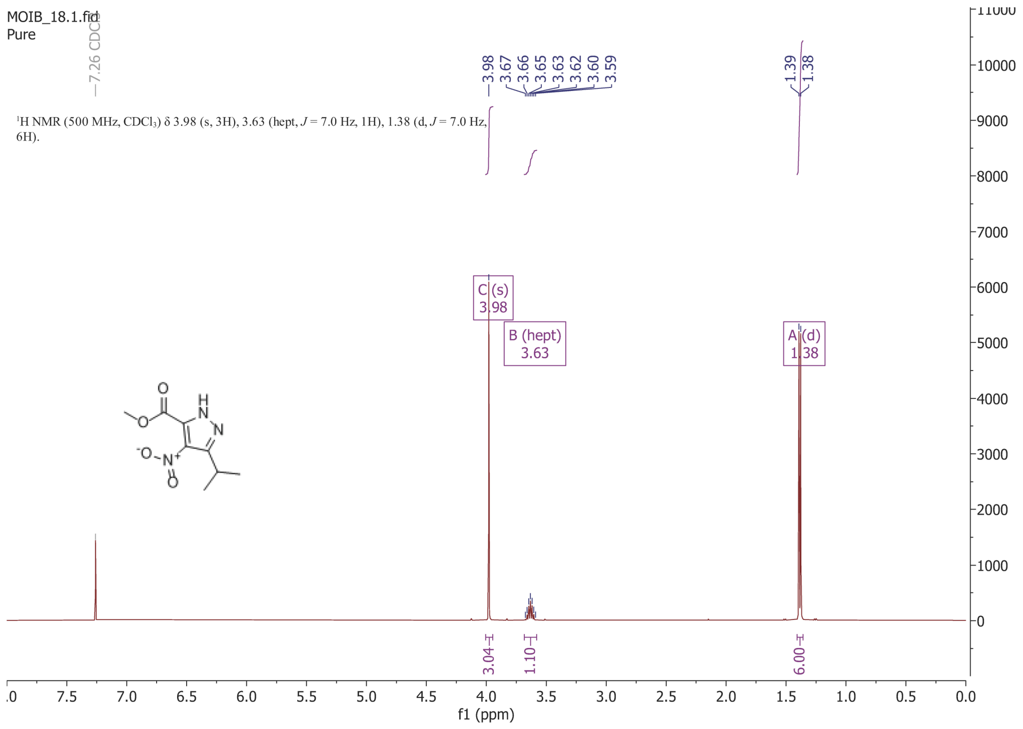

Figure S94. ^{1}H NMR spectrum of compound **23**.

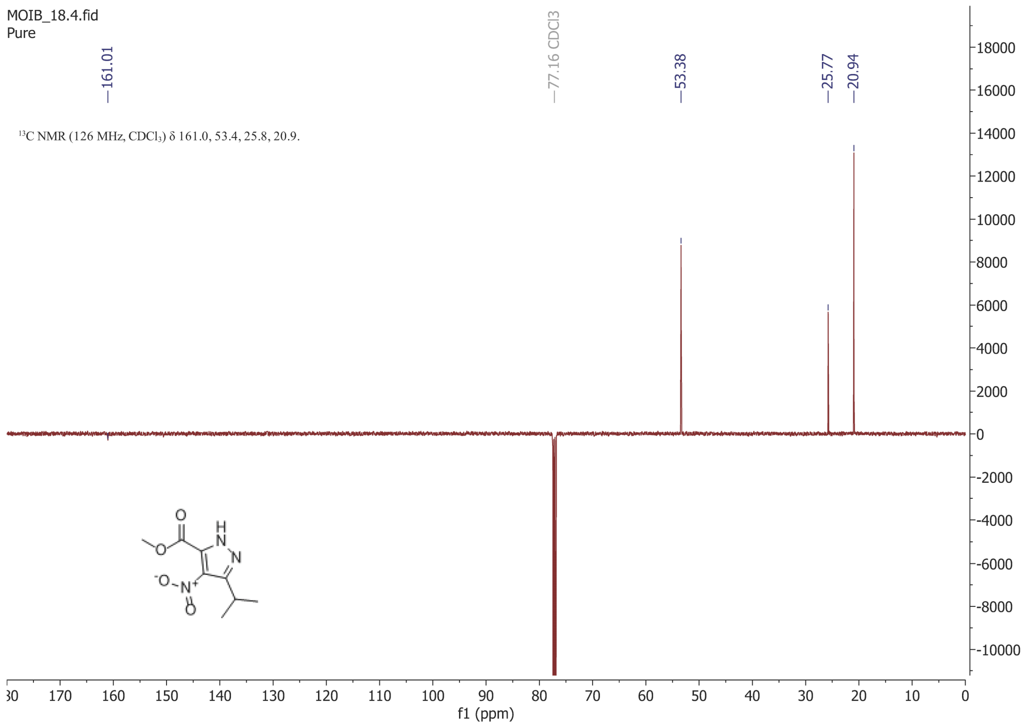

Figure S95. ^{13}C NMR spectrum of compound **23**.

Acquired by	: Admin
Date Acquired	: 16/7/2018 10:51:14 AM
Sample Name	: LABA-022
Sample ID	:
Tray#	: 1
Vial#	: 9
Injection Volume	: 1
Data File	: C:\LabSolutions\Data\2018\2018-wk29\LABA-022.lcd
Background File	: blanco 16072018.lcd
Method File	: Method SCAN ACID standard neg.lcm
Report Format	: DefaultLCMS.lcr
Tuning File	: C:\LabSolutions\Tuning\Tuning-ESI-pos-neg01072015a.lct
Processed by	: Admin
Modified Date	: 16/7/2018 11:08:48 AM

Chromatogram
LABA-022 C:\LabSolutions\Data\2018\2018-wk29\LABA-022.lcd

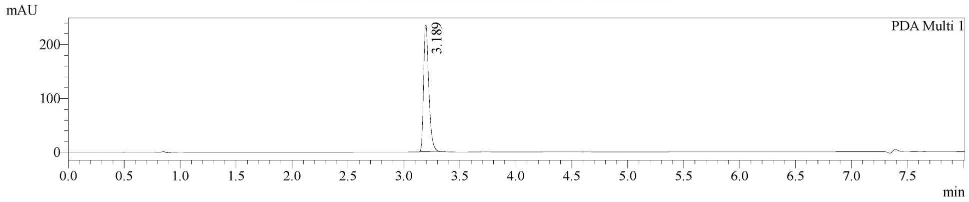

1 PDA Multi 1 / 254nm 4nm

Peak#	Name	Ret. Time	Area	Area %
1		3.189	765106	100.000

MS Chromatogram
LABA-022 C:\LabSolutions\Data\2018\2018-wk29\LABA-022.lcd

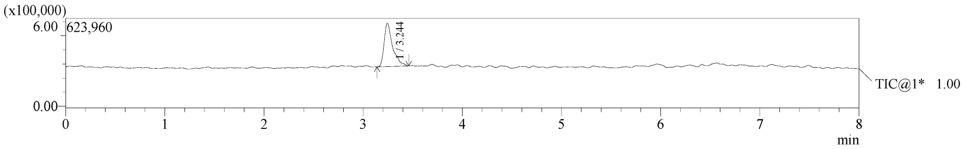

MS Spectrum Graph

#:1 Ret.Time:Averaged 3.230-3.250(Scan#:324-326)
Mass Peaks:24 Base Peak:179.00(141779) Polarity:Neg Segment1 - Event1

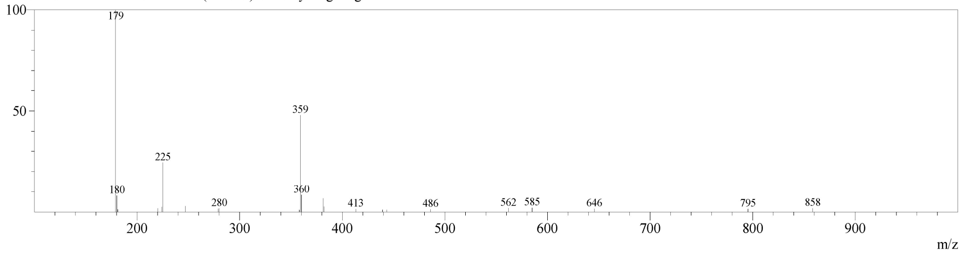

MS Spectrum Table

#:1 Ret.Time:
BG Mode:Calc 3.140<->3.460(315<->347)
Mass Peaks:24 Base Peak:179.00(141779) Polarity:Neg Segment1 - Event1

#	m/z	Abs.Inten.	Rel.Inten.	Charge	Polarity	Monoisotopic
1	179.00	141779	100.00			
2	180.25	11417	8.05			
3	181.20	1755	1.24			
4	220.15	2520	1.78			
5	224.15	3579	2.52			
6	225.05	34564	24.38			
7	247.05	4057	2.86			
8	279.10	2230	1.57			
9	280.00	2476	1.75			
10	358.10	1454	1.03			
11	359.15	67854	47.86			
12	360.15	11941	8.42			
13	381.25	9413	6.64			
14	382.20	3611	2.55			
15	413.10	2066	1.46			
16	439.15	1523	1.07			
17	443.25	1420	1.00			
18	486.00	1824	1.29			
19	561.95	2607	1.84			
20	585.05	2845	2.01			
21	645.70	2233	1.57			
22	795.40	2152	1.52			
23	858.30	2564	1.81			
24	1051.00	2181	1.54			

Figure S96. LCMS spectrum of compound **27a**.

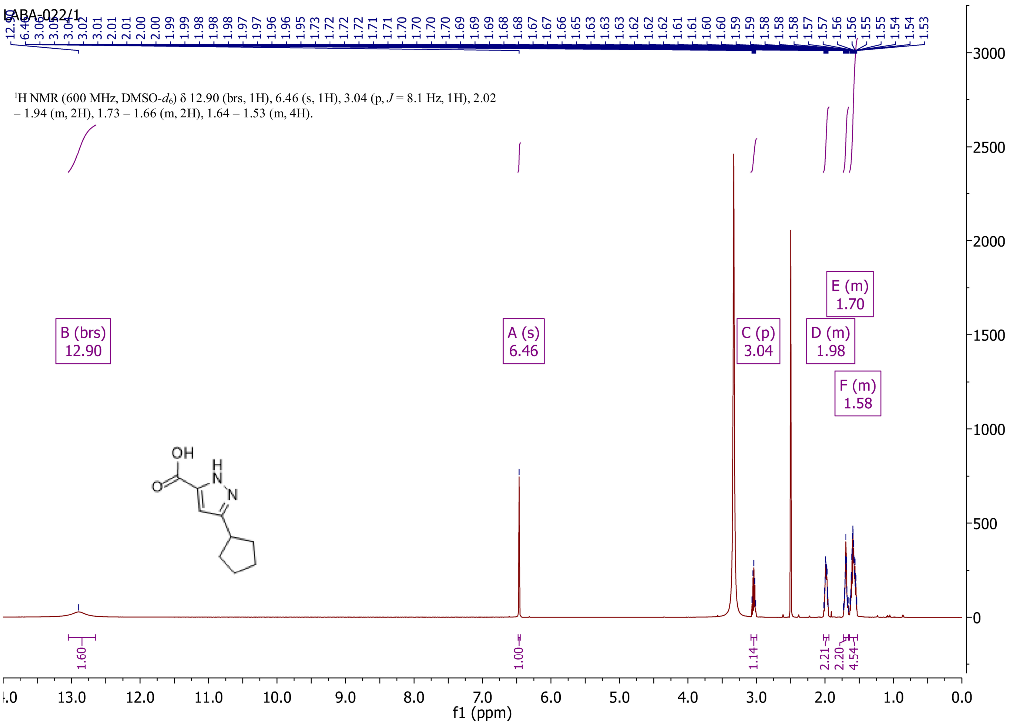

Figure S97. ^{1}H NMR spectrum of compound **27a**.

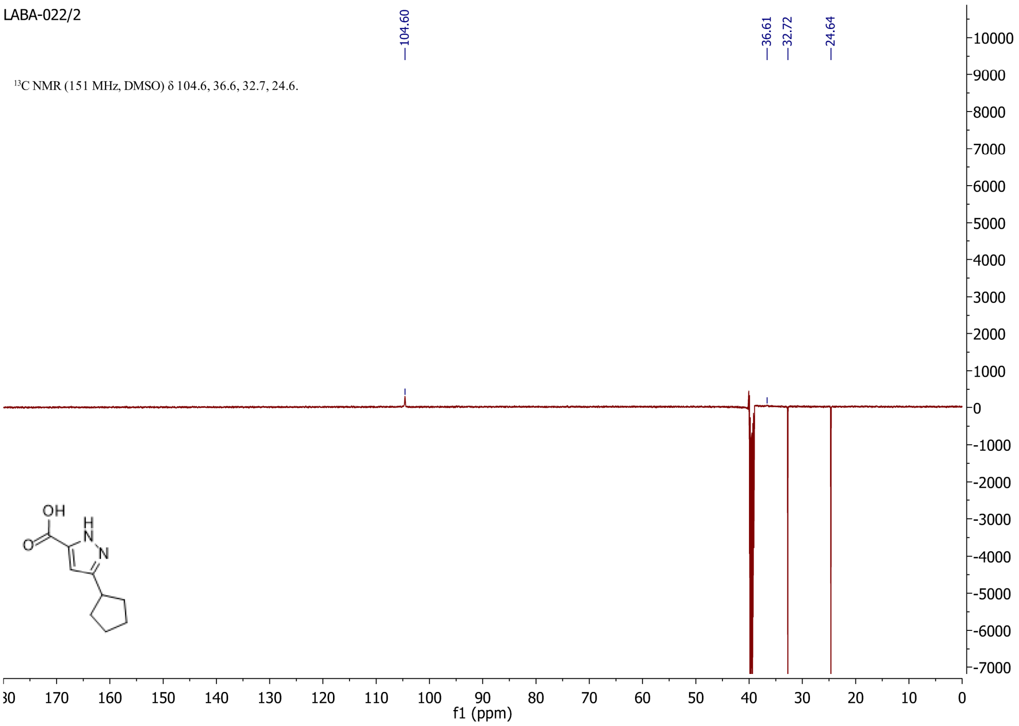

Figure S98. ^{13}C NMR spectrum of compound **27a**.

Acquired by	: Admin
Date Acquired	: 23/10/2018 2:30:32 PM
Sample Name	: ODVI01_018 crude 231018
Sample ID	:
Tray#	: 1
Vial#	: 17
Injection Volume	: 1
Data File	: C:\LabSolutions\Data\2018\2018-wk43\ODVI01_018 crude 231018.lcd
Background File	: 23102018_blanco.lcd
Method File	: Method SCAN ACID standard.lcm
Report Format	: DefaultLCMS.lcr
Tuning File	: C:\LabSolutions\Tuning\Tuning-ESI-pos-neg01072015a.lct
Processed by	: Admin
Modified Date	: 23/10/2018 2:40:02 PM

Chromatogram
ODVI01_018 crude 231018 C:\LabSolutions\Data\2018\2018-wk43\ODVI01_018 crude 231018.lcd

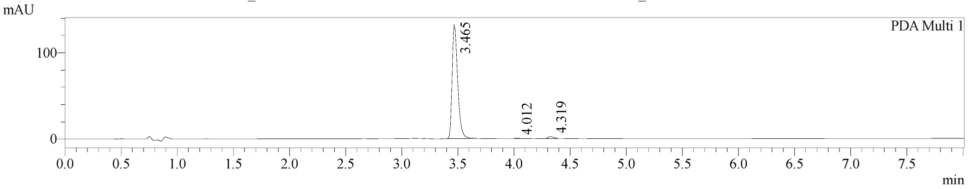

1 PDA Multi 1 / 254nm 4nm

Peak#	Name	Ret. Time	Area	Area %
1		3.465	452342	97.867
2		4.012	1072	0.232
3		4.319	8786	1.901

MS Chromatogram
ODVI01_018 crude 231018 C:\LabSolutions\Data\2018\2018-wk43\ODVI01_018 crude 231018.lcd

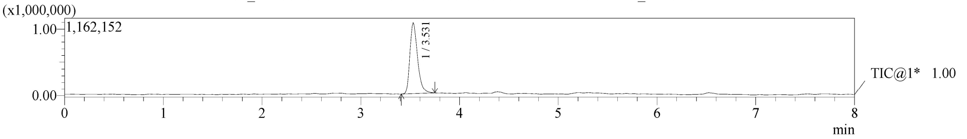

MS Spectrum Graph

#:1 Ret.Time:Averaged 3.520-3.540(Scan#:353-355)
Mass Peaks:8 Base Peak:195.05(585623) Polarity:Pos Segment1 - Event1

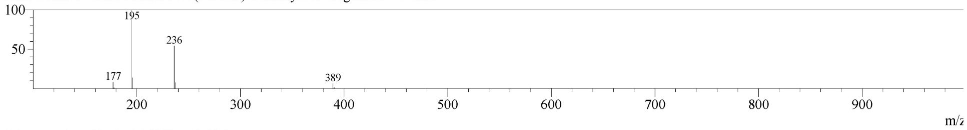

#:2 Ret.Time:Single 4.040(Scan#:405)
Mass Peaks:15 Base Peak:258.10(9188) Polarity:Pos Segment1 - Event1

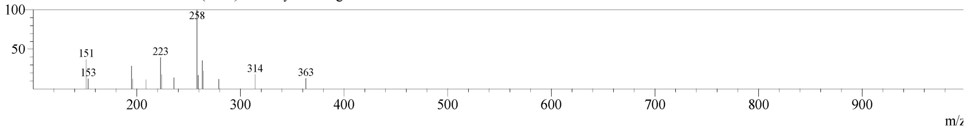

#:3 Ret.Time:Single 4.340(Scan#:435)
Mass Peaks:18 Base Peak:258.15(7349) Polarity:Pos Segment1 - Event1

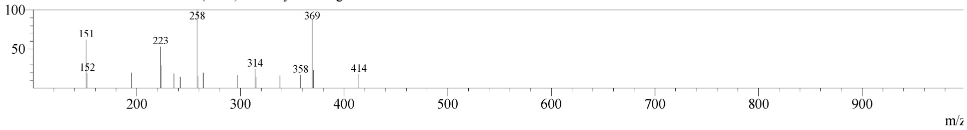

MS Spectrum Table

#:1 Ret.Time:
BG Mode:Calc 3.410<->3.750(342<->376)
Mass Peaks:8 Base Peak:195.05(585623) Polarity:Pos Segment1 - Event1

#	m/z	Abs.Inten.	Rel.Inten.	Charge	Polarity	Monoisotopic
1	177.05	47070	8.04			
2	178.05	6493	1.11			
3	195.05	585623	100.00			
4	196.05	79866	13.64			
5	236.05	317612	54.23			
6	237.10	42704	7.29			
7	389.25	35464	6.06			
8	390.30	8125	1.39			

Figure S99. LCMS spectrum of compound **27b**.

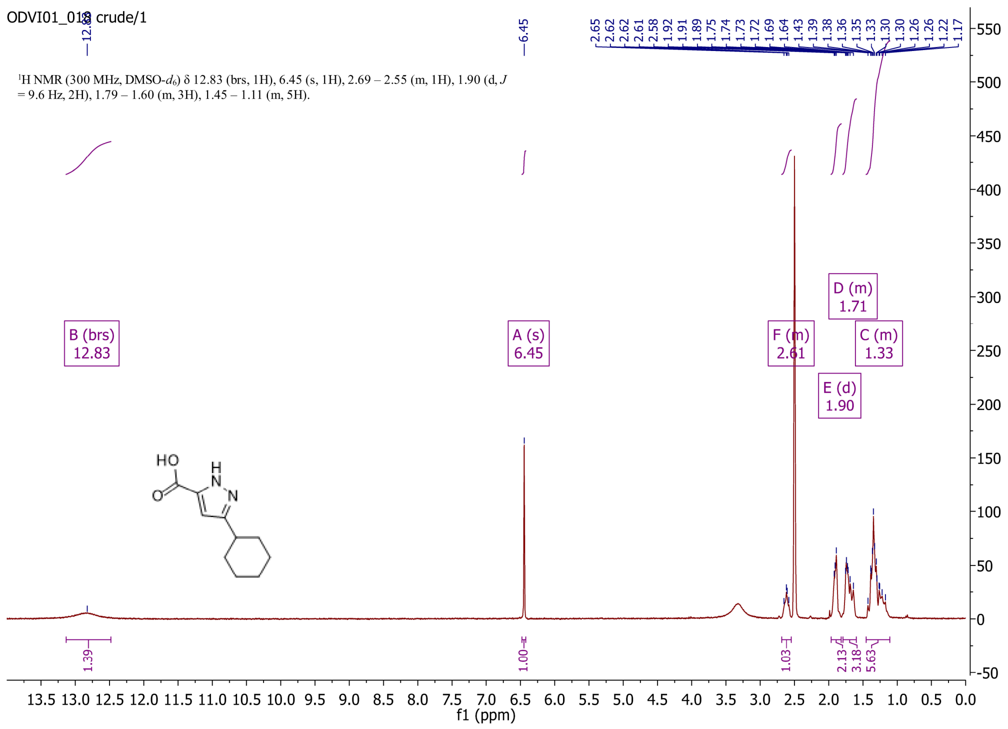

Figure S100. ^{1}H NMR spectrum of compound **27b**.

Acquired by	: Admin
Date Acquired	: 24/7/2018 10:42:48 AM
Sample Name	: LABA-026-CRUDE-PR
Sample ID	:
Tray#	: 1
Vial#	: 15
Injection Volume	: 1
Data File	: C:\LabSolutions\Data\2018\2018-wk30\LABA-026-CRUDE-PR.lcd
Background File	: blanco 24072018.lcd
Method File	: Method SCAN ACID standard neg.lcm
Report Format	: DefaultLCMS.lcr
Tuning File	: C:\LabSolutions\Tuning\Tuning-ESI-pos-neg01072015a.lct
Processed by	: Admin
Modified Date	: 24/7/2018 12:11:18 PM

Chromatogram

LABA-026-CRUDE-PR C:\LabSolutions\Data\2018\2018-wk30\LABA-026-CRUDE-PR.lcd

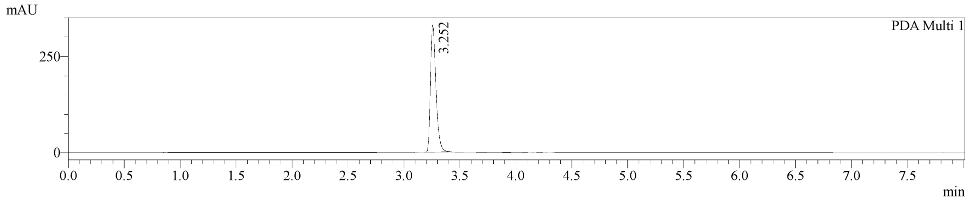

1 PDA Multi 1 / 254nm 4nm

Peak#	Name	Ret. Time	Area	Area %
1		3.252	1116635	100.000

MS Chromatogram

LABA-026-CRUDE-PR C:\LabSolutions\Data\2018\2018-wk30\LABA-026-CRUDE-PR.lcd

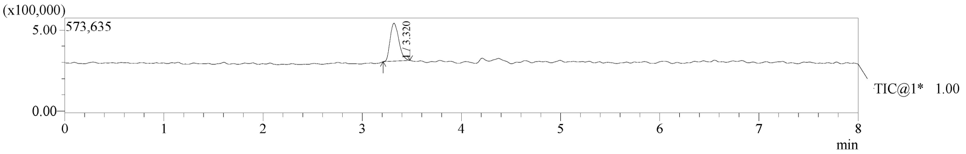

MS Spectrum Graph

#:1 Ret.Time:Averaged 3.310-3.330(Scan#:332-334)

Mass Peaks:14 Base Peak:224.05(105117) Polarity:Neg Segment1 - Event1

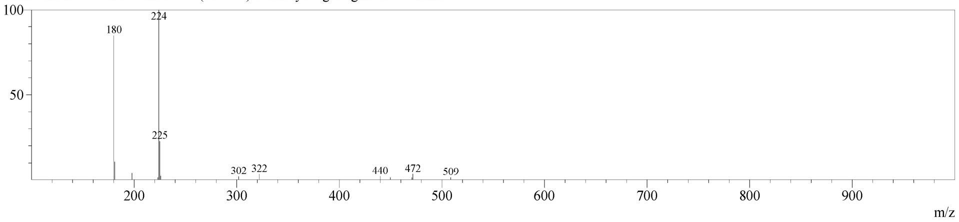

MS Spectrum Table

#:1 Ret.Time:

BG Mode:Calc 3.210<->3.480(322<->349)

Mass Peaks:14 Base Peak:224.05(105117) Polarity:Neg Segment1 - Event1

#	m/z	Abs.Inten.	Rel.Inten.	Charge	Polarity	Monoisotopic
1	180.05	89072	84.74			
2	181.05	11197	10.65			
3	197.95	3988	3.79			
4	223.10	1357	1.29			
5	224.05	105117	100.00			
6	225.05	23822	22.66			
7	225.95	2303	2.19			
8	301.85	1934	1.84			
9	321.95	3422	3.26			
10	439.75	1971	1.88			
11	449.70	1347	1.28			
12	470.70	1270	1.21			
13	471.70	3498	3.33			
14	508.60	1327	1.26			

Figure S101. LCMS spectrum of compound **28a**.

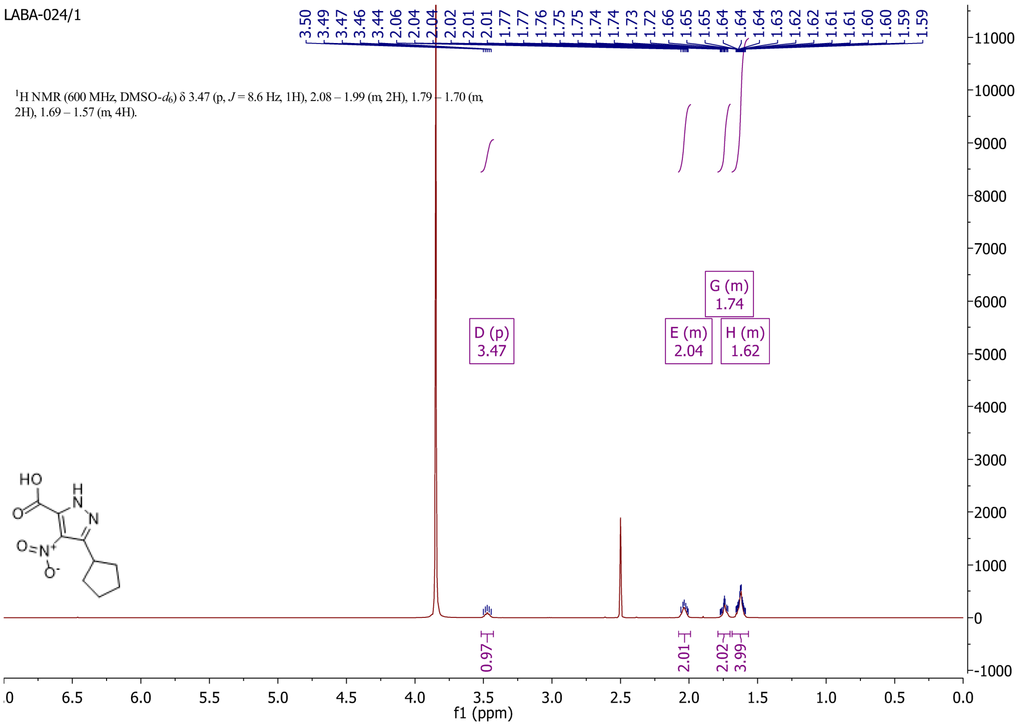

Figure S102. ^{1}H NMR spectrum of compound **28a**.

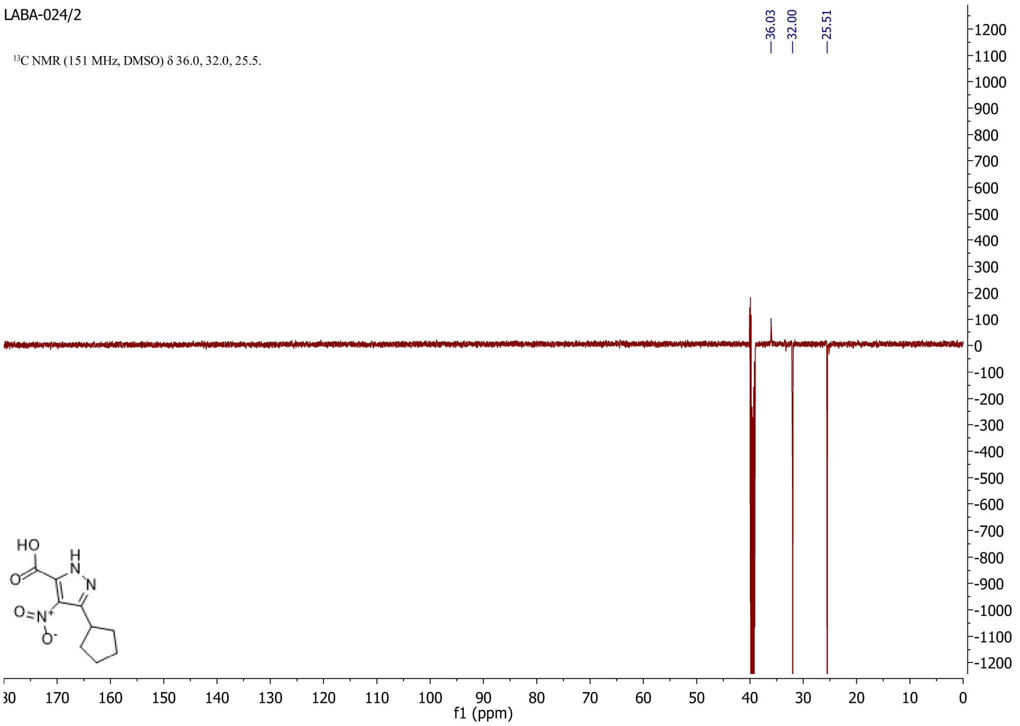

Figure S103. ^{13}C NMR spectrum of compound **28a**.

Acquired by	: Admin
Date Acquired	: 24/5/2018 12:30:40 PM
Sample Name	: YAZH01-126
Sample ID	:
Tray#	: 1
Vial#	: 12
Injection Volume	: 3
Data File	: C:\LabSolutions\Data\2018\2018-wk21\YAZH01-126.lcd
Background File	: blanco_24052018.lcd
Method File	: Method SCAN ACID standard.lcm
Report Format	: DefaultLCMS.lcr
Tuning File	: C:\LabSolutions\Tuning\Tuning-ESI-pos-neg01072015a.lct
Processed by	: Admin
Modified Date	: 24/5/2018 2:23:18 PM

Chromatogram
YAZH01-126 C:\LabSolutions\Data\2018\2018-wk21\YAZH01-126.lcd

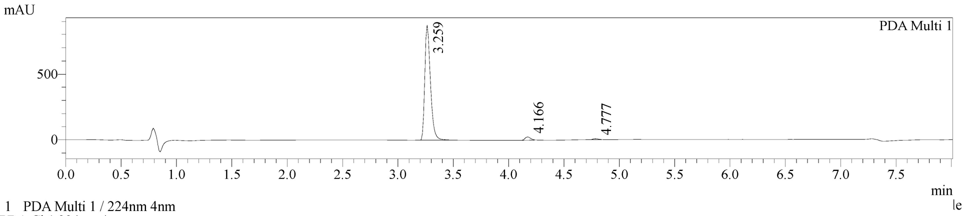

1 PDA Multi 1 / 224nm 4nm

PDA Ch1 224nm 4nm

Peak#	Name	Ret. Time	Area	Area %
1		3.259	3089630	96.427
2		4.166	87842	2.742
3		4.777	26639	0.831

MS Chromatogram
YAZH01-126 C:\LabSolutions\Data\2018\2018-wk21\YAZH01-126.lcd

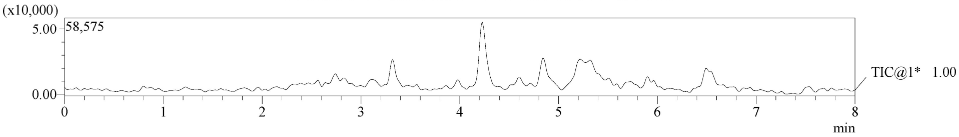

MS Spectrum Graph

#:1 Ret.Time:Single 3.310(Scan#:332)
Mass Peaks:14 Base Peak:214.00(9135) Polarity:Pos Segment1 - Event1

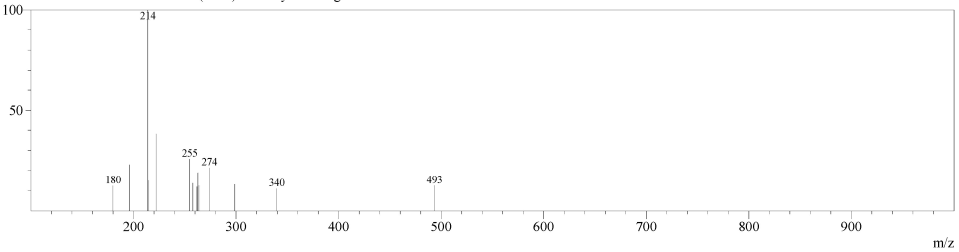

MS Spectrum Table

#:1 Ret.Time:
BG Mode:None
Mass Peaks:14 Base Peak:214.00(9135) Polarity:Pos Segment1 - Event1

#	m/z	Abs.Inten.	Rel.Inten.	Charge	Polarity	Monoisotopic
1	179.95	1133	12.40			
2	195.95	2092	22.90			
3	214.00	9135	100.00			
4	215.05	1374	15.04			
5	222.00	3478	38.07			
6	254.85	2347	25.69			
7	257.90	1253	13.72			

#	m/z	Abs.Inten.	Rel.Inten.	Charge	Polarity	Monoisotopic
8	261.85	1089	11.92			
9	262.85	1727	18.91			
10	263.85	1142	12.50			
11	273.85	1949	21.34			
12	298.80	1197	13.10			
13	339.75	1002	10.97			
14	493.50	1137	12.45			

Figure S104. LCMS spectrum of compound **28c**.

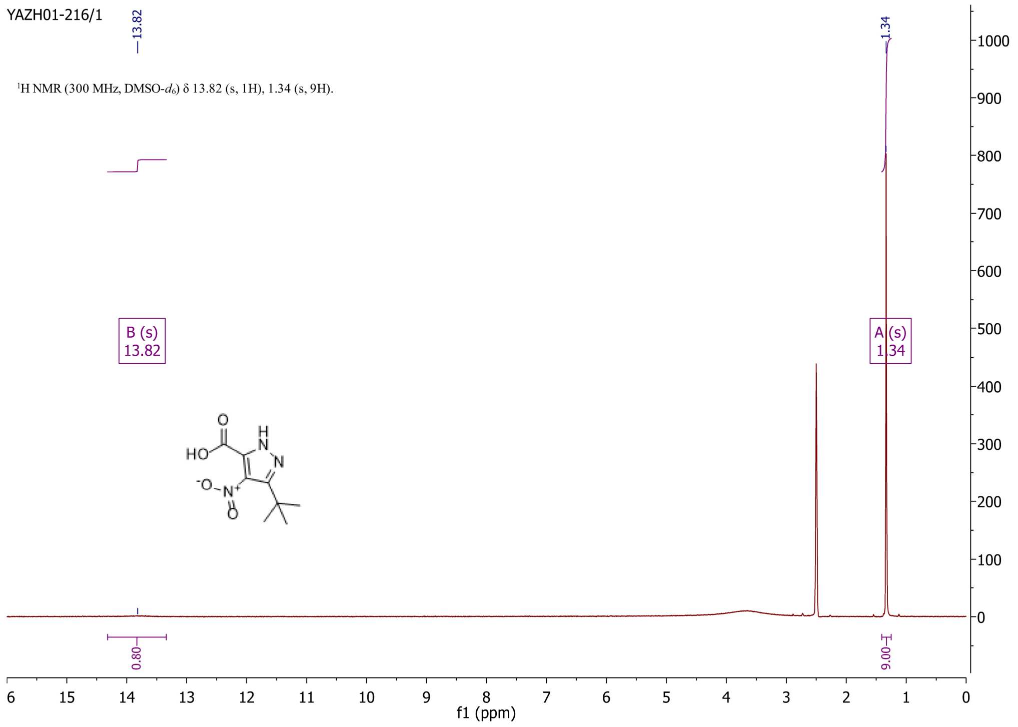

Figure S105. ^{1}H NMR spectrum of compound **28c**.

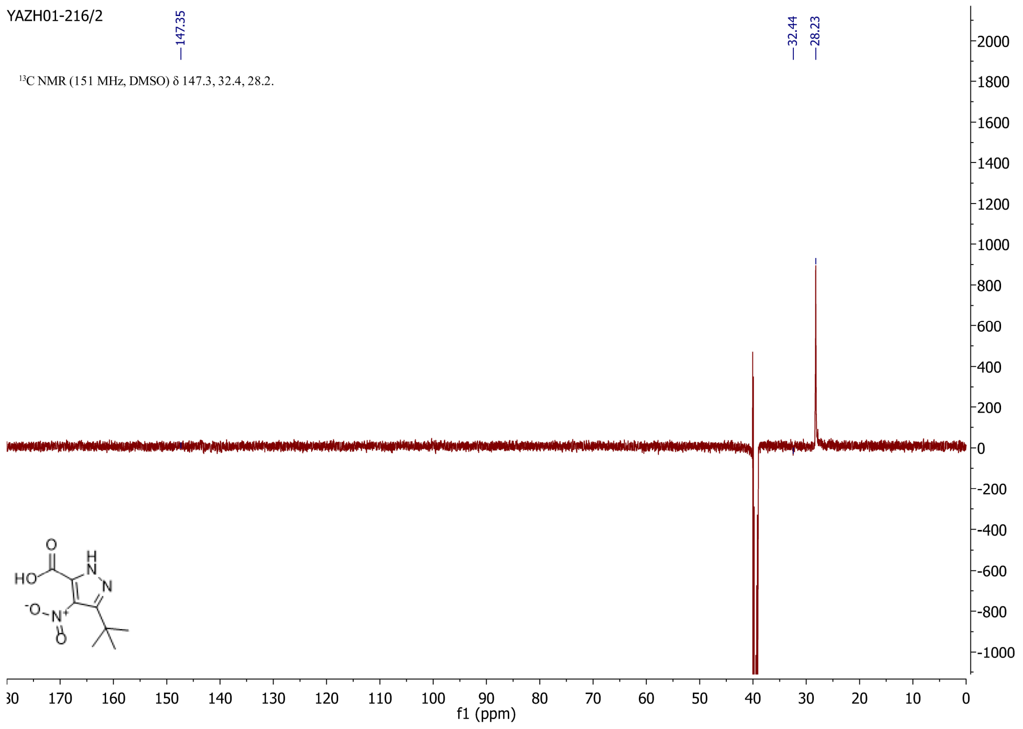

Figure S106. ^{13}C NMR spectrum of compound **28c**.

Acquired by	: Admin
Date Acquired	: 17/5/2018 4:33:53 PM
Sample Name	: YAZH01-215
Sample ID	:
Tray#	: 1
Vial#	: 12
Injection Volume	: 1
Data File	: C:\LabSolutions\Data\2018\2018-wk20\YAZH01-215-1.lcd
Background File	: blanco 170518.lcd
Method File	: Method SCAN ACID standard neg.lcm
Report Format	: DefaultLCMS.lcr
Tuning File	: C:\LabSolutions\Tuning\Tuning-ESI-pos-neg01072015a.lct
Processed by	: Admin
Modified Date	: 17/5/2018 6:33:28 PM

PDA Graph

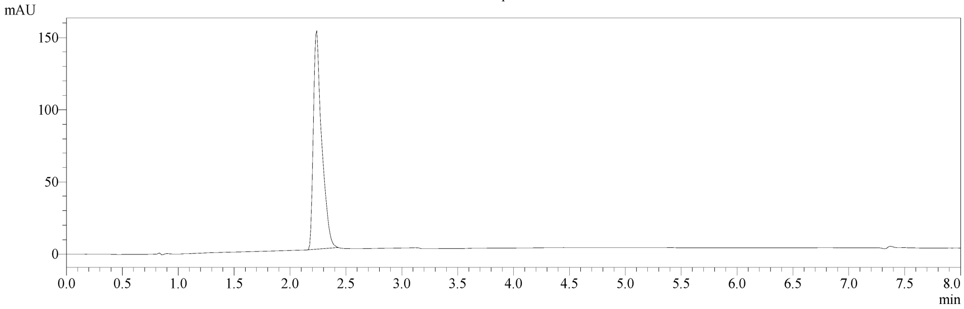

PDA Ch1 254nm 4nm

Peak#	Name	Ret. Time	Area	Area %
1		2.231	783773	100.000

MS Spectrum Graph

#:1 Ret.Time:Single 2.300(Scan#:231)
Mass Peaks:757 Base Peak:183.85(23845) Polarity:Neg Segment1 - Event1

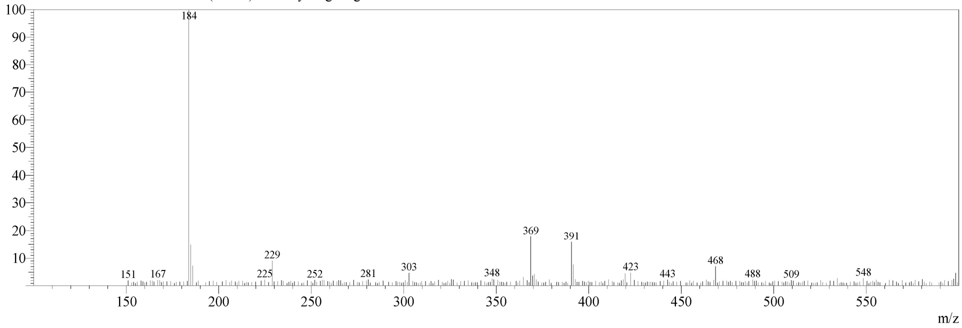

MS Spectrum Table

#:1 Ret.Time:
BG Mode:None
Mass Peaks:757 Base Peak:183.85(23845) Polarity:Neg Segment1 - Event1

#	m/z	Abs.Inten.	Rel.Inten.	Charge	Polarity	Monoisotopic
1	151.00	478	2.00			
2	153.00	288	1.21			
3	154.00	360	1.51			
4	155.00	258	1.08			
5	156.00	383	1.61			
6	158.00	460	1.93			
7	159.00	395	1.66			
8	160.00	305	1.28			
9	161.00	352	1.48			
10	163.00	487	2.04			
11	164.00	470	1.97			
12	165.00	368	1.54			
13	167.00	520	2.18			
14	168.00	501	2.10			
15	169.00	336	1.41			
16	170.00	401	1.68			
17	172.00	389	1.63			
18	174.00	423	1.77			
19	176.00	249	1.04			
20	177.00	363	1.52			
21	179.00	368	1.54			
22	181.00	404	1.69			
23	183.00	439	1.84			
24	183.85	23845	100.00			
25	184.90	3538	14.84			
26	185.90	1748	7.33			
27	187.90	313	1.31			
28	188.90	460	1.93			

Figure S107. LCMS spectrum of compound **28d**.

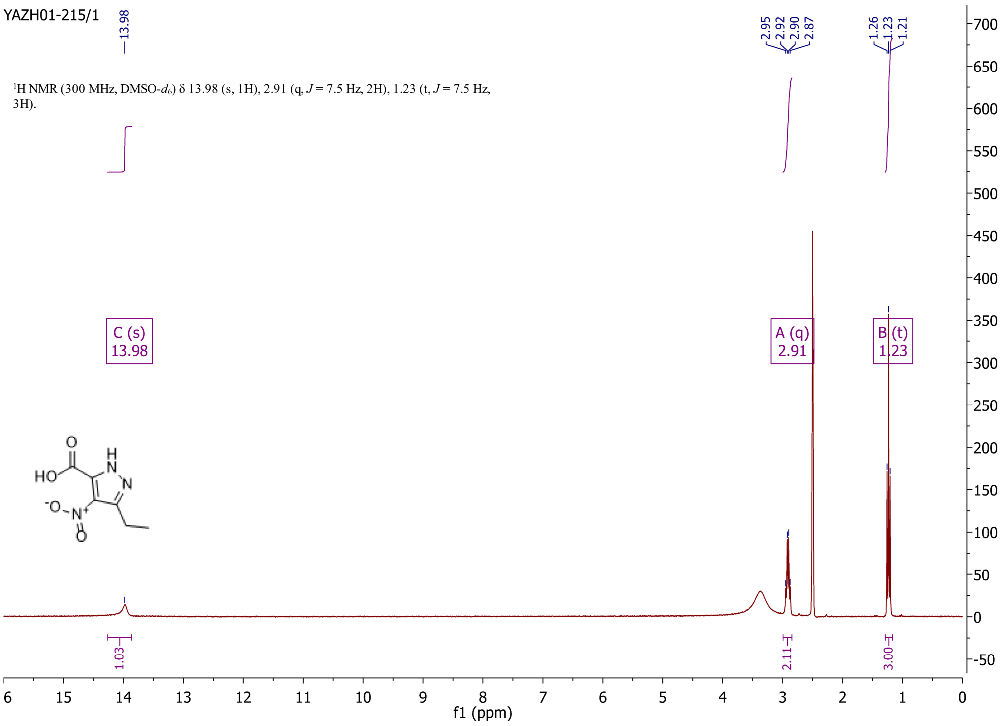

Figure S108. ^{1}H NMR spectrum of compound **28d**.

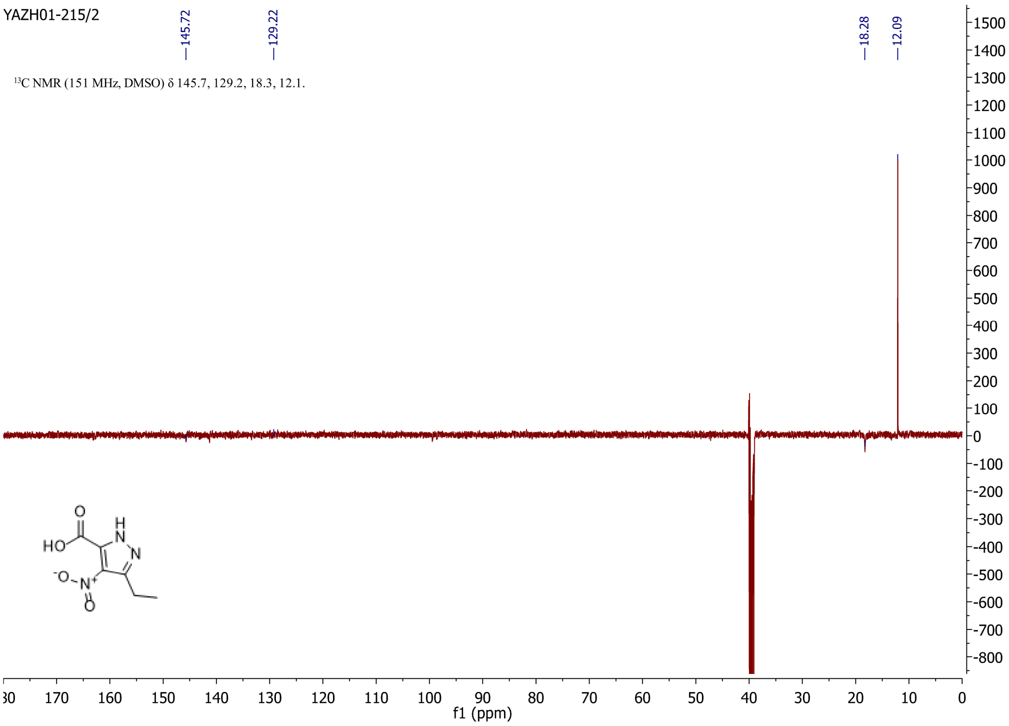

Figure S109. ^{13}C NMR spectrum of compound **28d**.

Acquired by	: Admin
Date Acquired	: 31/5/2018 4:42:22 PM
Sample Name	: YAZH01-217-20180531
Sample ID	:
Tray#	: 1
Vial#	: 10
Injection Volume	: 10
Data File	: C:\LabSolutions\Data\2018\2018-wk22\YAZH01-217-20180531.lcd
Background File	: blanco 310518.lcd
Method File	: Method SCAN ACID standard MW50.lcm
Report Format	: DefaultLCMS.lcr
Tuning File	: C:\LabSolutions\Tuning\Tuning-ESI-pos-neg01072015a.lct
Processed by	: Admin
Modified Date	: 1/6/2018 9:46:55 AM

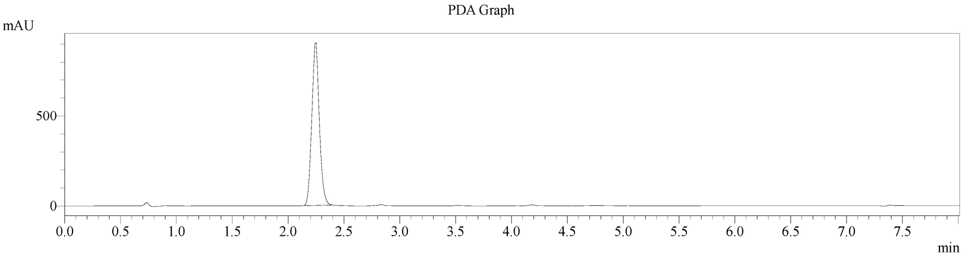

PeakTable

PDA Ch1 254nm 4nm

Peak#	Name	Ret. Time	Area	Area %
1		2.240	4229681	100.000

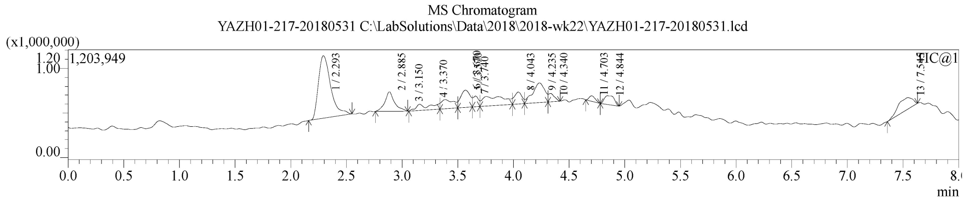

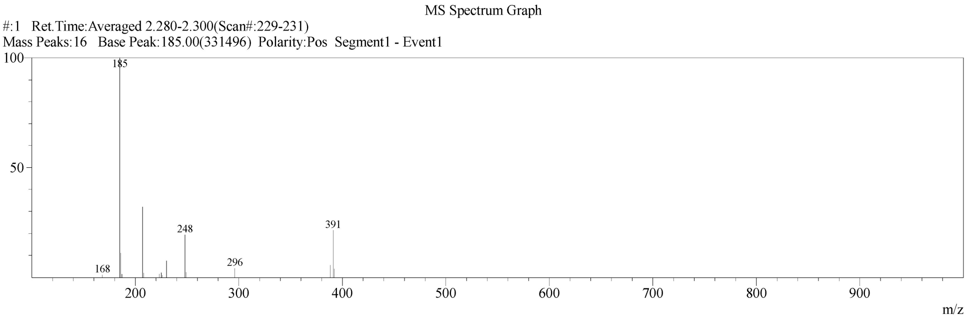

MS Spectrum Table

#:1 Ret.Time:
BG Mode:Calc 2.160<->2.550(217<->256)
Mass Peaks:16 Base Peak:185.00(331496) Polarity:Pos Segment1 - Event1

#	m/z	Abs.Inten.	Rel.Inten.	Charge	Polarity	Monoisotopic
1	168.00	4220	1.27			
2	185.00	331496	100.00			
3	186.00	36378	10.97			
4	187.05	5223	1.58			
5	206.95	106159	32.02			
6	208.00	6975	2.10			
7	223.15	5540	1.67			
8	225.05	7276	2.19			
9	226.05	3643	1.10			
10	230.15	25081	7.57			
11	248.05	64445	19.44			
12	249.00	7954	2.40			
13	296.00	13869	4.18			
14	388.40	18523	5.59			
15	391.10	71165	21.47			
16	392.00	13085	3.95			

Figure S110. LCMS spectrum of compound **29d**.

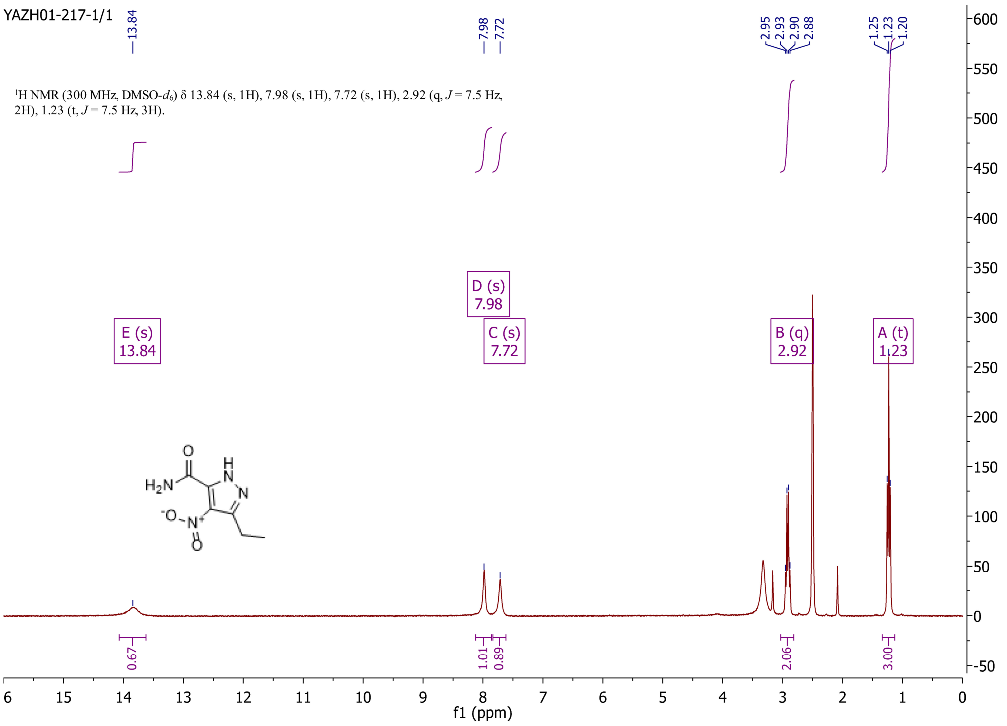

Figure S111. ^{1}H NMR spectrum of compound **29d**.

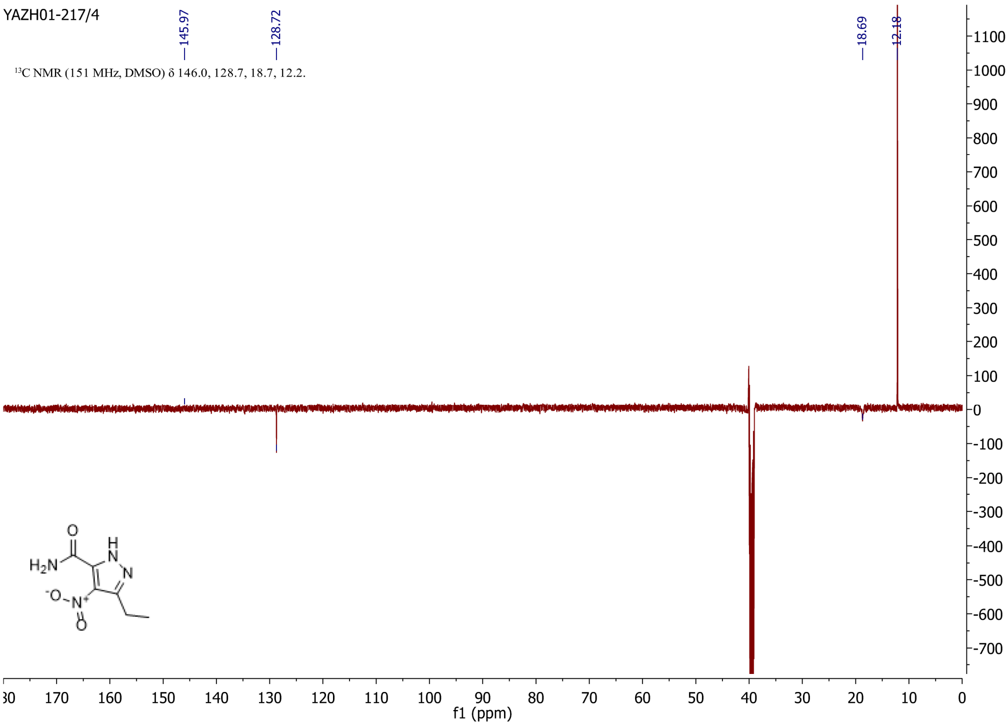

Figure S112. ^{13}C NMR spectrum of compound **29d**.

Acquired by	: Admin
Date Acquired	: 8/21/2017 3:12:01 PM
Sample Name	: YAZH01-134
Sample ID	:
Tray#	: 1
Vial#	: 40
Injection Volume	: 1
Data File	: C:\LabSolutions\Data\2017\2017-wk34\YAZH01-134.lcd
Background File	: BLANCO21082017.lcd
Method File	: Method SCAN ACID standard.lcm
Report Format	: DefaultLCMS.lcr
Tuning File	: C:\LabSolutions\Tuning\Tuning-ESI-pos-neg01072015a.lct
Processed by	: Admin
Modified Date	: 8/21/2017 3:40:37 PM

Chromatogram
YAZH01-134 C:\LabSolutions\Data\2017\2017-wk34\YAZH01-134.lcd

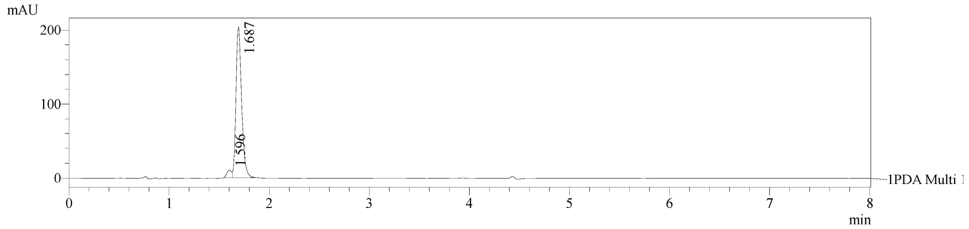

1 PDA Multi 1 / 254nm 4nm

Peak#	Name	Ret. Time	Area	Area %
1		1.596	36034	4.373
2		1.687	787999	95.627

MS Chromatogram
YAZH01-134 C:\LabSolutions\Data\2017\2017-wk34\YAZH01-134.lcd

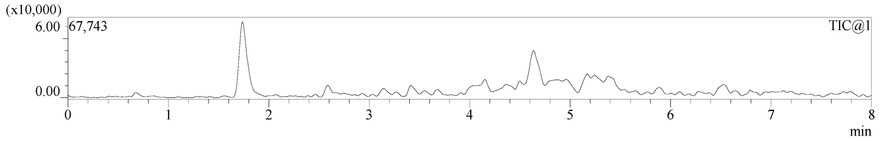

MS Spectrum Graph

#:1 Ret.Time:Single 1.750(Scan#:176)
Mass Peaks:12 Base Peak:171.00(20339) Polarity:Pos Segment1 - Event1

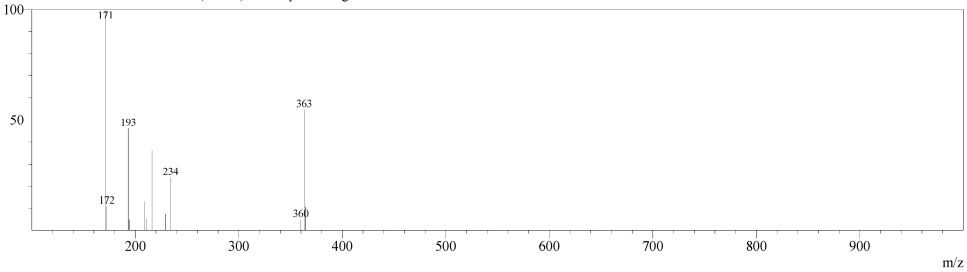

MS Spectrum Table

#:1 Ret.Time:
BG Mode:None
Mass Peaks:12 Base Peak:171.00(20339) Polarity:Pos Segment1 - Event1

#	m/z	Abs.Inten.	Rel.Inten.	Charge	Polarity	Monoisotopic
1	171.00	20339	100.00			
2	172.20	2238	11.00			
3	193.05	9397	46.20			
4	194.00	1006	4.95			
5	209.00	2641	12.98			
6	211.00	1085	5.33			
7	216.10	7347	36.12			
8	229.05	1522	7.48			
9	234.00	4866	23.92			
10	359.85	1012	4.98			
11	363.05	11123	54.69			
12	364.10	2179	10.71			

Figure S113. LCMS spectrum of compound **29e**.

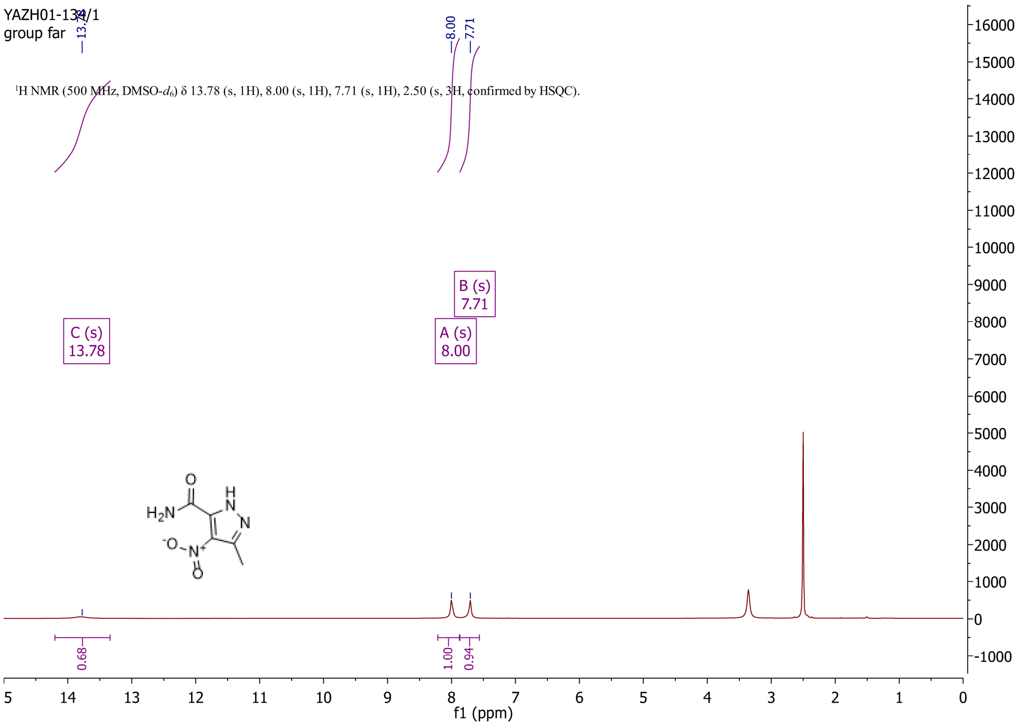

Figure S114. ^{1}H NMR spectrum of compound **29e**.

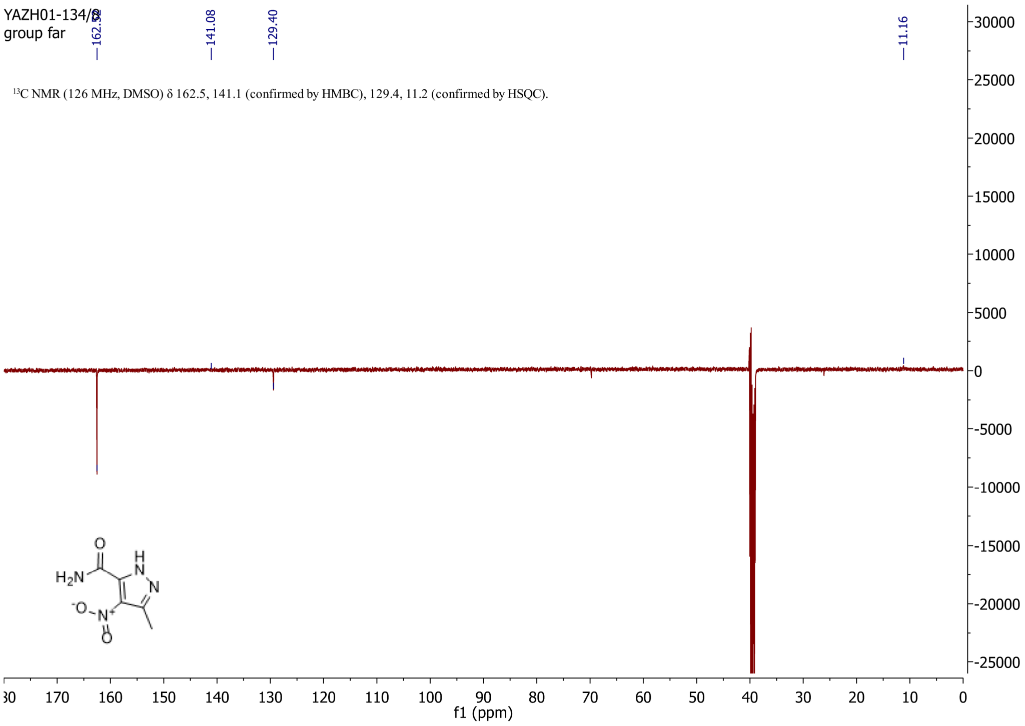

Figure S115. ^{13}C NMR spectrum of compound **29e**.

Acquired by	: Admin
Date Acquired	: 8/25/2017 11:08:22 AM
Sample Name	: YAZH01-137-1
Sample ID	:
Tray#	: 1
Vial#	: 12
Injection Volume	: 10
Data File	: C:\LabSolutions\Data\2017\2017-wk34\YAZH01-137-1.lcd
Background File	: BLANCO25082017.lcd
Method File	: Method SCAN ACID standard MW50.lcm
Report Format	: DefaultLCMS.lcr
Tuning File	: C:\LabSolutions\Tuning\Tuning-ESI-pos-neg01072015a.lct
Processed by	: Admin
Modified Date	: 8/25/2017 12:14:03 PM

Chromatogram
YAZH01-137-1 C:\LabSolutions\Data\2017\2017-wk34\YAZH01-137-1.lcd

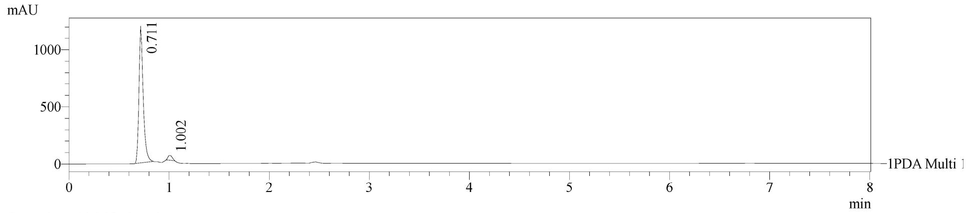

1 PDA Multi 1 / 254nm 4nm

PeakTable

PDA Ch1 254nm 4nm

Peak#	Name	Ret. Time	Area	Area %
1		0.711	3474924	96.648
2		1.002	120517	3.352

MS Chromatogram
YAZH01-137-1 C:\LabSolutions\Data\2017\2017-wk34\YAZH01-137-1.lcd

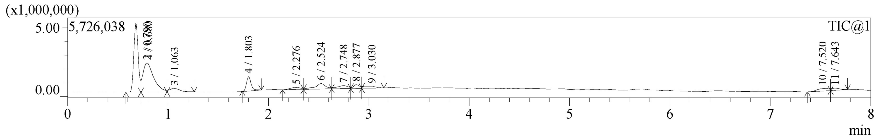

MS Spectrum Graph

#:1 Ret.Time:Averaged 0.780-0.800(Scan#:79-81)
Mass Peaks:32 Base Peak:141.00(571800) Polarity:Pos Segment1 - Event1

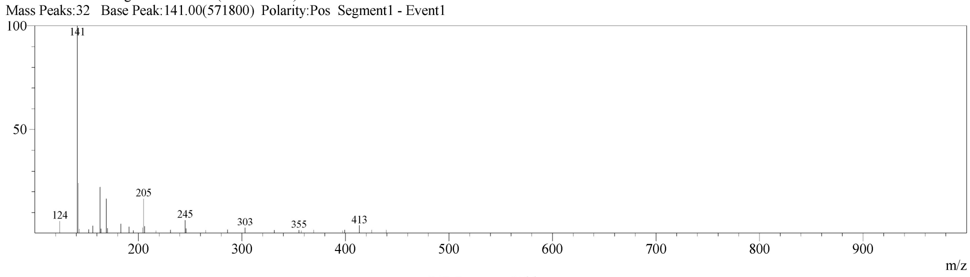

MS Spectrum Table

#:1 Ret.Time:
BG Mode:Calc 0.730<->0.990(74<->100)
Mass Peaks:32 Base Peak:141.00(571800) Polarity:Pos Segment1 - Event1

#	m/z	Abs.Inten.	Rel.Inten.	Charge	Polarity	Monoisotopic
1	124.00	33633	5.88			
2	141.00	571800	100.00			
3	142.00	137717	24.08			
4	143.00	10576	1.85			
5	152.10	9772	1.71			
6	156.00	19768	3.46			
7	163.00	127066	22.22			
8	163.95	11210	1.96			
9	169.05	94928	16.60			
10	170.10	12503	2.19			
11	183.10	25925	4.53			
12	191.05	17087	2.99			
13	195.15	6916	1.21			
14	204.05	14362	2.51			
15	205.05	94767	16.57			
16	206.05	17858	3.12			

Figure S116. LCMS spectrum of compound **30e**.

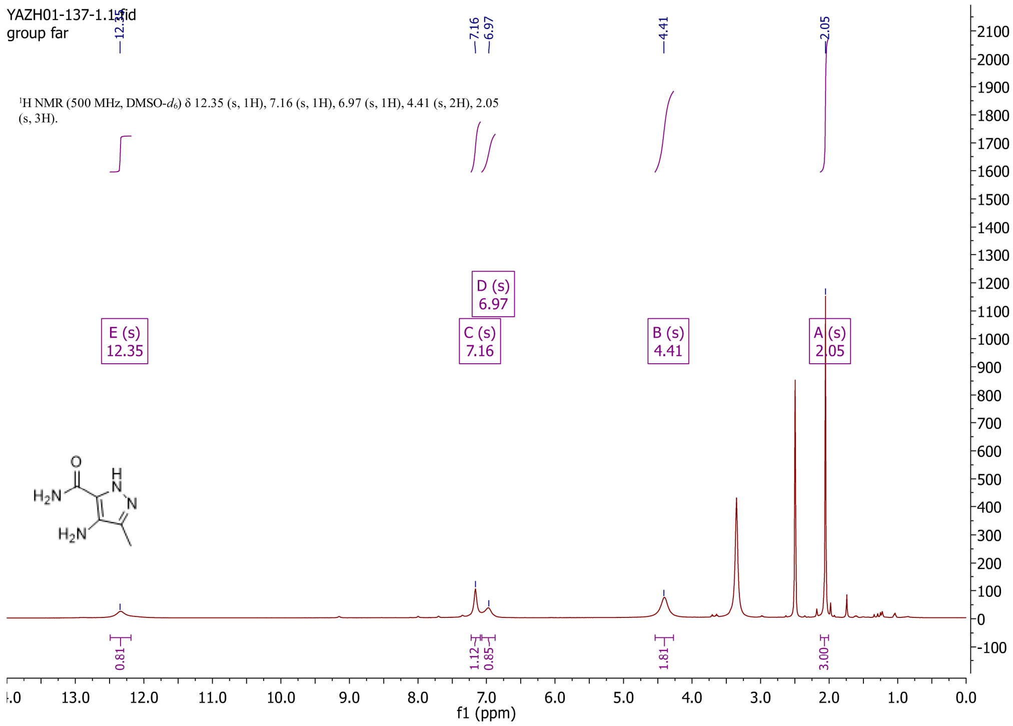

Figure S117. ^{1}H NMR spectrum of compound **30e**.

Acquired by	: Admin
Date Acquired	: 17/7/2018 3:20:53 PM
Sample Name	: LABA-025-ENDPR
Sample ID	:
Tray#	: 1
Vial#	: 32
Injection Volume	: 1
Data File	: C:\LabSolutions\Data\2018\2018-wk29\LABA-025-ENDPR.lcd
Background File	: blanco 17072018.lcd
Method File	: Method SCAN ACID standard.lcm
Report Format	: DefaultLCMS.lcr
Tuning File	: C:\LabSolutions\Tuning\Tuning-ESI-pos-neg01072015a.lct
Processed by	: Admin
Modified Date	: 17/7/2018 3:56:31 PM

Chromatogram
LABA-025-ENDPR C:\LabSolutions\Data\2018\2018-wk29\LABA-025-ENDPR.lcd

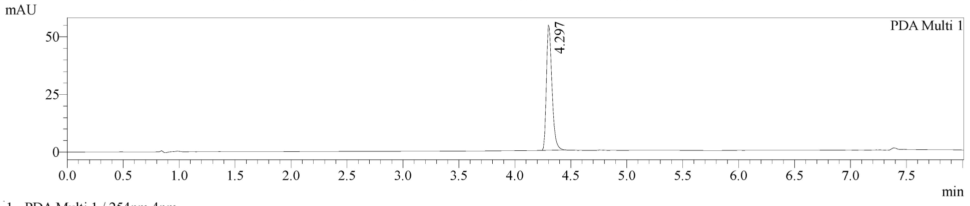

1 PDA Multi 1 / 254nm 4nm

Peak#	Name	Ret. Time	Area	Area %
1		4.297	187800	100.000

MS Chromatogram
LABA-025-ENDPR C:\LabSolutions\Data\2018\2018-wk29\LABA-025-ENDPR.lcd

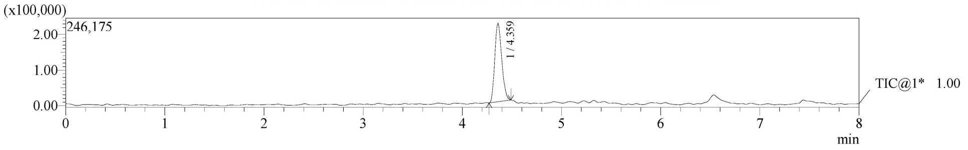

MS Spectrum Graph

#:1 Ret.Time:Averaged 4.350-4.370(Scan#:436-438)
Mass Peaks:12 Base Peak:299.15(141864) Polarity:Pos Segment1 - Event1

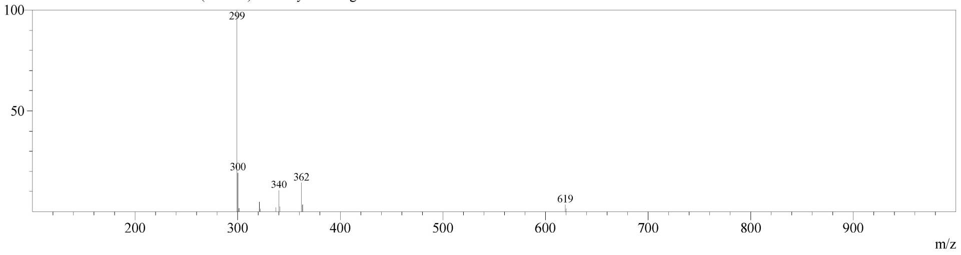

MS Spectrum Table

#:1 Ret.Time:
BG Mode:Calc 4.270<->4.490(428<->450)
Mass Peaks:12 Base Peak:299.15(141864) Polarity:Pos Segment1 - Event1

#	m/z	Abs.Inten.	Rel.Inten.	Charge	Polarity	Monoisotopic
1	299.15	141864	100.00			
2	300.15	27404	19.32			
3	301.35	2464	1.74			
4	321.10	6833	4.82			
5	322.15	2042	1.44			
6	337.15	2872	2.02			

#	m/z	Abs.Inten.	Rel.Inten.	Charge	Polarity	Monoisotopic
7	340.20	14881	10.49			
8	341.15	3574	2.52			
9	362.15	20335	14.33			
10	363.15	4944	3.49			
11	619.25	4795	3.38			
12	620.20	2208	1.56			

Figure S118. LCMS spectrum of compound **31a**.

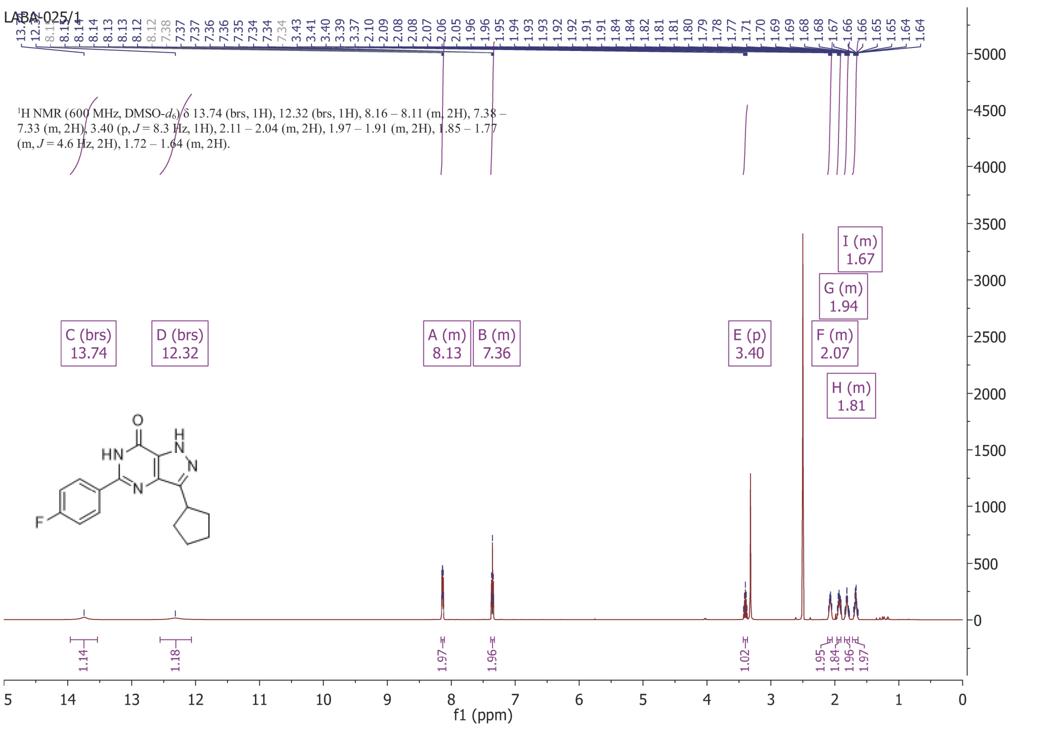

Figure S119. ^{1}H NMR spectrum of compound **31a**.

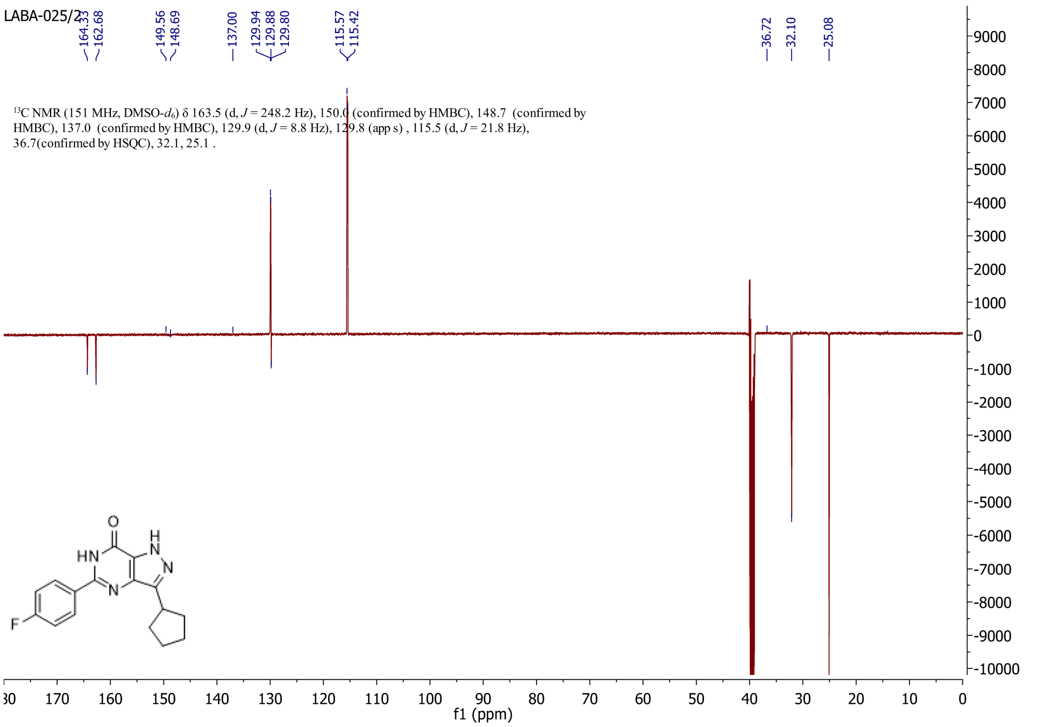

Figure S120. ^{13}C NMR spectrum of compound **31a**.

Acquired by	: Admin
Date Acquired	: 10/12/2018 9:07:44 AM
Sample Name	: ODVI01_044 3-6 10122018
Sample ID	:
Tray#	: 1
Vial#	: 2
Injection Volume	: 2
Data File	: C:\LabSolutions\Data\2018\2018-wk50\ODVI01_044 3-6 10122018.lcd
Background File	: blanco 10122018.lcd
Method File	: Method SCAN ACID standard.lcm
Report Format	: DefaultLCMS.lcr
Tuning File	: C:\LabSolutions\Tuning\Tuning-ESI-pos-neg01072015a.lct
Processed by	: Admin
Modified Date	: 10/12/2018 9:17:37 AM

Chromatogram
ODVI01_044 3-6 10122018 C:\LabSolutions\Data\2018\2018-wk50\ODVI01_044 3-6 10122018.lcd

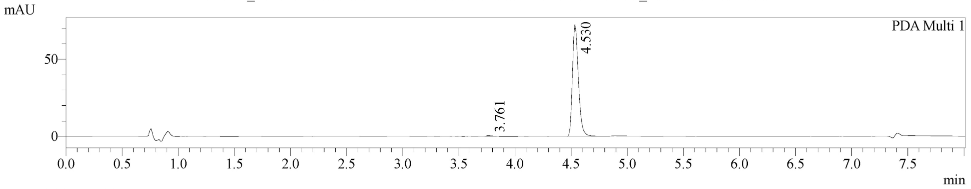

1 PDA Multi 1 / 254nm 4nm
PDA Ch1 254nm 4nm

Peak#	Name	Ret. Time	Area	Area %
1		3.761	942	0.351
2		4.530	266986	99.649

MS Chromatogram
ODVI01_044 3-6 10122018 C:\LabSolutions\Data\2018\2018-wk50\ODVI01_044 3-6 10122018.lcd

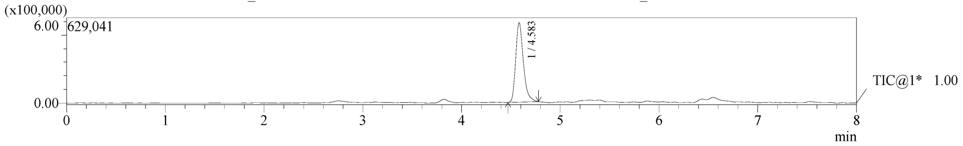

MS Spectrum Graph

#:1 Ret.Time:Averaged 4.570-4.590(Scan#:458-460)
Mass Peaks:12 Base Peak:313.10(356819) Polarity:Pos Segment1 - Event1

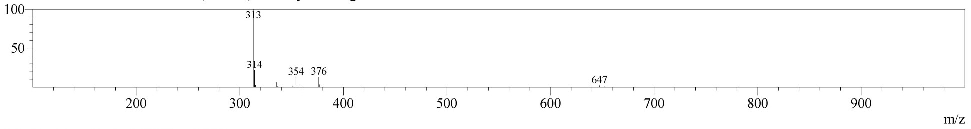

#:2 Ret.Time:Single 3.820(Scan#:383)
Mass Peaks:7 Base Peak:258.15(21219) Polarity:Pos Segment1 - Event1

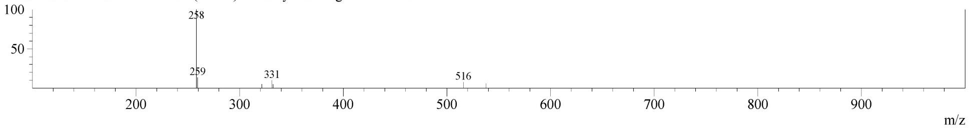

MS Spectrum Table

#:1 Ret.Time:
BG Mode:Calc 4.470<->4.780(448<->479)
Mass Peaks:12 Base Peak:313.10(356819) Polarity:Pos Segment1 - Event1

#	m/z	Abs.Inten.	Rel.Inten.	Charge	Polarity	Monoisotopic	#	m/z	Abs.Inten.	Rel.Inten.	Charge	Polarity	Monoisotopic
1	313.10	356819	100.00				7	354.15	43530	12.20			
2	314.10	75772	21.24				8	355.15	8991	2.52			
3	315.05	8093	2.27				9	376.10	44498	12.47			
4	335.15	21537	6.04				10	377.05	11658	3.27			
5	336.15	5160	1.45				11	647.20	6549	1.84			
6	351.15	6088	1.71				12	652.50	5918	1.66			

#:2 Ret.Time:
BG Mode:None
Mass Peaks:7 Base Peak:258.15(21219) Polarity:Pos Segment1 - Event1

#	m/z	Abs.Inten.	Rel.Inten.	Charge	Polarity	Monoisotopic	#	m/z	Abs.Inten.	Rel.Inten.	Charge	Polarity	Monoisotopic
1	258.15	21219	100.00				5	332.10	1029	4.85			
2	259.35	2860	13.48				6	515.80	1603	7.55			
3	321.25	1058	4.99				7	537.75	1177	5.55			
4	331.10	2095	9.87										

Figure S121. LCMS spectrum of compound **31b**.

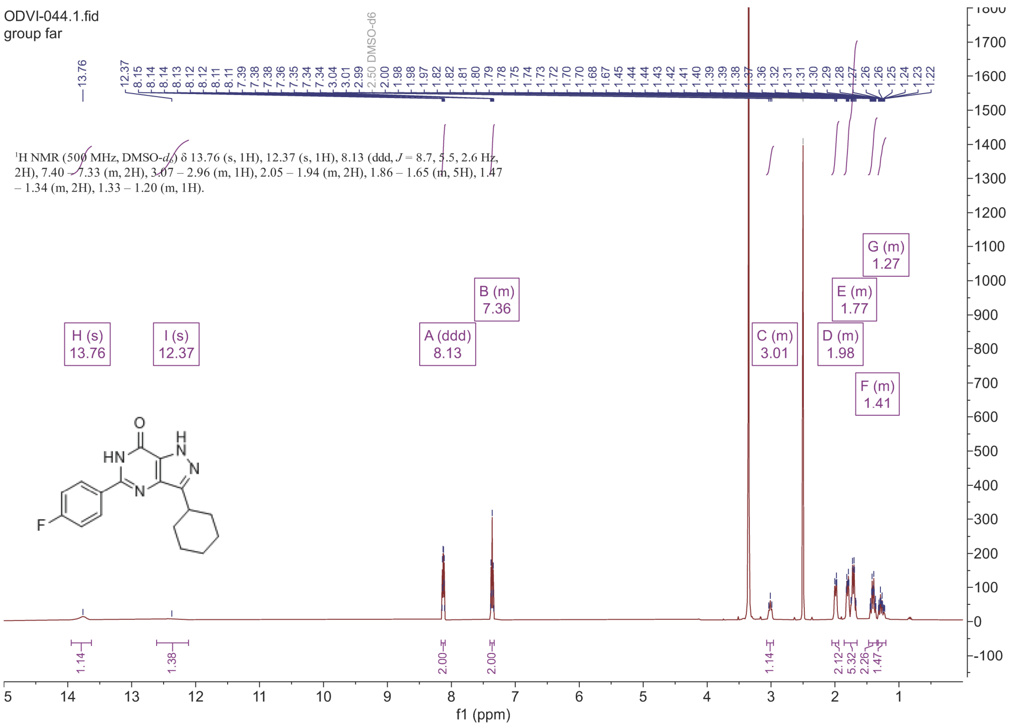

Figure S122. ¹H NMR spectrum of compound **31b**.

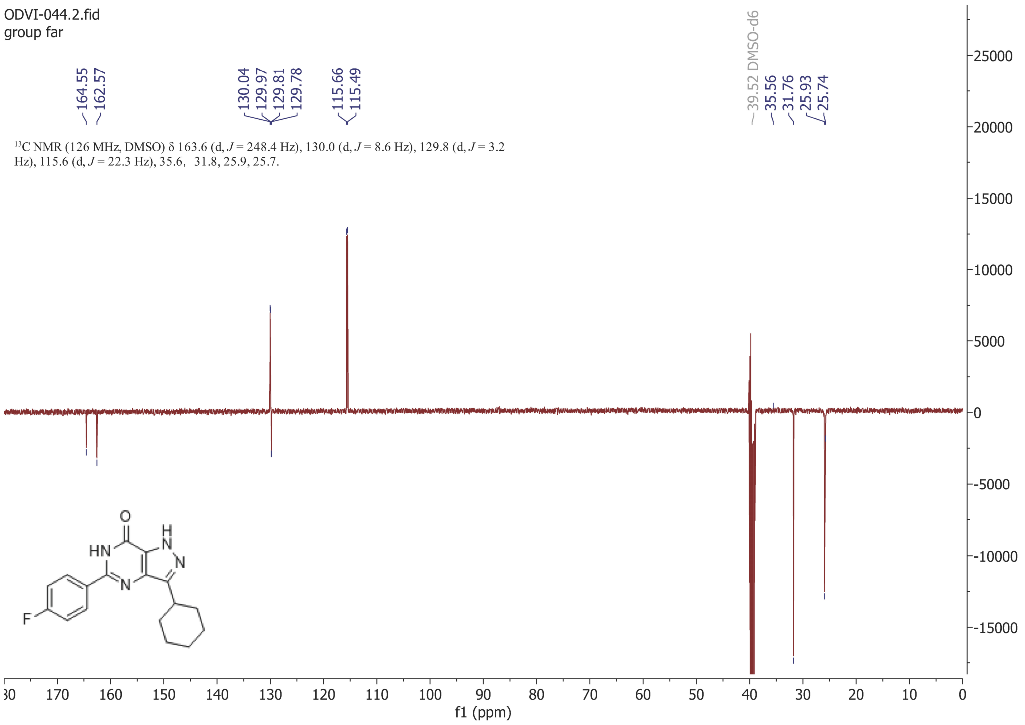

Figure S123. ¹³C NMR spectrum of compound **31b**.

Acquired by	: Admin
Date Acquired	: 7/8/2018 10:24:01 AM
Sample Name	: YAZH01-233
Sample ID	:
Tray#	: 1
Vial#	: 6
Injection Volume	: 3
Data File	: C:\LabSolutions\Data\2018\2018-wk32\YAZH01-233.lcd
Background File	: blanco 070818.lcd
Method File	: Method SCAN ACID standard.lcm
Report Format	: DefaultLCMS.lcr
Tuning File	: C:\LabSolutions\Tuning\Tuning-ESI-pos-neg01072015a.lct
Processed by	: Admin
Modified Date	: 7/8/2018 10:33:54 AM

Chromatogram
YAZH01-233 C:\LabSolutions\Data\2018\2018-wk32\YAZH01-233.lcd

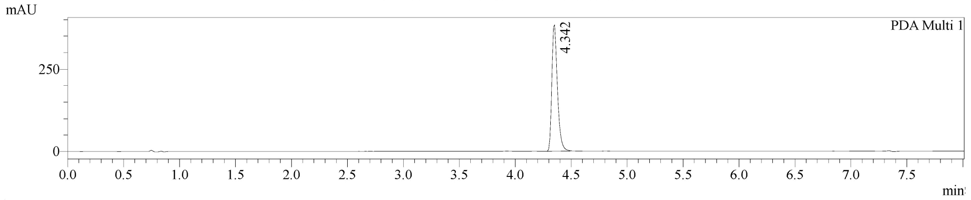

1 PDA Multi 1 / 254nm 4nm

Peak#	Name	Ret. Time	Area	Area %
1		4.342	1344980	100.000

MS Chromatogram
YAZH01-233 C:\LabSolutions\Data\2018\2018-wk32\YAZH01-233.lcd

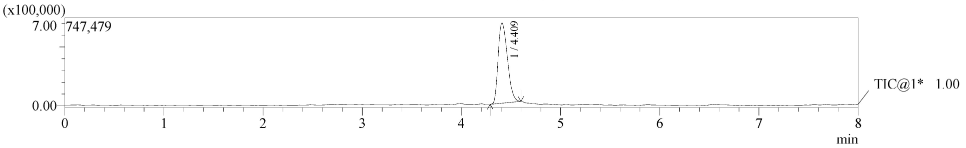

MS Spectrum Graph
#:1 Ret.Time:Averaged 4.400-4.420(Scan#:441-443)
Mass Peaks:15 Base Peak:287.15(323082) Polarity:Pos Segment1 - Event1

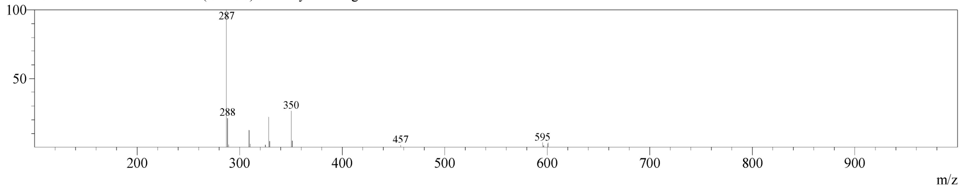

MS Spectrum Table

#:1 Ret.Time:
BG Mode:Calc 4.290<->4.600(430<->461)
Mass Peaks:15 Base Peak:287.15(323082) Polarity:Pos Segment1 - Event1

#	m/z	Abs.Inten.	Rel.Inten.	Charge	Polarity	Monoisotopic
1	287.15	323082	100.00			
2	288.15	68146	21.09			
3	289.25	5304	1.64			
4	309.10	40327	12.48			
5	310.10	7211	2.23			
6	325.05	5165	1.60			
7	328.15	70804	21.92			
8	329.15	14802	4.58			
9	350.10	85019	26.31			
10	351.20	15835	4.90			
11	456.95	4410	1.36			
12	595.40	10554	3.27			
13	596.45	3293	1.02			
14	600.45	7979	2.47			
15	600.70	10022	3.10			

Figure S124. LCMS spectrum of compound **31c**.

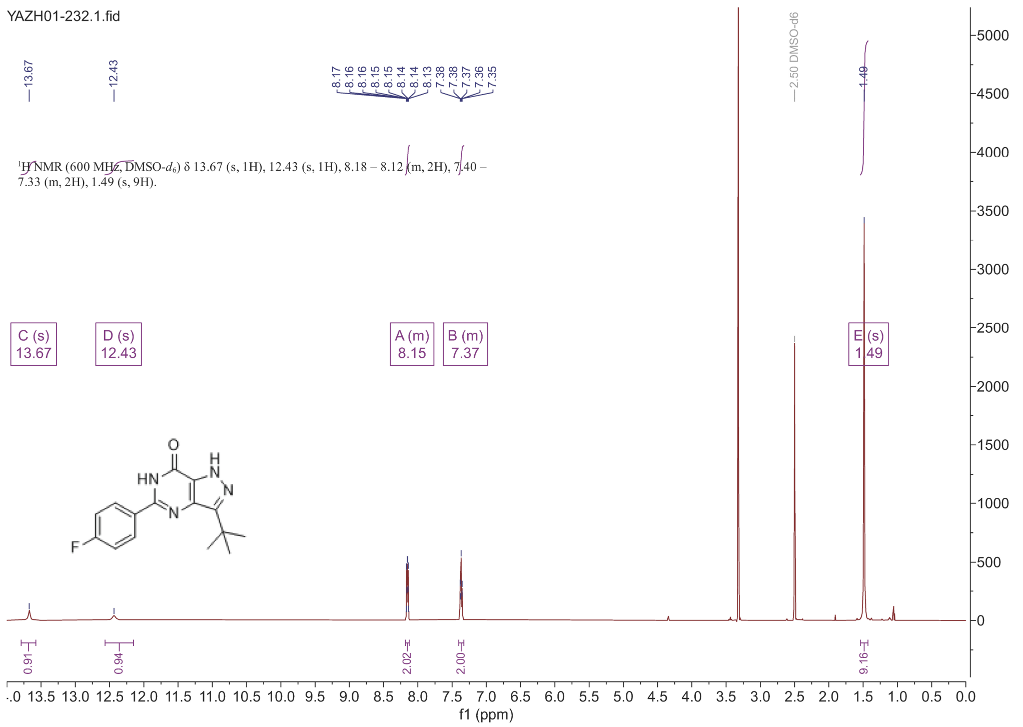

Figure S125. ^{1}H NMR spectrum of compound **31c**.

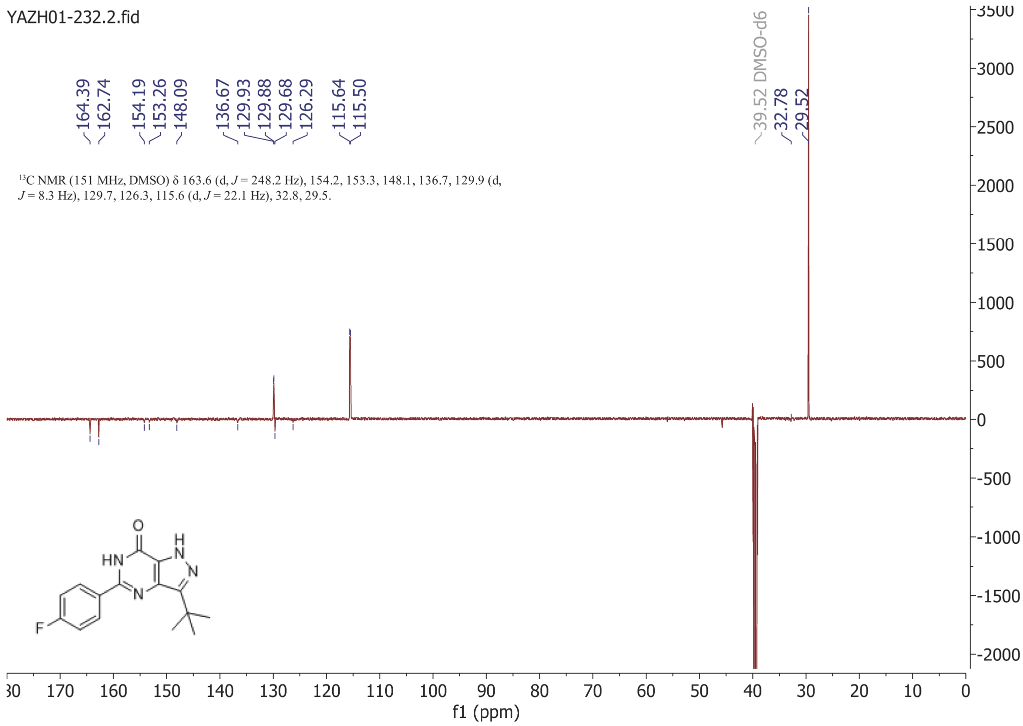

Figure S126. ^{13}C NMR spectrum of compound **31c**.

Acquired by	: Admin
Date Acquired	: 27/6/2018 12:16:59 PM
Sample Name	: YAZH01-221
Sample ID	:
Tray#	: 1
Vial#	: 46
Injection Volume	: 3
Data File	: C:\LabSolutions\Data\2018\2018-wk26\YAZH01-221.lcd
Background File	: blanco 27062018.lcd
Method File	: Method SCAN ACID standard.lcm
Report Format	: DefaultLCMS.lcr
Tuning File	: C:\LabSolutions\Tuning\Tuning-ESI-pos-neg01072015a.lct
Processed by	: Admin
Modified Date	: 27/6/2018 12:35:32 PM

Chromatogram

YAZH01-221 C:\LabSolutions\Data\2018\2018-wk26\YAZH01-221.lcd

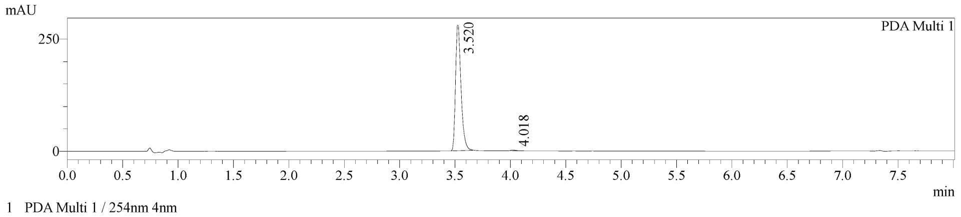

1 PDA Multi 1 / 254nm 4nm

PeakTable

PDA Ch1 254nm 4nm

Peak#	Name	Ret. Time	Area	Area %
1		3.520	963705	99.477
2		4.018	5062	0.523

MS Chromatogram

YAZH01-221 C:\LabSolutions\Data\2018\2018-wk26\YAZH01-221.lcd

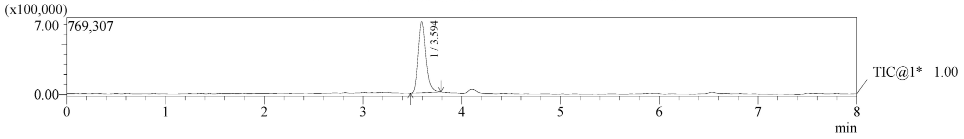

MS Spectrum Graph

#:1 Ret.Time:Averaged 3.580-3.600(Scan#:359-361)

Mass Peaks:10 Base Peak:259.10(451571) Polarity:Pos Segment1 - Event1

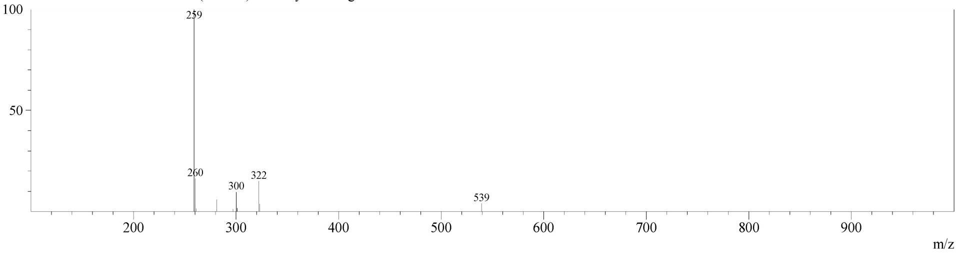

MS Spectrum Table

#:1 Ret.Time:

BG Mode:Calc 3.480<->3.790(349<->380)

Mass Peaks:10 Base Peak:259.10(451571) Polarity:Pos Segment1 - Event1

#	m/z	Abs.Inten.	Rel.Inten.	Charge	Polarity	Monoisotopic
1	259.10	451571	100.00			
2	260.10	73084	16.18			
3	261.05	7254	1.61			
4	281.10	27348	6.06			
5	297.00	6123	1.36			
6	300.15	43860	9.71			
7	301.15	8388	1.86			
8	322.15	67636	14.98			
9	323.05	17785	3.94			
10	539.30	18577	4.11			

Figure S127. LCMS spectrum of compound **31d**.

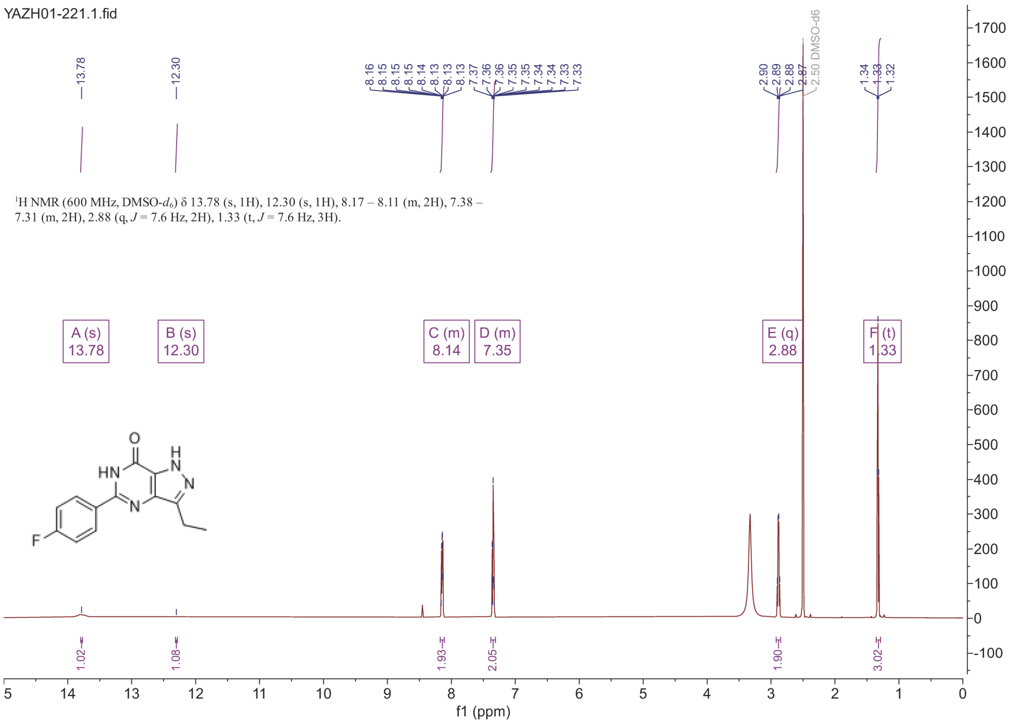

Figure S128. ^{1}H NMR spectrum of compound **31d**.

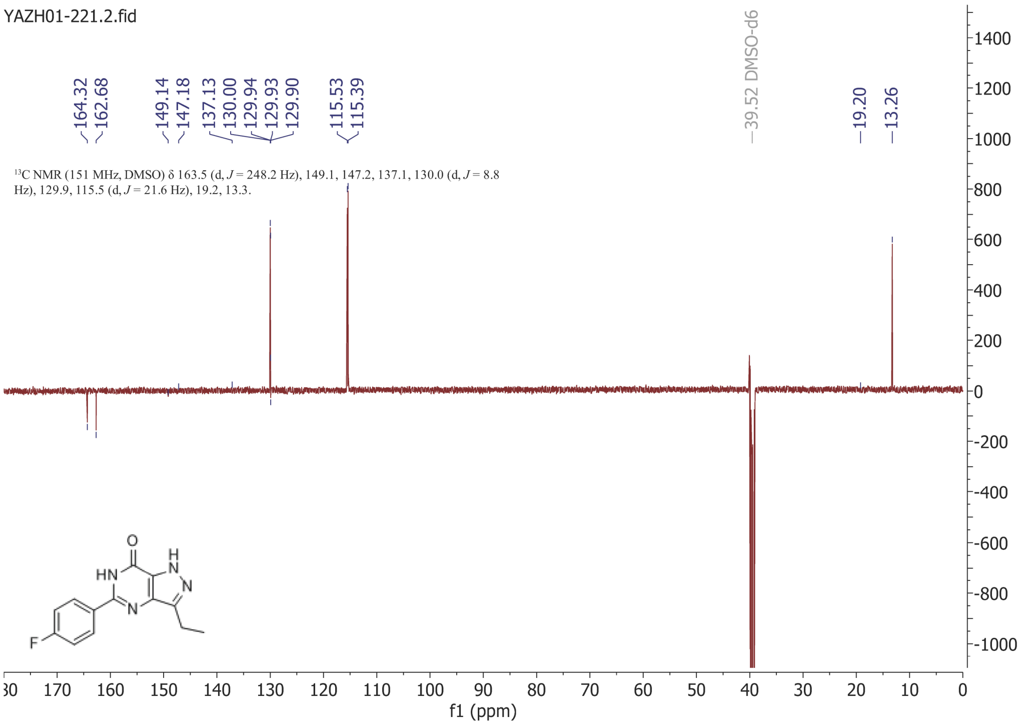

Figure S129. ^{13}C NMR spectrum of compound **31d**.

Acquired by	: Admin
Date Acquired	: 20/10/2017 4:13:41 PM
Sample Name	: YAZH01-153-4
Sample ID	:
Tray#	: 1
Vial#	: 14
Injection Volume	: 10
Data File	: C:\LabSolutions\Data\2017\2017-wk42\YAZH01-153-4.lcd
Background File	: blanco 20102017.lcd
Method File	: Method SCAN ACID standard.lcm
Report Format	: DefaultLCMS.lcr
Tuning File	: C:\LabSolutions\Tuning\Tuning-ESI-pos-neg01072015a.lct
Processed by	: Admin
Modified Date	: 7/6/2019 5:51:55 PM

PDA Graph

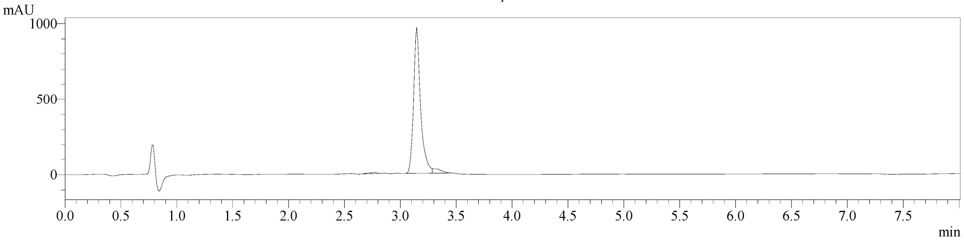

PeakTable

PDA Ch1 230nm 4nm

Peak#	Name	Ret. Time	Area	Area %
1		2.755	39494	0.905
2		3.140	4178158	95.689
3		3.296	148718	3.406

MS Chromatogram
YAZH01-153-4 C:\LabSolutions\Data\2017\2017-wk41-51\2017-wk42\YAZH01-153-4.lcd

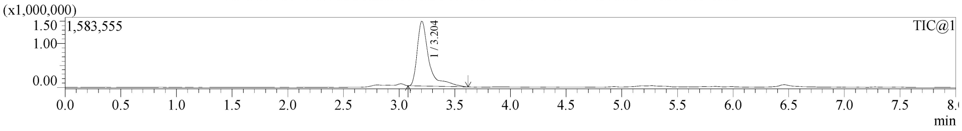

MS Spectrum Graph

#:1 Ret.Time:Averaged 3.190-3.210(Scan#:320-322)
BG Mode:Calc 3.080<->3.620(309<->363)
Mass Peaks:14 Base Peak:313.10(616217) Polarity:Pos Segment1 - Event1

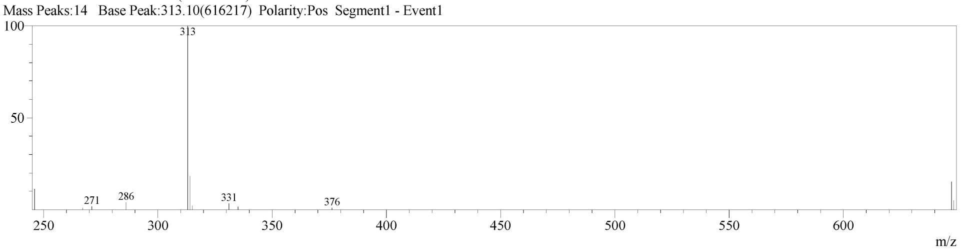

MS Spectrum Table

#:1 Ret.Time:
BG Mode:Calc 3.080<->3.620(309<->363)
Mass Peaks:14 Base Peak:313.10(616217) Polarity:Pos Segment1 - Event1

#	m/z	Abs.Inten.	Rel.Inten.	Charge	Polarity	Monoisotopic
1	245.05	450100	73.04			
2	246.05	69475	11.27			
3	267.10	6364	1.03			
4	271.10	11398	1.85			
5	286.05	24835	4.03			
6	313.10	616217	100.00			
7	314.10	112209	18.21			
8	315.15	13252	2.15			
9	331.10	20953	3.40			
10	335.10	10290	1.67			
11	376.20	6628	1.08			
12	647.35	93552	15.18			
13	648.35	31493	5.11			
14	649.45	6417	1.04			

Figure S130. LCMS spectrum of compound **31e**.

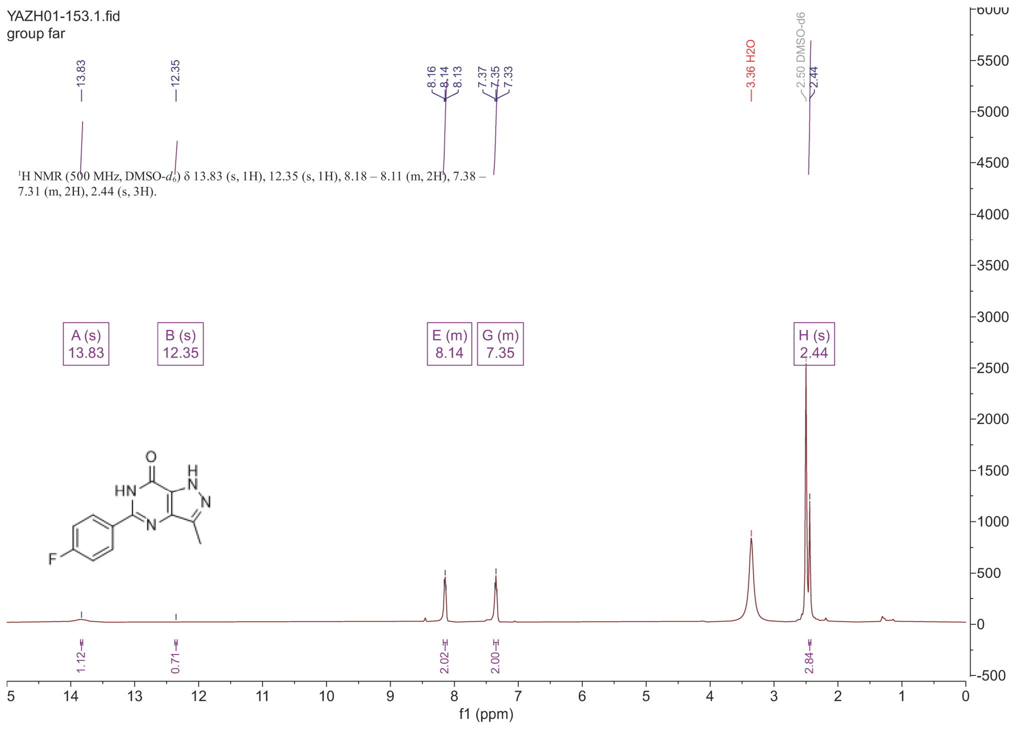

Figure S131. ^{1}H NMR spectrum of compound **31e**.

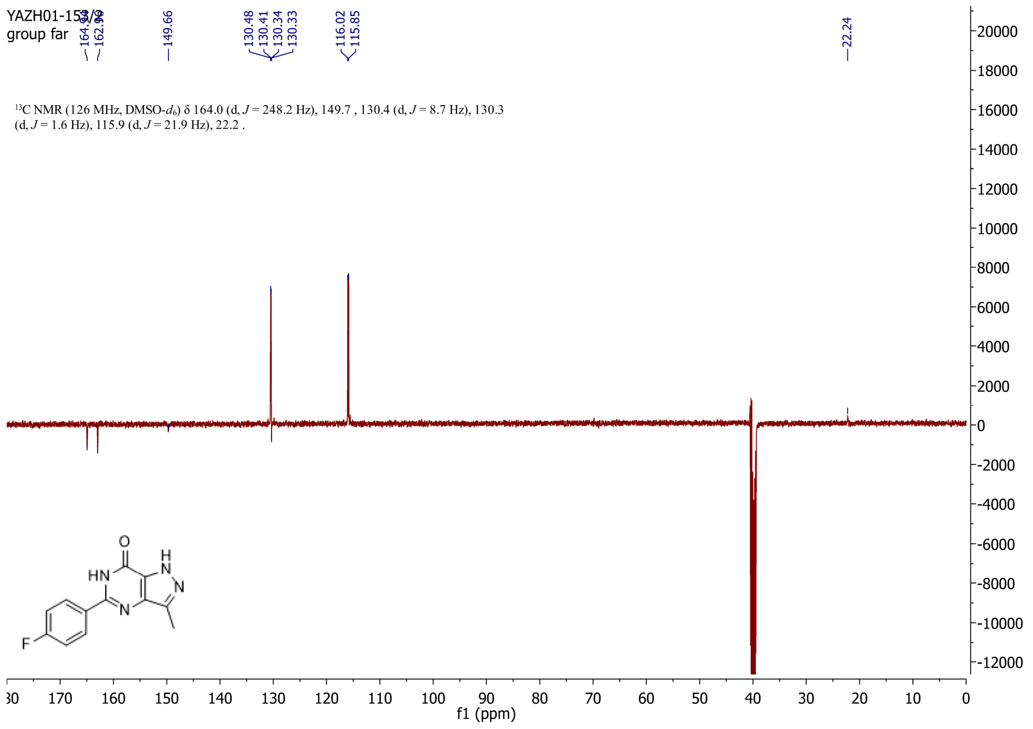

Figure S132. ^{13}C NMR spectrum of compound **31e**.

Acquired by	: Admin
Date Acquired	: 18/6/2019 3:39:49 PM
Sample Name	: VUF16375
Sample ID	:
Tray#	: 1
Vial#	: 35
Injection Volume	: 5
Data File	: C:\LabSolutions\Data\2019\2019-wk25\VUF16375.lcd
Background File	: blanco 18062019.lcd
Method File	: Method SCAN ACID standard.lcm
Report Format	: DefaultLCMS.lcr
Tuning File	: C:\LabSolutions\Tuning\Tuning-ESI-pos-neg01072015a.lct
Processed by	: Admin
Modified Date	: 18/6/2019 3:51:25 PM

PDA Graph

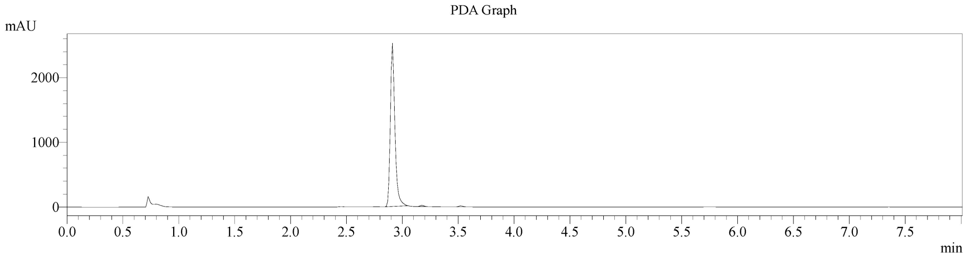

PeakTable

PDA Ch1 254nm 4nm

Peak#	Name	Ret. Time	Area	Area %
1		2.906	7092524	98.714
2		3.170	46741	0.651
3		3.517	45662	0.636

MS Chromatogram
VUF16375 C:\LabSolutions\Data\2019\2019-wk25\VUF16375.lcd

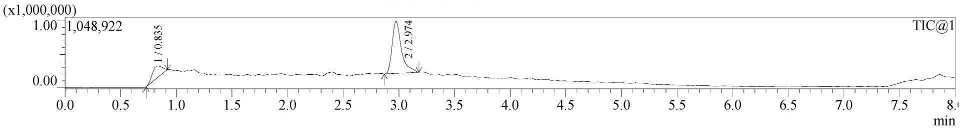

MS Spectrum Graph

#:1 Ret.Time:Averaged 2.960-2.980(Scan#:297-299)
BG Mode:Calc 2.870<->3.180(288<->319)
Mass Peaks:17 Base Peak:231.00(441377) Polarity:Pos Segment1 - Event1

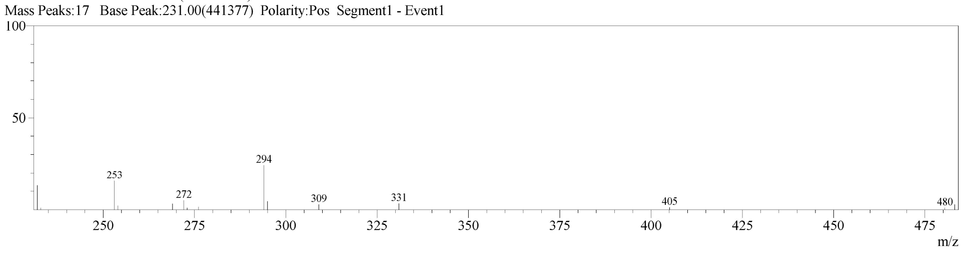

MS Spectrum Table

#:1 Ret.Time:
BG Mode:Calc 2.870<->3.180(288<->319)
Mass Peaks:17 Base Peak:231.00(441377) Polarity:Pos Segment1 - Event1

#	m/z	Abs.Inten.	Rel.Inten.	Charge	Polarity	Monoisotopic
1	231.00	441377	100.00			
2	231.95	58109	13.17			
3	232.90	4546	1.03			
4	253.05	69571	15.76			
5	254.05	9430	2.14			
6	269.00	14447	3.27			
7	272.05	22163	5.02			
8	273.00	4580	1.04			
9	276.10	6628	1.50			

#	m/z	Abs.Inten.	Rel.Inten.	Charge	Polarity	Monoisotopic
10	294.00	107749	24.41			
11	294.95	19940	4.52			
12	309.00	12625	2.86			
13	330.95	14689	3.33			
14	405.10	5598	1.27			
15	480.45	4520	1.02			
16	483.15	12558	2.85			
17	484.15	4810	1.09			

Figure S133. LCMS spectrum of compound **31f**.

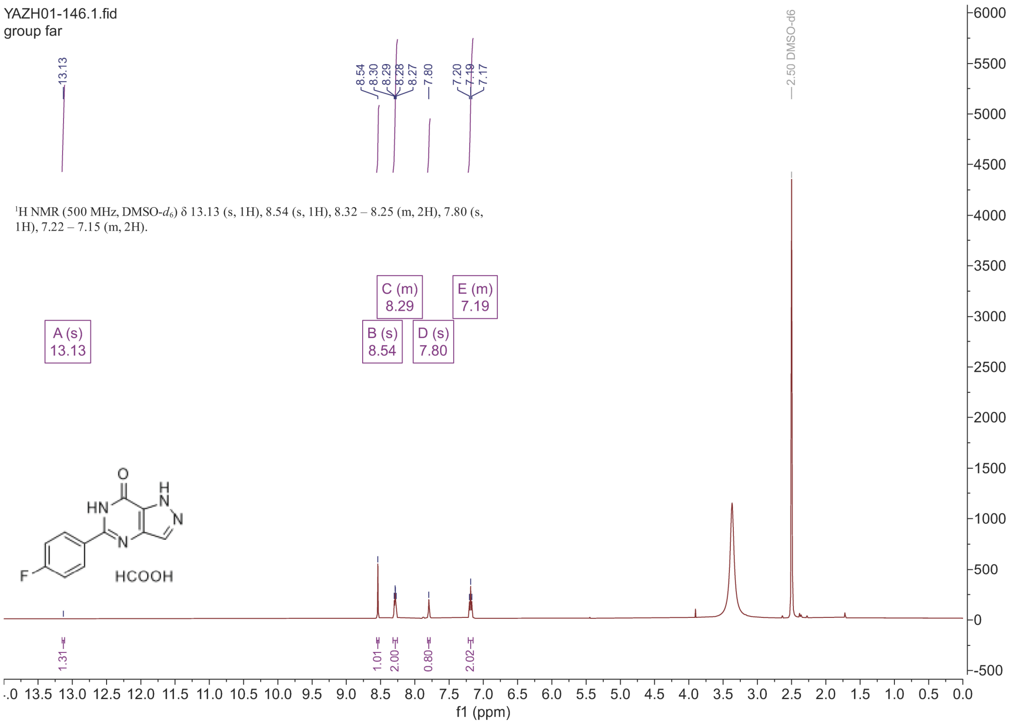

Figure S134. ^{1}H NMR spectrum of compound **31f**.

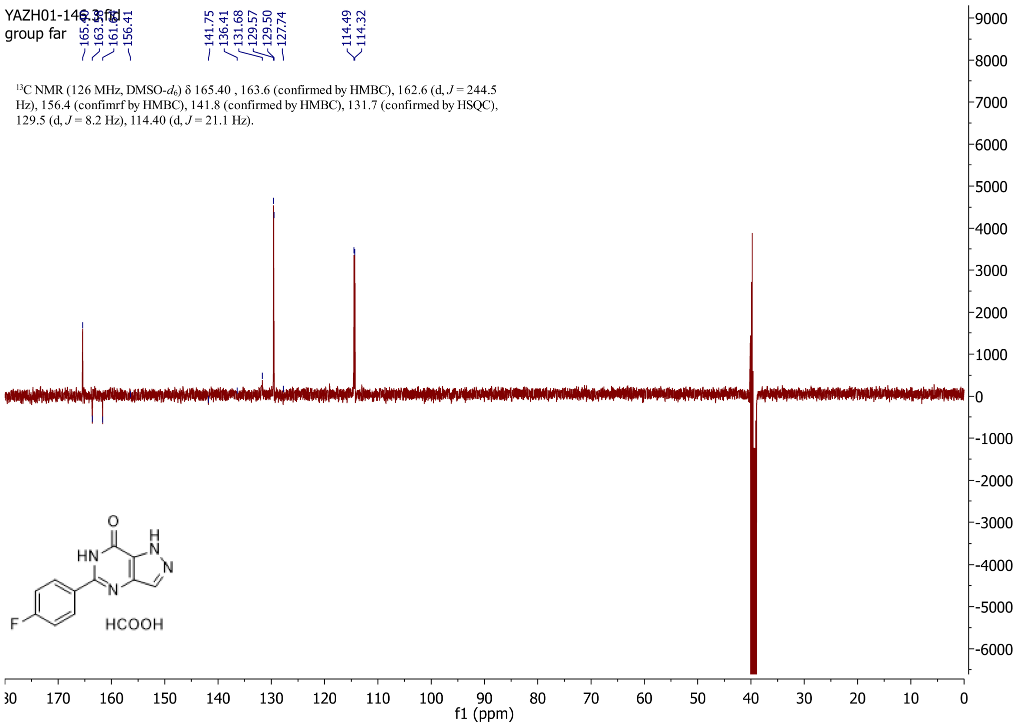

Figure S135. ^{13}C NMR spectrum of compound **31f**.

Acquired by	: Admin
Date Acquired	: 21/2/2019 2:41:40 PM
Sample Name	: LABI-009-NANP
Sample ID	:
Tray#	: 1
Vial#	: 11
Injection Volume	: 1
Data File	: C:\LabSolutions\Data\2019\2019-wk07\LABI-009-NANP.lcd
Background File	: blanco 21022019.lcd
Method File	: Method SCAN ACID standard.lcm
Report Format	: DefaultLCMS.lcr
Tuning File	: C:\LabSolutions\Tuning\Tuning-ESI-pos-neg01072015a.lct
Processed by	: Admin
Modified Date	: 19/7/2019 12:06:34 PM

Chromatogram
LABI-009-NANP C:\LabSolutions\Data\2019\2019-wk01-10\2019-wk08\LABI-009-NANP.lcd

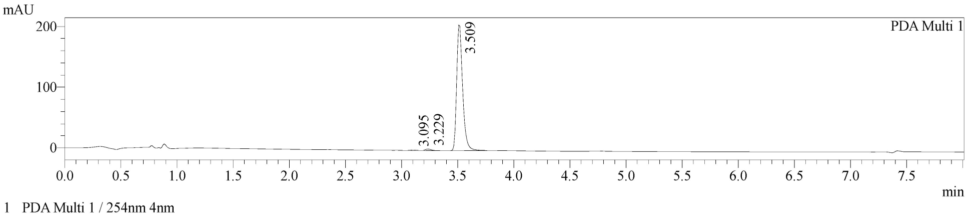

1 PDA Multi 1 / 254nm 4nm

PeakTabl

PDA Ch1 254nm 4nm

Peak#	Name	Ret. Time	Area	Area %
1		3.095	2251	0.291
2		3.229	8753	1.130
3		3.509	763389	98.579

MS Chromatogram
LABI-009-NANP C:\LabSolutions\Data\2019\2019-wk01-10\2019-wk08\LABI-009-NANP.lcd

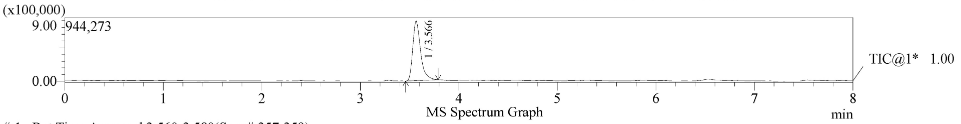

MS Spectrum Graph

#:1 Ret.Time:Averaged 3.560-3.580(Scan#:357-359)
Mass Peaks:13 Base Peak:232.05(477051) Polarity:Pos Segment1 - Event1

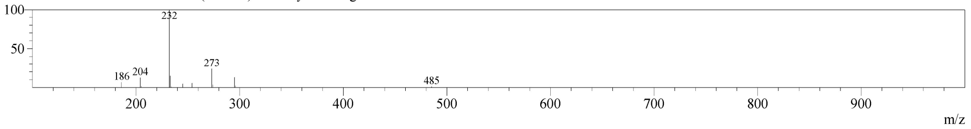

MS Spectrum Table

#:1 Ret.Time:
BG Mode:Calc 3.460<->3.790(347<->380)
Mass Peaks:13 Base Peak:232.05(477051) Polarity:Pos Segment1 - Event1

#	m/z	Abs.Inten.	Rel.Inten.	Charge	Polarity	Monoisotopic
1	185.90	31347	6.57			
2	204.05	59882	12.55			
3	205.05	7954	1.67			
4	232.05	477051	100.00			
5	233.05	70683	14.82			
6	234.05	5072	1.06			
7	245.05	22245	4.66			
8	254.05	26742	5.61			
9	273.10	113864	23.87			
10	274.10	16207	3.40			
11	295.10	62053	13.01			
12	296.05	10037	2.10			
13	485.25	5272	1.11			

Figure S136. LCMS spectrum of compound **33**.

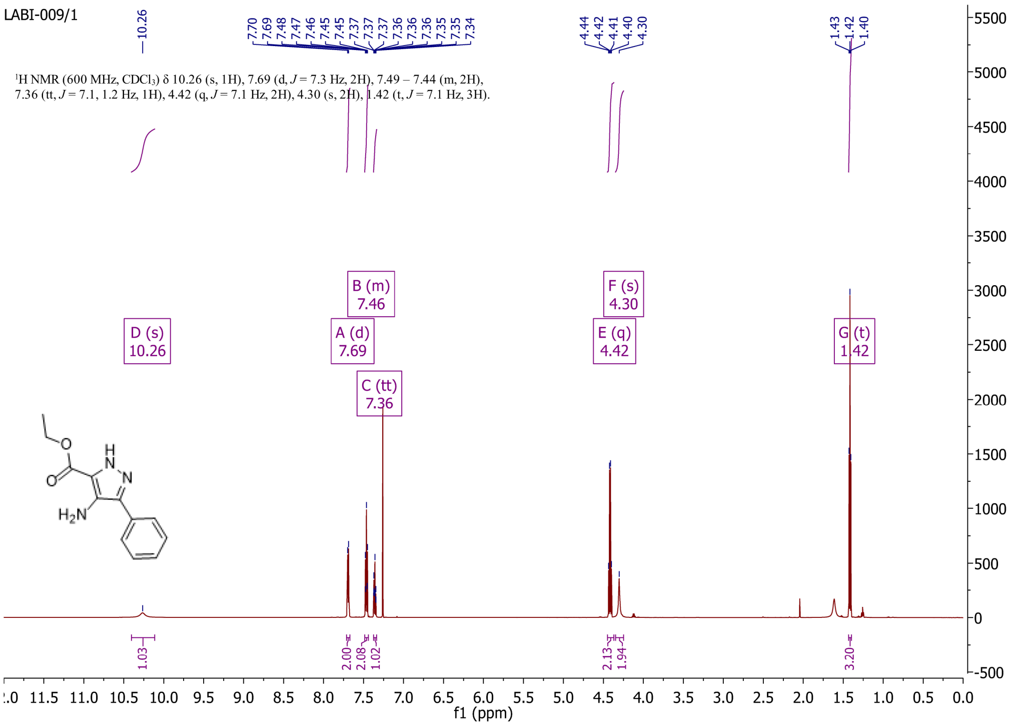

Figure S137. ^{1}H NMR spectrum of compound **33**.

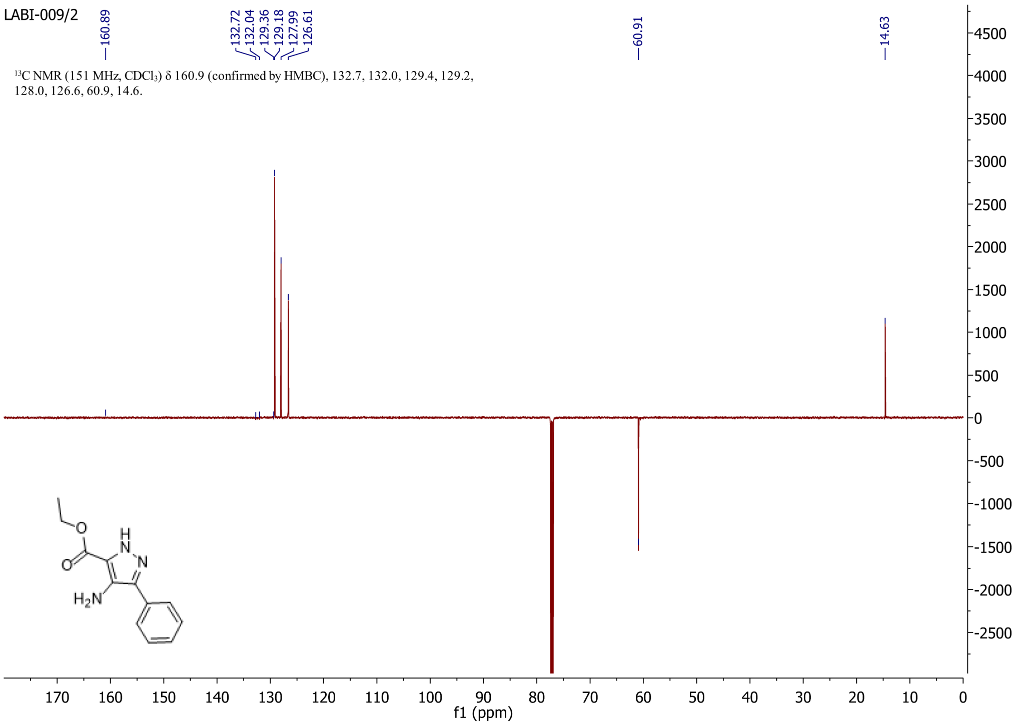

Figure S138. ^{13}C NMR spectrum of compound **33**.

Acquired by	: Admin
Date Acquired	: 18/3/2019 2:12:47 PM
Sample Name	: LABI-22-FINAL-2
Sample ID	:
Tray#	: 1
Vial#	: 34
Injection Volume	: 3
Data File	: C:\LabSolutions\Data\2019\2019-wk11\LABI-22-FINAL-2.lcd
Background File	: blanco 18032019.lcd
Method File	: Method SCAN ACID standard.lcm
Report Format	: DefaultLCMS.lcr
Tuning File	: C:\LabSolutions\Tuning\Tuning-ESI-pos-neg01072015a.lct
Processed by	: Admin
Modified Date	: 18/3/2019 2:23:04 PM

Chromatogram
LABI-22-FINAL-2 C:\LabSolutions\Data\2019\2019-wk11\LABI-22-FINAL-2.lcd

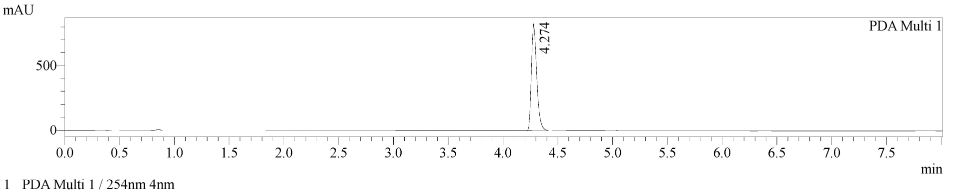

1 PDA Multi 1 / 254nm 4nm

PeakTable

PDA Ch1 254nm 4nm

Peak#	Name	Ret. Time	Area	Area %
1		4.274	2829474	100.000

MS Chromatogram
LABI-22-FINAL-2 C:\LabSolutions\Data\2019\2019-wk11\LABI-22-FINAL-2.lcd

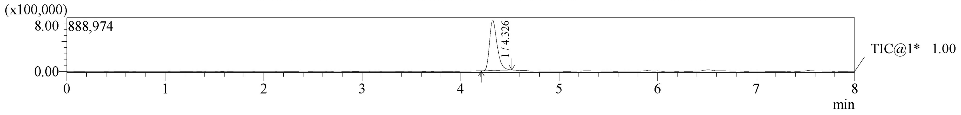

MS Spectrum Graph

#:1 Ret.Time:Averaged 4.320-4.340(Scan#:433-435)
Mass Peaks:12 Base Peak:307.05(475327) Polarity:Pos Segment1 - Event1

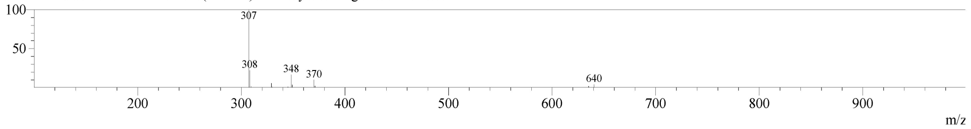

MS Spectrum Table

#:1 Ret.Time:
BG Mode:Calc 4.210<->4.520(422<->453)
Mass Peaks:12 Base Peak:307.05(475327) Polarity:Pos Segment1 - Event1

#	m/z	Abs.Inten.	Rel.Inten.	Charge	Polarity	Monoisotopic
1	307.05	475327	100.00			
2	308.05	105556	22.21			
3	309.40	8327	1.75			
4	328.95	28045	5.90			
5	330.00	5891	1.24			
6	344.95	5676	1.19			
7	348.10	79010	16.62			
8	349.10	18277	3.85			
9	370.10	47873	10.07			
10	371.10	11539	2.43			
11	635.20	9222	1.94			
12	640.50	20656	4.35			

Figure S139. LCMS spectrum of compound **35**.

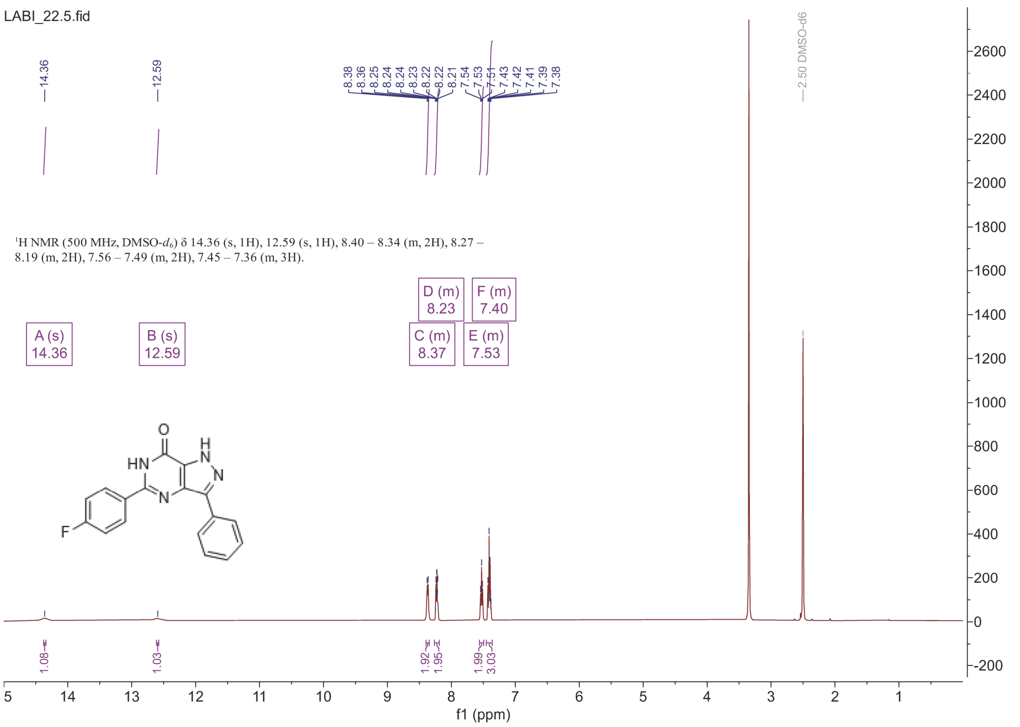

Figure S140. ¹H NMR spectrum of compound **35**.

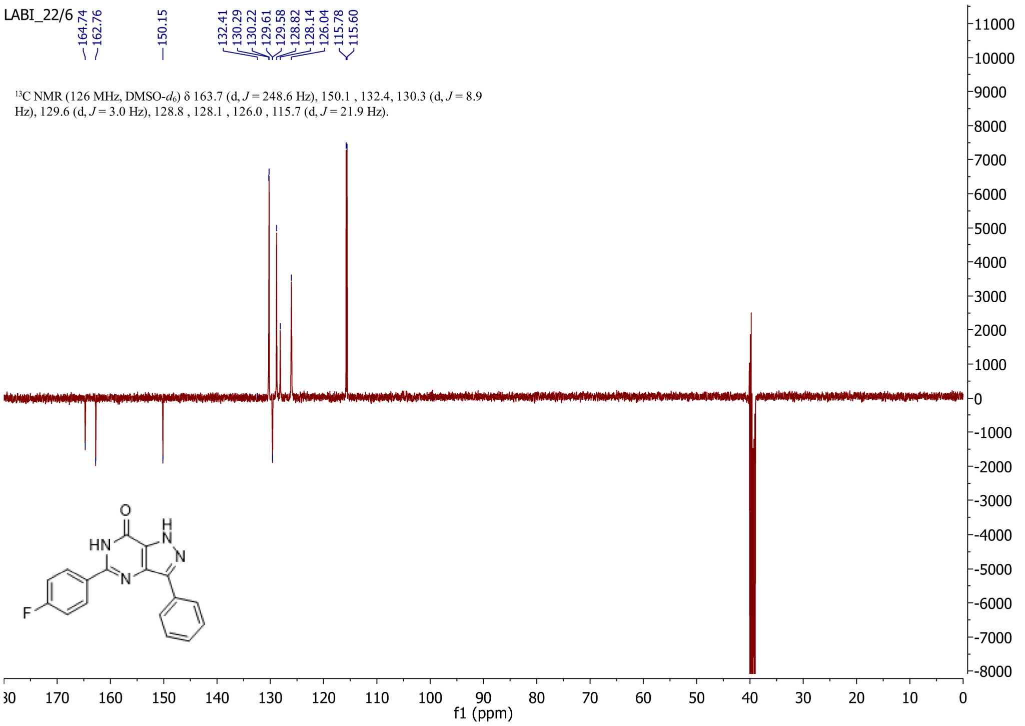

Figure S141. ¹³C NMR spectrum of compound **35**.
